# Scalable thioarylation of unprotected peptides and biomolecules under Ni/photoredox catalysis†
†Electronic supplementary information (ESI) available. See DOI: 10.1039/c7sc04292b


**DOI:** 10.1039/c7sc04292b

**Published:** 2017-11-13

**Authors:** Brandon A. Vara, Xingpin Li, Simon Berritt, Christopher R. Walters, E. James Petersson, Gary A. Molander

**Affiliations:** a Roy and Diana Vagelos Laboratories , Department of Chemistry , University of Pennsylvania , 231 South 34th Street , Philadelphia , Pennsylvania 19104-6323 , USA . Email: gmolandr@sas.upenn.edu

## Abstract

Site-specific functionalization of unprotected native peptides and biomolecules remains a useful transformation in synthetic design and chemical biology, yet until recently, advancements in transition metal-catalyzed methods, which have prevailed in organic synthesis, have been relatively ineffective when applied to large and structurally complex biomolecules. Here, the mechanistically distinct, Ni/photoredox-catalyzed arylation of unprotected, native thiols (*e.g.*, cysteine residues) is reported – a process initiated through a visible light-promoted, hydrogen atom transfer (HAT) event under ambient conditions. Sub-stoichiometric loadings of the dual-catalyst system (≤5 mol%) are employed, granting excellent site-specificity, broad substrate scope, and low chemical waste. Reaction scalability (from μg to grams) has been achieved through modest reagent adjustments, and high throughput experimentation (HTE) demonstrates the ease of reaction setup, enabling prompt screening of aryl halide coupling partners and conditions. Scores of thiol substrates and aryl entities were examined and effectively conjugated, suggesting further diverse, practical applications.

## Abstract 2

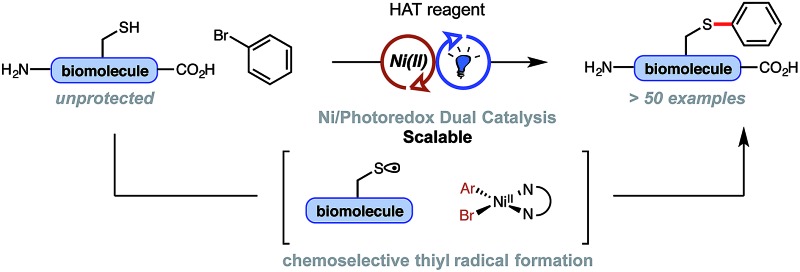
A mechanistically distinct, Ni/photoredox-catalyzed arylation of unprotected, native thiols (*e.g.*, cysteine residues) is reported – a process initiated through a visible light-promoted, hydrogen atom transfer (HAT) event under ambient conditions.

## Introduction

Transition metal-catalyzed cross-couplings are an undisputable staple in synthetic chemistry, yet granting widespread substrate and functional group tolerance to such a critical class of reactions remains an overarching goal of much synthetic effort. Prominent among these are carbon–carbon and carbon–heteroatom (primarily O, N, and S) bond-forming reactions, which use (pro)nucleophilic species primed for transmetalation largely following historically similar, ionic (two-electron) mechanistic cycles in the presence of base. Within these classical paradigms, protecting group chemistry may be required when designing multi-step syntheses for complex molecules, especially peptides and other biomolecules, resulting in increased waste and poor chemical and step economy.^1^ Consequently, efficient cross-coupling reactions conducted on natural, unmodified biological substrates remain scarce.^2^,^3^ By contrast, a Ni/photoredox dual-catalyzed cross-coupling event driven by an overall redox-neutral process initiated by photon absorption (*via* focused visible light) can be employed within complex systems, orchestrated by catalytic amounts of photosensitizers and the selective pairing of single electron oxidation and reduction potentials with targeted reagents. As encouraging as the burgeoning field of radical photoredox chemistry has been, a mild and catalytic cross-coupling protocol in concert with native, unprotected biomolecules through a single electron transfer (SET) manifold has yet to be realized.

Cysteine (Cys) sulfhydryl (R–SH) moieties are critical handles for chemical manipulation in bioconjugations, native chemical ligation, and peptide chemistry broadly, primarily because of the high relative nucleophilicity of sulfur and its low natural abundance in peptides and proteins.^4^ For example, nonmetal-catalyzed Michael reactions and maleimide conjugations *via* reactive thiolate intermediates predominate, even when considering that the resulting thioether (Csp^3^–S) linkages can be labile and chemically reversible (*via* retro-Michael reactions) in physiological or basic environments.^5^ This may be particularly problematic when designing clinical antibody–drug conjugates^6^ or biological probes. Radical photo-crosslinking^7^ of biological thiols and related open-shell transformations are well documented,^8^ but incorporation of small molecules outside of thiol–ene/yne chemistry remain under-studied.^9^ Accessing more resilient, irreversible aryl sulfide linkages [Csp^2^–S] from peptidic alkyl sulfhydryl groups presents a challenging, yet desirable, opportunity in synthetic cross-coupling chemistry to introduce a vast range of functional, aromatic small molecules.^10^ The myriad protic, Lewis basic, and thiol functional groups inherent to native biomolecules have notoriously complicated applications of transition metal catalysis,^11^ leading to catalyst deactivation, undesired cross reactivity, and complex reaction mixtures. Given these challenges, a reaction of practical significance would require mild, aqueous, and dilute reaction conditions near neutral pH (pH 6–8), favorable reaction kinetics, and highly chemoselective reagents without production of excessive waste.

Arylative cross-coupling of cysteine residues in peptides has been recently documented using finely tuned palladium reagents in aqueous environments. Although innovative and uniquely important for protein elaboration, these conditions typically require superstoichiometric, pre-complexed palladium reagents (2–10 equiv. Pd)^12^ or a large excess of an aryl halide (up to 500 equiv. of aryl iodide),^13^ and therefore may not be economically feasible or practical when applied to larger scale syntheses of small molecules and peptides. Ullmann C–S coupling strategies have made impressive advances, yet still generally require high temperatures and protected substrates.^14^ Separately, nonmetal-catalyzed, “thiol-click” arylations^15^ and S_N_Ar reactions employ electron deficient, perfluorinated arenes, with useful applications in thiopeptide stapling,^16^ but are inherently limited in arene scope and additional applications. A single, elegant example detailing the reductive coupling of unprotected thioglycosides with aryl iodides using Ni catalysis through a two-electron process has also been reported.^17^ Finally, while the current contribution was under review, Noël and coworkers reported a method for cysteine arylation under photoredox conditions using *in situ* generated aryldiazonium salts as the reactive partners.^18^ The reactions were performed in both batch and flow, but the nature of this transformation required protection of all amine functional groups unless isolated diazonium salts were used in the reactions, in which case 10 equivalents of this reacting partner were required. The largest scale attempted was 0.25 mmol. A welcome advance to the thiolation of peptides and biomolecules would employ non-precious metals in catalytic stoichiometry, simple or no ligands, high chemoselectivity, inexpensive and available starting materials, and broad functional group tolerance.

**Fig. 1:**
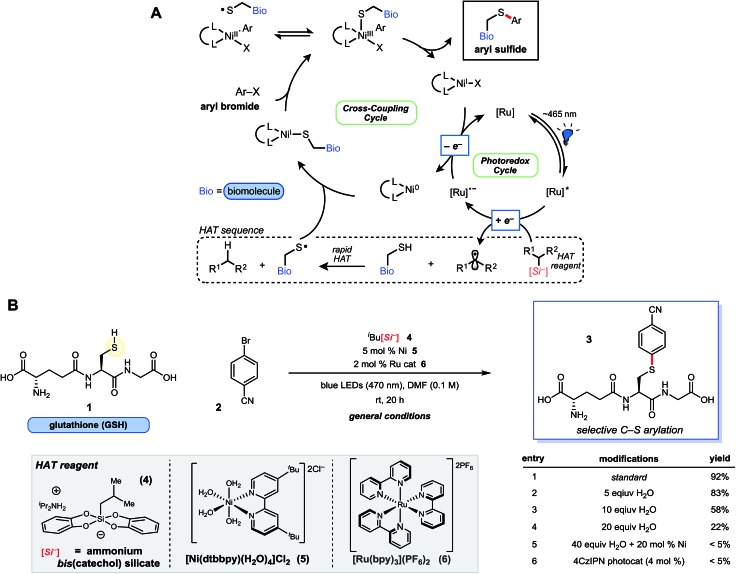
Proposed Ni/photoredox catalytic cycle and thioarylation reaction scheme. (A) Catalytic cycle is initiated by photon absorption, generating excited state Ru photocatalyst, followed by oxidation of the HAT reagent *via* SET. Rapid H-atom abstraction from the sulfhydryl group generates a thiyl radical, which adds to Ni(0). This is followed by Ni(i) oxidative addition^23^ with the requisite aryl bromide. Reductive elimination from Ni(iii) affords the desired thioarylated biomolecule, and the dual catalytic cycles are closed by a final SET. (B) Ni/photoredox thioarylation reaction with GSH (**1**) and **2** affords the arylated peptide. Select experiments are outlined that deviate from the general conditions. Additional experiments and the structure of 4CzlPN can be found in the ESI.†

To this end, a synergistic pairing of photoredox-generated thiyl radicals with Ni cross-coupling catalysis was envisioned to meet these criteria and avoid many common challenges associated with reactive thiolate chemistry (*e.g.*, catalyst poisoning), considering the harder nature of nickel and rapid reductive elimination from Ni(iii) intermediates at room temperature under SET photoredox conditions (Fig. 1A). Herein, a single-electron approach is demonstrated to be distinct and complementary to two-electron thioarylation cross-couplings, not only in mechanism, but in the choice of chemical handles and reagents utilized, which permits orthogonal reactivity with a broad range of aryl halides. Additionally, a suite of adaptable reaction conditions is presented that allow a scalable protocol for various quantities of peptides [from micrograms to grams of substrate/peptide] for the first time by strategically modulating reaction concentrations and rates while keeping catalyst loading low. The conjugation of abundant aryl halides with peptides *via* Ni/photoredox cross-coupling chemistry has permitted rapid and unique diversification of biomolecules previously considered inaccessible, potentially leading to new catalysts, biological probes, and therapeutic lead compounds at nearly any stage in a synthesis.

## Results and discussion

Ammonium bis(catechol)alkylsilicates were recently found to be effective hydrogen atom transfer (HAT) reagents for organic thiols, revealing thiyl radicals (RS˙) primed for Csp^2^–S coupling under the Ni/photoredox manifold.^19^ These silicon-based reagents are increasingly useful in SET chemistry and Csp^3^–Csp^2^ cross-couplings because of their relatively low oxidation potentials (*E*_0_ = +0.75 V *vs.* SCE for 1° alkylsilicates, on average) and straightforward preparation and handling.^20^,^21^ However, it was unclear if the inherent stability and reactivity of these ionic species could be preserved when facilitating the HAT process with alkyl sulfhydryl groups (RS–H) of cysteine residues (BDE = 86 kcal mol^–1^ for cysteine) buried among reactive functional groups. Of particular concern were nucleophilic amines, salts, or known ligand-like substructures of biomolecules, which could disrupt reagent (or catalyst) structure and alter the oxidation potential of ammonium alkylsilicates necessary for an effective HAT event. Nevertheless, efforts were undertaken to take advantage of a rapid HAT and subsequent cross-coupling of biomolecular sulfhydryl moieties (*k*_298K_ = 2 × 10^7^ M^–1^ s^–1^, for HAT with primary alkyl radical donor)^22^ initiated by the production of alkyl radicals generated *via* selective photoredox catalysis.

The feasibility of the thioarylation reaction was initially examined with the tripeptide l-glutathione (γ-Glu-Cys-Gly; GSH, **1**) – an endogenous antioxidant found in nearly all living cells – along with aryl bromide **2** (Fig. 1B). From the outset, keeping reagent stoichiometry proportional was a priority, in hopes of generalizing the findings to small molecule/peptide synthesis (protected or unprotected). Consumption of bromide **2** was satisfactory at 0.1 M in dry DMF, relatively concentrated in comparison to traditional peptide chemistry, but providing conditions that lend themselves to straightforward isolation and purification of unprotected small molecules. Just 5 mol% of a nickel(ii) dtbbpy (dtbbpy = 4,4′-di-*tert*-butyl-2,2′-bipyridine) precatalyst (**5**) and 2 mol% of commercially available [Ru(bpy)_3_(PF_6_)_2_] photocatalyst was found to be effective in the presence of unprotected tripeptide, ultimately affording the desired conjugated adduct **3** in 92% yield under blue light-emitting diode (LED) irradiation at neutral pH (Fig. 1B). Metal-free, organic photosensitizers (*e.g.*, 4CzIPN, Fig. 1B, entry 6), explored in hopes of simplifying purification, were not effective photocatalysts for this transformation. Oxygen or nitrogen cross-coupled adducts were not detected by ultra-performance liquid chromatography coupled with mass spectrometry (UPLC-MS), suggesting selective thiyl radical generation and coupling as envisioned, although trace disulfide formation could be observed (*vide infra*). All reaction components are fully solvated in DMF, apart from peptide **1**, which is visibly insoluble; however, this did not appear to affect the reaction outcome. Up to 10 equiv. of water (2% by volume) in DMF is tolerated (Fig. 1B, entry 3), whereas larger ratios of H_2_O led to poorer reaction conversion (Fig. 1B, entry 4; see ESI†). In direct contrast to palladium reagent-mediated thioarylations of polypeptides, this base-free, Ni/photoredox-mediated arylation is effective with just 5 mol% Ni(ii) catalyst loading without any evidence of thiolate formation or related byproducts, and discrete reaction quenching is not needed. Importantly, all reagents employed are bench stable solids, enabling a “dump and stir” reaction protocol without the need for preformation of the active catalyst.

### Examination of aryl halides

**Chart 1:**
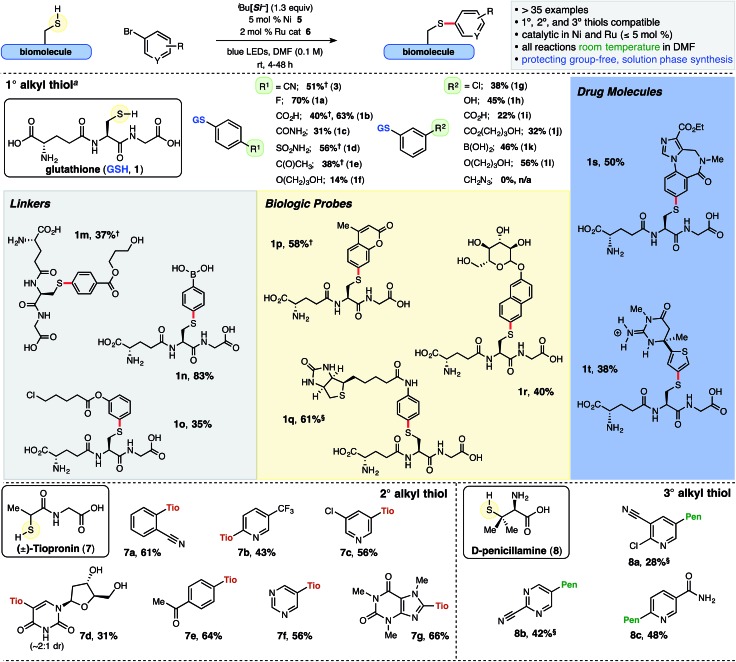
Ni/Photoredox thioarylation reaction and scope of various thiol and arene small molecules. ^i^Bu[Si^–^] = diisopropylammonium bis(catechol)isobutylsilicate. DMF = *N*,*N*-dimethylformamide. Reactions conducted with 0.1 mmol thiol and ArBr, unless otherwise noted; isolated yields are reported (TFA salt omitted for clarity, see ESI† for additional details). ^*a*^ 0.12 mmol GSH employed. ^§^2 equiv. ArBr was employed. ^†^The adduct was filtered following precipitation from the aqueous solution.

In addition to showcasing broad functional group/substrate tolerance, a secondary focus was to incorporate bio-relevant handles for additional chemical and/or bioconjugation strategies. Two distinct thiols, in addition to GSH, were surveyed with various aryl bromides to probe the scope of this transformation – chiral, racemic secondary thiol, tiopronin, a marketed pharmaceutical for the treatment of urologic cystinuria, and a trifunctional, tertiary alkyl thiol, d-penicillamine,^24^ which is used in treatments for Wilson's disease and as a precursor to β-lactam antibiotics and other pharmaceuticals (Chart 1). The inclusion of unnatural or modified amino acids is a validated technique to improve peptide or peptide–drug conjugate properties such as potency or bioavailability, as well as to decelerate metabolism.^25^

Isolated yields reported herein (Chart 1) were enabled by high throughput experimentation and purification *via* mass/UV-directed reverse phase preparatory liquid chromatography (prep-LC) and optimized for purity. Thus, yields do not necessarily reflect accurate conversion to product (see ESI† for details). Additionally, over the course of these studies, it proved prudent to examine workup and purification strategies carefully, as isolation of polar small molecules and peptides can be challenging, particularly on larger scales (>5 μmol). Undesired organic byproducts from the crude thioarylation reaction mixture (catechol, unreacted aryl bromide, and importantly, DMF) could be removed into the organic media *via* extraction from water using CH_2_Cl_2_ when GSH (**1**) or d-penicillamine (**8**) were employed as starting materials. Moreover, select polar or amphoteric aryl sulfide adducts were agreeably found to precipitate from the aqueous solution following extraction, and the solids could be washed and vacuum filtered (see ESI† for details).

Cys-containing glutathione **1** was examined with an array of (hetero)aryl bromides under general conditions. Various unmasked functional handles were tolerated in both the *para* and *meta* positions of bromoarenes, including acids (**1b**, **1i**), benzamide (**1c**), sulfonamide (**1d**), ketone (**1e**), and phenol (**1h**), and the resulting aryl sulfide adducts were isolated in moderate to good yields (1° alkyl thiols, Chart 1). Electron-rich arenes (**1f**) were employed as well, albeit providing lower isolated yields. The aryl bromide bearing an azide was not compatible with this SET chemistry, and activated olefins^26^ yielded a mixture of compounds.

Aromatics bearing tethered primary alcohols (**1f**, **1j**, **1l** and **1m**, Chart 1), which resemble aryl PEG linkers, as well as a primary chloride (**1o**) were selectively linked with GSH (56%, 37%, and 35% yield, respectively). Notably, displacement of the primary chloride was not observed under the mild reaction conditions. Remarkably, free boronic acids were incorporated and isolated in excellent yields (**1k**, **1n**; 46% and 83%, respectively) following a simple acid workup of the crude pinacol boronate (BPin) ester product. Boronic acids have shown unique function as covalent binders with *cis*-diols and cellular surface glycosides.^27^ A pendant biotin derivative (**1q**), coumarin (**1p**), and unprotected glycoside (**1r**) were installed with excellent selectivity at sulfur in good yields (61%, 58%, and 40% yield, respectively). The conjugated coumarin adduct **1p** with an electron-donating sulfur atom in the 7-position is noteworthy, possessing an excitation maximum at 332 nm and emission maximum at 422 nm with a larger Stokes shift than the more common 7-methoxycoumarin.^28^ Additionally, structurally complex, drug-like small molecules such as benzodiazepine **1s**, with potential applications in drug delivery,^29^ and the basic guanidine-containing thiophene **1t** were generated in synthetically useful yields (50% and 38%, respectively).

Thioarylation of the secondary thiol, tiopronin (**7**), proved quite broad when examined with various (hetero)aryl bromides. Hindered *ortho* substituents, as well as the unprotected, deoxyuridine-derived bromide (**7d**, 31%) and drug-like heteroaromatics (**7c** and **7g**, 56% and 66%, respectively) were incorporated. The tertiary thiol, d-penicillamine (**8**) progressed sluggishly in comparison to tiopronin and GSH, likely attributable to increased steric demand around sulfur and the more reversible nature of the thiyl radical addition to the Ni metal center. In general, thiol homodimerization was more evident (5–15%) with increasing alkyl branching. Nonetheless, these hindered aryl sulfides were isolated in useful yields from tertiary thiol **8**, including unprotected nicotinamide derivative **8c** (48% yield, Chart 1), in contrast to the lack of similar reported compounds in Ni/photoredox-catalyzed heteroarylation (O–C^30^ or N–C^31^) reactions.

### Microscale examination of diverse halides

**Fig. 2:**
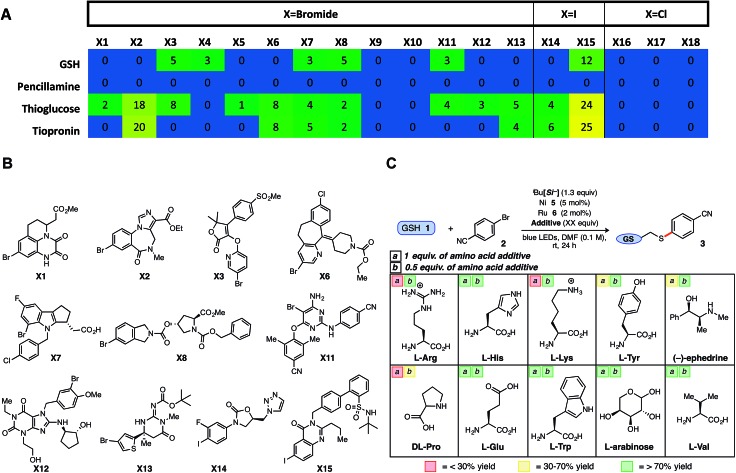
Rapid exploration of Merck aryl halide informer plate *via* HTE and tolerability studies of biological additives under optimized conditions. (A) Merck halides run with 4 diverse thiols; reported numbers in cells present product area%/internal std area% (normalized). (B) Structures of successful Merck halides (**X1–X15**) as explored in A. (C) Biological additives (1 equiv., box a; 0.5 equiv., box b) were screened under optimized conditions; yield determined *vs.* internal standard (average of 2 runs).

In a final, comprehensive demonstration of aryl halide scope, the Ni/photoredox thioarylation protocol was examined *via* high-throughput experimentation (HTE) to evaluate 18 complex halides from a cross-coupling reaction informer set provided by Merck Research Laboratories. Standardized reaction conditions [1 : 2 thiol/halide stoichiometry, 5 mol% Ni (**5**) and 2 mol% Ru (**6**)] were employed across a microscale informer plate, examining four distinct thiols, including a secondary thioglucose derivative, over 24 h on a bed of blue LEDs (Fig. 2A, B and Chart SI-4†). Gratifyingly, the majority of aryl halides showed reactivity ‘hits’ (aryl chlorides inactive) with the employed thiols, demonstrating the reaction's tolerance to dynamic, drug-like aryl bromides, and in particular, aryl iodides (**X14**, **X15**; Fig. 2B).

However, in comparison to penicillamine and in contrast with previous findings, GSH provided uncharacteristically low success rates, likely because of solubility/stirring issues. This informer plate study reveals the potential of the developed thiol-conjugation platform to be a broad-spectrum Csp^2^–S cross-coupling reaction, providing a protocol that leads to reasonable conversions over a variety of complex thiol and aryl bromide partners.

Although an array of functionalized, brominated arenes were deemed compatible, studies were next conducted to gauge the tolerance of this SET thioarylation in the presence of challenging amino acid additives as a basis for applications in more complex peptide chemistry. Encouraged by the GSH results, efforts were focused on more challenging protic, basic, and polyfunctionalized amino acids and other biomolecules (Fig. 2C). The majority of biological small molecule additives examined did not adversely affect the model GSH reaction, with average yields close to that of the control reaction (>70% yield) when 0.5 equiv. of the additive was employed under otherwise normal conditions with aryl bromide **2**. It is worth noting a single prior report using SET photoredox chemistry (macrocyclization, not cross-coupling) on complex biological substrates, required full protection of nearly all amino acid residues.^32^ Acids (*e.g.*, Glu) and aromatic amines (*e.g.*, Trp) were tolerated under Ni/photoredox coupling conditions, as well as the polyol l-arabinose at a full equivalent. The redox- or photoactive aromatic amino acid tyrosine (Tyr) evoked lower conversion to product, and protonated amines (*e.g.*, Arg, Lys) inhibited the reaction when a full equivalent was employed as the additive (<30% yield),^33^ although reactivity could be restored (>70% yield) when 0.5 equivalents of the additive were employed. Nonetheless, more than half of the notoriously problematic, unprotected amino acid additives provided aryl sulfide product in >50% yield under the developed Ni/photoredox cross-coupling.

### Applications in complex biomolecule synthesis

**Fig. 3:**
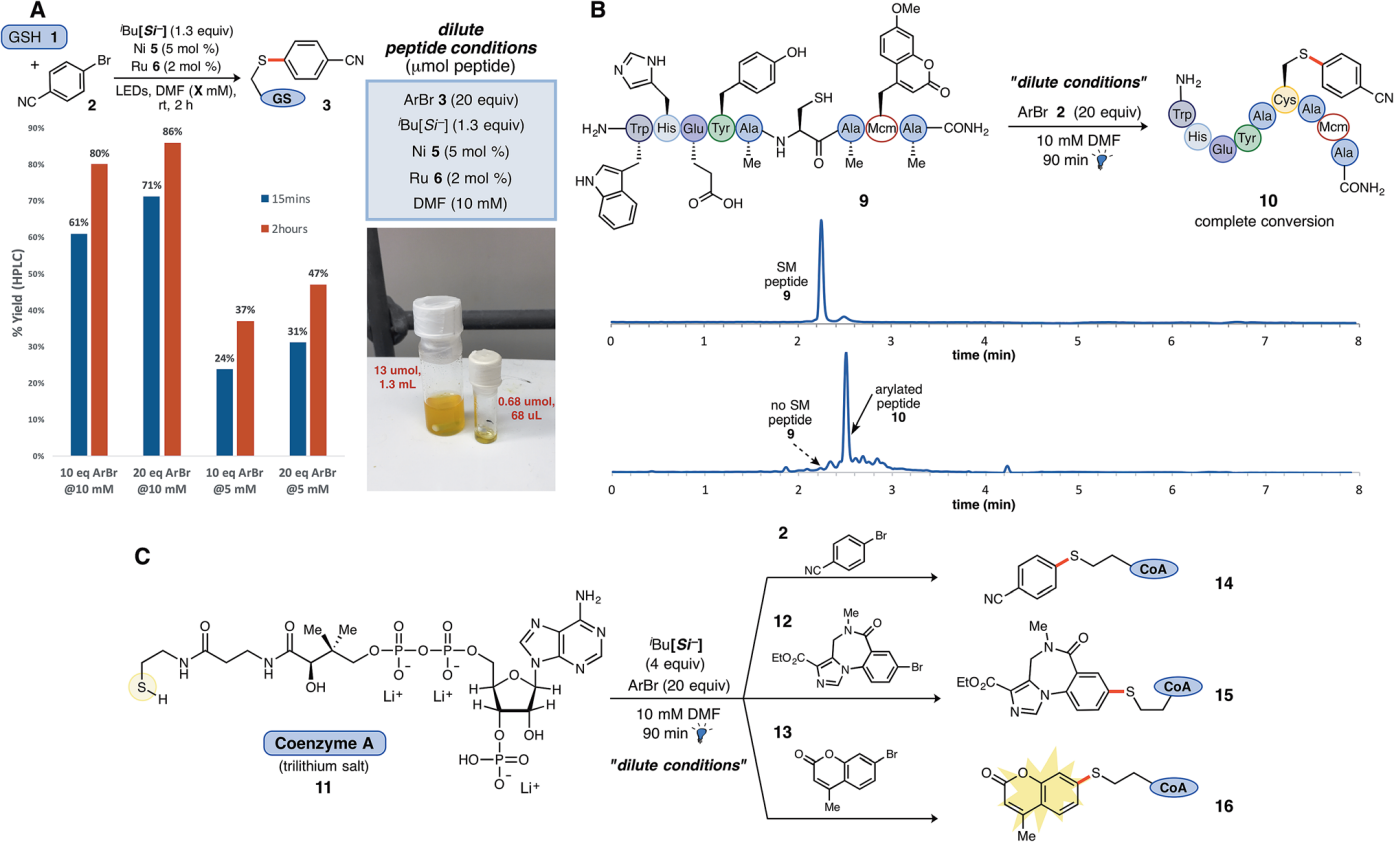
Application of thioarylation reaction conditions to native and diverse biological substrates. (A) Optimized “dilute conditions” with GSH at 10 mM for enabling small-scale peptide thioarylations. Employing 20 equiv. of aryl bromide, **2** was found to increase relative reaction rate, as compared to 5 mM reactions *vs.* time. (B) Peptide **9** was subjected to optimized conditions at 10 mM for 90 min under blue LED irradiation. HPLC chromatogram depicts reaction progression and formation of product (see ESI† for reaction details). (C) Thioarylation of coenzyme A (**11**, 3Li salt) with three diverse aryl bromides (4 equiv. silicate **4** employed) afforded the conjugated products under the developed conditions in all cases.

An adaptable thioarylation reaction employing both small and larger quantities of complex peptides with minimal alterations to reaction parameters is highly desirable. Hence, experiments were designed to determine if established protocols would translate to reactions carried out on micromoles (μg μmol^–1^) of peptide, which are necessarily conducted under more dilute conditions than those previously explored. Cognizant of long-term substrate stability issues, shorter reaction times (<4 h) were sought while maintaining high chemoselectivity. More dilute conditions were scrutinized using GSH (**1**) as the model peptide. Increasing the loading of aryl bromide alone was determined to recover the diminished rate of the reaction at more dilute concentrations (10 mM), furnishing aryl sulfide **3** in 86% yield after 2 hours using the same source of LED irradiation (71% yield after just 15 minutes under the “dilute conditions”; Fig. 3A).^34^ No further adjustments to the nickel or photocatalyst stoichiometry were needed to effect the desired reaction and outcome as described above. These studies generally support aryl bromide activation by Ni oxidative addition as the likely rate determining step (see ESI† for complete studies). Lower peptide concentrations (<5 mM) were not investigated for these studies. Of note, a stock solution containing all required reagents, except the peptide substrate, was allowed to mature for 2 weeks (refrigerated under Ar, no light) and yielded identical results as a freshly prepared reaction solution. Such simple, temporal adjustments through modulation of aryl bromide loading at various concentrations establish a foundation for more diverse or specialized applications.

Complex, globally unprotected Cys-containing peptides were next prepared to validate these dilute reaction parameters. Based on the previous studies with biochemical additives, more dilute reaction conditions were postulated to offset the deleterious effects of the challenging amino acids (*e.g.*, Tyr) on the reaction outcome, as employing only 50 mol% of the amino acid additive largely restored favorable reactivity (see Fig. 2C). Peptide **9**, containing an internal Cys residue within an array of amino acids, was subjected to thioarylation reaction conditions at a 10 mM concentration (Fig. 3B). Full consumption of peptide **9** within 2 hours was confirmed by LCMS analysis, affording the thioarylated peptide **10**. As before, transition metal catalyst loading of just 5 mol% Ni (**5**) and 2 mol% Ru photocatalyst was needed for complete thioarylation, presenting a sharp improvement in catalyst loading as compared to reported thioarylation protocols on polypeptidic systems.^35^ Neighboring amino acid residues were left unmodified as confirmed by matrix-assisted laser desorption ionization (MALDI) tandem MS/MS (see ESI† for complete details).

The notoriously sensitive thiol cofactor, coenzyme A (CoA, **11**), was appropriated as a model substrate under the dilute thioarylation reaction protocol as an additional illustrative example. Chemical entities including peptides, fluorophores, and carbohydrates have been conjugated to CoA^36^*via* thioethers, thioesters, or disulfide linkages to study phosphopantetheinyl transferases (PPTase)-catalyzed protein labeling,^37^,^38^ but to our knowledge, arylative cross-couplings at sulfur have not been carried out on this particular thiol. Initial trial reactions with bromide **2** provided the anticipated aryl sulfide adduct, but in only modest conversion after 2 h. In this case, increasing the amount of HAT reagent (4 equiv.), while keeping all other reaction parameters constant led to rapid and selective thioarylation in 90 min. Given this new finding, complete consumption of CoA **11** when reacted with various aryl bromides was observed in all cases, delivering aryl sulfides **14**, **15**, and the incorporated mercaptocoumarin tag **16** (Fig. 3C). Together, these examples provide encouraging proof-of-concept for the rapid and direct conjugation of complex biological thiols with various arenes using a Ni/photoredox cross-coupling protocol, and may serve as a template for future radical thiolarylative modifications.

**Fig. 4:**
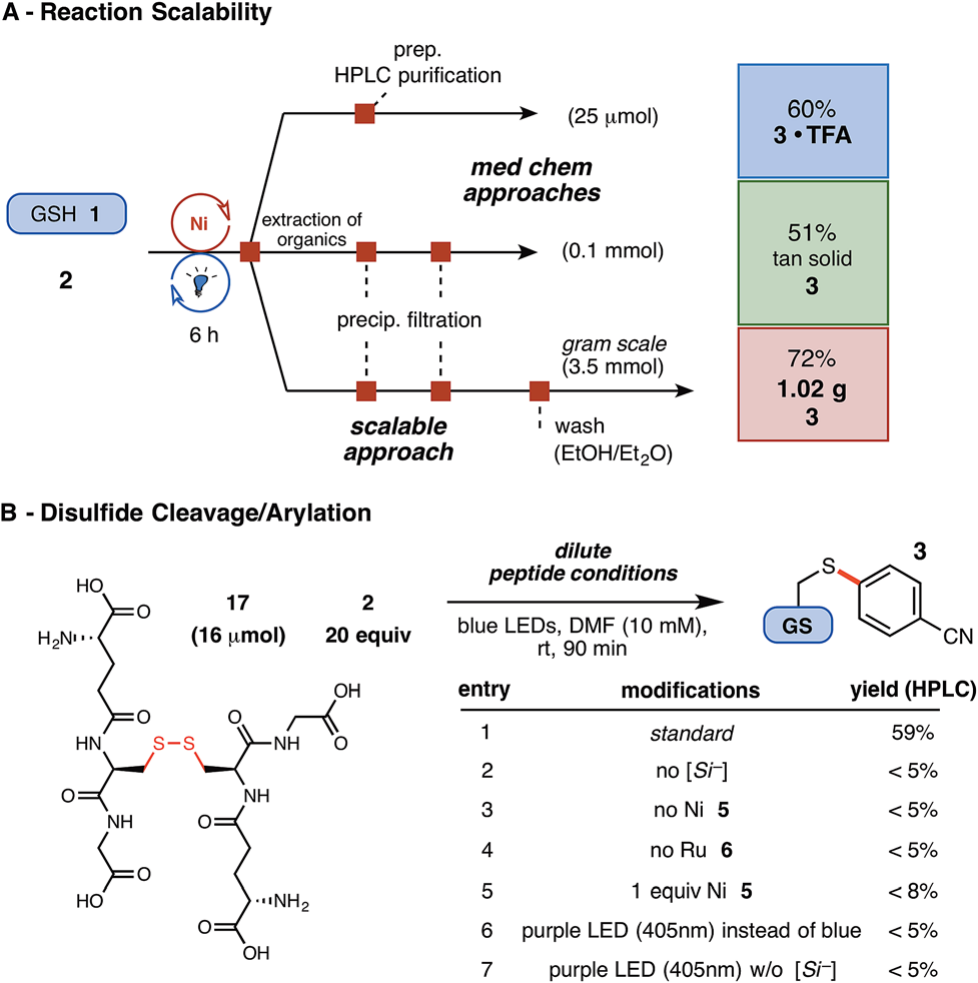
(A) Demonstration of reaction scalability for the Ni/photoredox thioarylation of peptides (see ESI† for details); isolated yields reported. (B) Dilute conditions employed: 5 mol% Ni **5**, 2 mol% Ru **6**, and 1.5 equiv. silicate **4**. HPLC yields reported *via* product/internal standard ratios.

Finally, having demonstrated exceptional functional group and substrate tolerance for the reaction, we examined their aptitude in gram scale setups – a feat unprecedented in peptide literature and potentially useful for early- or late-stage material preparation – as well as the proclivity of disulfide linkages to undergo productive arylation under these conditions. Enabled by an inexpensive Ni catalyst and by avoiding superfluous concentrations, the optimized thioarylation parameters with GSH (in 0.1 M DMF) translated agreeably on larger scale (3.5 mmol, 1.07 g of GSH), employing the same source of LED irradiation, to afford just over a gram of arylated GSH (**1**) following extraction and vacuum filtration (Fig. 4A). As presented, this economical reaction can tolerate various quantities of unprotected substrate and requires little additional reaction adjustments for the amount or type of starting thiol employed.

Using this radical reaction pathway, trace disulfide formation was observed in the early stages of optimization, which provoked the exploration of employing preformed disulfide substrates in this chemistry. Disulfide linkages play a critical role in peptide tertiary structure integrity, yet provide potentially useful, natural linchpins for chemical manipulation.^39^ In this study, an unprecedented two-step, disulfide cleavage/arylation of oxidized GSH dimer (**17**) was uncovered (Fig. 4B), without requiring additional reducing agents. The resultant arylated thiol **3** was isolated in 44% yield following extraction and purification, and other arylated or modified GSH adducts were not detected by UPLC-MS analysis. Control studies suggest that all components of the reaction are required for success, and higher energy LEDs (near-UV), which might promote disulfide homolysis, proved unproductive (Fig. 4B, entries 6–7). The mechanistic nuances of this multi-component reaction are to date unclear, but the results nonetheless provide insight into the low amounts of disulfide byproduct observed in the principle reaction (5–15%, depending on thiol used) when alkyl sulfhydryl substrates are employed.^40^,^41^ Photoredox catalysis, in this regard, may offer a selective and well-situated solution to disulfide cleavage and modification in peptidic or similarly complex systems.

## Summary and conclusions

In summary, we present the first application of a Ni/photoredox, dual-catalyzed cross-coupling on unprotected, biologically-derived thiols with wide scope in both thiol and aryl halide partners. Although water as a cosolvent was not generally tolerated, otherwise robust reaction parameters under this radical, HAT-initiated protocol allow simple temporal adjustments, depending on the type (primary through tertiary alkyl thiols) or quantity (μg to grams) of thiol employed, and cysteine-containing polypeptides were selectively conjugated to various arenes in under 90 min in DMF (10 mM). Straightforward reaction set-up also permitted microscale high throughput experimentation (HTE) to screen optimal conditions and substrates in an effort to broaden the reach of this transformation in unprotected systems. Rapid screening of aryl halides and reaction scalability were largely enabled by an inexpensive, versatile, and bench-stable Ni precatalyst (5 mol% employed). The designed Ni/photoredox thioarylation reaction does not require transition-metal reagents, large excess of aryl halide (>20 equiv.), nor elaborate ligand design, and may well serve research communities interested in quickly accessing native, protected and/or unprotected, thioarylated small biomolecules, thus serving as a practical complement to Pd-catalyzed processes that have proven effective for protein bioconjugation.

## Conflicts of interest

There are no conflicts to declare.

## Supplementary Material

Supplementary information
