# Supplementary material for: Scalable thioarylation of unprotected peptides and biomolecules under Ni/photoredox catalysis
Source: Chem Sci. 2017 Nov 13;9(2):336–44. doi: 10.1039/c7sc04292b (PMC5868321; doi:10.1039/c7sc04292b)
Supplement: Supplementary file 1 [file SC-009-C7SC04292B-s001.pdf]

Electronic Supplementary Material (ESI) for Chemical Science.
This journal is © The Royal Society of Chemistry 2017

Supporting Information

Scalable Thioarylation of Unprotected Peptides and Biomolecules under Ni/Photoredox Catalysis

Brandon A. Vara, Xingpin Li, Simon Berritt, Christopher R. Walters, E. James Petersson, and Gary A. Molander*

Roy and Diana Vagelos Laboratories, Department of Chemistry, University of Pennsylvania, 231 South 34th Street, Philadelphia, Pennsylvania 19104-6323, United States

*To whom correspondence should be addressed. E-mail: gmolandr@sas.upenn.edu

Table of Contents

General Considerations 2
- **Photochemistry** 2
- **General peptide purification parameters:** 3
- **High-Throughput Experimentation** 3
- **General High-Throughput Experimentation Protocol** 3

Select Reaction Optimization Studies 5

General Procedure for Thioarylation of Peptides (0.1 mmol scale) 8

Gram-scale Thioarylation Reaction with Glutathione 9

Merck HTE informer plate data 11

General Procedure for Thioarylation of Peptides under "Dilute Conditions" 13

Thioarylation of Peptide 9 under "Dilute Conditions" (0.68 μmol scale) 14
- **Peptide HTE Screen** 15

Compound Characterization Data 19

Thioarylated Peptide 10 Data 31
- **HPLC and MALDI Analysis of Peptide Cys Arylation Reactions** 31

Enzyme CoA Data 33
- **Reaction preparation and execution for Enzyme CoA Reactions (14-16):** 33

Cleavage/Arylation of GSH Disulfide (17) 38
- **Reaction preparation and execution for Enzyme CoA Reactions:** 38

Supporting Data 39
- **Select Supplemental HPLC traces for arylated GSH adducts** 39
- **Synthesis of Cys Containing Polypeptide 9** 47

NMR Spectral Data 49

General Considerations

All reactions were carried out under an inert atmosphere of nitrogen or argon in oven-dried glassware, unless otherwise noted. Conventional solvents (THF, Et_2O, CH_2Cl_2, toluene, xylenes) were dried using a solvent system. DMF (99.8%, extra dry) was used as received, and all other reagents were purchased commercially and used as received, unless otherwise noted. $RuCl_3{\cdot}xH_2O$ and $[NiCl_2(dme)]$ were purchased from commercial sources. $[Ni(dtbbpy)(H_2O)_4]Cl_2$ precatalyst, [1] $[Ru(bpy)_3(PF_6)_2]$ photocatalyst, [2] and 4CzIPN [3] were prepared following recent literature reports. Diisopropylammonium bis(catechol)isobutyl silicate was prepared using a published procedure.[4] As previously reported, various similar silicate reagents can be employed in this chemistry.[5] *iso*-Butyl silicate was chosen because of the volatility of the byproduct, *iso*-butane. Aryl halides were purchased from commercial suppliers and used without further purification. Reaction monitoring was carried out by reverse phase ultra-performance liquid chromatography coupled with mass spectrometry (UPLC-MS, 3 minute runs) and/or reverse phase high performance liquid chromatography (HPLC, 8 minute runs). Accurate mass measurement data were acquired on a Waters LCT Premier XE by use of electrospray ionization (unless otherwise stated) with an internal lock mass reference of leucine enkephalin. Waters instruments are calibrated, and report by use of neutral atom masses. NMR spectra (^1H, ^{13}C {^1H}, ^{19}F {^{13}C}) were performed at 298 K. ^1H (500.4 MHz) and ^{13}C {^1H} (125.8 MHz) NMR chemical shifts are reported relative to internal TMS (δ = 0.00 ppm; $CHCl_3$: 7.26 ppm for ^1H nuclei and 77.00 for ^{13}C nuclei); (δ = 0.00 ppm; D_2O: 4.80 ppm for ^1H nuclei); (δ = 0.00 ppm; DMSO-d_6: 2.50 ppm for ^1H nuclei and 40.00 for ^{13}C nuclei); (δ = 0.00 ppm; CD_3OD: 3.31 ppm for ^1H nuclei and 49.0 for ^{13}C nuclei). ^{11}B (128.4 MHz) and ^{19}F {1H} NMR (470.8 MHz) chemical shifts were referenced to external $BF_3{\bullet}Et_2O$ (0.0 ppm) and $CFCl_3$ (0.0 ppm), respectively. Data are presented as follows: chemical shift (ppm), multiplicity (s = singlet, d = doublet, t = triplet, q = quartet, sept = septet, m = multiplet, br = broad), coupling constant *J* (Hz) and integration.

Photochemistry:

Photoredox reactions were irradiated with blue LED (~ 470 nm, 32,918 mcd ft^{-1}) strips, placed along the inside of a Pyrex® crystallizing dish (125 X 65 mm), and the temperature (~ 30 °C) was controlled using one external desk fan set up ~ 15-20 cm away from this photoreactor bed. The fan was employed to ensure reactions remained at or near room temperature when using LEDs which warmed during the reaction. A modified test tube rack was designed to allow multiple reaction setups simultaneously. LEDs were configured as outlined in the photochemical reactor design (image right). Purple LEDs (near UV, 400 nm) were also tested and set up in an analogous manner.

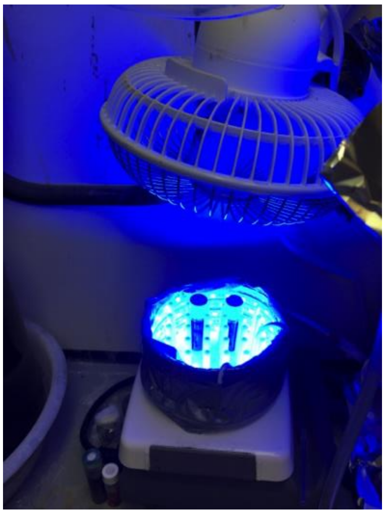

Reactions run in high-throughput fashion were carried out in a clear vial holder situated over a bed of small blue LEDs (**Figure SI-1 b and c**), and a fan was placed ~ 20 cm away from the reaction plate (not shown).

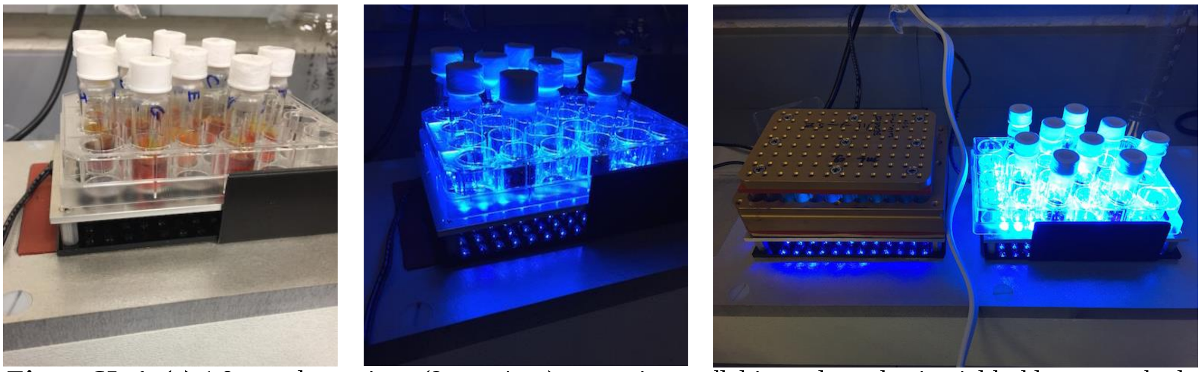

Figure SI- 1. **(a)** 1.0 mmol reactions (9 reactions) set up in parallel in a clear plastic vial holder over a bed of blue LEDs (turned off) and magnetic stir pad. **(b)** Same 0.1 mmol reactions irradiated and stirring over a bed of blue LEDs. Fan is off-camera and not pictured. **(c)** Ni/photoredox reaction setup with 0.1 mmol GSH reactions (right) beside a 24-well HTE plate (left) assembled and sealed in the glovebox (see HTE reactions guidelines for setup and procedure).

General peptide purification parameters:
Reverse phase preparative chromatography for the small molecule peptides was performed by mass/UV (254 nm) directed preparatory liquid phase chromatography (Prep-LC), specifically Waters AutoPurification System equipped with Waters 3100 MS. Waters Sunfire, C18, 5 µm 19x100 mm column was used, unless otherwise noted. Reverse phase preparatory chromatography for larger polypeptides was performed on a Varian Prostar HPLC system. Analytical HPLC for analysis of polypeptide purity and product formation was performed on an Agilent 1100 series HPLC system. Polypeptide starting materials were analyzed by low resolution electrospray ionization mass spectrometry (ESI-LRMS) on a Waters Acquity Ultra Performance LC connected to a single quadrupole detector (SQD) mass spectrometer. Polypeptide reactions were analyzed using Matrix Assisted Laser/Desorption Ionization-mass spectrometry (MALDI-MS) on a Bruker Ultraflex III mass spectrometer with a time-of-flight detector.

High-Throughput Experimentation:
High Throughput Experimentation (HTE) was performed at the Penn/Merck Center for High Throughput Experimentation at the University of Pennsylvania. All reagents were used as purchased from commercial suppliers. Solvents were purchased anhydrous and used with no further purification. All reactions were performed inside a glovebox operating with a N_2-atmosphere (oxygen typically < 5 ppm). Reaction experimental design was aided by the use of Accelrys Library Studio. 2.5 µmol scale reactions for the limiting reagent were carried out in HPLC vial glass inserts, 4 x 21 mm, 50 µL, flat bottom) equipped with magnetic tumble stir bars in 96-well reaction plates. Liquid handling was carried out using single and multi-channel pipettors (10, 100, 200, and 1000 µL). On completion of solution dosing the plates were covered by a perfluoroalkoxy alkane (PFA) mat, followed by two silicon rubber mats, and an aluminum cover which was tightly and evenly sealed by 9 screws. Reactions were monitored by UPLC-MS. Column: Acquity UPLC HSS C18 1.7 µm 2.1x50 mm, using 0.1% TFA MeCN and 0.1% TFA H_2O as mobile phases. The instrument was equipped with an SQD detector with electrospray ionization (ESCi) source in positive and negative mode. High throughput data analysis was carried out with Virscidian Analytical Studio™ software.

General High-Throughput Experimentation Protocol
Stock solutions of the relevant catalyst or ligand were prepared in DCE or THF. Using a 200 µL pipettor, the catalyst or ligand solutions were dispensed into 1 mL glass vials arrayed in a

96 well microtitre plate according to the required Ni/L loading per reaction screen. The solvent was removed *in vacuo*. Inorganic bases (if applicable) Cs_2CO_3, K_2CO_3, K_3PO_4, and Na_2CO_3 were added either by slurry addition of the relevant base (25 mg/mL slurry stock solution in THF) or by manual solid addition using a BioDot™ DisPro MAR Series Adjustable Mass Powder Dispenser. The vials were then charged with tumble stir bars. Using a multichannel pipettor, a solution of aryl bromide and thiol (100 µL) in the relevant screen solvent was added to each 1 mL vial. Stock solutions of each additive (1 M/20 µL in THF) was then added using a 200 µL pipettor. The 96 well reaction plate was covered with the PFA film, sealed, removed from the glovebox, and placed on a bed of blue LEDs (**Figure SI-1c**). The vials were stirred at the corresponding reaction temperature for the allotted time. The reactions (if required) were cooled to ambient temperature and quenched with a stock solution of 4,4'-dimethylbiphenyl in MeCN (0.04 M, 500 µL) and mixed thoroughly. Aliquots of the quenched reactions were taken (20 µL), further diluted with 700 µL of MeCN, and subjected to UPLC and/or UPLC-MS analysis.

Select Reaction Optimization Studies

Additional studies examining various reaction conditions as deviated from the standard:

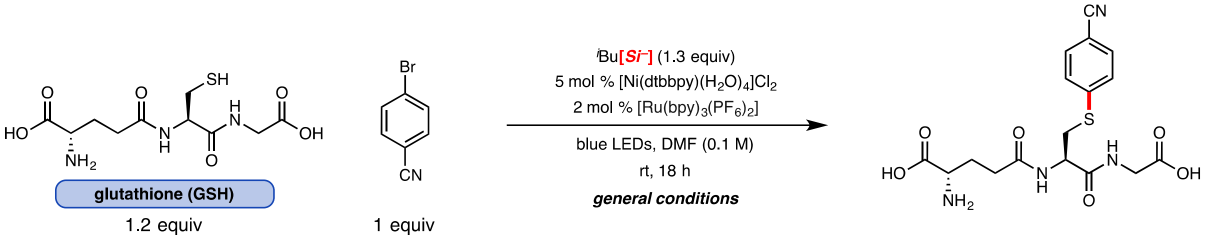

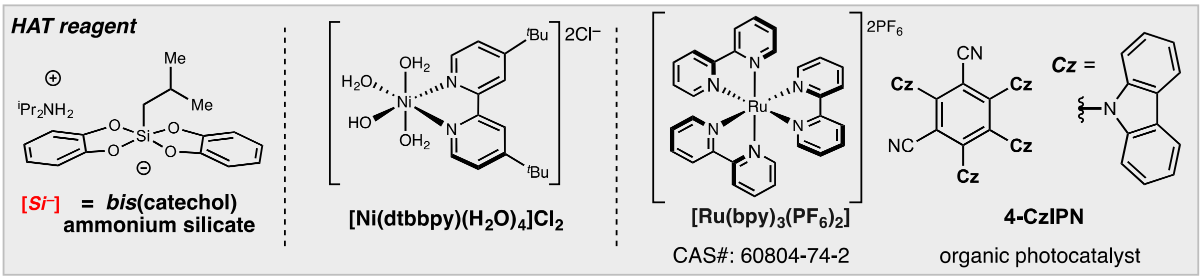

entry	change from standard	% yield (HPLC)
1	n/a	> 90%
2	20 equiv H_2O	22%
3	40 equiv H_2O	< 20%
4	40 equiv H_2O + 10 mol % Ni	Trace
5	40 equiv H_2O + 50 mol % Ni	Trace
6	10 mol % Ni	45%
7	1 mol % Ru	59%
8	4 mol % Ru + 10 mol % Ni	< 30%
9	8 mol % Ru + 20 mol % Ni	< 8%
10	1 mol % Ru + 2.5 mol % Ni	40%
11	1.5 equiv ^{t}Bu[Si$^-$]	> 90%
12	1.0 equiv ^{t}Bu[Si$^-$]	74%
13	4-CzIPN photocatalyst (4 mol%)	Trace

Table SI-1. HPLC yield as determined to an internal standard.

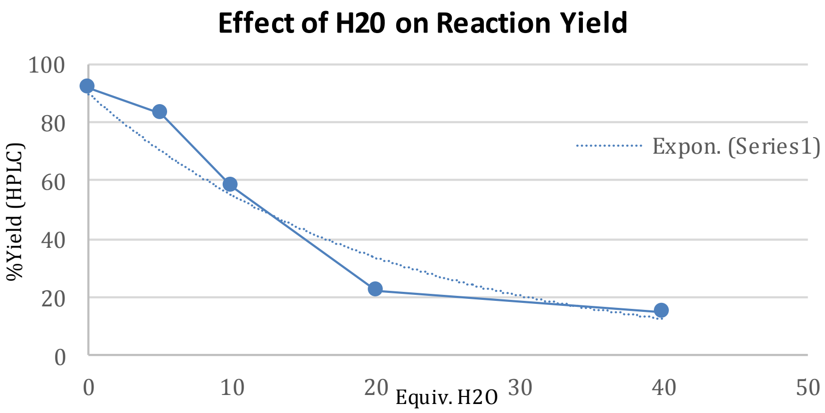

Effect of aryl bromide (4-cyano bromobenzene) loading on the reaction outcome at various time points. In general, with higher aryl bromide loading, the faster the reaction was after 1 h, although decomposition of the arylated GSH to unidentified products was observed after prolonged reaction times (**Chart SI-1**). These reactions were run in triplicate (averaged), and the optimized reaction conditions/yield can be observed using 1 equiv ArBr after 18 h. The trend-lines overlaid to show the effects of increasing ArBr equiv over temporal extremes (1 and 18 h), all else constant.

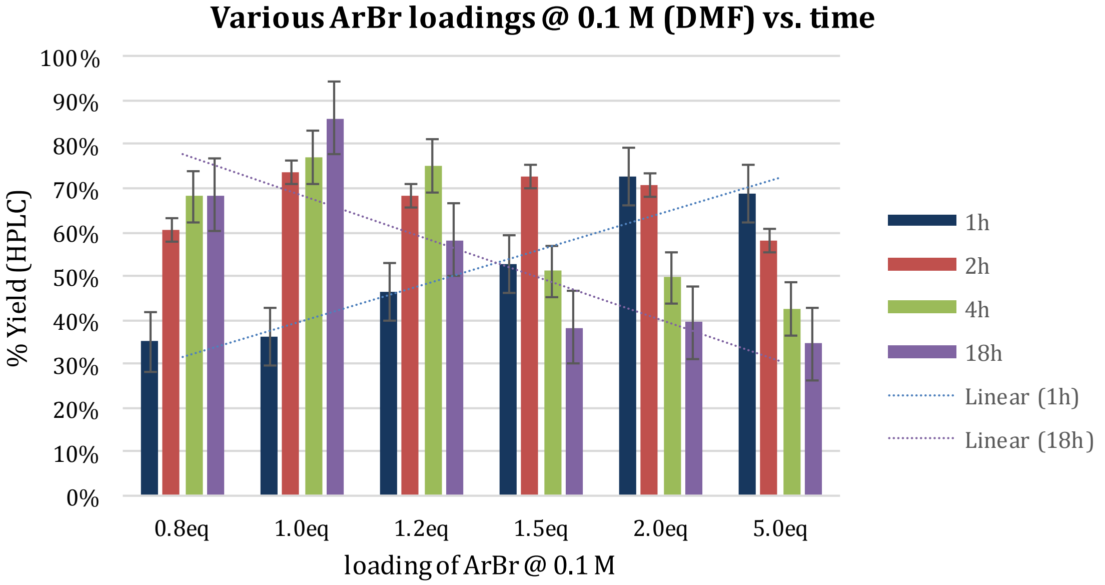

Chart SI- 1. Optimization experiments examining aryl bromide loading with GSH (1.2 equiv, 0.1 M DMF) at various time points under otherwise standard reaction conditions. Yield as compared to product/IS ratios.

More dilute reaction conditions were pursued employing 1 equiv of the ArBr and GSH (1.2 equiv) at various time points, which would be ideal when examining small quantities of peptide thiols (**Chart SI-2**). As depicted, 0.1 M DMF proved optimal with these reagent parameters, and reactivity diminished (nearly linearly) at more dilute concentrations.

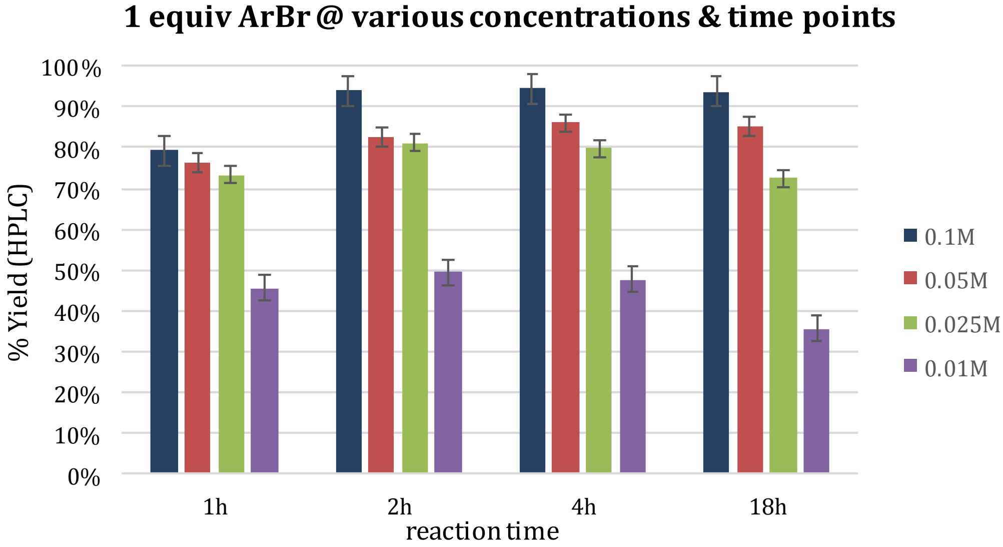

Chart SI- 2. ArBr loading at various reaction concentrations over time under otherwise standard reaction conditions. Yield as compared to product/IS ratios.

Additional optimization reactions to uncover "dilute conditions" while maintaining fast reaction kinetics (< 4 h) was pursued at various loadings of aryl bromide (reactions run together in duplicate, **Chart SI-3**). 20 equiv of ArBr was found to optimal in a side-by-side

comparison of the reactions after 2 h, although 10 equiv may be substituted in certain situations when ArBr is very expensive. As noted earlier, decomposition of the product was seen with higher ArBr loadings after prolonged reaction times (e.g., 20 h).

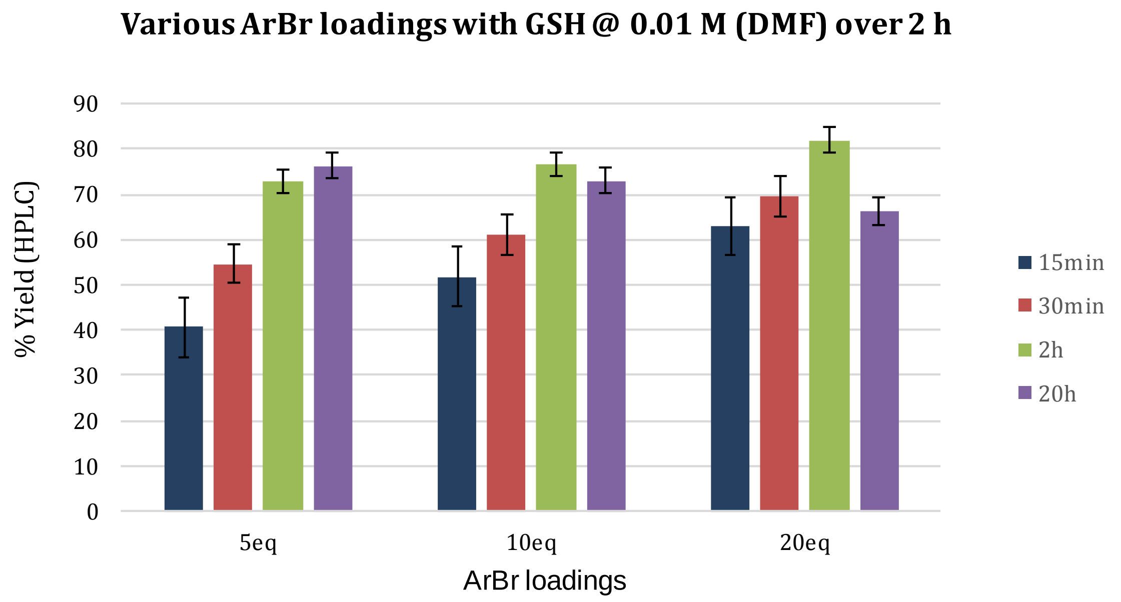

Chart SI- 3. ArBr loadings with GSH at 0.01 M DMF and 1.3 equiv silicate ("dilute conditions") at various time points under otherwise standard reaction conditions

General Procedure for Thioarylation of Peptides (0.1 mmol scale)

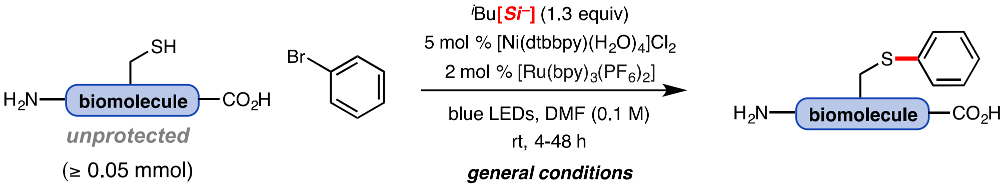

A 4 mL sealable screw cap vial with septum was charged with the unprotected peptide (0.1 mmol, 1.0 equiv) and the aryl bromide (0.1 mmol OR 0.12 mmol, 1.0/1.2 equiv), followed by addition of diisopropylammonium bis(catechol)isobutylsilicate (54 mg, 0.13 mmol, 1.3 equiv), [Ni(dtbbpy)(H_2O)$_4$]Cl_2 (2 mg, 0.05 mmol, 5 mol %), and [Ru(bpy)$_3$(PF_6)$_2$] (1.7 mg, 0.02 mmol, 2 mol %) in succession. The vial was sealed, and three vacuum/Ar cycles were carried out. Next, dry and degassed DMF (1.0 mL) was added. The vial containing all the reagents was further sealed with Parafilm, placed in the photoreator vessel ~5-10 cm away from the blue LEDs (see **Figure SI-1**), and allowed to stirred vigorously for 4-48 h at rt. The temperature of the reaction was maintained at approximately 30 °C via a fan. Reaction progress was monitored by HPLC and/or UPLC/MS. Once complete, the now darker, milky-brown solution can be diluted with purified H_2O (0.3-1.0 mL, or until sufficiently homogeneous) and directly purified by reverese phase preparatory liquid chromatography.*

Extraction protocol for catechol removal: To the now darker, milky-brown solution was added deionized H_2O (1 mL), and CH_2Cl_2 (preferably, 1 mL) or CH_3Cl (1 mL). This mixture (3 mL solution, total) was vigorously shaken in the original reaction vial, the layers were allowed to separate, and the organic layer (containing catechol, dtbbpy, unreacted silicate, and DMF) was removed via syringe or glass pipette. This sequence was repeated 2-4 times. The remaining aqueous layer (containing starting peptide [if applicable], thioarylated peptide, *i*-Pr_2NH, Ru photocatalyst, and trace DMF) was removed and subsequenly purified via reverese phase prep-LC.

Precipitation of desired thioarlyated peptide (only tested with arylated GSH adducts): The extraction process as detailed above was carried out and repeated 2-4 times. In cases where the thioarylated adduct is sparingly soluble in pure water, this pure product can slowly precipitate given suitable conditions. Following extraction, if solid material was present, it was helpful to let the aqueous solution sit on the benchtop and/or gradually cool (0-5 °C) for 2-18 h to encourage maximum precipitation. The thioarylated peptide precipitate was then vacuum filtered through a glass frit or Büchner funnel and gently washed with ice water (no more than 2 mL), then Et_2O. The filtered solid was analytically pure in nearly all cases.

**NOTE:* In many cases, the final thioarylated peptide may not be fully soluble in the resulting DMF/H_2O solution. Filtering this heterogeneous mixture before prep-LC has resutled in decreased yield as the filtered solid contained a fair amount of desired arylated peptide.

Gram-scale Thioarylation Reaction with Glutathione

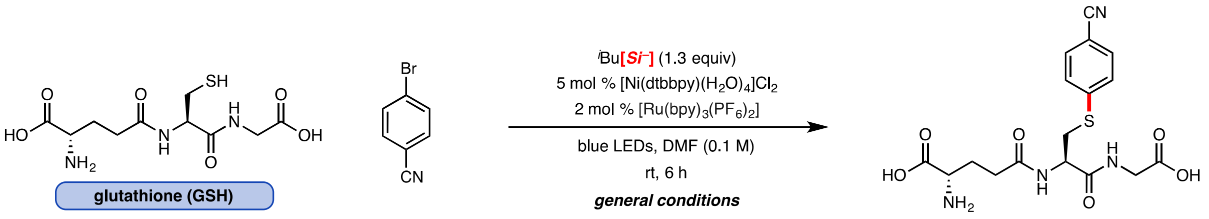

3.5 mmol scale reaction: A 40 mL scintillation vial with air-tight, sealable septum was charged with glutathione (1.07 g, 3.5 mmol, 1.0 equiv, not fully soluble) and the aryl bromide (637 mg, 3.5 mmol, 1.0 equiv), followed by addition of diisopropylammonium bis(catechol)isobutyl silicate (1.41 g, 4.55 mmol, 1.3 equiv), [Ni(dtbbpy)(H_2O)$_4$]Cl_2 (70.0 mg, 0.175 mmol, 5 mol %), and [Ru(bpy)$_3$(PF_6)$_2$] (60 mg, 0.07 mmol, 2 mol %) in succession. The vial was sealed, connected to a dual line manifold with needle, and four vacuum/Ar cycles (3 min each) were carried out. Next, dry and degassed DMF (35 mL) was added under Ar. The vial containing all the reagents was further sealed with Parafilm, placed in the photoreactor vessel ~5-10 cm away from the blue LEDs (see **Figure SI-2b**), allowed to stirred vigorously for 6 h at rt, and periodically checked for reaction completion by HPLC analysis. The temperature of the reaction was maintained at approximately 32 °C via a fan. The now darker, milky-brown solution was transferred to a separatory funnel with deionized H_2O (35 mL), followed by CH_2Cl_2 (35 mL). This mixture was vigourously shaken, the layers were allowed to separate, and the organic layer was removed. This sequence was repeated 4 times. The remaining aqueous layer containing solid precipitate was transffered to a 50 mL Erlenmeyer flask and placed in the refrigerator for 7 h. The solid precipitate was vacuum filtered via Büchner funnel, washed with ice water (10 mL), then Et_2O (30 mL) to afford a light beige solid (1.02 g, 72% yield).

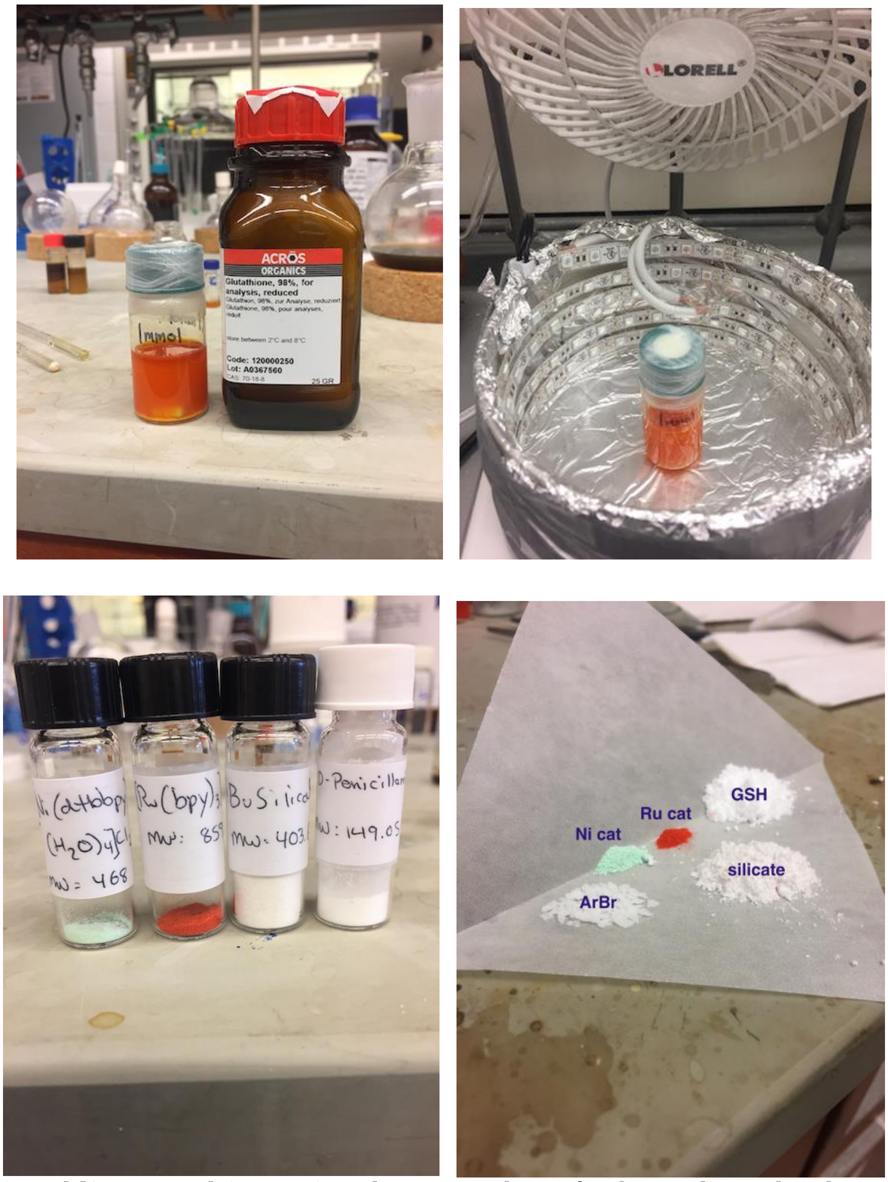

Figure SI- 2. (**a** and **b**) 1.0 mmol (307 mg) scale reaction shown for the Ni/photoredox thioarylation reaction employing glutathione. Similar reaction vial and setup were applied to the gram scale reaction, using the same blue LED photoreactor. (**c** and **d**) All the necessary reagents weighed out on the benchtop in the air, before direct addition to the reaction vial.

Merck HTE informer plate data

Thiol	Conditions	X1	X2	X3	X4	X5	X6	X7	X8	X9	X10	X11	X12	X13	X14	X15	X16	X17	X18
		X=Bromide													X=I		X=Cl		
GSH	0.1M 1.eqv ArX	0	7	0	0	0	0	0	0	0	0	0	0	0	0	0	0	0	0
GSH	0.1M 2 eqv. ArX	0	0	5	3	0	0	3	5	0	0	3	0	0	0	12	0	0	0
GSH	0.05M 2 eqv. ArX	0	0	0	0	0	0	0	0	0	0	0	0	0	0	33	0	0	0
GSH	Standard Conditions	3	26	0	0	0	0	0	0	0	0	0	0	0	0	9	0	0	0
Thioglucose	0.1M 1.eqv ArX	1	5	2	0	1	2	2	0	0	0	0	1	1	5	14	0	0	0
Thioglucose	0.1M 2 eqv. ArX	2	18	8	0	1	8	4	2	0	0	4	3	5	4	24	0	0	0
Thioglucose	0.05M 2 eqv. ArX	6	15	6	4	3	12	8	7	0	3	6	7	0	12	45	0	0	0
Thioglucose	Standard Conditions	0	13	7	0	2	12	5	7	0	0	6	0	0	10	34	0	0	0
Tiopronin	0.1M 1.eqv ArX	0	0	0	0	0	5	0	0	0	0	0	0	1	5	16	0	0	0
Tiopronin	0.1M 2 eqv. ArX	0	20	0	0	0	8	5	2	0	0	0	0	4	6	25	0	0	0
Tiopronin	0.05M 2 eqv. ArX	0	37	0	0	0	16	0	0	0	0	0	0	0	21	43	0	0	0
Tiopronin	Standard Conditions	1	29	0	1	1	14	2	1	0	0	0	1	3	7	35	0	0	0

Chart SI-4. Complete study of 4 distinct thiols against the Merck halide informer plate. Product %area/IS %area ratios [normalized against IS (10 mol% IS) giving relative values] are reported in the box cells and colored coded based on the relative ratios (conversion to product).

Product %area/IS %area ratios are reported [normalized against IS (10 mol %) giving relative values]. GSH provided uncharacteristically poor results in this HTE format, most likely because of poor solubility, stirring, or a combination of both. Parameters and conditions were explored to improve conversion across the board with GSH to little avail. Notably, bromide **X2** was employed GSH under standard reaction conditions (0.1 mmol scale) and the thioarylated adduct was isolated in 50% yield (see manuscript). Thioglucose (tetraacetate used because of commercial availability) and tiopronin were agreeably soluble under the DMF screening conditions, and exhibited exceptional reactivity considering these were more hindered, secondary thiols.

class of distinct thiols examined in HTE against Merck informer plate

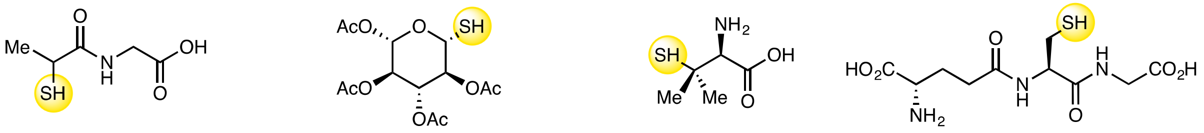

(±)-Tiopronin thioglucose tetraacetate D-penicillamine glutathione

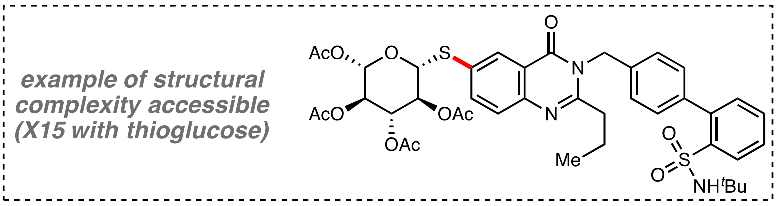

Complete list of Merck informer halides examined:

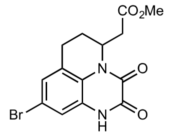

X1

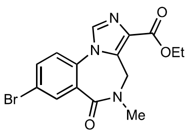

X2

X3

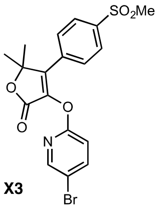

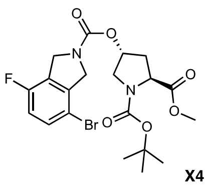

X4

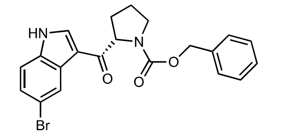

X5

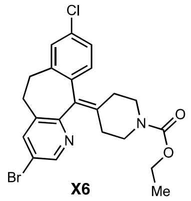

X6

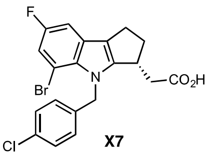

X7

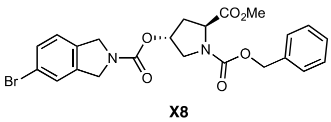

X8

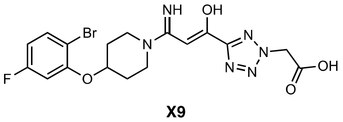

X9

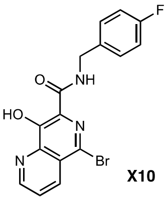

X10

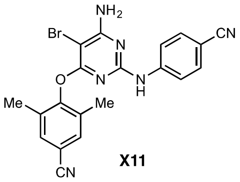

X11

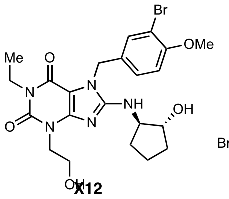

X12

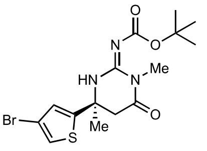

X13

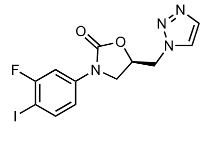

X14

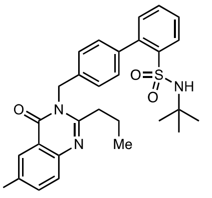

X15

X16

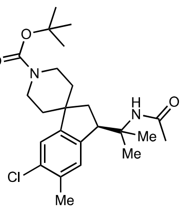

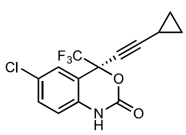

X17

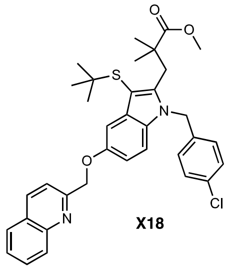

X18

General Procedure for Thioarylation of Peptides under "Dilute Conditions"

For peptides and enzyme CoA reactions

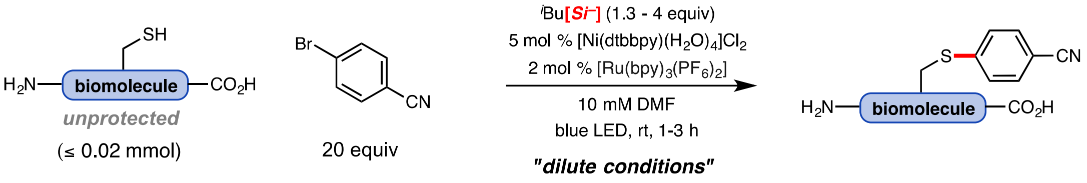

General preparation of stock solution: A sealable screw cap vial with septum was charged with the aryl bromide (230 mg, 1.28 mmol, 20 equiv), diisopropylammonium bis(catechol)isobutyl silicate (34 mg, 85 µmol, 1.3 equiv), [Ni(dtbbpy)(H_2O)$_4$]Cl_2 (1.3 mg, 3.3 µmol, 5 mol %), and [Ru(bpy)$_3$(PF_6)$_2$] (1.1 mg, 1.3 µmol, 2 mol %) in succession. The vial was sealed, and three vacuum/Ar cycles were carried out to remove oxygen. Next, dry and degassed DMF (6.5 mL) was added under Ar. As noted, the stock solution can be stored in the refridgerator, in the dark, under Ar for at least 2 weeks with identical reaction efficacy as compared to a freshly prepared stock solution.

Reaction preparation and execution: In a separate, oven-dried vial was added a magnetic stirbar, the peptide or thiol of choice (13 µmol, 1 equiv), followed by three vacuum/Ar cycles. To the vial containing the peptide and stirbar was added 1.3 mL of the DMF stock solution containing the reagents, under Ar, and the vial was sealed with Parafilm. This reaction vessel was placed in a blue LED photoreactor ~4-8 cm away from the irradiation source, and the reaction was allowed to stir for 1-3 h. The temperature of the reaction was maintained at approximately 29 °C via an overhead fan (see **Figure SI-3c**). Reaction progress was carefully monitored via syringe needle by HPLC and/or UPLC-MS, and once complete, the slightly darker solution was either **a)** diluted with purified H_2O (~ 0.5 mL) and directly purified by reverese phase preparatory liquid chromatography, or **b)** diluted with purified H_2O (1.3 mL) and the aqueous layer extracted with CH_2Cl_2 three times – the reamining aqueous layer was subjected to purification.

NOTE: Reactions with enzyme CoA and the various aryl bromides (20 equiv) were carried out in identical manner to that described above, except 4 equiv of the diisopropylammonium bis(catechol)isobutyl silicate was employed. See line listing reaction details below for additional details.

Thioarylation of Peptide 9 under "Dilute Conditions" (0.68 μmol scale)

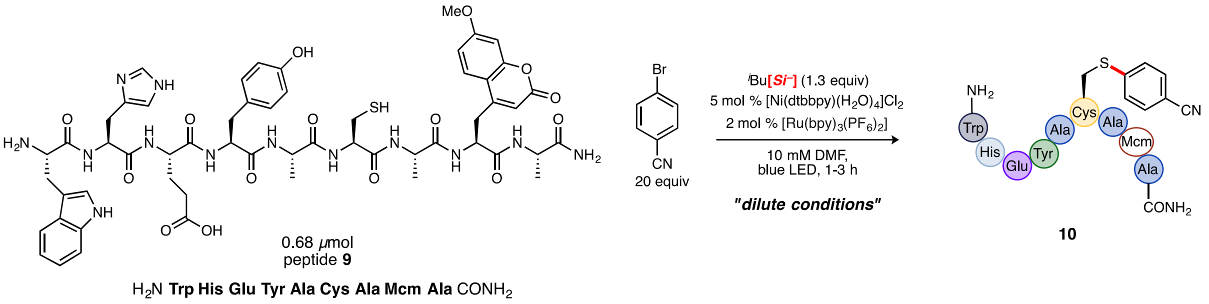

*A stock solution was prepared as described above in the general procedure, relative to 0.68 μmol (1 equiv) of the starting peptide substrate.**

Reaction preparation and execution: In a separate, oven-dried HPLC (or equivalent) vial, was added a magnetic stirbar and the thiol peptide **9** (0.68 μmol, 1 equiv). The vial was sealed with a tight fitting cap or septum, further sealed with Parafilm, and three gentle vacuum/Ar cycles ensued (**Figure SI-3a)**. To the vial containing the peptide and stirbar under Ar was added 68 μL of the DMF stock solution containing the reagents (**Figure SI-3b)**. The reaction vessel was placed in a blue LED photoreactor ~4-8 cm away from the irradiation source. The reaction was allowed to stir for 90 min. The temperature of the reaction was maintained at approximately 30 °C via an overhead fan (see **Figure SI-3c**). Reaction progress was carefully monitored via syringe needle (not to allow significant amounts of oxygen to enter) by HPLC and/or UPLC-MS. Once complete, the slightly darker solution was diluted with purified H_2O (~ 0.5 mL, or until homogeneous). Analytical HPLC samples were then prepared as follows: the crude reaction was diluted to 2.5 mL in 50 % v/v acetonitrile in H_2O with 0.1 % TFA. 200 μL of this sample was then diluted in 800 μL H_2O with 0.1 % TFA and filtered using a PTFE membrane 0.22 μm filter. 200 μL of this dilute sample was then directly used for analytical HPLC analysis.

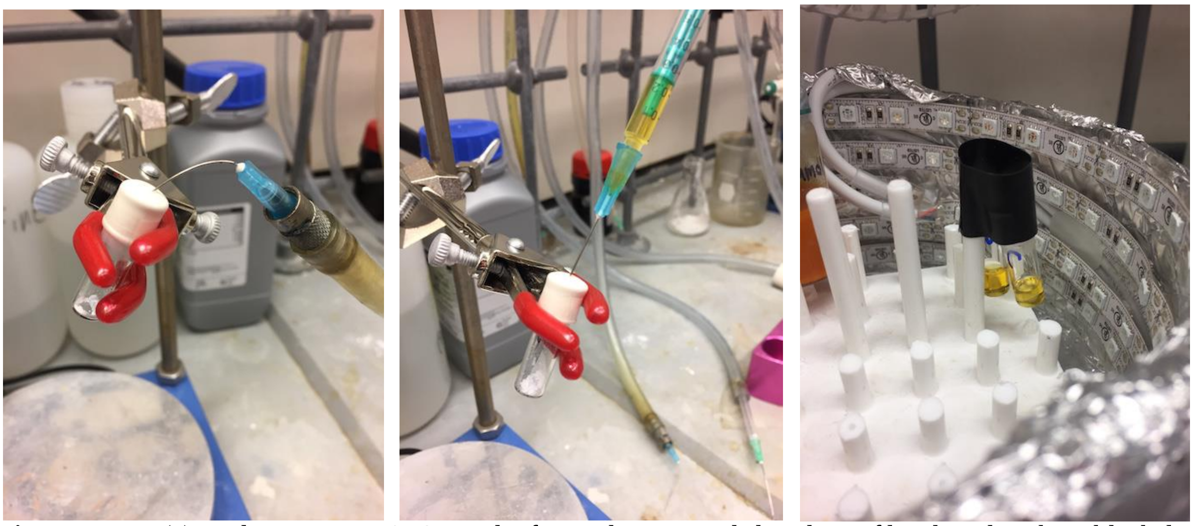

Figure SI- 3. (**a**) Vial containing 0.68 μmol of peptide in a sealed and Parafilmed vial with Schlenk line undergoing vacuum/Ar cycles. (**b**) Schlenk line has been removed, and the DMF stock solution is contained within the syringe just before injection. (**c**) Two reactions side-by-side (conveniently fastened by electrical tape…not essential) in the photoreactor (turned off, fan present) in close proximity to the LED source.

Peptide HTE Screen

An HTE, micro-scale reaction screen (< 0.16 µmol, ~0.1 mg peptide/reaction well) in a 24-well plate was conducted with thiol peptide **9** (primarily because of the limited availability of the peptide substrate) in an oxygen free glovebox. The 24-well plate containing all the reagents (in DMF stock solution, as previously described in the general experimental section) was screwed/sealed shut, free of oxygen, and run outside the glovebox over a bed of blue LEDs for 1 and 3 h. Crude reactions were analyzed by UPLC-MS (results in **Table SI-2**). Cleaner reaction profiles to **10** (see traces below) were generally observed under these oxygen-free conditions than the previously optimized conditions on the benchtop.

***Note:** It has been previously documented that lower photocatalyst loadings (< 0.25 mol %) can be more effective in certain Ni/photoredox cross-coupling reactions,[6] although this trend was not corroborated in this peptide thioarylation chemistry.

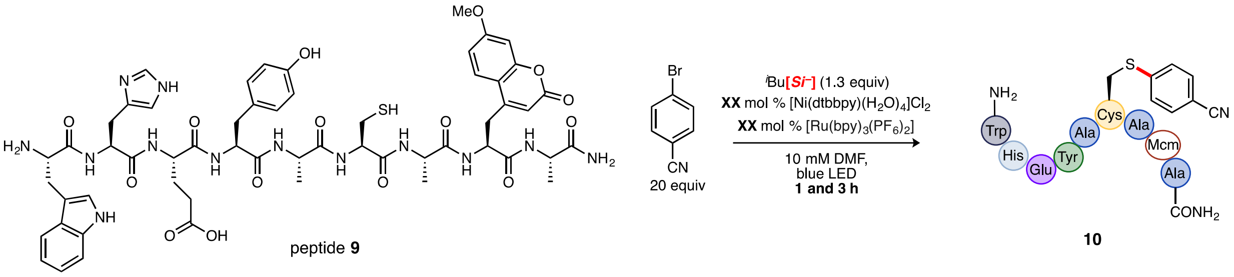

Sum of % Conv SM to Prod			
		time/h	
Ru Loading /mol %	Ni Loading/mol %	1	3
0.1	2.5	3.2	3.2
	5	3.0	5.1
	10	7.8	12.6
0.25	2.5	3.9	8.3
	5	11.4	10.6
	10	17.2	27.8
1	2.5	13.6	16.1
	5	23.2	33.1
	10	44.7	64.8
2	2.5	16.1	18.7
	5	30.4	42.6
	10	49.9	71.3

Table SI-2. HTE micro-scale screen with peptide **10** examining various catalytic reaction conditions at 1 and 3h. Product %area/IS %area ratios (normalized against IS giving relative values) are reported in the boxcells and colored coded based on the relative ratios (conversion to product).

UPLC-MS traces (254 nm) of the crude reaction mixture for the highest yielding reaction well for the microscale peptide thioarylation described above. MW product: 1295.40

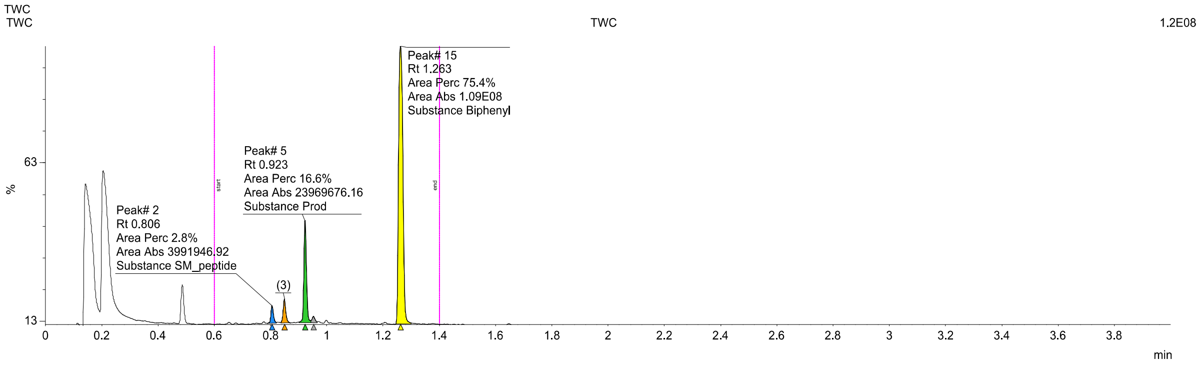

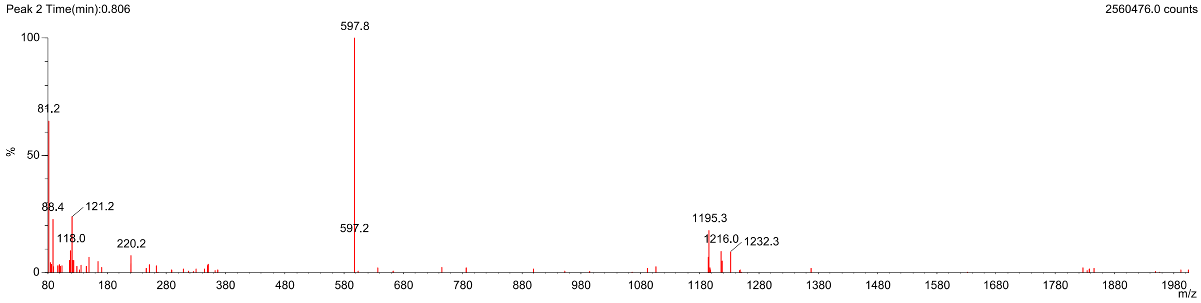

Peak	Mass Found	BPM(+)	BPM(-)	Formula
2				

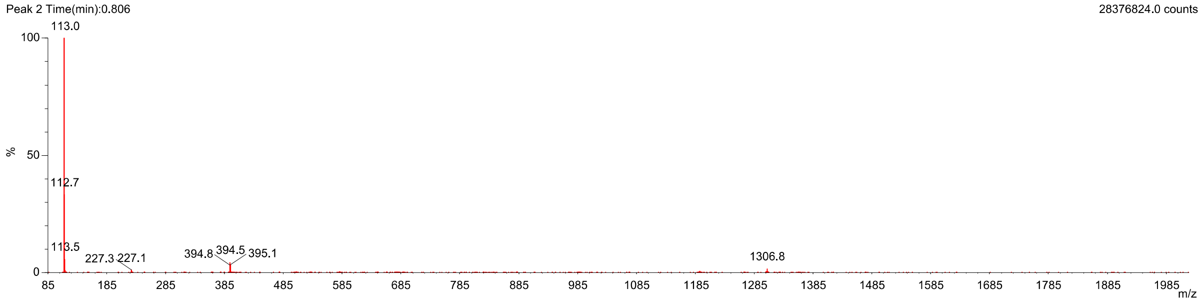

Peak	Mass Found	BPM(+)	BPM(-)	Formula
2				

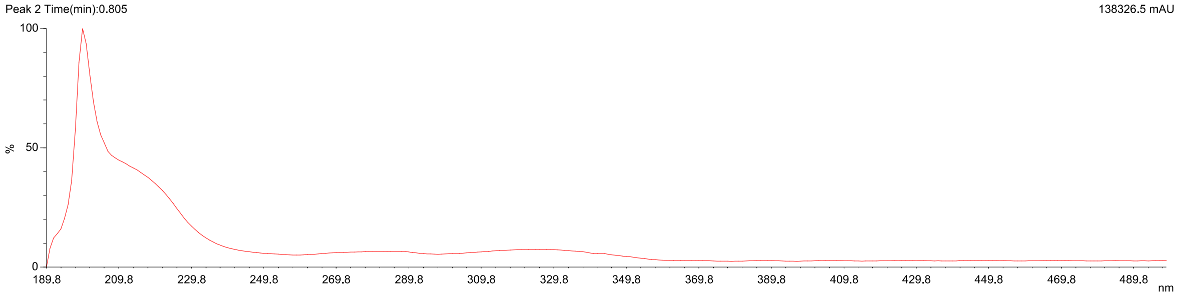

Peak **Mass Found** **BPM(+)** **BPM(-)** **Formula**

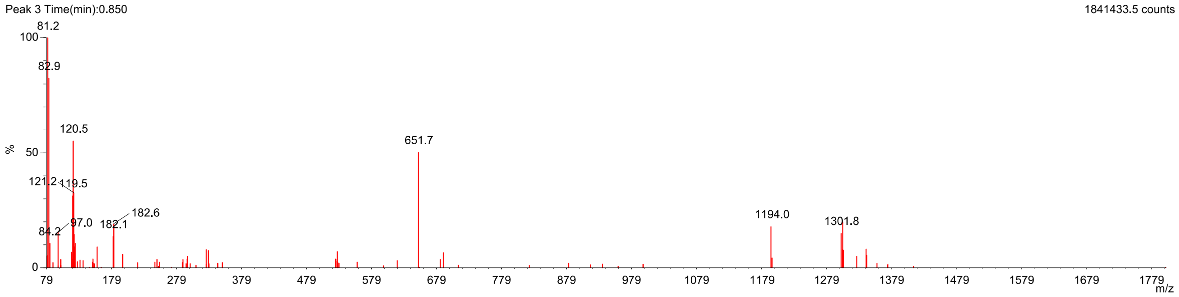

Peak	Mass Found	BPM(+)	BPM(-)	Formula
3				

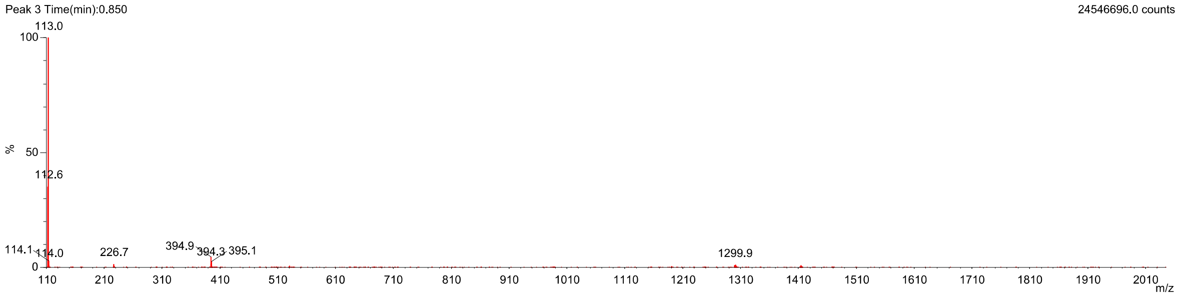

Peak	Mass Found	BPM(+)	BPM(-)	Formula
3				

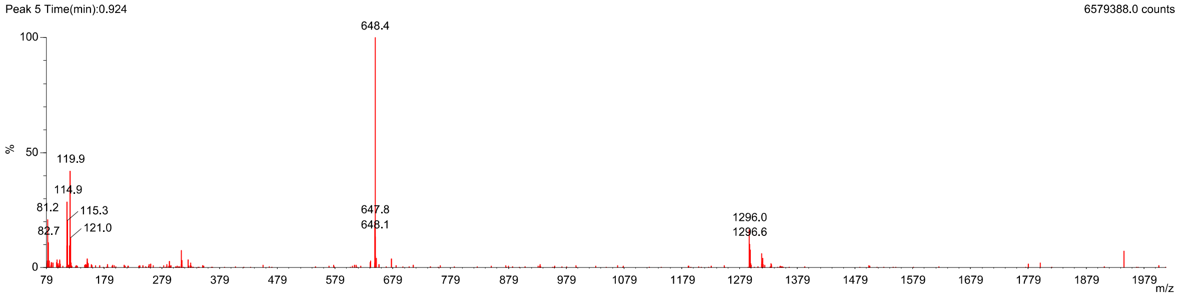

Peak	Mass Found	BPM(+)	BPM(-)	Formula
5				

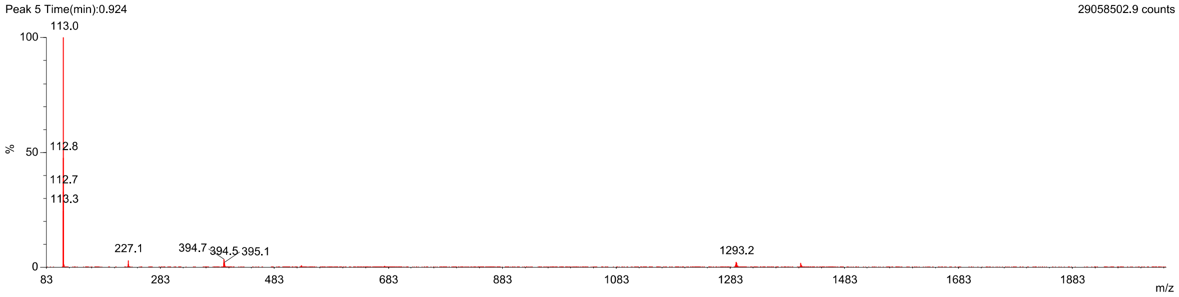

Peak	Mass Found	BPM(+)	BPM(-)	Formula
5				

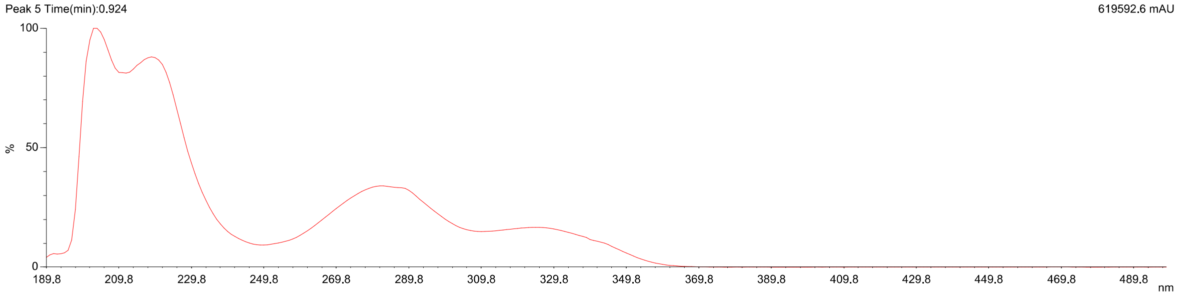

Peak	Mass Found	BPM(+)	BPM(-)	Formula

Compound Characterization Data

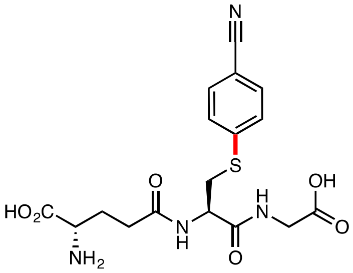

Peptide No. 3: 16 h reaction time. The product was obtained as a light yellow solid (21 mg, 51%) following cold filtration with ice water; ^{1}H NMR (DMSO-d_6, 500.4 MHz): δ 8.74 (br s, 1H), 8.63 (d, *J* = 6.5 Hz, 1H), 7.74 (d, *J* = 8.5 Hz, 2H), 7.49 (d, *J* = 8.5 Hz, 2H), 4.50 (br s, 1H), 3.70 (br s, 2H), 3.52–3.45 (m, 1H), 3.36 (br s, 1H), 3.19 (dd, *J* = 13.0, 10.0 Hz, 1H), 2.36–2.31 (m, 2H), 1.93–1.88 (m, 2H); ^{13}C {^{1}H} NMR (DMSO-d_6, 125.8 MHz): δ 172.4, 171.4, 171.4, 170.4, 144.4, 132.9, 127.2, 119.2, 107.6, 53.5, 52.1, 41.8, 33.7, 31.8, 27.1 ppm; HRMS (ESI) m/z calc. for $C_{17}H_{21}N_4O_6S$ $[M+H]^+$ 409.1182, found 409.1197.

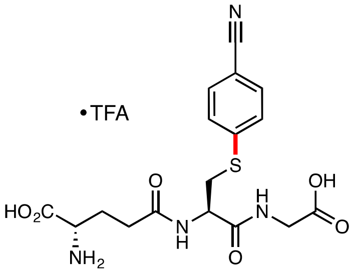

16 h reaction time, 25 μmol scale. Crude workup via extraction protocol. Isolation of **3•TFA** from reverse phase prep HPLC: ^{1}H NMR (DMSO-d_6, 500.4 MHz): δ 8.74 (br s, 1H), 8.49 (d, *J* = 9.5 Hz, 1H), 7.74 (d, *J* = 8.5 Hz, 2H), 7.50 (d, *J* = 8.5 Hz, 2H), 6.57 (br s, 2H), 4.53 (ddd, *J* = 13.5, 9.0, 5.0 Hz, 1H), 3.76-3.72 (m, 2H), 3.54–3.45 (m, 1H), 3.36 (br s, 1H), 3.20-3.15 (m, 1H), 2.37-2.30 (m, 2H), 1.96-1.88 (m, 2H); ^{19}F {^{13}C} NMR (D_2O, 470.8 MHz): δ –75.6 ppm; HRMS (ESI) m/z calc. for $C_{17}H_{21}N_4O_6S$ $[M+H]^+$ 409.1182, found 409.1194.

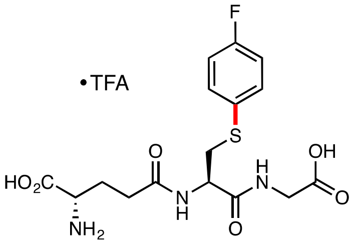

Peptide No. 1a: 16 h reaction time. Crude workup via extraction protocol. The product was obtained as a yellow oil following reverse phase preparatory LC purification (36 mg, 90%); ^{1}H NMR (D_2O, 500.4 MHz): δ 7.36 (dd, *J* = 8.5, 5.5 Hz, 2H), 7.00 (dd, *J* = 9.0, 8.5 Hz, 2H), 4.38 (dd, *J* = 8.0, 5.0 Hz, 1H), 3.95 (t, *J* = 6.5 Hz, 1H), 3.74 (s, 2H), 3.27 (dd, *J* = 14.5, 5.0 Hz, 1H), 3.12 (dd, *J* = 14.5, 8.0 Hz, 1H), 2.41–2.31 (m, 2H), 2.07–2.00 (m, 2H); ^{13}C {^{1}H} NMR (D_2O, 125.8 MHz): δ 173.9, 172.6, 172.1, 171.2, 163.0 (q, $^{2}J_{CF}$ = 36 Hz, TFA), 162.5 (d, $^{1}J_{CF}$ = 245 Hz), 133.6 (d, $^{3}J_{CF}$ = 8.6 Hz) 128.4, 116.1 (d, $^{2}J_{CF}$ = 22 Hz), 53.1, 52.0, 40.8, 35.7, 30.7, 25.2 ppm; ^{19}F {^{13}C} NMR (D_2O, 470.8 MHz): δ –75.6 (s, TFA), –114.5 (q, J_{CF} = 5.7 Hz) ppm; HRMS (ESI) m/z calc. for $C_{16}H_{21}FN_3O_6S$ $[M]^+$ 402.1135, found 402.1113.

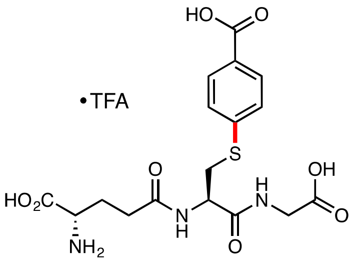

Peptide No. 1b: 16 h reaction time. Crude workup via extraction protocol. The product was obtained as a yellow oil following reverse phase preparatory purification (34 mg, 63%); ^{1}H NMR (D_2O, 500.4 MHz): δ 7.74 (d, *J* = 8.5 Hz, 2H), 7.00 (d, *J* = 8.5 Hz, 2H), 4.52–4.50 (m, 1H), 3.91 (t, *J* = 6.5 Hz, 1H), 3.74 (s, 2H), 3.40 (dd, *J* = 14.5, 9.5 Hz, 1H), 3.25 (dd, *J* = 15.0, 8.0 Hz, 1H), 2.35–2.44 (m, 2H), 2.02–1.96 (m, 2H); ^{13}C {^{1}H} NMR (D_2O, 125.8 MHz): δ 173.8, 172.6, 171.8, 171.1, 169.8, 162.5 (q, $^{2}J_{CF}$ = 36 Hz), 141.7, 130.1, 128.1, 127.2, 116.1 (q, $^{1}J_{CF}$ = 291 Hz), 52.9, 51.9, 40.9, 33.2, 30.7, 25.1 ppm; ^{19}F {^{13}C} NMR (D_2O, 470.8 MHz): δ –75.6 ppm; HRMS (ESI) m/z calc. for $C_{19}H_{22}N_3O_8S$ $[M]^+$ 428.1128, found 428.1120.

Peptide 1b can also be obtained as a tan solid following extraction, precipitation, and cold filtration with ice water (17 mg, 40%).

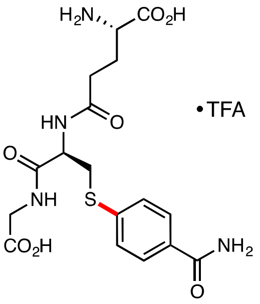

Peptide No. 1c: 16 h reaction time. Crude workup via extraction protocol. The product was obtained as a colorless oil following reverse phase preparatory purification (17 mg, 31%); ^{1}H NMR (D_2O, 500.4 MHz): δ 7.67 (d, *J* = 8.0 Hz, 2H), 7.44 (d, *J* = 8.0 Hz, 2H), 4.55 (dd, *J* = 13.0, 7.5 Hz, 1H), 3.96 (d, *J* = 7.0 Hz, 1H), 3.79 (s, 2H), 3.47 (dd, *J* = 15.0, 5.0 Hz, 1H), 3.32 (dd, *J* = 14.5, 8.0 Hz, 1H), 2.34 (dddd, *J* = 32.0, 23.5, 16.0, 8.0 Hz, 2H), 2.04 (dd, *J* = 14.0, 7.0 Hz, 2H); ^{13}C {^{1}H} NMR (D_2O, 125.8 MHz): δ 173.9, 172.7, 172.1, 171.9, 171.3, 162.7 (q, $^{2}J_{CF}$ = 36 Hz), 139.9, 130.6, 128.9, 128.0, 116.2 (q, $^{1}J_{CF}$ = 290 Hz), 53.0, 52.0, 40.9, 33.6, 30.7, 25.2 ppm; ^{19}F {^{13}C} NMR (D_2O, 470.8 MHz): δ 75.6 ppm; HRMS (ESI) m/z calc. for $C_{17}H_{23}N_4O_7S$ $[M+H]^+$ 427.1287, found 427.1263.

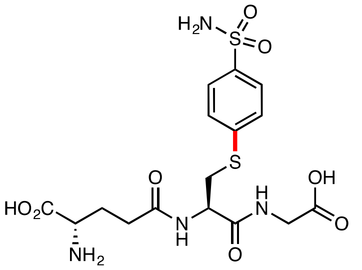

Peptide No. 1d: 24 h reaction time. Crude workup via extraction protocol, and the product was obtained as a light yellow solid (25 mg, 54%) following cold filtration with ice water; ^{1}H NMR (DMSO-d_6, 500.4 MHz): δ 8.68 (br s, 1H), 8.57 (d, *J* = 8.0 Hz, 1H), 7.72 (d, *J* = 8.0 Hz, 2H), 7.49 (d, *J* = 8.5 Hz, 2H), 7.36 (br s, 2H), 4.52-4.44 (m, 1H), 3.70 (d, *J* = 5.0 Hz, 2H), 3.48 (dd, *J* = 13.5, 4.0 Hz, 1H), 3.38-3.30 (m, 1H), 3.17 (dd, *J* = 13.5, 10.0 Hz, 1H), 2.34-2.24

(m, 2H), 1.96-1.82 (m, 2H); ^{13}C {^{1}H} NMR (DMSO-d_6, 125.8 MHz): δ 172.3, 171.3, 171.0, 170.5, 141.6, 141.3, 127.5, 126.2, 53.5, 52.3, 41.7, 34.1, 31.8, 27.1 ppm; HRMS (ESI) m/z calc. for $C_{16}H_{23}N_4O_8S_2$ $[M+H]^+$ 463.0957, found 463.0946.

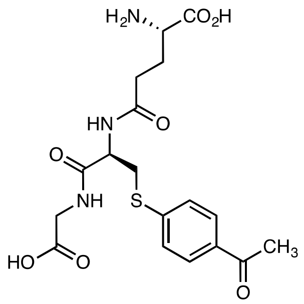

Peptide No.1e: 16 h reaction time. Crude workup via extraction protocol, and the product was obtained as a light tan solid (15 mg, 38%) following filtration with ice water; ^{1}H NMR (DMSO-d_6, 500.4 MHz): δ 8.79 (br s, 1H), 8.59 (br s, 1H), 7.87 (d, J = 8.0 Hz, 2H), 7.44 (d, J = 8.0 Hz, 2H), 4.53–4.45 (m, 1H), 3.70 (s, 2H), 3.51 (dd, J = 14.5, 4.0 Hz, 2H), 3.34–3.28 (m, 1H), 3.17 (dd, J = 10.0, 3.5 Hz, 2H), 2.54 (s, 3H), 2.36-2.28 (m, 2H), 1.95-1.83 (m, 2H); ^{13}C {^{1}H} NMR (DMSO-d_6, 125.8 MHz): δ 197.5, 172.4, 171.3, 170.7, 170.6, 143.6, 134.0, 129.2, 126.7, 53.5, 52.2, 41.7, 33.7, 31.8, 27.1, 26.9 ppm; HRMS (ESI) m/z calc. for $C_{18}H_{24}N_3O_7S$ [M+H] 426.1335, found 426.1316.

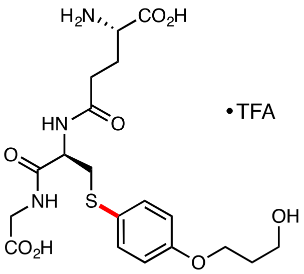

Peptide No. 1f: 48 h reaction time. Crude workup via extraction protocol. The product was obtained as a colorless oil following reverse phase preparatory purification (8 mg, 14%); ^{1}H NMR (D_2O, 500.4 MHz): δ 7.39 (d, J = 8.5 Hz, 2H), 6.94 (d, J = 9.0 Hz, 2H), 4.38 (dd, J = 8.0, 5.0 Hz, 1H), 4.09 (t, J = 4.0 Hz, 2H), 3.74 (s, 2H), 3.70 (t, J = 6.5 Hz, 2H), 3.48 (dd, J = 15.0, 5.0 Hz, 1H), 3.33 (dd, J = 14.5, 8.0 Hz, 1H), 2.38 (t, J = 7.5 Hz, 2H), 2.08-2.00 (m, 2H), 1.96-1.94 (m, 2H); ^{13}C {^{1}H} NMR (D_2O, 125.8 MHz): δ 173.2, 173.2, 173.1, 173.1, 163.0 (q, $^{2}J_{CF}$ = 36 Hz), 161.8, 127.5, 127.1, 116.3 (q, $^{1}J_{CF}$ = 285 Hz), 115.9, 65.5, 58.3, 53.8, 47.6, 41.4, 31.0, 30.9, 30.8, 25.6 ppm; ^{19}F {^{13}C} NMR (D_2O, 470.8 MHz): δ –75.5 ppm; HRMS (ESI) m/z calc. for $C_{19}H_{28}N_3O_8S$ $[M]^+$ 458.1597, found 458.1570.

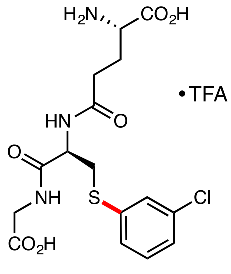

Peptide No. 1g: 16 h reaction time. Crude workup via extraction protocol. The product was obtained as a light tan oil following reverse phase preparatory purification (19 mg, 38%); ^{1}H NMR (D_2O, 500.4 MHz): δ 7.55 (s, 1H), 7.44–7.34 (m, 3H), 4.61 (dd, J = 8.0, 5.5 Hz, 1H), 3.94–3.84 (m, 3H), 3.52 (dd, J = 14.5, 5.0 Hz, 1H), 3.37 (dd, J = 14.5, 8.0 Hz, 1H), 2.51–2.38

(m, 2H), 2.15 (dd, J = 15.0, 8.0 Hz, 2H); ^{13}C {^{1}H} NMR (D_2O, 125.8 MHz): δ 173.0, 172.8, 172.4, 172.4, 162.8 (q, $^{2}J_{CF}$ = 36 Hz), 130.4, 129.9, 128.7, 127.3, 116.2 (q, $^{1}J_{CF}$ = 291 Hz), 53.1, 52.9, 41.0, 34.5, 30.9, 25.2 ppm; ^{19}F {^{13}C} NMR (D_2O, 470.8 MHz): δ –75.5 ppm; HRMS (ESI) m/z calc. for $C_{16}H_{21}ClN_3O_6S$ $[M]^+$ 418.0840, found 418.0859.

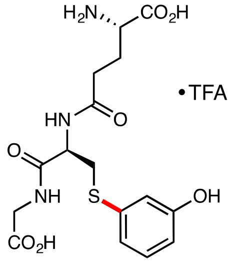

Peptide No. 1h: 24 h reaction time. Crude workup via extraction protocol. The product was btained as a light tan oil following reverse phase preparatory purification (23 mg, 45%); ^{1}H NMR (D_2O, 500.4 MHz): δ 7.28 (t, J = 8.0 Hz, 1H), 7.03 (d, J = 7.5 Hz, 1H), 6.97 (br s, 1H), 6.83 (d, J = 8.5 Hz, 1H), 4.61 (dd, J = 12.0, 5.0 Hz, 1H) 4.08 (t, J = 7.0 Hz, 1H), 3.90 (br s, 2H), 3.48 (dd, J = 14.5, 4.5 Hz, 1H), 3.33 (dd, J = 15.0, 8.0 Hz, 1H), 2.50–2.37 (m, 2H), 2.17 (dd, J = 13.5, 6.5 Hz, 2H); ^{13}C {^{1}H} NMR (D_2O, 125.8 MHz): δ 174.0, 172.7, 172.1, 171.3, 162.6 (q, $^{2}J_{CF}$ = 36 Hz), 155.9, 135.0, 130.5, 122.3, 116.9, 116.1 (q, $^{1}J_{CF}$ = 290 Hz), 114.4, 53.2, 52.0, 40.9, 34.4, 30.7, 25.2 ppm; ^{19}F {^{13}C} NMR (D_2O, 470.8 MHz): δ –75.6 ppm; HRMS (ESI) m/z calc. for $C_{16}H_{22}N_3O_7S$ $[M]^+$ 400.1178, found 400.1176.

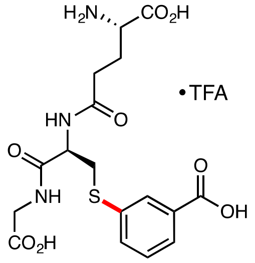

Peptide No. 1i: 24 h reaction time. Crude workup via extraction protocol. The product was obtained as a light yellow oil following reverse phase preparatory purification (12 mg, 22%); ^{1}H NMR (D_2O, 500.4 MHz): δ 8.00 (s, 1H), 7.86 (d, J = 8.0 Hz, 1H), 7.67 (d, J = 8.0 Hz, 1H), 7.45 (t, J = 8.0 Hz, 1H), 4.51 (dd, J = 8.0, 5.0 Hz, 1H), 3.91 (t, J = 6.5 Hz, 1H), 3.77 (d, J = 1.5 Hz, 2H), 3.45 (dd, J = 15.0, 5.0 Hz, 1H), 3.31 (dd, J = 15.0, 8.0 Hz, 1H), 2.36 (dddd, J = 23.5, 19.0, 16.0, 8.0 Hz, 2H), 2.06 (dd, J = 14.5, 7.5 Hz, 2H); ^{13}C {^{1}H} NMR (D_2O, 125.8 MHz): δ 174.0, 172.7, 172.0, 171.8, 169.7, 162.7 (q, $^{2}J_{CF}$ = 36 Hz), 135.6, 134.5, 131.4, 130.6, 129.5, 128.4, 116.1 (q, $^{1}J_{CF}$ = 291 Hz), 53.1, 52.5, 40.9, 34.6, 30.8, 25.1 ppm; ^{19}F {^{13}C} NMR (D_2O, 470.8 MHz): δ –75.6 ppm; HRMS (ESI) m/z calc. for $C_{19}H_{22}N_3O_8S$ $[M]^+$ 428.1128, found 428.1118.

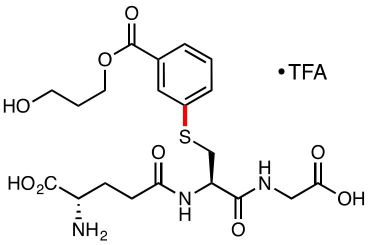

Peptide No. 1j: 16 h reaction time. Crude workup via extraction protocol. The product was obtained as a light tan oil following reverse phase preparatory purification (19 mg, 32%); ^{1}H NMR (D_2O, 500.4 MHz): δ 8.07 (s, 1H), 7.96 (d, J = 8.0 Hz, 1H), 7.76 (d, J = 7.5 Hz, 1H),

7.54 (t, J = 7.5 Hz, 1H), 4.61 (dd, J = 6.5, 5.5 Hz, 1H), 4.49 (t, J = 6.5 Hz, 2H), 4.03 (t, J = 6.5 Hz, 1H), 3.88 (d, J = 2.0 Hz, 2H), 3.83 (t, J = 6.5 Hz, 2H), 3.55 (dd, J = 14.5, 5.0 Hz, 1H), 3.41 (dd, J = 14.5, 8.0 Hz, 1H), 2.49 (dddd, J = 21.8, 19.0, 15.2, 8.0 Hz, 2H), 2.20-2.17 (m, 2H), 2.08 (dd, J = 12.5, 6.0 Hz, 2H); ^{13}C {^{1}H} NMR (D_2O, 125.8 MHz): δ 174.0, 172.7, 172.0, 171.8, 167.9, 162.9 (q, $^{2}J_{CF}$ = 36 Hz), 135.4, 134.5, 131.1, 130.4, 129.4, 128.2, 116.1 (q, $^{1}J_{CF}$ = 289 Hz), 62.9, 58.3, 53.1, 52.4, 40.9, 34.6, 30.9, 30.3, 25.4 ppm; ^{19}F {^{13}C} NMR (D_2O, 470.8 MHz): δ –75.5 ppm; HRMS (ESI) m/z calc. for $C_{20}H_{28}N_3O_9S$ $[M]^+$ 486.1546, found 486.1537.

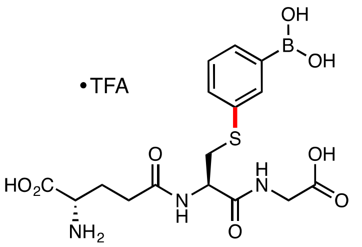

Peptide No. 1k: 24 h reaction time. Crude workup via extraction protocol. To the aqueous layer was added 5 equiv TFA (38 μL), and the resulting mixture was allowed to stir at ambient temperature for 30 min. The aqueous layer was subjected to reverse phase preparatory purification, and the adduct was obtained as a tan oil (25 mg, 46%); ^{1}H NMR (D_2O, 500.4 MHz): δ 7.86 (s, 1H), 7.71 (d, J = 7.5 Hz, 1H), 7.63 (d, J = 8.0 Hz, 1H), 7.47 (t, J = 7.0 Hz, 1H), 4.60 (dd, J = 8.0, 5.0 Hz, 1H), 4.04 (t, J = 7.0 Hz, 1H), 3.87 (d, J = 2.5 Hz, 2H), 3.52 (dd, J = 15.0, 5.0 Hz, 1H), 3.38 (dd, J = 14.5, 8.0 Hz, 1H), 2.46 (dddd, J = 30.5, 22.5, 15.0, 7.0 Hz, 2H), 2.17 (dd, J = 14.5, 7.5 Hz, 2H); ^{13}C {^{1}H} NMR (D_2O, 125.8 MHz): δ 174.0, 172.7, 172.1, 171.7, 135.8, 133.2, 133.1, 132.5, 128.9, 53.2, 52.4, 40.9, 34.8, 30.8, 25.3 ppm; ^{19}F {^{13}C} NMR (D_2O, 470.8 MHz): δ –75.5 ppm; ^{11}B {^{13}C} NMR (D_2O, 400.1 MHz): δ 17.0 ppm; HRMS (ESI) m/z calc. for $C_{16}H_{23}BN_3O_8S$ $[M]^+$ 428.1299, found 428.1311.

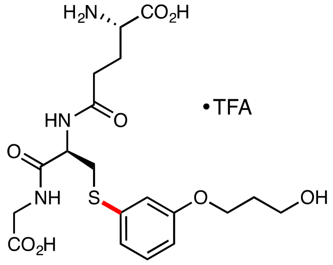

Peptide No. 1l: 24 h reaction time. Crude workup via extraction protocol. The product was obtained as a colorless oil following reverse phase preparatory purification (33 mg, 56%); ^{1}H NMR (D_2O, 500.4 MHz): δ 7.33 (t, J = 8.0 Hz, 1H), 7.11–7.07 (m, 2H), 6.94 (d, J = 8.0 Hz, 1H), 4.58 (dd, J = 7.5, 5.0 Hz, 1H), 4.16 (t, J = 6.0 Hz, 2H), 4.05 (t, J = 6.5 Hz, 1H), 3.87 (t, J = 2.0 Hz, 2H), 3.79 (t, J = 6.5 Hz, 2H), 3.48 (dd, J = 15.0, 5.0 Hz, 1H), 3.33 (dd, J = 14.5, 5.0 Hz, 1H), 2.46 (dddd, J = 28.5, 23.5, 16.0, 8.0 Hz, 2H), 2.16 (dd, J = 14.5, 7.5 Hz, 2H), 2.05-2.01 (m, 2H); ^{13}C {^{1}H} NMR (D_2O, 125.8 MHz): δ 173.9, 172.7, 172.1, 171.4, 162.8 (q, $^{2}J_{CF}$ = 36 Hz), 158.5, 135.0, 130.3, 123.1, 116.6, 116.1 (q, $^{1}J_{CF}$ = 288 Hz), 115.0, 65.3, 58.3, 53.2, 52.1, 40.9, 34.5, 30.8, 30.7, 25.3 ppm; ^{19}F {^{13}C} NMR (D_2O, 470.8 MHz): δ –75.6 ppm; HRMS (ESI) m/z calc. for $C_{19}H_{28}N_3O_8S$ $[M]^+$ 458.1597, found 458.1576.

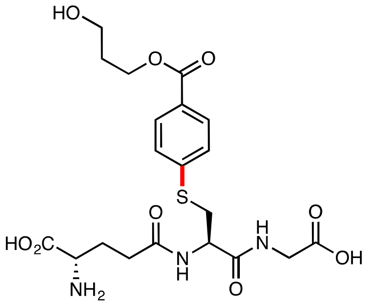

Peptide No. 1m: 16 h reaction time. Crude workup via extraction protocol. The product was obtained as a light yellow solid (18 mg, 37%) following cold filtration with ice water; ^{1}H NMR (DMSO-d_6, 500.4 MHz): δ 8.71–8.56 (m, 2H), 7.84 (dd, J = 15.0, 8.5 Hz, 2H), 7.42 (dd, J = 14.5, 8.0 Hz, 2H), 4.49 (s, 1H), 4.33–4.25 (m, 2H), 3.70 (br s, 1H), 3.57–3.46 (m, 3H), 3.39–3.36 (m, 1H), 3.17 (dd, J = 13.5, 10.0 Hz, 1H), 2.32 (br s, 2H), 2.00–1.77 (m, 4H), 0.86 (br s, 1H); ^{13}C {^{1}H} NMR (DMSO-d_6, 125.8 MHz): δ 172.3, 171.4, 171.1, 170.5, 165.8, 143.6, 130.0, 126.9, 126.8, 62.3, 57.6, 53.4, 52.2, 41.8, 33.9, 31.9, 31.8, 27.1 ppm; HRMS (ESI) m/z calc. for $C_{20}H_{28}N_3O_9S$ $[M]^+$ 486.1546, found 486.1538.

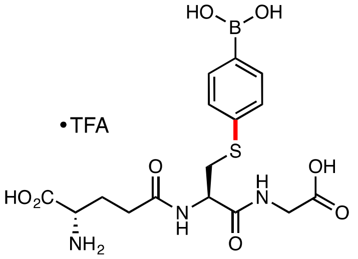

Peptide No. 1n: 24 h reaction time. Crude workup via extraction protocol. To the aqueous layer was added 5 equiv TFA (38 μL), and the mixture was allowed to stir at ambient temperature for 30 min. The aqueous layer was subjected to reverse phase preparatory purification, and the adduct was obtained as a colorless oil (49 mg, 83%); ^{1}H NMR (DMSO-d_6, 500.4 MHz): δ 8.42-8.38 (m, 2H), 8.27 (br s, 2H). 7.71 (d, J = 7.0 Hz, 2H), 7.29 (d, J = 7.0 Hz, 2H), 4.51 (ddd, J = 13.5, 8.5, 4.5 Hz, 1H), 3.73 (t, J = 5.5 Hz, 1H), 3.73 (m, 2H), 3.40-3.35 (m, 1H), 3.11-3.06 (m, 1H), 2.41-2.31 (m, 2H), 2.07-1.97 (m, 2H);^{13}C {^{1}H} NMR (D_2O, 125.8 MHz): δ 173.9, 172.7, 172.0, 171.3, 162.6 (q, $^{2}J_{CF}$ = 36 Hz), 137.2, 134.3, 128.9, 116.2 (q, $^{1}J_{CF}$ = 286 Hz), 53.2, 52.0, 40.9, 33.8, 30.7, 25.2 ppm; ^{11}B NMR (D_2O, 128.4 MHz): δ 19.0 ppm; ^{19}F {^{13}C} NMR (D_2O, 470.8 MHz): δ –75.6 ppm; HRMS (ESI) m/z calc. for $C_{16}H_{23}BN_3O_8S$ $[M]^+$ 428.1299, found 428.1312.

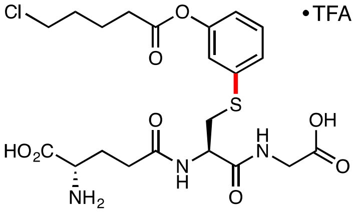

Peptide No. 1o: 16 h reaction time. Crude workup via extraction protocol. The product was obtained as a light tan oil following reverse phase preparatory purification (22 mg, 35%); ^{1}H NMR (D_2O, 500.4 MHz): δ 7.50–7.40 (m, 2H), 7.27 (s, 1H), 7.09 (d, J = 7.5 Hz, 1H), 4.62 (m, 1H), 4.04 (t, J = 5.5 Hz, 1H), 3.92 (s, 2H), 3.72 (br s, 2H), 3.52-3.45 (m,1H), 3.37 (dd, J = 14.5, 8.5 Hz, 1H), 2.75 (d, J = 1.5 Hz, 2H), 2.54-2.43 (m, 2H), 2.23-2.13 (m, 2H), 1.92 (m, 4H); ^{13}C {^{1}H} NMR (D_2O, 125.8 MHz): δ 175.2, 174.0, 172.7, 172.1, 171.5, 162.8 (q, $^{2}J_{CF}$ = 36 Hz), 135.6, 130.3, 123.2, 120.5, 119.7, 112.7, 116.2 (q, $^{1}J_{CF}$ = 291 Hz), 53.1, 52.2, 44.9,

40.9, 34.5, 32.9, 31.0, 30.7, 25.3, 21.5 ppm; ^{19}F {^{13}C} NMR (D_2O, 470.8 MHz): δ –75.5 ppm; HRMS (ESI) m/z calc. for $C_{21}H_{29}ClN_3O_8S$ $[M]^+$ 518.1364, found 518.1364.

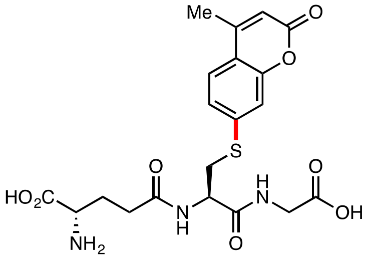

Peptide No. 1p: 24 h reaction time. Crude workup via extraction protocol. The product was obtained as a light yellow solid (25 mg, 54%) following cold filtration with ice water; ^{1}H NMR (DMSO-d_6, 500.4 MHz): δ 8.77 (br s, 1H), 8.63 (d, J = 8.0 Hz, 1H), 7.67 (d, J = 8.5 Hz, 1H), 7.35 (s, 1H), 7.29 (d, J = 8.0 Hz, 1H), 6.31 (s, 1H), 4.51 (dd, J = 12.0, 8.0 Hz, 1H), 3.71 (br s, 2H), 3.52 (dd, J = 13.5, 4.0 Hz, 1H), 3.35 (t, J = 6.0 Hz, 1H), 3.19 (dd, J = 13.5, 10.0 Hz, 1H), 2.40 (s, 3H), 2.33 (m, 2H), 1.98–1.82 (m, 2H); ^{13}C {^{1}H} NMR (D_2O, 125.8 MHz): δ 172.4, 171.2, 170.6, 160.0, 153.7, 153.5, 142.3, 126.1, 123.3, 117.4, 114.0, 113.6, 55.3, 53.4, 52.2, 41.6, 34.0, 31.8, 27.1, 18.4 ppm; HRMS (ESI) m/z calc. for $C_{20}H_{24}N_3O_8S$ $[M]^+$ 466.1284, found 466.1311.

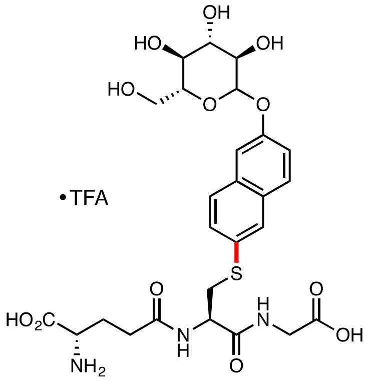

Peptide No. 1r: 36 h reaction time. Crude workup via extraction protocol. The product was obtained as a light tan oil following reverse phase preparatory purification (24 mg, 40%); ^{1}H NMR (DMSO-d_6, 500.4 MHz), major diastereomer: δ 8.50-8.45 (m, 2H), 8.23 (br s, 2H), 7.85 (m, 1H), 7.81-7.73 (m, 2H), 7.70-7.66 (m, 1H), 7.48-7.40 (m, 2H), 7.27-7.23 (m, 1H), 5.50 (d, J = 5.0 Hz, 1H), 5.44-5.31 (m, 1H), 5.23 (d, J = 8.0 Hz, 1H, -OH) 5.18-5.08 (m, 1H), 5.09 (d, J = 8.0 Hz, 1H, -OH), 5.00 (d, J = 7.0 Hz, 1H, -OH), 4.69 (dd, J = 12.0, 2.0 Hz, 1H, -OH), 4.56-4.45 (m, 1H), 3.94-3.86 (m, 1H), 3.73 (d, J = 6.0 Hz, 2H), 3.72-3.70 (m, 1H), 3.58 (t, J = 8.5 Hz, 1H), 3.50-3.47 (m, 1H), 3.24-3.20 (m, 1H), 3.16-3.10 (m, 1H), 2.36 (dddd, J = 28.5, 23.5, 16.0, 8.0 Hz, 2H), 2.01 (dddd, J = 26.0, 21.5, 14.5, 7.5 Hz, 2H); ^{13}C {^{1}H} NMR (DMSO-d_6, 125.8 MHz), major diastereomer: δ 171.6, 171.4, 171.3, 170.7, 158.2 (q, $^2J_{CF}$ = 38 Hz), 156.7, 155.6, 155.4, 132.7, 131.4, 129.9, 129.0, 128.0, 127.1, 121.4, 119.9, 117.7 (q, $^1J_{CF}$ = 280 Hz), 110.9, 101.0, 77.6, 76.6, 75.0, 73.6, 70.1, 68.0, 52.2, 31.2, 26.5 ppm; ^{19}F {^{13}C} NMR (DMSO-d_6, 470.8 MHz): δ –75.6 ppm; HRMS (ESI) m/z calc. for $C_{26}H_{34}N_3O_{12}S$ $[M+H]^+$ 612.1863, found 612.1868.

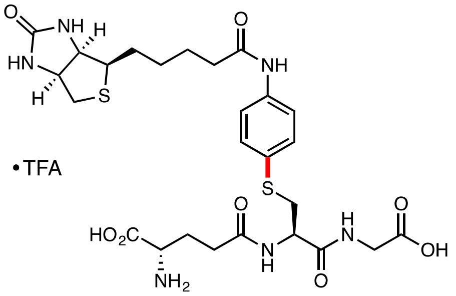

Peptide No. 1q: 48 h reaction time (*p*-amino amide is sluggish). Crude workup via extraction protocol. The product was obtained as a colorless oil following reverse phase preparatory purification (46 mg, 61%); ^{1}H NMR (DMSO-d_6, 500.4 MHz): δ 9.94 (s, 1H), 8.38-8.32 (m, 2H), 8.28 (d, *J* = 3.5 Hz, 3H), 7.56 (d, *J* = 8.5 Hz, 2H), 7.31 (d, *J* = 9.0 Hz, 2H), 6.44 (br s, 1H), 4.42 (ddd, *J* = 14.0, 9.0, 5.0 Hz, 1H), 3.31 (dd, *J* = 7.5, 5.0 Hz, 1H), 4.14 (dd, *J* = 7.5, 4.5 Hz, 1H), 3.97–3.91 (m, 1H), 3.72 (d, *J* = 6.0 Hz, 2H), 3.25 (dd, *J* = 13.5, 5.0 Hz, 1H), 3.12 (ddd, *J* = 10.5, 8.5, 6.0 Hz, 1H), 2.97 (dd, *J* = 13.5, 9.5 Hz, 1H), 2.82 (dd, *J* = 12.5, 5.0 Hz, 1H), 2.58 (d, *J* = 12 Hz, 2H), 2.44-2.37 (m, 4H), 2.10-1.95 (m, 2H), 1.69-1.56 (m, 3H), 1.53-1.45 (m, 1H), 1.42-1.31 (m, 2H); ^{13}C {^{1}H} NMR (DMSO-d_6, 125.8 MHz): δ 171.6, 171.4, 171.2, 171.2, 170.6, 163.1, 158.8 (q, $^2J_{CF}$ = 36 Hz), 138.6, 131.2, 128.7, 120.0, 116.2 (q, $^1J_{CF}$ = 291 Hz), 61.4, 59.6, 55.7, 52.2, 52.0, 41.0, 37.0, 36.6, 31.0, 28.6, 28.4, 26.3, 25.4 ppm; ^{19}F {^{13}C} NMR (DMSO-d_6, 470.8 MHz): δ –74.8 ppm; HRMS (ESI) m/z calc. for $C_{26}H_{37}N_6O_8S_2$ $[M+H]^+$ 625.2114, found 625.2112.

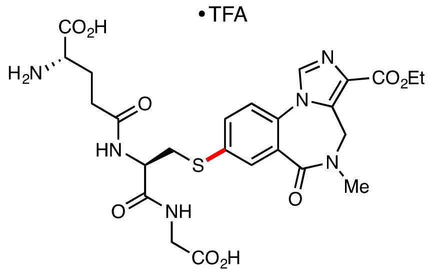

Peptide No. 1s: 36 h reaction time. Crude workup via extraction protocol. The product was obtained as a yellow oil following reverse phase preparatory purification (35 mg, 50%); ^{1}H NMR (D_2O, 500.4 MHz): δ 9.38 (s, 1H), 7.95 (s, 1H), 7.81 (s, 1H), 7.67 (d, *J* = 8.5 Hz, 1H), 5.12 (d, *J* = 16.5 Hz, 1H), 4.62-4.55 (m, 2H), 4.52-4.47 (m, 2H), 4.07 (br s, 1H), 3.90 (s, 2H), 3.61–3.56 (m, 1H), 3.41-3.37 (m, 1H), 3.21 (s, 3H), 2.52 (br s, 2H), 2.19–2.14 (m, 2H), 1.42 (t, *J* = 7.0 Hz, 3H); ^{13}C {^{1}H} NMR (D_2O, 125.8 MHz): δ 174.0, 172.6, 171.8, 171.2, 166.8, 162.4 (q, $^2J_{CF}$ = 36 Hz), 159.0, 138.4, 135.5, 133.7, 131.7, 123.8, 121.0, 120.9, 119.6, 116.1 (q, $^1J_{CF}$ = 289 Hz), 116.0, 63.4, 52.6, 52.0, 41.8, 40.9, 35.7, 34.0, 30.8, 25.2, 13.2 ppm; ^{19}F {^{13}C} NMR (D_2O, 470.8 MHz): δ –75.6 ppm; HRMS (ESI) m/z calc. for $C_{25}H_{31}N_6O_9S$ $[M]^+$ 591.1873, found 591.1877.

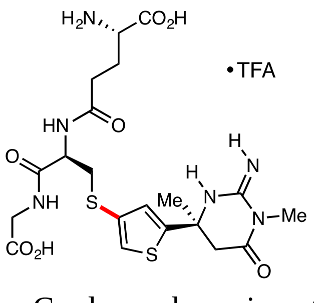

Peptide No. 1t: 36 h reaction time. Crude workup via extraction protocol. The product was obtained as a light tan oil following reverse phase preparatory purification (24 mg, 38%); ^{1}H

NMR (D_2O, 500.4 MHz): δ 7.43 (s, 1H), 7.11 (s, 1H), 4.48 (dd, *J* = 7.0, 1.0 Hz, 1H), 4.07 (t, *J* = 6.5 Hz, 1H), 3.90 (s, 2H), 3.45-3.36 (m, 2H), 3.29-3.28 (m, 1H), 3.26 (s, 3H), 3.19 (dd, *J* = 14.5, 8.0 Hz, 1H), 2.57 (t, *J* = 7.5 Hz, 2H), 2.24 (dd, *J* = 13.5, 6.5 Hz, 2H), 1.83 (s, 3H); ^{13}C {^{1}H} NMR (D_2O, 125.8 MHz): δ 174.1, 172.7, 172.2, 171.8, 168.1, 162.9 (q, $^{2}J_{CF}$ = 33 Hz), 154.9, 147.7, 129.1, 127.0, 126.4, 116.6 (q, $^{1}J_{CF}$ = 291 Hz), 53.7, 52.8, 52.4, 43.7, 40.9, 35.5, 30.8, 28.5, 28.2, 25.4 ppm; ^{19}F {^{13}C} NMR (D_2O, 470.8 MHz): δ –75.6 ppm; HRMS (ESI) m/z calc. for $C_{20}H_{29}N_6O_7S_2$ $[M+H]^+$ 529.1539, found 529.1541.

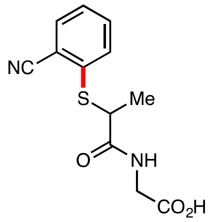

(±)–Tiopronin adduct 7a. 24 h reaction time. Crude reaction diluted with 1 mL of H_2O (no extraction) and subjected to purification. The product was obtained as a light tan oil following reverse phase preparatory LC purification (16 mg, 61%); ^{1}H NMR (DMSO-d_6, 500.4 MHz): δ 8.50 (t, J = 5.5 Hz, 1H), 7.83 (d, J = 7.5 Hz, 1H), 7.67-7.65 (m, 2H), 7.45-7.41 (m, 1H), 4.50 (br s, 1H) 4.15 (q, J = 7.0 Hz, 1H), 3.77 (dd, J = 5.5, 1.5 Hz, 2H), 1.41 (d, J = 7.0 Hz, 3H). ^{13}C {^{1}H} NMR (DMSO-d_6, 125.8 MHz): δ 171.3, 171.2, 139.0, 134.2, 133.9, 131.4, 127.7, 117.4, 113.6, 45.3, 41.3, 18.3 ppm; HRMS (ESI) m/z calc. for $C_{12}H_{12}N_2NaO_3S$ $[M+Na]^+$ 287.0466, found 287.0490.

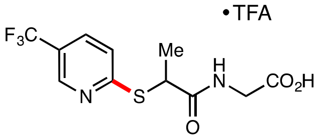

(±)–Tiopronin adduct 7b. 24 h reaction time. Crude reaction diluted with 1 mL of H_2O (no extraction) and subjected to purification. Obtained as a tan solid following reverse phase preparatory LC purification as the TFA salt (18 mg, 43%); ^{1}H NMR (CD_3OD, 500.4 MHz): δ 8.68 (d, J = 1.0 Hz, 1H), 7.86 (dd, J = 3.5, 2.0 Hz, 1H), 7.45 (d, J = 8.5 Hz, 1H), 4.61 (q, J = 7.5 Hz, 1H), 3.71 (s, 2H), 1.60 (d, J = 7.5 Hz, 3H). ^{13}C {^{1}H} NMR (CD_3OD, 125.8 MHz): δ 174.4, 168.9, 163.1, 145.6, 132.9 (d, $^{4}J_{CF}$ = 3.1 Hz), 122.4 (q, $^{2}J_{CF}$ = 34.7 Hz), 121.4, 116.7 (q, $^{1}J_{CF}$ = 291 Hz), 41.5, 39.9, 16.4 ppm; ^{19}F {^{13}C} NMR (CD_3OD, 470.8 MHz): δ –63.7, –76.9 (TFA) ppm; HRMS (ESI) m/z calc. for $C_{11}H_{12}F_3N_2O_3S$ $[M+H]^+$ 309.0521, found 309.0519.

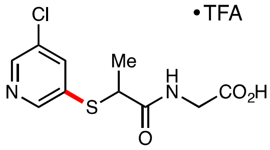

(±)–Tiopronin adduct 7c. 36 h reaction time. Crude reaction diluted with ~1 mL of H_2O (no extraction) and subjected to purification. The product was obtained as a colorless solid following reverse phase preparatory LC purification as the TFA salt (22 mg, 56%); ^{1}H NMR (CD_3OD, 500.4 MHz): δ 8.51 (d, J = 2.0 Hz, 1H), 8.45 (d, J = 2.0 Hz, 1H), 8.03 (dd, J = 2.0, 2.0 Hz, 1H), 3.93 (q, J = 7.5 Hz, 1H), 3.87 (s, 2H), 1.48 (d, J = 7.5 Hz, 3H). ^{13}C {^{1}H} NMR (CD_3OD, 125.8 MHz): δ 174.9, 172.9, 151.8, 148.7, 141.6, 134.6, 133.9, 47.9, 42.4, 18.6 ppm; ^{19}F {^{13}C} NMR (CD_3OD, 470.8 MHz): δ –76.9 ppm; HRMS (ESI) m/z calc. for $C_{10}H_{12}ClN_2O_3S$ $[M+H]^+$ 275.0257, found 275.0259

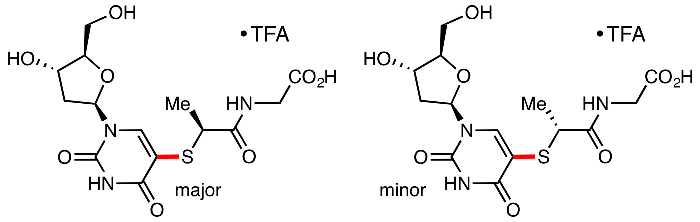

mixture of diastereomers

(±)–Tiopronin adduct 7d. 36 h reaction time. Crude reaction diluted with ~1 mL of H_2O (no extraction) and subjected to purification. The product was obtained as a colorless oil following reverse phase preparatory LC purification as the TFA salts (12 mg, 31%). Shallow

solvent gradient (prep-LC) was employed for adequate separation (0-5% MeCN in H_2O, 2 min; 5-15% MeCN in H_2O, 6 min; 15-40% MeCN in H_2O, 5 min; 35-90% MeCN in H_2O, 4 min). Mixture of diastereomers isolated in ~2:1 dr, (pictured), major diastereomer listed: ^1H NMR (D_2O, 500.4 MHz): δ 8.30 (s, 1H), 6.27 (dd, J = 6.5, 6.5 Hz, 1H), 4.49 (d, J = 4.5 Hz, 1H), 4.06 (dd, J = 8.5, 3.5 Hz, 1H), 4.03 (s, 2H), 3.89 (dd, J = 4.5, 3.5 Hz, 1H), 3.80 (dd, J = 4.5, 3.5 Hz, 1H), 3.39 (dd, J = 7.0, 7.0 Hz, 1H), 2.49-2.42 (m, 3H), 1.52 (d, J = 7.0 Hz, 3H). ^{13}C {^1H} NMR (D_2O, 125.8 MHz): δ 177.1, 173.1, 164.9, 161.8, 150.8, 141.2, 96.3, 86.7, 85.7, 70.1, 60.8, 41.2, 38.9, 20.5 ppm; ^{19}F {^{13}C} NMR (D_2O, 470.8 MHz): δ –75.6 ppm; HRMS (ESI) m/z calc. for $C_{14}H_{20}N_3NaO_8S$ $[M+Na]^+$ 412.0791, found 412.0775.

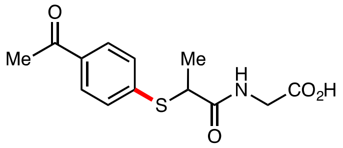

(±)–Tiopronin adduct 7e. 24 h reaction time. Crude reaction diluted with 1 mL of H_2O (no extraction) and subjected to purification. The product was obtained as a colorless oil following reverse phase preparatory LC purification as the TFA salt (18 mg, 64%); ^1H NMR ($CDCl_3$, 500.4 MHz): δ 9.69 (br s, 1H), 7.84 (d, J = 7.5 Hz, 2H), 7.34 (d, J = 7.5 Hz, 2H), 7.34-7.33 (m, 1H), 4.03 (m, 3H), 2.56 (s, 3H), 1.60 (d, J = 6.5 Hz, 3H). ^{13}C {^1H} NMR ($CDCl_3$, 125.8 MHz): δ 198.0, 176.0, 174.7, 173.0, 140.9, 135.6, 129.0, 128.3, 45.4, 26.4, 18.0 ppm; HRMS (ESI) m/z calc. for $C_{13}H_{16}NO_4S$ $[M+H]^+$ 282.0800, found 282.0773.

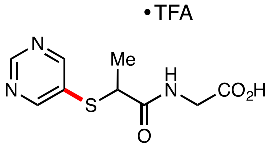

(±)–Tiopronin adduct 7f. 24 h reaction time. Crude reaction diluted with 1 mL of H_2O (no extraction) and subjected to purification. The product was obtained as a colorless oil following reverse phase preparatory LC purification as the TFA salt (20 mg, 56%); ^1H NMR (CD_3OD, 500.4 MHz): δ 9.06, (s, 1H), 8.88 (s, 2H), 3.91 (q, J = 7.0 Hz, 1H), 3.87 (d, J = 3.5 Hz, 2H), 1.48 (d, J = 7.0 Hz, 3H). ^{13}C {^1H} NMR (CD_3OD, 125.8 MHz): δ 174.3, 172.4, 161.8, 158.2, 131.5, 116.1 (q, $^1J_{CF}$ = 295 Hz, TFA), 47.2, 41.9, 18.0 ppm; ^{19}F {^{13}C} NMR (CD_3OD, 470.8 MHz): δ –77.6 ppm; HRMS (ESI) m/z calc. for $C_9H_{12}N_3O_3S$ $[M+H]^+$ 242.0599, found 242.0597.

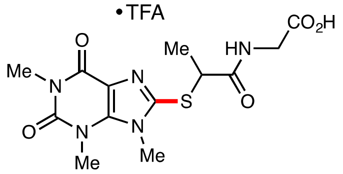

(±)–Tiopronin adduct 7g. 16 h reaction time. Crude reaction diluted with 1 mL of H_2O and 300 µL of DMSO (no extraction) and subjected to purification. The product was obtained as a colorless solid following reverse phase preparatory LC purification as the TFA salt (31 mg, 66%); ^1H NMR (CD_3OD, 500.4 MHz): δ 4.48 (q, J = 7.0 Hz, 1H), 3.91 (s, 2H), 3.84 (s, 3H), 3.52 (s, 3H), 3.31 (s, 3H), 1.61 (d, J = 7.0 Hz, 3H). ^{13}C {^1H} NMR (CD_3OD, 125.8 MHz): δ 173.8, 172.4, 155.9, 152.9, 150.4, 149.6, 110.0, 46.5, 42.2, 32.9, 30.1, 28.2, 18.0 ppm; ^{19}F {^{13}C} NMR (CD_3OD, 470.8 MHz): δ –76.9 ppm; HRMS (ESI) m/z calc. for $C_{13}H_{18}N_5O_5S$ $[M+H]^+$ 356.1029, found 356.1038.

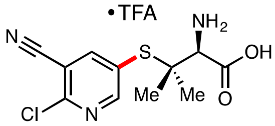

D-Penicillamine adduct 8a. 48 h reaction time. 2 equiv of the aryl bromide was employed and the reaction was run for 36 h. The product was obtained as a light maroon solid following reverse phase preparatory LC purification as the TFA salt (11 mg, 28%, ≥ 95% pure); ^{1}H NMR (DMSO-d_6, 500.4 MHz): δ 8.84 (d, J = 2.5 Hz, 1H), 8.72 (d, J = 2.5 Hz, 1H), 8.5 (br s, 2H), 3.88 (br m, 1H), 1.41 (s, 3H), 1.40 (s, 3H). ^{13}C {^{1}H} NMR (DMSO, 125.8 MHz): δ 168.7, 160.1 158.5 (q, $^{2}J_{CF}$ = 33 Hz, TFA), 152.8, 151.7, 126.7, 116.8 (q, $^{1}J_{CF}$ = 294 Hz, TFA) 114.9, 110.8, 59.4, 50.2, 27.0, 25.1 ppm; ^{19}F {^{13}C} NMR (DMSO-d_6, 470.8 MHz): δ –74.1 ppm; HRMS (ESI) m/z calc. for $C_{11}H_{14}ClN_3O_2S$ $[M+2H]^{2+}$ 143.5248, found 143.5232. *Ru(bpy)$_3$ (bpy = bipyridine) or Ru(bpy)$_3$·TFA is present in the final sample [assuming the maximum amount, 2 mol % Ru(bpy)$_3$] as an impurity unable to be entirely removed by washing or reverse phase prep-LC; ^{1}H NMR (DMSO-d_6, 500.4 MHz): δ 8.55 (d, J = 5.0 Hz, 2H), 8.16 (dd, J = 8.0, 1.5 Hz, 2H), 7.73 (d, J = 5.0 Hz, 2H), 7.53 (ddd, J = 8.0, 5.5, 1.5 Hz, 2H) and represents ≤ 4% impurity [assuming 3 equiv of bpy present per maximum of 2 mol % Ru(bpy)$_3$].

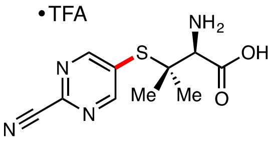

D-Penicillamine adduct 8b. 36 h reaction time. 2 equiv of the aryl bromide was employed and the reaction was run for 40 h. The product was obtained as a light tan oil following reverse phase preparatory LC purification as the TFA salt (15 mg, 42%); ^{1}H NMR (D_2O, 500.4 MHz): δ 9.14 (s, 2H), 3.81 (s, 1H), 1.57 (s, 3H), 1.52 (s, 3H). ^{13}C {^{1}H} NMR (D_2O, 125.8 MHz): δ 167.5, 163.3, 160.7 (q, $^{2}J_{CF}$ = 35 Hz, TFA), 141.2, 128.4, 112.9, 59.2, 48.4, 24.8, 22.3 ppm; ^{19}F {^{13}C} NMR (D_2O, 470.8 MHz): δ –75.6 ppm; HRMS (ESI) m/z calc. for $C_{10}H_{13}N_4O_2S$ $[M+H]^{+}$ 253.0759, found 253.0761.

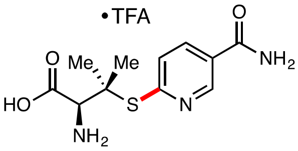

D-Penicillamine adduct 8c. 36 h reaction time. The product was obtained as a white oil following reverse phase preparatory LC purification as the TFA salt (18 mg, 48%); ^{1}H NMR (DMSO-d_6, 500.4 MHz): δ 9.10 (d, J = 2.0 Hz, 1H), 8.86 (d, J = 2.5 Hz, 1H), 8.53 (br s, 2H), 8.45 (dd, J = 2.5, 2.0 Hz, 1H), 8.26 (br s, 2H), 7.74 (br s, 2H), 3.74 (m, 1H), 1.38 (s, 3H), 1.34 (s, 3H). ^{13}C {^{1}H} NMR (DMSO-d_6, 125.8 MHz): δ 168.8, 166.0, 158.9, 158.8 (q, $^{2}J_{CF}$ = 30 Hz, TFA), 150.0, 144.1, 130.6, 126.8, 116.2 (q, $^{1}J_{CF}$ = 290 Hz, TFA), 59.8, 49.4, 27.2, 25.2 ppm; ^{19}F {^{13}C} NMR (DMSO-d_6, 470.8 MHz): δ –74.7 ppm; HRMS (ESI) m/z calc. for $C_{11}H_{16}N_3O_3S$ $[M+H]^{+}$ 270.0912, found 270.0901.

Thioarylated Peptide 10 Data

HPLC and MALDI Analysis of Peptide Cys Arylation Reactions

Analytical HPLC of peptide **10** was performed on a Phenomenex Luna C8 column using gradient **5**, and an injection volume of 200 µL (flow rate of 1.0 mL/min). Absorbance was monitored at 215, 254, and 325 nm. The collected fractions from the product peak were analyzed by MALDI TOF-MS, and to confirm the selectively of the arylation for the Cys residue, tandem MS/MS fragmentation was performed. The fragmentation pattern for the major peak in peptide **10** (retention time = 22.7 min) shows unambiguous modification at the Cys residue without any apparent modifications at the Trp, His, or Tyr residues (**Figure SI-4**). All other impurities in the chromatogram for peptide C do not correspond with a mass consistent with aberrant arylation at other residues or desulfurization of the Cys.

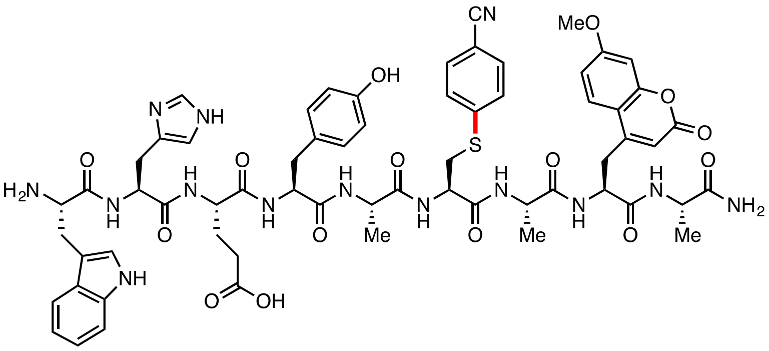

Peptide **10**

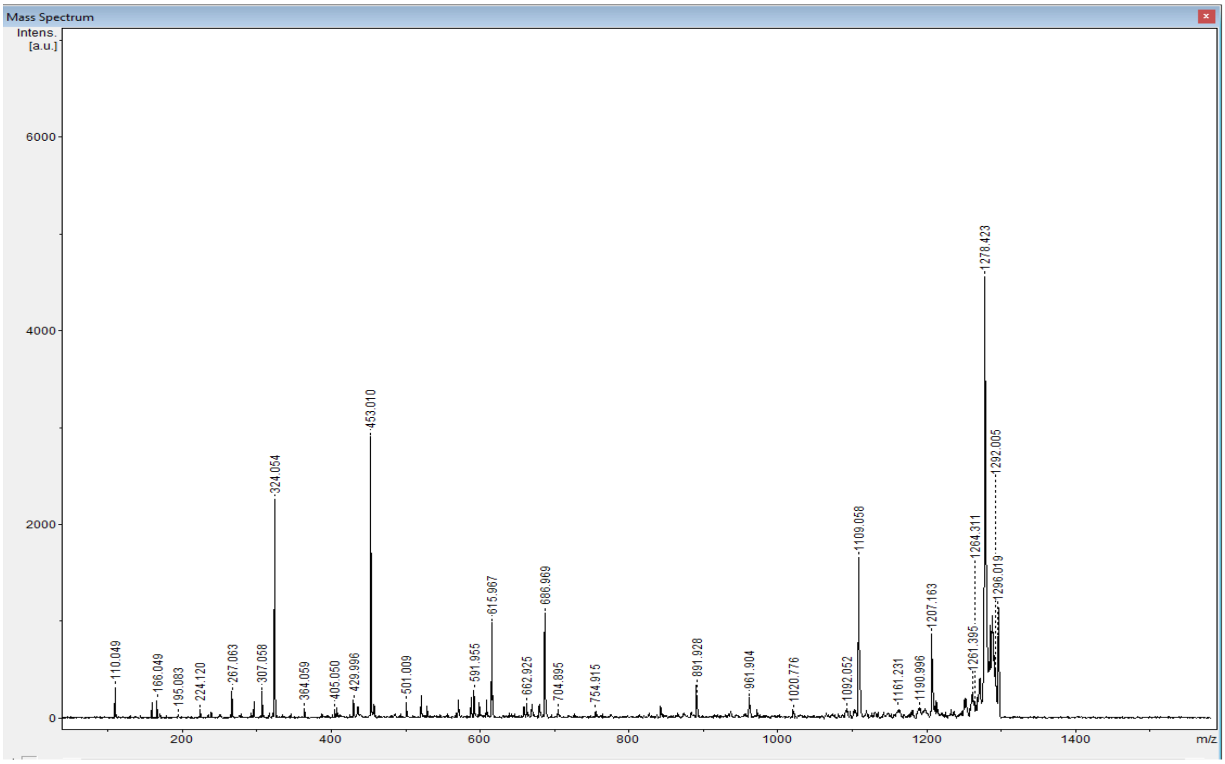

Figure SI-4. Tandem MS/MS fragmentation of peak corresponding to aryl sulfide peptide **10** (above) purified by analytical HPLC (expected $[M+H]^+$ = 1296.40 Da; observed 1296.01 Da). Y-axis = intensity; x-axis = m/z.

Sequence	Observed Series	a (m/z)	Observed a (m/z)	b (m/z)	Observed b (m/z)	x (m/z)	Obserevd x (m/z)	y (m/z)	Observed y (m/z)
W	a	159.210	159.093	187.221	---	---	---	1296.401	---
H	a,b,y	296.352	296.074	324.361	324.054	1136.182	---	1110.188	1109.058
E	b,y	425.467	---	453.477	453.010	999.041	---	973.047	971.940
Y	a,b	588.643	587.983	616.653	615.967	869.926	---	843.931	---
A	a,b,x,y	659.722	659.002	687.732	686.969	706.749	704.895	680.755	679.935
C^A	b,y	863.969	---	891.979	891.928	635.671	---	609.677	608.936
A	b,x,y	935.047	---	963.057	961.904	431.424	429.996	405.429	405.050
Mcm	b,x	1180.281	---	1208.291	1207.163	360.345	364.059	334.351	---
A	a,b	1250.376	1252.329	1278.386	1278.423	115.112	---	89.117	---

Table SI-3. MS/MS fragmentation observed mass list (a-, x-, b-, and y-series) from **Figure SI-4**

Enzyme CoA Data

A stock solution was prepared as described above in the general dilute reaction condition procedure, relative to 13 µmol (1 equiv) of the enzyme CoA substrate.

Reaction preparation and execution for Enzyme CoA Reactions (14-16):

Reactions with enzyme CoA (13 µmol, 1 equiv, 10 mg) and the various aryl bromides (20 equiv) were carried out in identical manner to that described previously under the dilute reaction conditions procedure, except 4 equiv of the diisopropylammonium bis(catechol)isobutyl silicate (52 µmol, 20.8 mg) were employed. Conversion to product was determined by UPLC-MS vs internal standard, until CoA starting material was consumed (90 min). Purification of the CoA adducts was attempted by reverse-phase prep LCMS in 0.1% TFA buffer (MeCN/H_2O); however, clean material for spectral data analysis could not be fully ascertained because of product instability. UPLC-MS and HRMS data was collected from the crude reaction mixture following the organic extraction protocol.

CoA aryl sulfide **14**

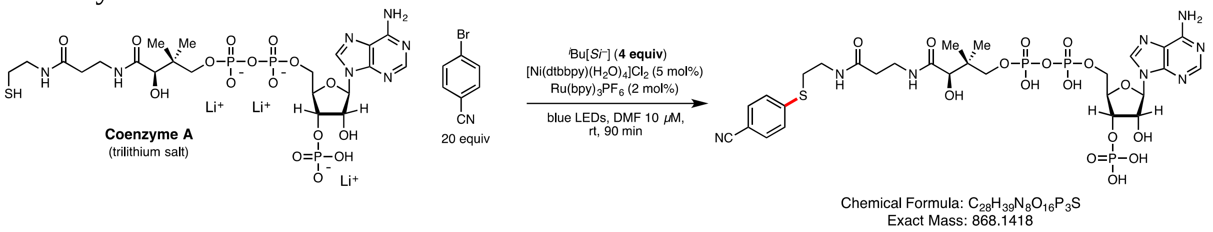

The LCMS trace for CoA **14** can be seen below. HRMS (ESI) m/z calc. for $C_{28}H_{40}N_8O_{16}P_3S$ $[M+H]^+$ 869.1496, found 869.1483.

Aqueous layer following the extraction protocol of CoA thioarylated adduct **14**. Reverse phase LCMS **Waters Sunfire C18 analytical column** 10 µm, 4.6 x 150 nm employed. Attempts to purify the thioarylated adduct under these conditions proved unsuccessful.

No.	Time (min)	MeCN (%)
1	0	98
2	2:00	95
3	5:00	85
4	10:00	60
5	15:00	5

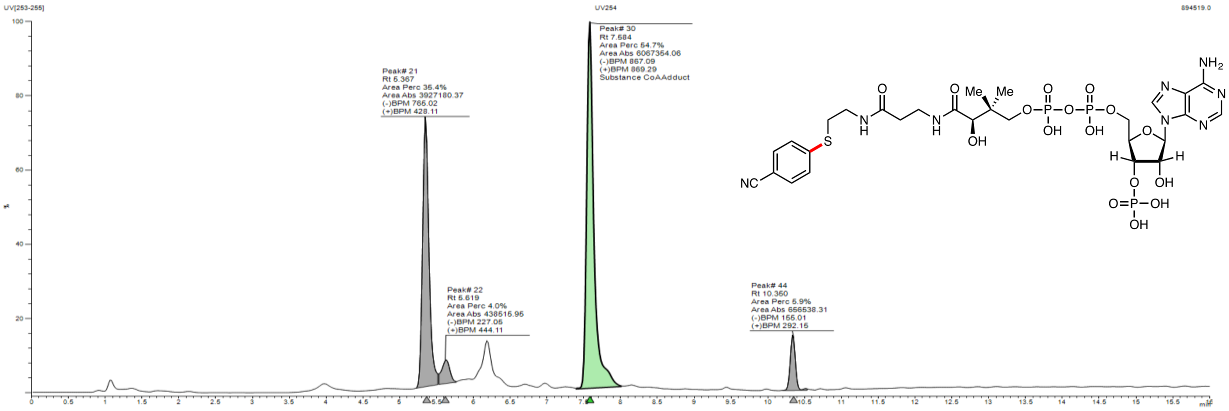

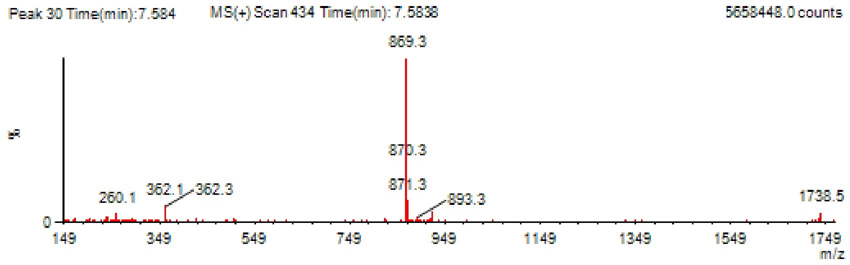

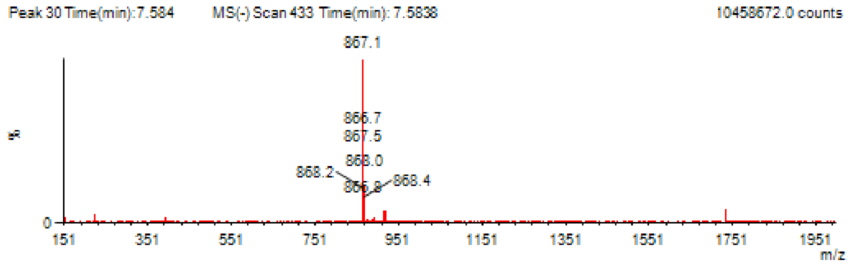

Aliquot from the <u>crude reaction mixture</u> of CoA thioarylated adduct **14** (in green) with excess ArBr present (in blue). Reverse phase LCMS **Waters Atlantis® column (polyamide)** analytical column (5 µm, 4.6 x 150 nm).

No.	Time (min)	MeCN (%)
1	0	98
2	2:00	95
3	5:00	80
4	10:00	60
5	15:00	5

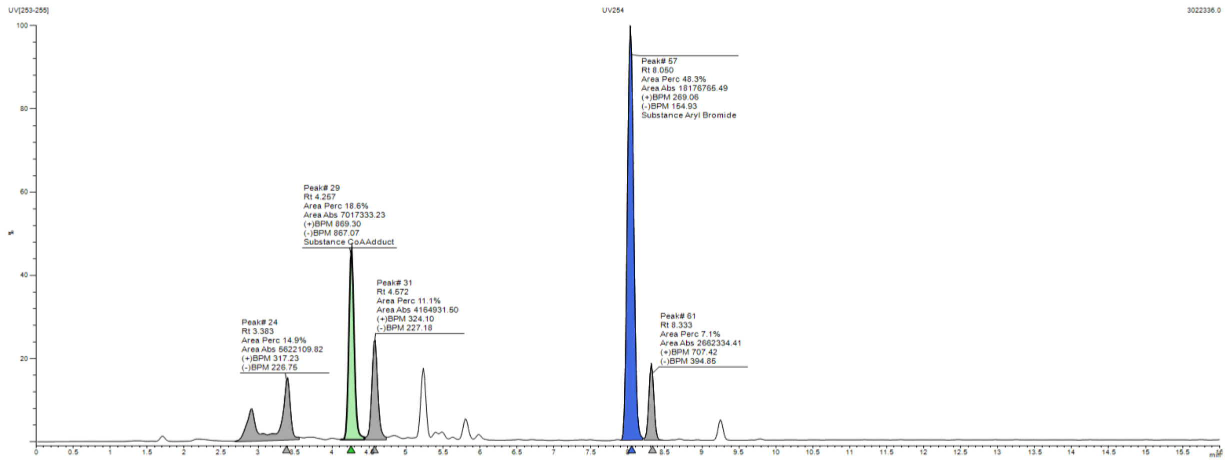

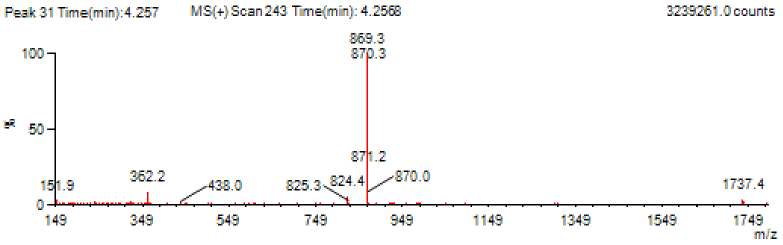

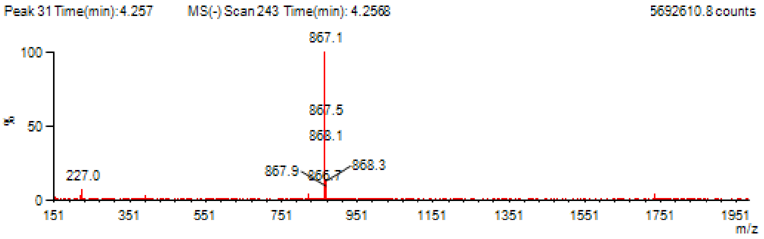

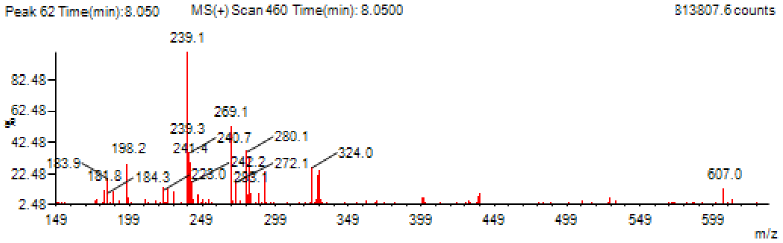

CoA aryl sulfide **15**

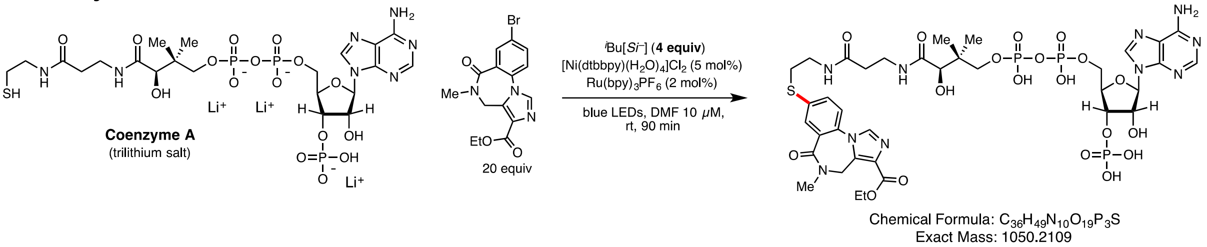

CoA thioarylated adduct **15**: LCMS was determined from the crude reaction mixture. The LCMS trace and mass chromatograms for this compound are below. LRMS m/z calc. for $C_{36}H_{50}N_{10}O_{19}P_3S$ $[M+H]^+$ 1051.2187, found 1050.2. Tentative structure assigned by analogy to aryl CoA **14**.

Aliquot from the crude reaction mixture of CoA thioarylated adduct **15** (in green) with remaining (excess) ArBr present (in blue) via UPLC-MS.

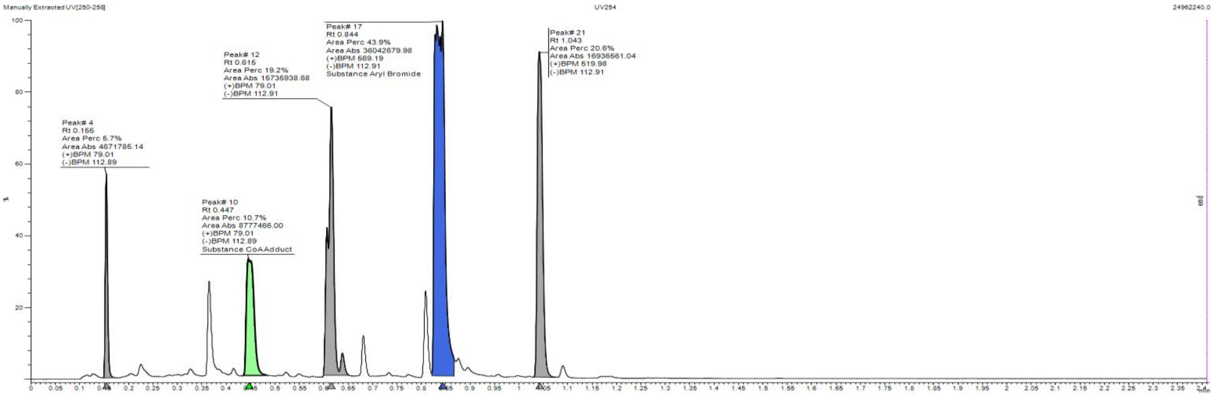

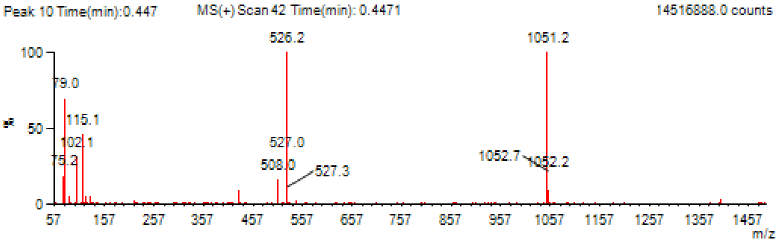

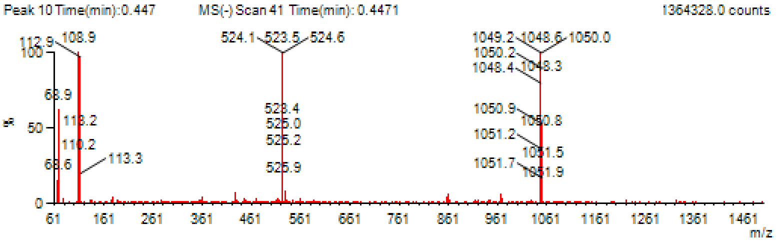

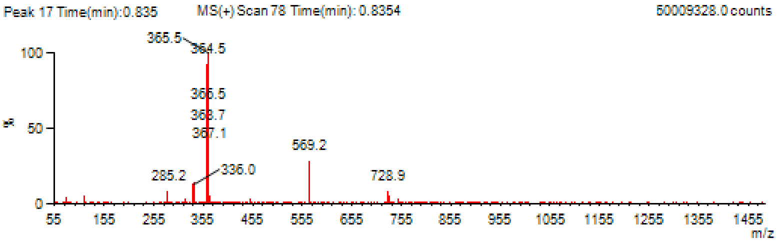

CoA aryl sulfide **16**

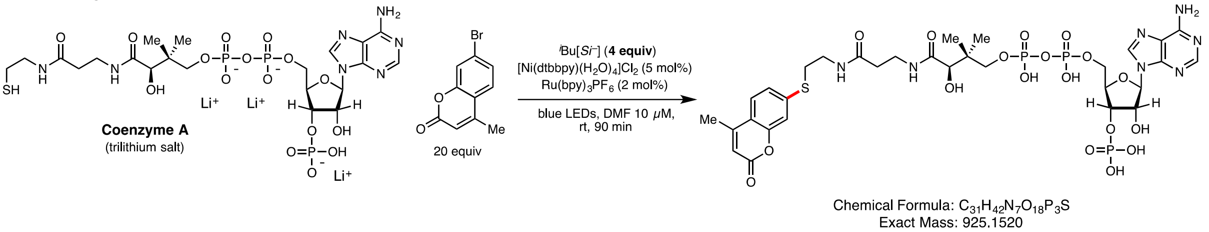

CoA thioarylated adduct **16**: LCMS was determined from the crude reaction mixture. The LCMS trace and mass chromatograms for this compound are below. LRMS m/z calc. for $C_{31}H_{43}N_7O_{18}P_3S$ $[M+H]^+$ 926.1598, found 926.0. Tentative structure assigned by analogy to aryl CoA **14**.

Aliquot from the crude reaction mixture of CoA thioarylated adduct **16** (in green) with excess ArBr present (in blue) via UPLC-MS.

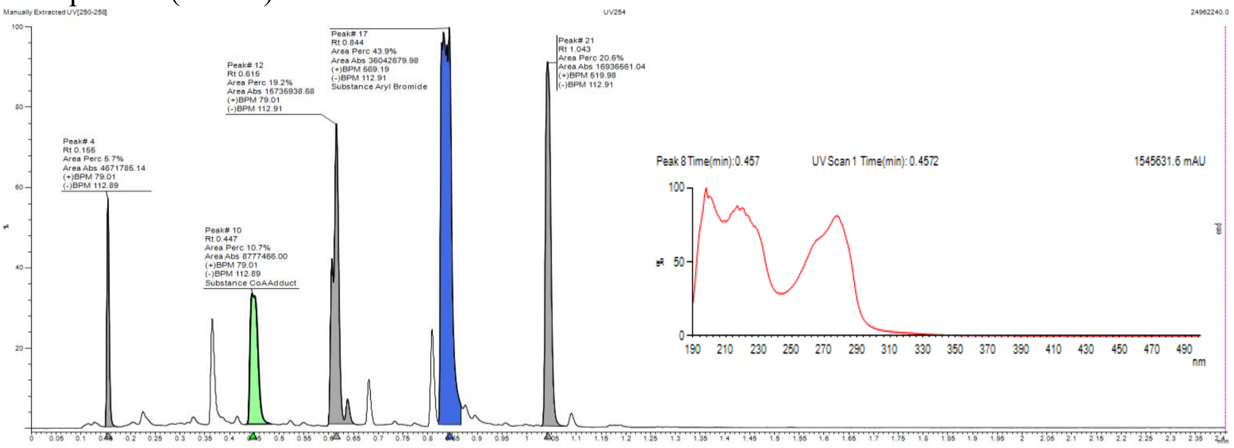

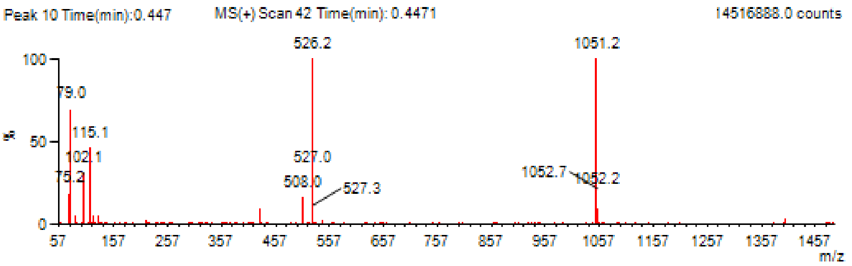

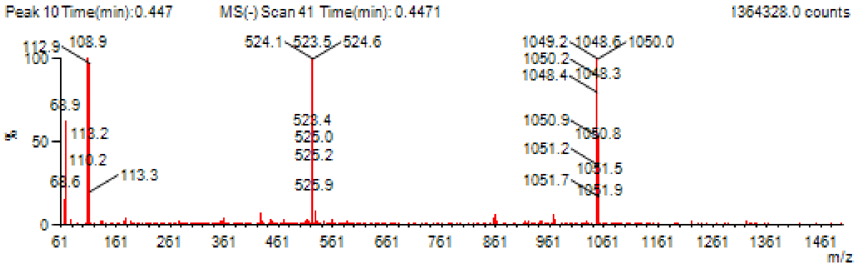

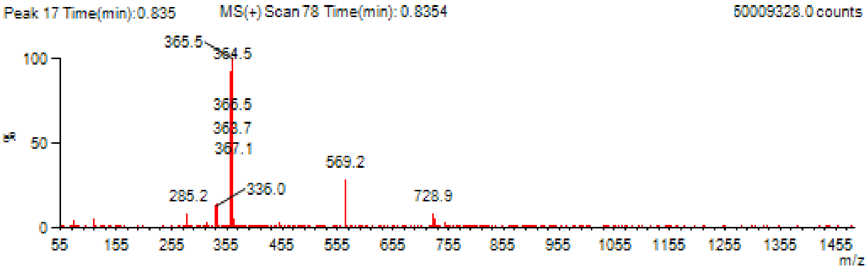

Cleavage/Arylation of GSH Disulfide (17)

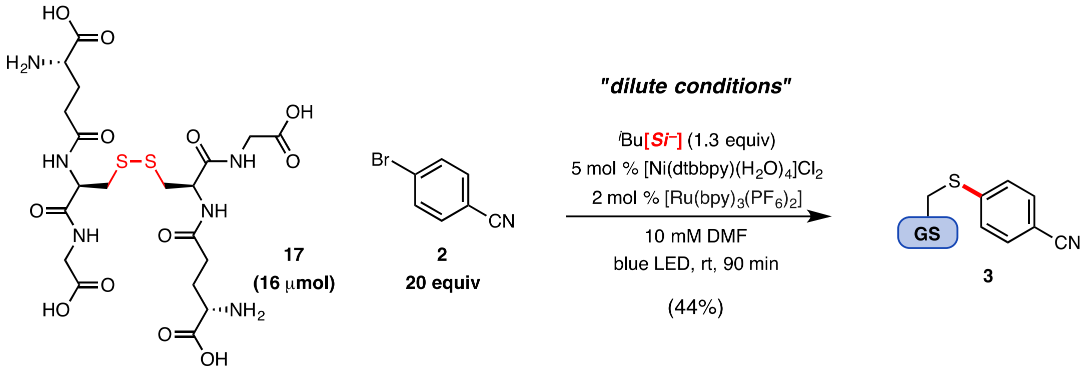

A stock solution was prepared as described above in the general dilute reaction condition procedure, relative to 16 µmol (0.5 equiv) of the disulfide peptide substrate **17***.*

Reaction preparation and execution for Enzyme CoA Reactions:
Reactions with GSH disulfide **17** (16 µmol, 0.5 equiv) and aryl bromide **2** (20 equiv) were carried out in an identical manner to that described previously under the dilute reaction conditions procedure. Conversion to product was monitored and determined by LCMS/HPLC vs internal standard (90 min). Starting disulfide could still be detected by LCMS. The crude reaction mixture was diluted with H_2O and extracted (CH_2Cl_2) according to the general extraction protocol. The peptide (aqueous layer) was purified via reverse phase LCMS, to afford the title compound **3** as the TFA salt in 44% yield.

Analytical data matched that of the previously characterized compound.

Supporting Data

Select Supplemental HPLC traces for arylated GSH adducts

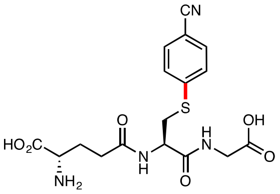

Chemical Formula: $C_{17}H_{20}N_4O_6S$
Molecular Weight: 408.4290

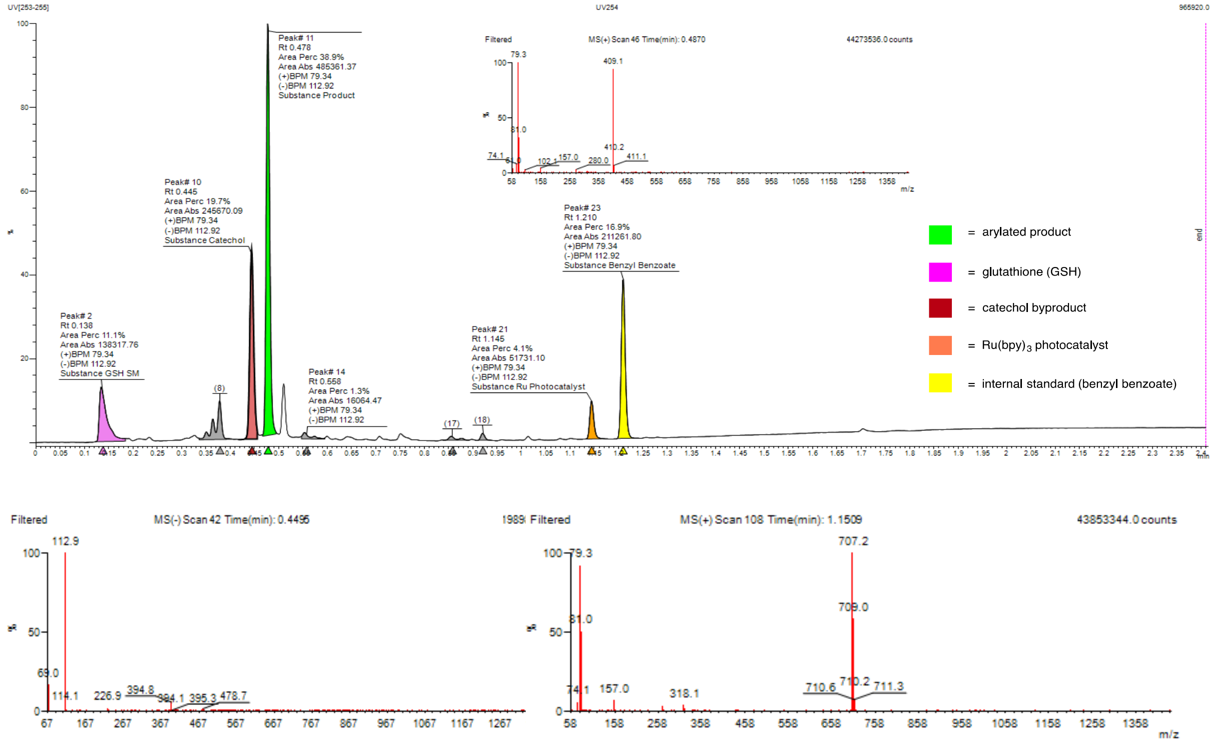

The above trace profiles the <u>crude</u> reaction mixture of the thioarylated GSH adduct **3**. The color-coded peaks designate product and byproducts including catalyst, ligands, and starting material (GSH). Mass chromatograms are included to verify arylated peptide (green, 0.48 min), catechol (red, 0.45 min), and photocatalyst (orange, 1.15 min), and to assist in the user experience.

The traces below are aliquots from select reactions of the final aq layers following organic extraction with DCM (upon reaction completion) and before reverse-phase prep-LCMS. Unreacted aryl bromide and GSH can be observed in some cases, but traces are otherwise clean following the extraction protocol.

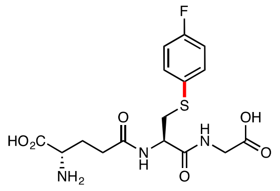

Chemical Formula: $C_{16}H_{20}FN_3O_6S$
Molecular Weight: 401.4094

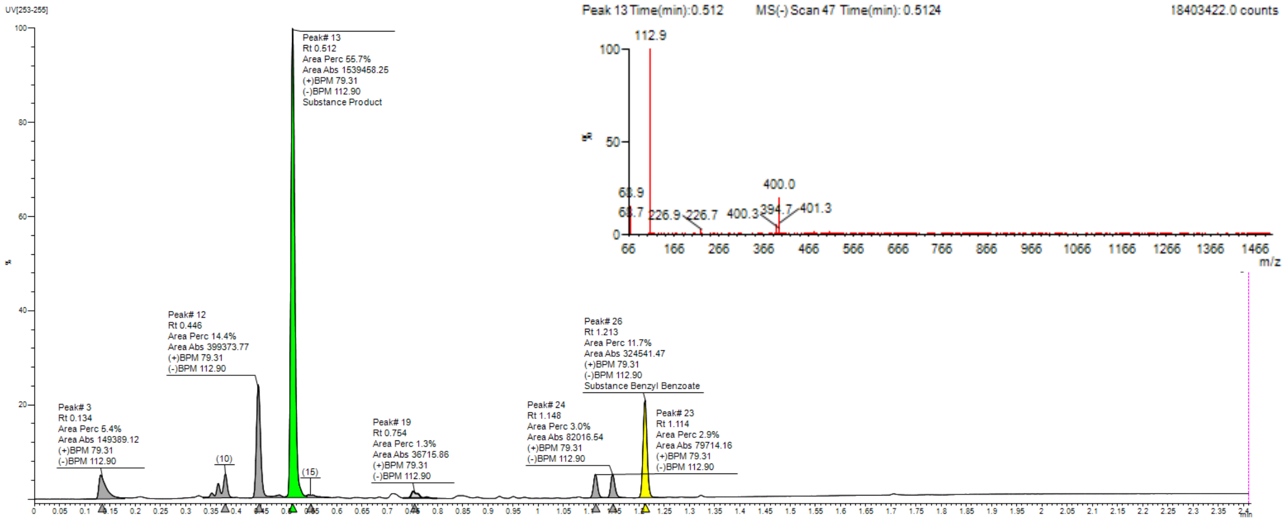

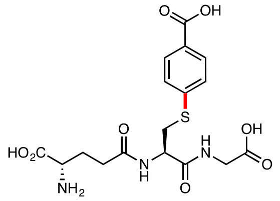

Chemical Formula: $C_{17}H_{21}N_3O_8S$
Molecular Weight: 427.4280

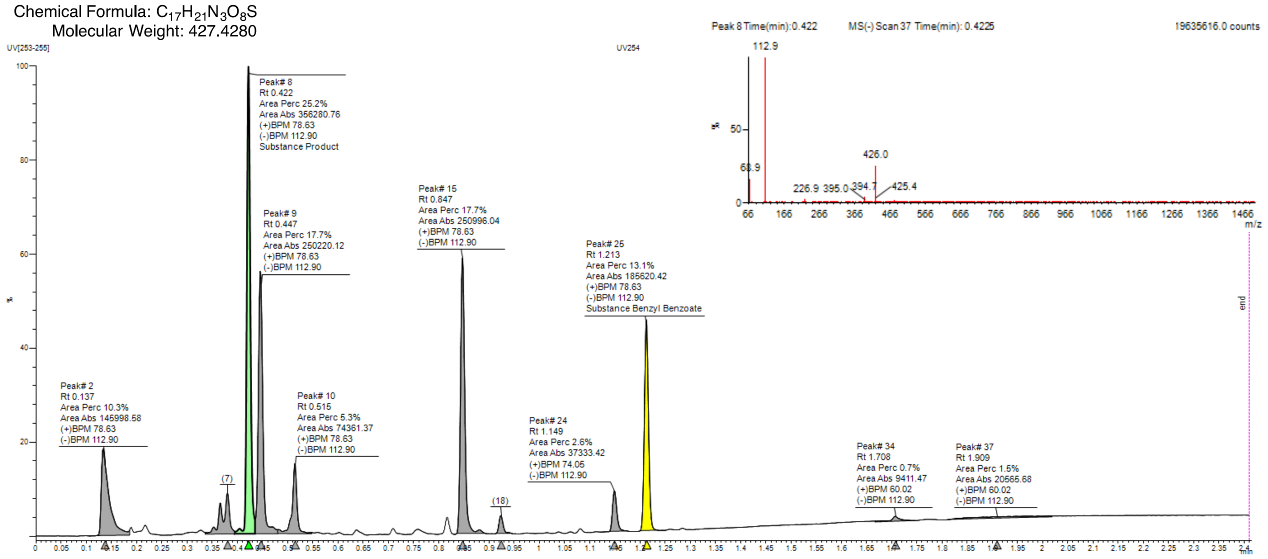

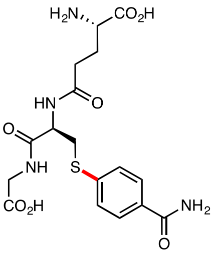

Chemical Formula: $C_{17}H_{22}N_4O_7S$
Molecular Weight: 426.4440

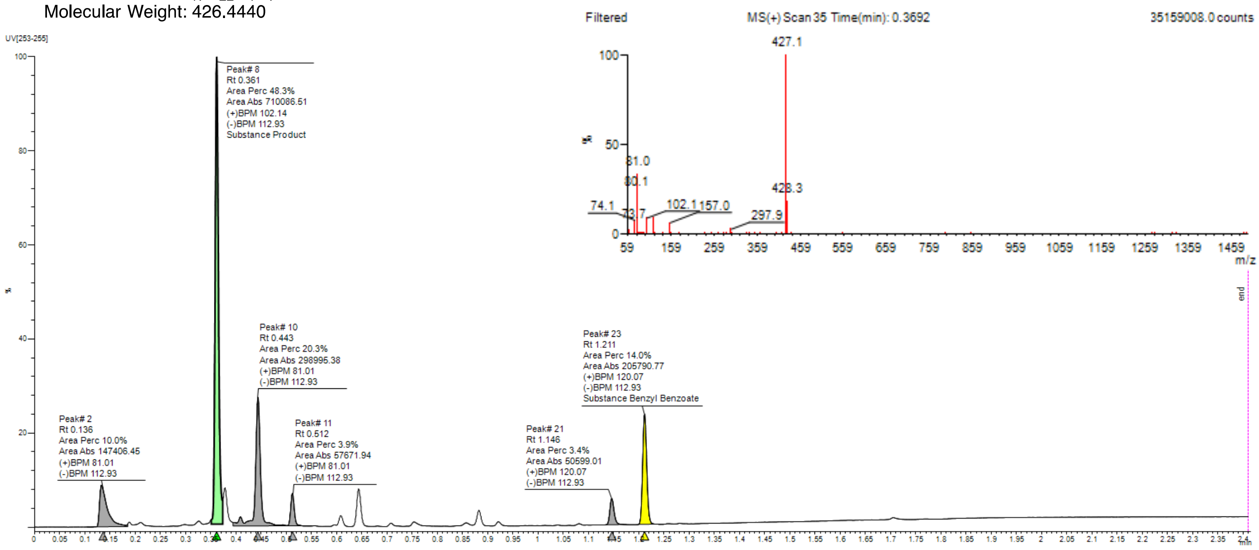

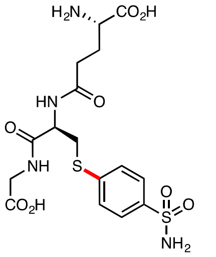

Chemical Formula:
$C_{16}H_{22}N_4O_8S_2$
Molecular Weight: 462.4920

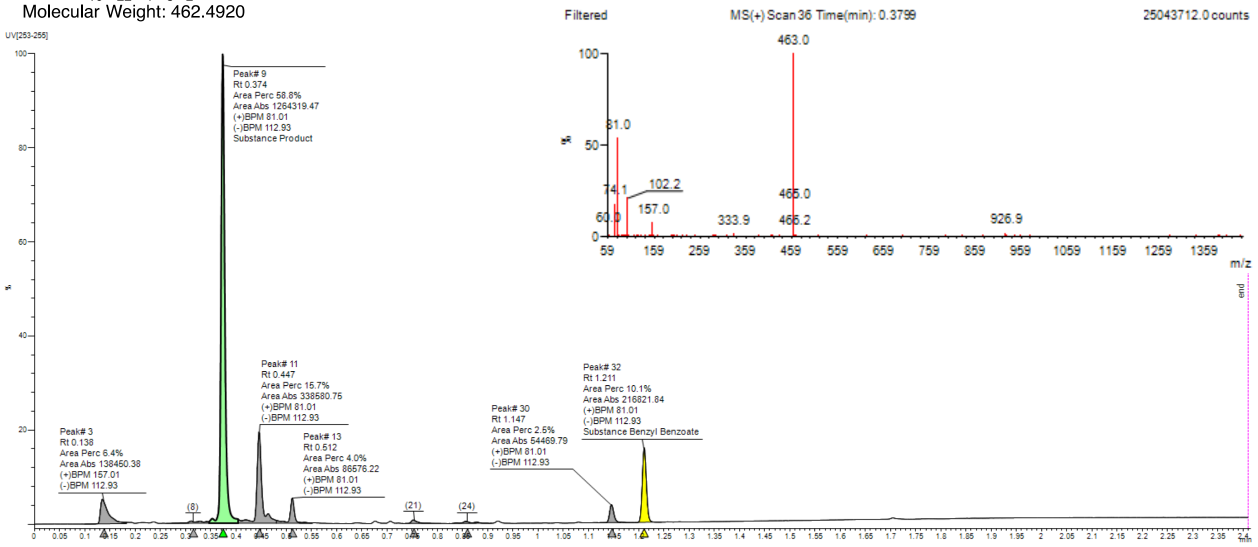

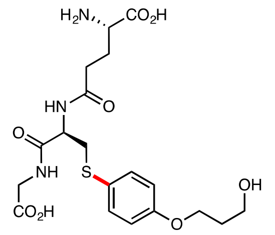

Chemical Formula: $C_{19}H_{27}N_3O_8S$
Molecular Weight: 457.4980

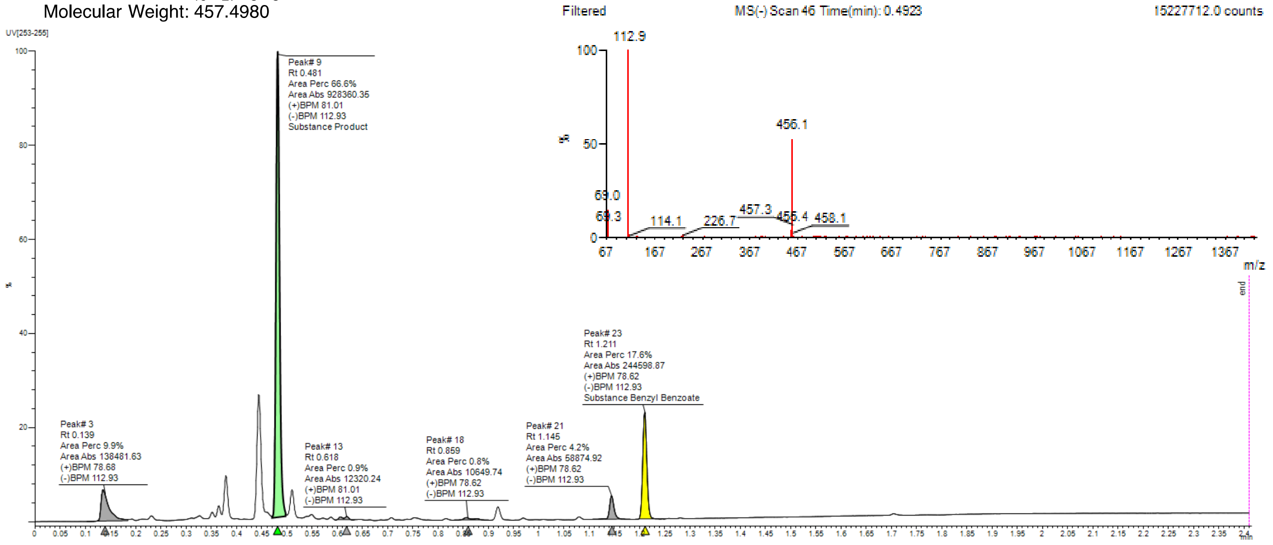

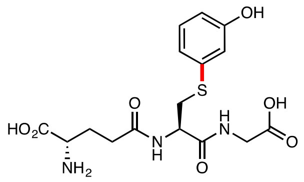

Chemical Formula: $C_{16}H_{21}N_3O_7S$
Molecular Weight: 399.4180

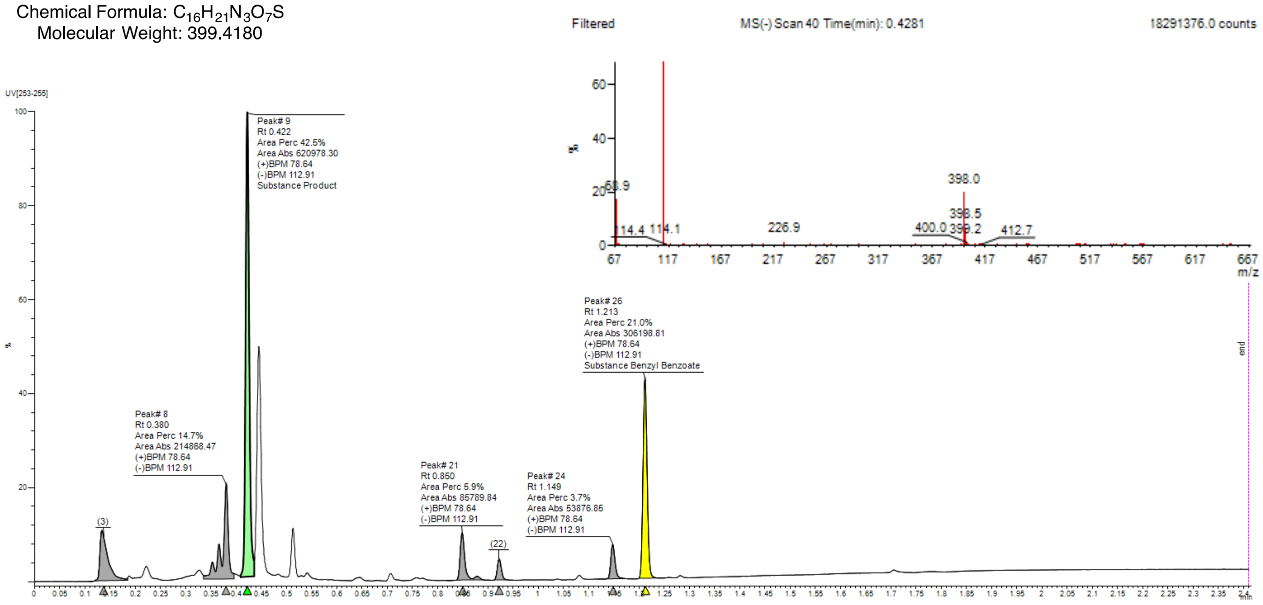

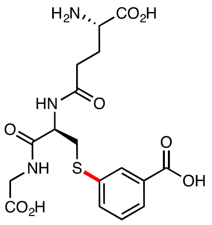

Chemical Formula: $C_{17}H_{21}N_3O_8S$
Molecular Weight: 427.4280

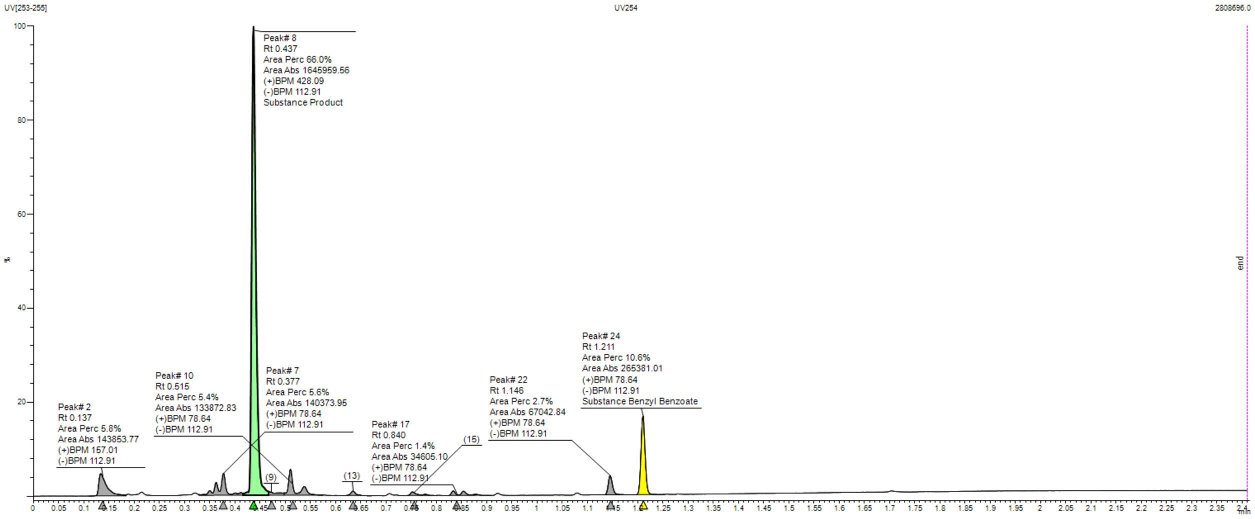

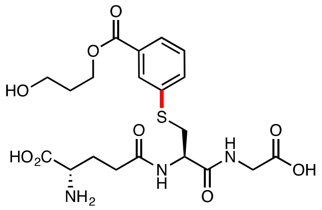

Chemical Formula: $C_{20}H_{27}N_3O_9S$
Molecular Weight: 485.5080

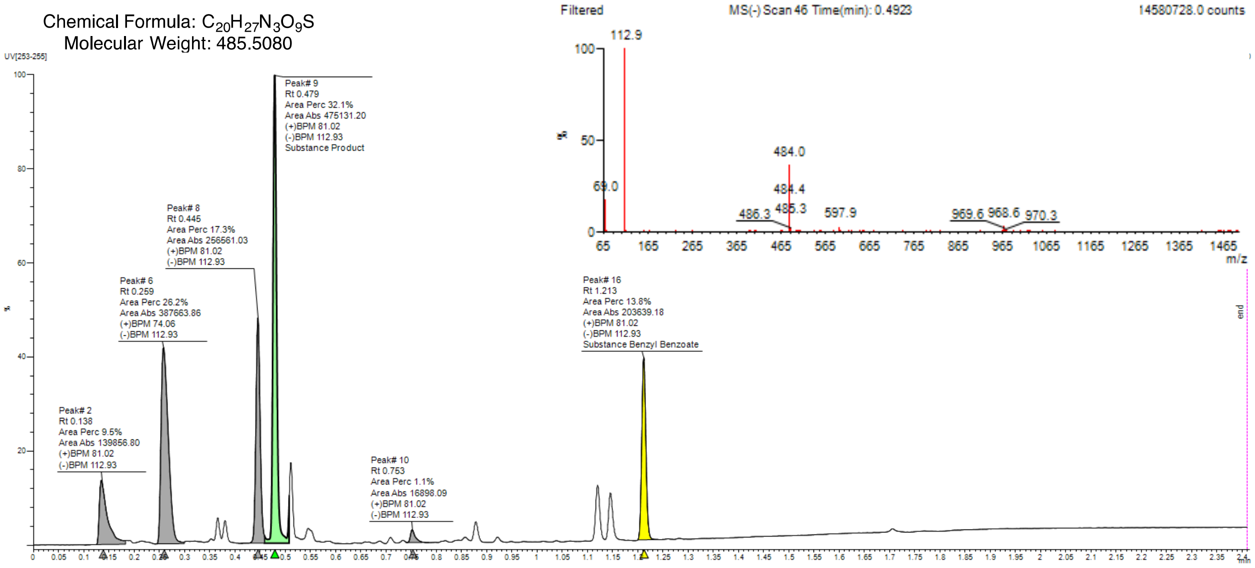

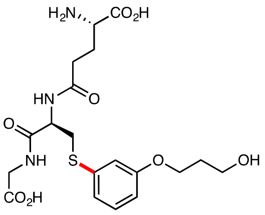

Chemical Formula: $C_{19}H_{27}N_3O_8S$
Molecular Weight: 457.4980

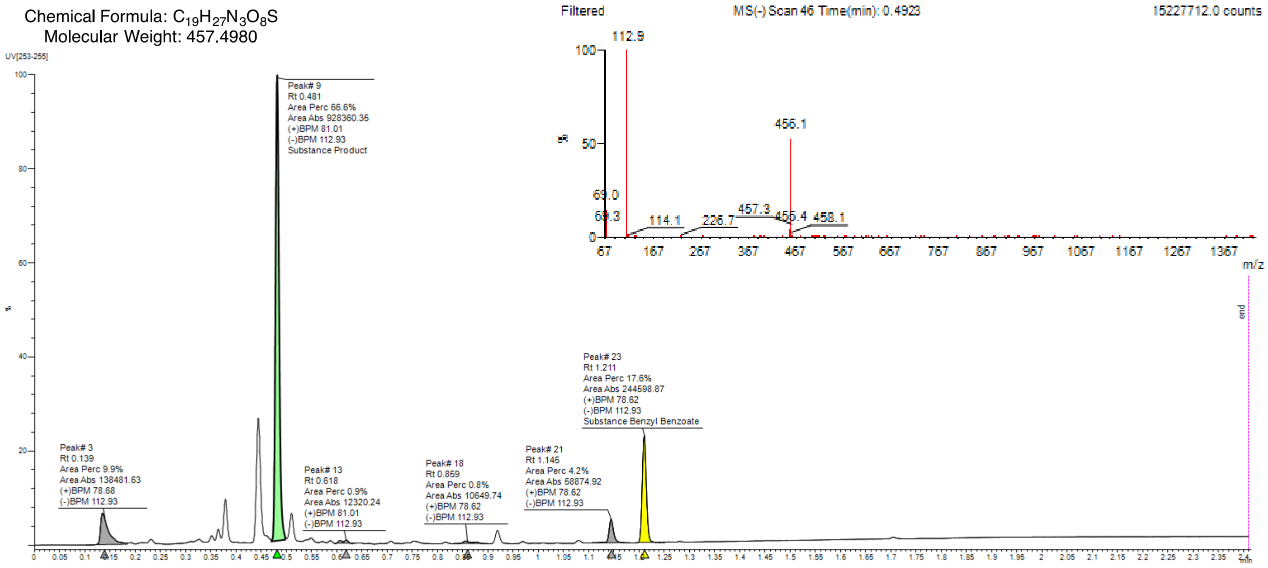

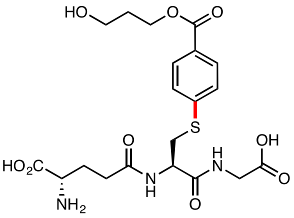

Chemical Formula: $C_{20}H_{27}N_3O_9S$
Molecular Weight: 485.5080

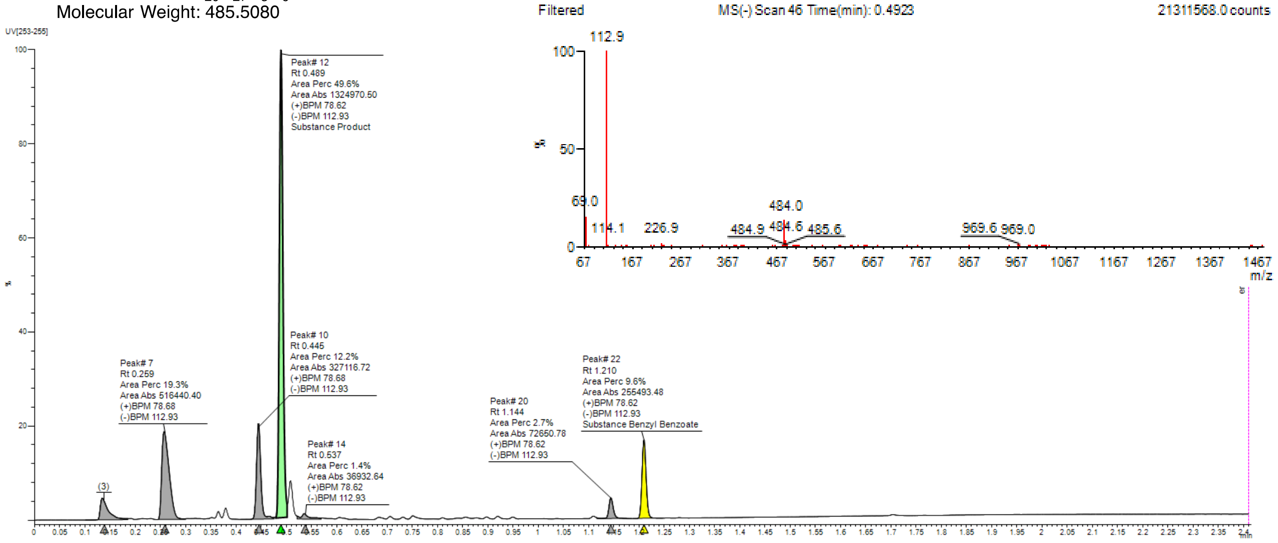

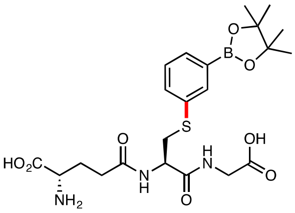

Chemical Formula: $C_{22}H_{32}BN_3O_8S$
Molecular Weight: 509.3810

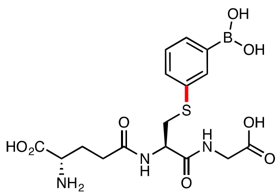

Chemical Formula: $C_{16}H_{22}BN_3O_8S$
Molecular Weight: 427.2350

(BPin adduct AND the hydrolyzed boronic acid (green) both detected prior to the post reaction addition of 5 equiv of TFA)

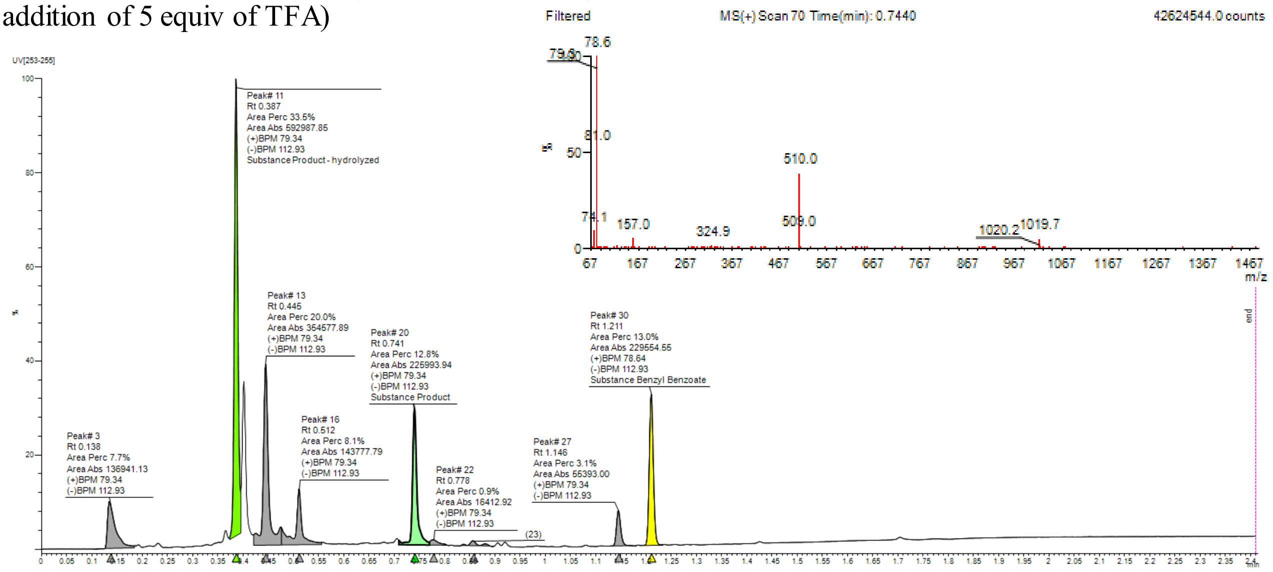

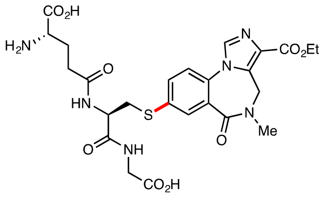

Chemical Formula: $C_{25}H_{30}N_6O_9S$
Molecular Weight: 590.6080

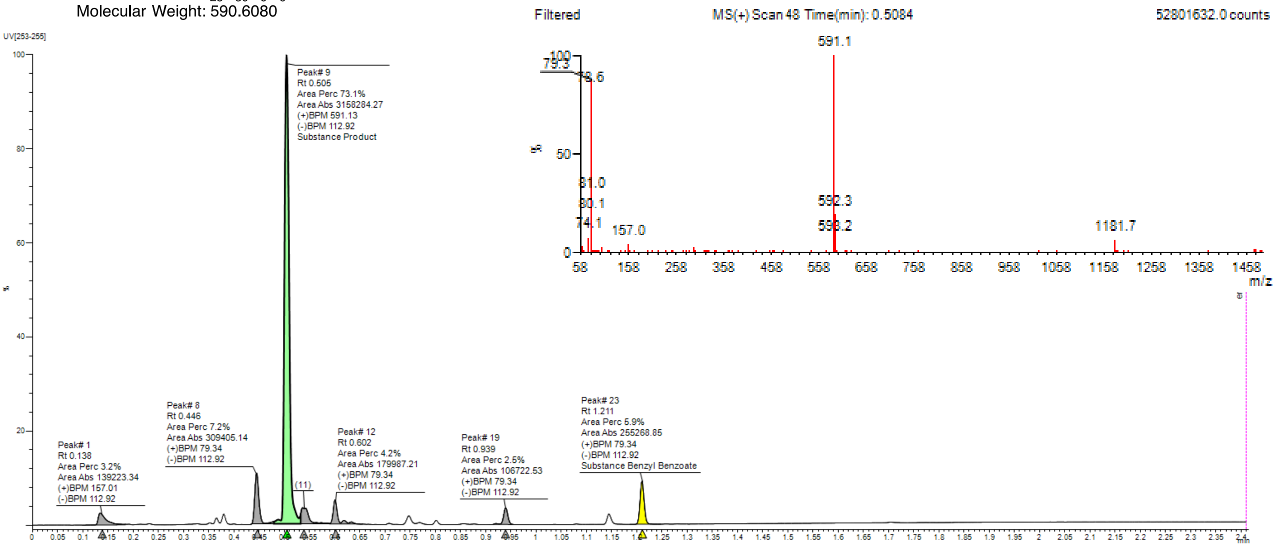

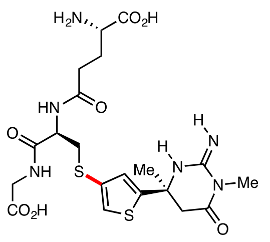

Chemical Formula: $C_{20}H_{28}N_6O_7S_2$
Molecular Weight: 528.5990

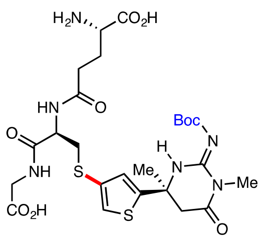

Chemical Formula: $C_{25}H_{36}N_6O_9S_2$
Molecular Weight: 628.7160

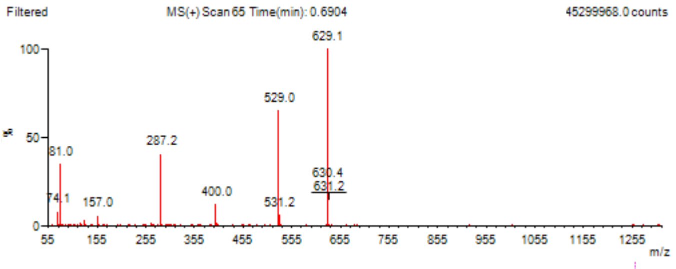

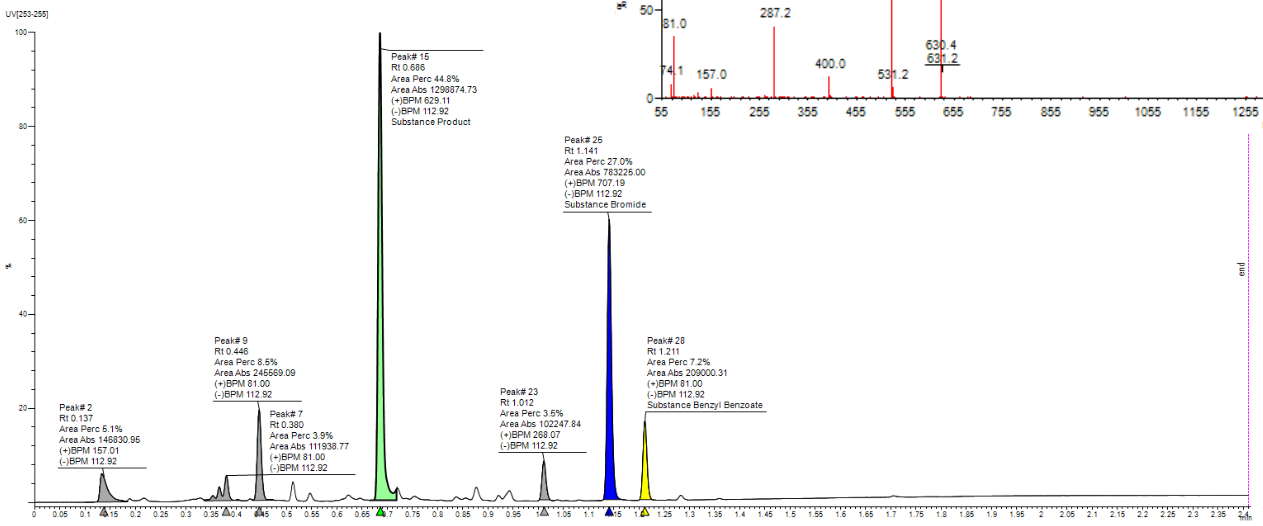

(Boc group removed upon purification and concentration with TFA)

Synthesis of Cys Containing Polypeptide 9

Solid phase peptide synthesis (SPPS) of peptide **9** was performed on Rink amide resin (100-200 mesh, 50 μmol scale) in a 10 mL reaction vessel. The resin was swelled in DMF (4 mL, 30 min) while stirring. The initial fluorenylmethyloxycarbonyl (Fmoc) deprotection was performed by treating the resin with a 4 mL of a solution of 20 % v/v piperidine in DMF for 20 min at rt. The vessel was then drained, and the resin was washed with DMF (2 x 2 mL), CH_2Cl_2 (2 x 2 mL), and DMF again (1 x 4 mL). Fmoc-Ala-OH (5 equiv) and HBTU (5 equiv) were dissolved in DMF (2 mL), and DIPEA (10 equiv) was added to activate the amino acid for coupling. This solution was added to the resin and allowed to stir for 1 h at rt. After completion of the coupling reaction, the vessel was drained and the resin was washed with DMF (2 x 2 mL), CH_2Cl_2 (2 x 2 mL), and DMF again (1 x 4 mL). Each subsequent Fmoc deprotection and coupling was performed using the above steps, and the appropriate Fmoc protected amino acid, with the exception of Fmoc-β-(7-methoxy-coumarin-4-yl)-alanine (Fmoc-Mcm-OH), in which fewer equivalents of amino acid were used to conserve material (3 equiv of Fmoc-Mcm-OH and HBTU; 4 equivalents of DIPEA). After the last Fmoc group was removed, the resin was washed with DMF, followed by CH_2Cl_2 (2 x 4 mL each). The resin was then dried for 30 min under vacuum. A cleavage cocktail (2 mL total volume) was prepared with 90:5:2.5:2.5 TFA:TIPS:EDT:thioanisole. This was added to the resin, and the mixture was allowed to stir at rt for 30 min before being drained and collected. CH_2Cl_2 (3 mL) was added to the resin to wash out any remaining peptide, and the collected solution was dried under rotary evaporation. The crude peptide product was then precipitated by adding cold Et_2O (15 mL) and vortexing vigorously. The precipitate was pelleted by centrifugation, and the Et_2O supernatant was discarded. The resulting precipitate was dried and stored under argon at –20 °C until HPLC purification could proceed.

Preparative HPLC of Peptide 9

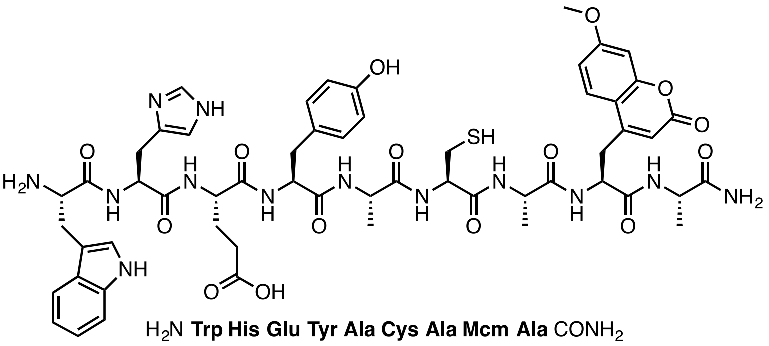

H_2N **Trp His Glu Tyr Ala Cys Ala Mcm Ala** $CONH_2$

Chemical Formula: $C_{56}H_{67}N_{13}O_{15}S$
Molecular Weight: 1194.2880

The crude precipitate from the above procedure was dissolved in 40 % v/v MeCN in H_2O with 0.1 % TFA (8 mL) and treated with 5 μL of TCEP Bond Breaker™ to reduce any disulfide formation at the Cys residues. Purification was performed by reverse phase HPLC on a Grace Vydac C18 Preparatory column using the gradient **2** (peptide **9**) described in **Table SI-4** (12.0 mL/min flow rate). Fractions were collected, and the product peak was identified by ESI-LRMS. The collected fractions were frozen in liquid N_2 and lyophilized to a white powder. The resulting solid was redissolved in 40 % v/v MeCN/ H_2O with 0.1 % TFA (4 mL) and treated with 5 μL of TCEP Bond Breaker™. Peptide **9** was subjected to a second pass of reverse phase HPLC purification using gradient **4** listed in **Table SI-4** The collected fractions were frozen in liquid N_2 and lyophilized to a white powder. Isolated yields were

calculated by measuring the absorption at 325 nm of the Mcm residue (ε_{325} = 11, 820 M^{-1} cm^{-1}) in a diluted sample. These samples were prepared by dissolving the peptides in 3 mL of 10% v/v MeCN in H_2O and diluting an aliquot of the solution 1:25 in H_2O (130 µL final volume) before measuring the absorbance at 325 nm. Isolated percent yields was 19.4 % (9.7 µmol) for peptide **9**, respectively. Calculated m/z for peptide **9** ($C_{56}H_{68}N_{13}O_{15}$) $[M+H]^+$ is 1195.3, found 1195.0)

No.	Time (min)	Buffer A (%)	No.	Time (min)	Buffer A (%)	No.	Time (min)	Buffer A (%)
**1**	0	98	**2**	0	98	**3**	0	98
	5:00	98		5:00	98		5:00	98
	10:00	75		10:00	68		10:00	75
	30:00	50		30:00	60		30:00	60
	32:00	0		32:00	0		32:00	0
	36:00	0		36:00	0		36:00	0
	38:00	98		38:00	98		38:00	98
	42:00	98		42:00	98		42:00	98
**4**	0	98	**5**	0	98	**6**	0	98
	5:00	98		10:00	98		10:00	98
	10:00	68		15:00	75		15:00	69
	20:00	64		35:00	60		35:00	65
	22:00	0		37:00	2		37:00	2
	26:00	0		41:00	2		41:00	2
	28:00	98		43:00	98		43:00	98
	32:00	98		47:00	98		47:00	98

Table SI- 4. Preparatory and analytical HPLC gradients. Buffer A: Milli-Q H_2O with 0.1 % TFA. Buffer B: MeCN with 0.1 % TFA

NMR Spectral Data

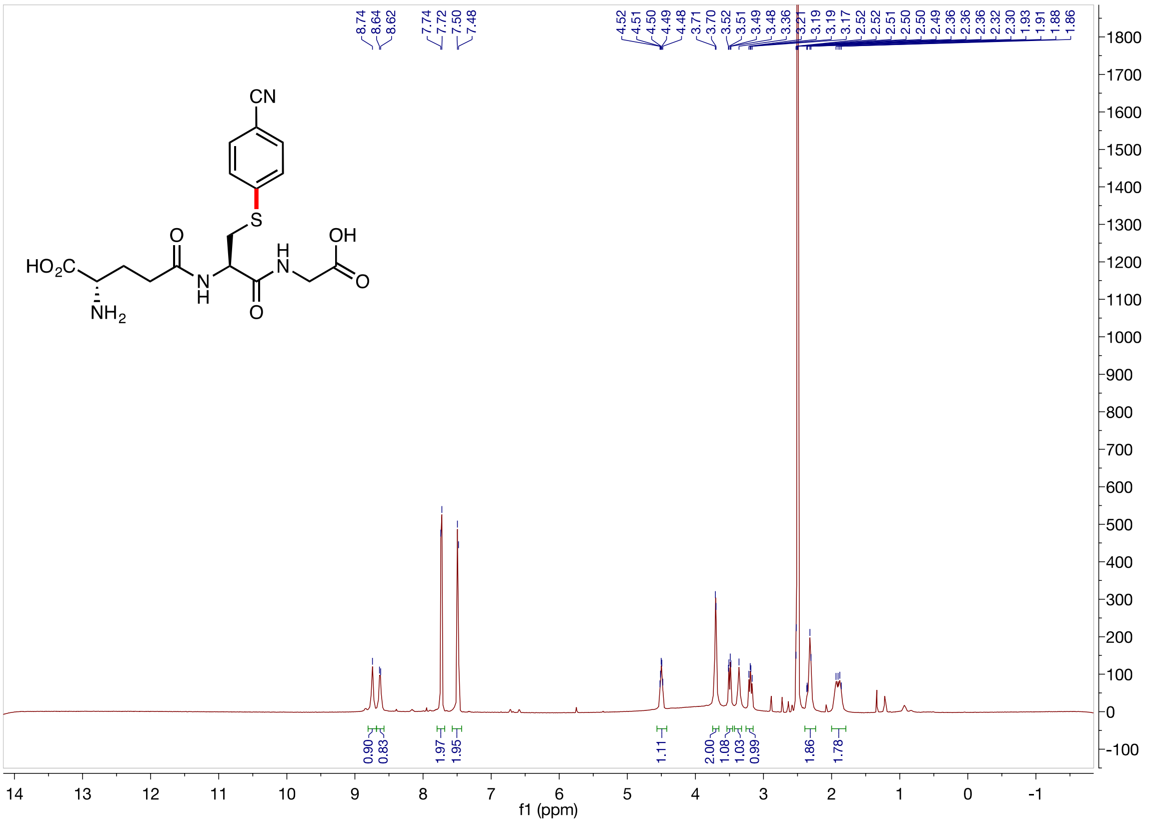

^{1}H NMR (DMSO-d_6, 500.4 MHz) of (**3**)

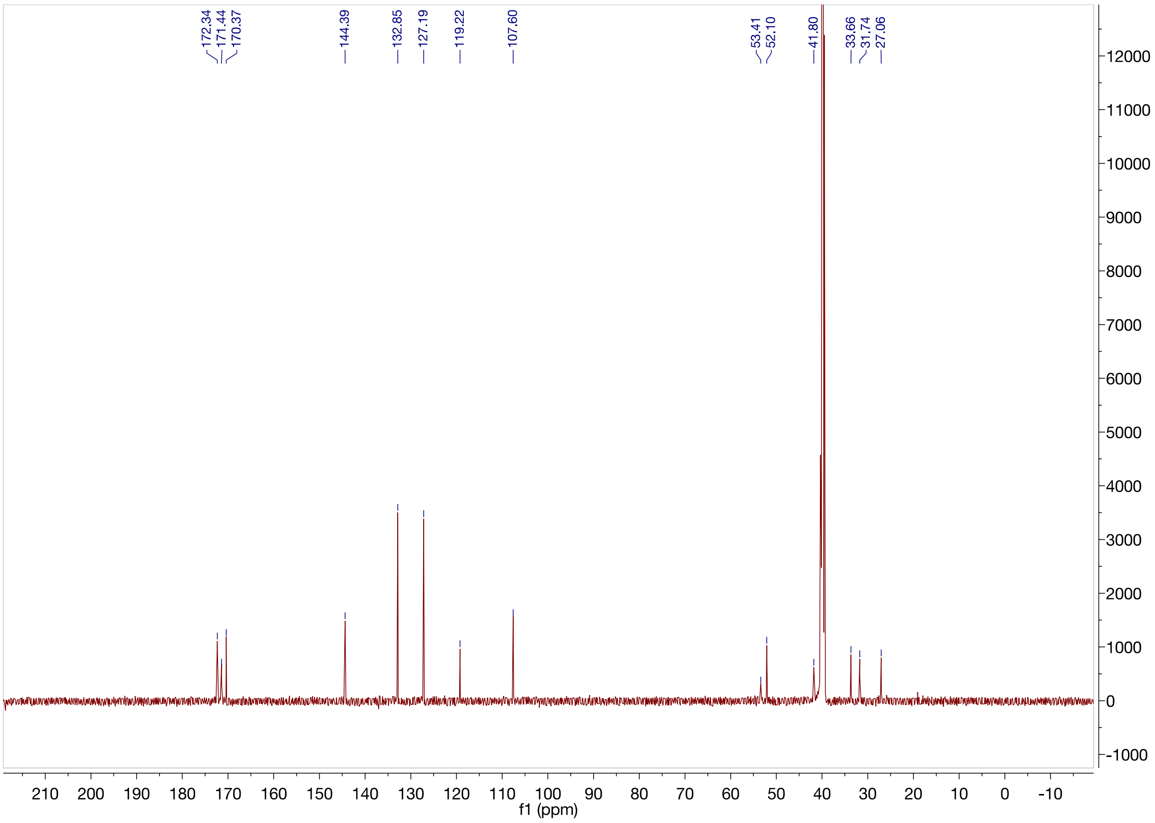

^{13}C {^{1}H} NMR (DMSO-d_6, 125.8 MHz) of (**3**)

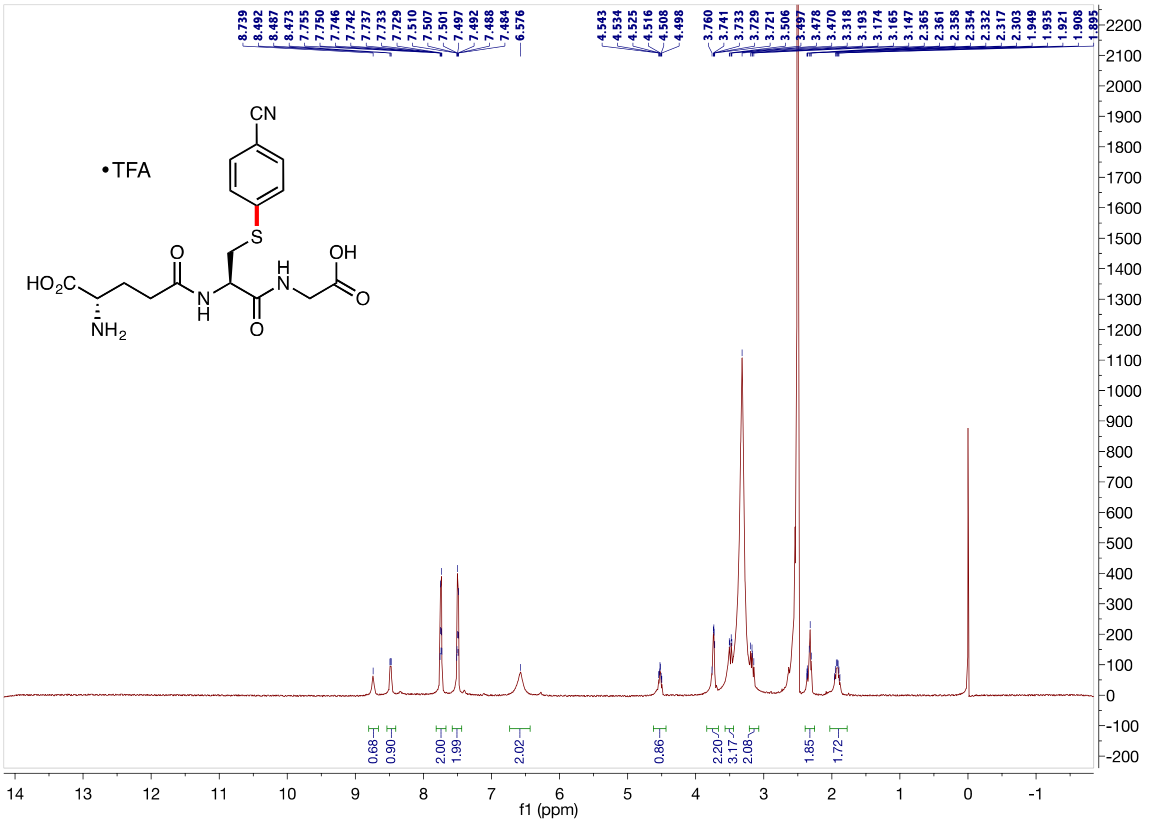

^{1}H NMR (DMSO-d_6, 500.4 MHz) of (**3**)

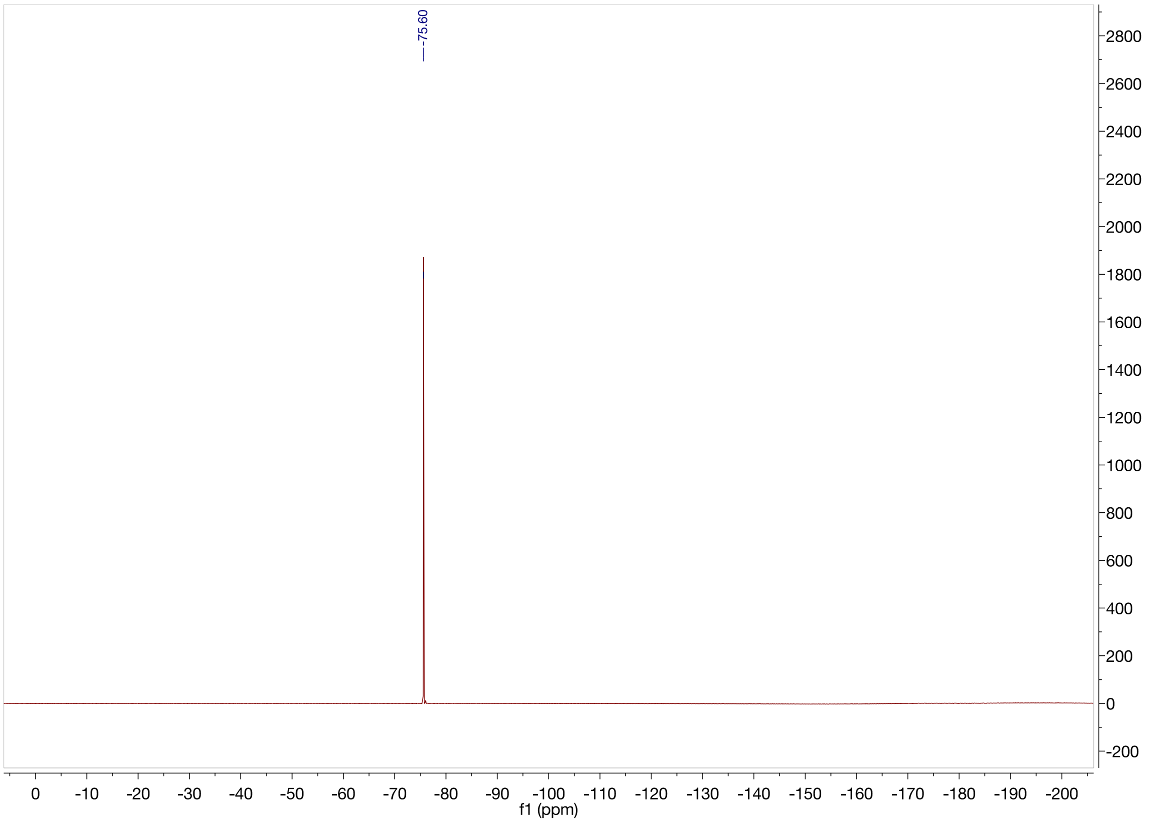

^{19}F {^{1}H} NMR (DMSO-d_6, 470.8 MHz) of (**3**)

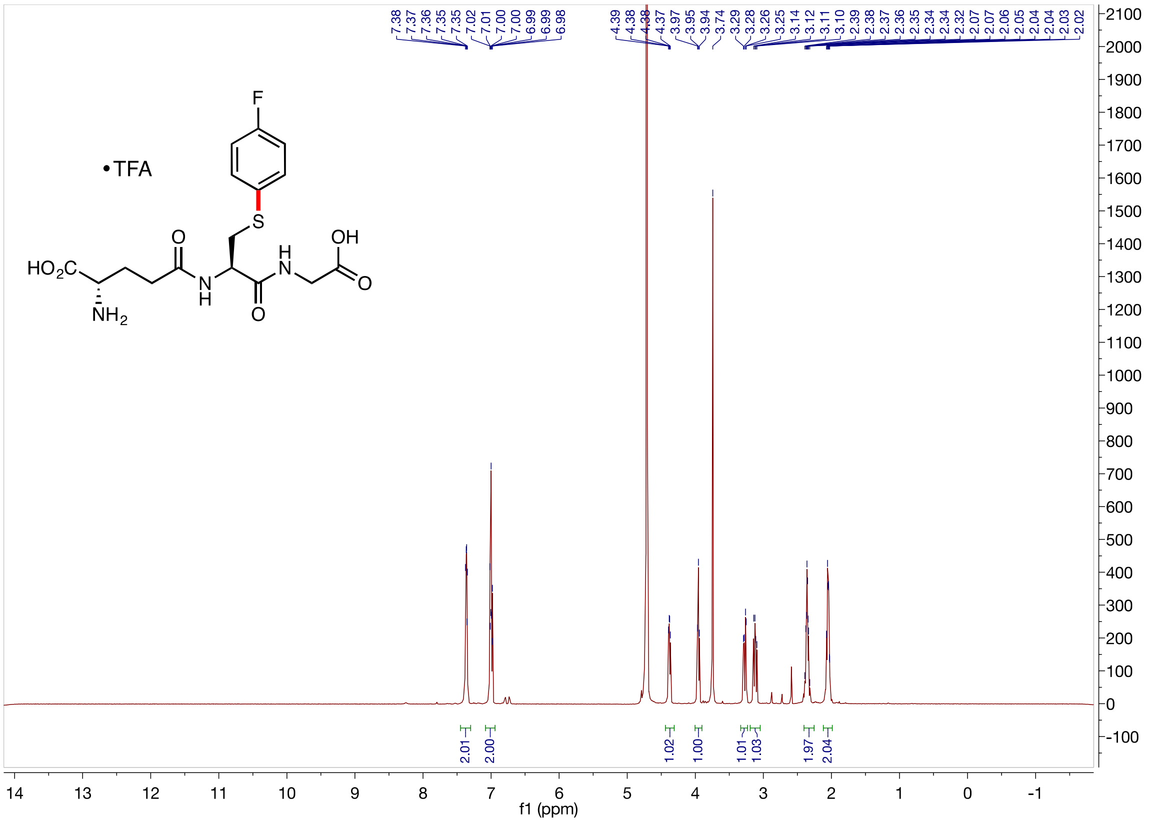

^{1}H NMR (D_2O, 500.4 MHz) of (**1a**)

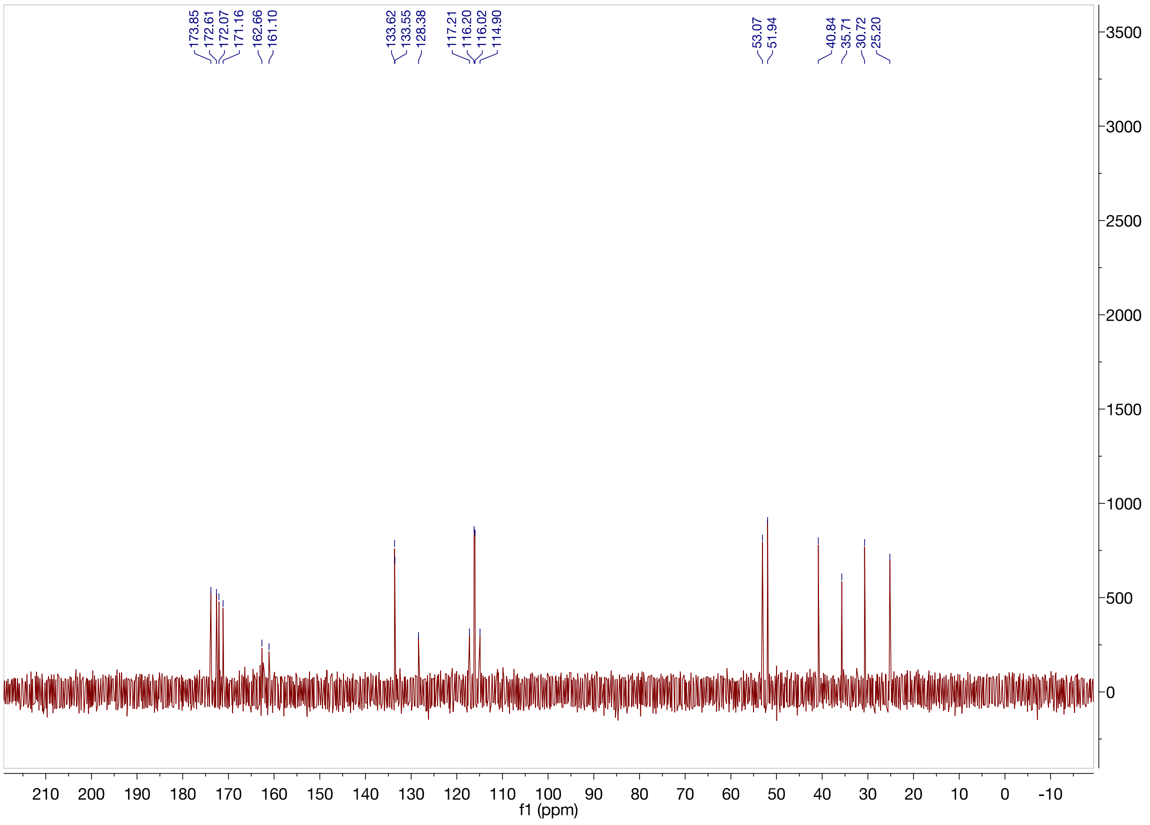

^{13}C {^{1}H} NMR (D_2O, 125.8 MHz) of (**1a**)

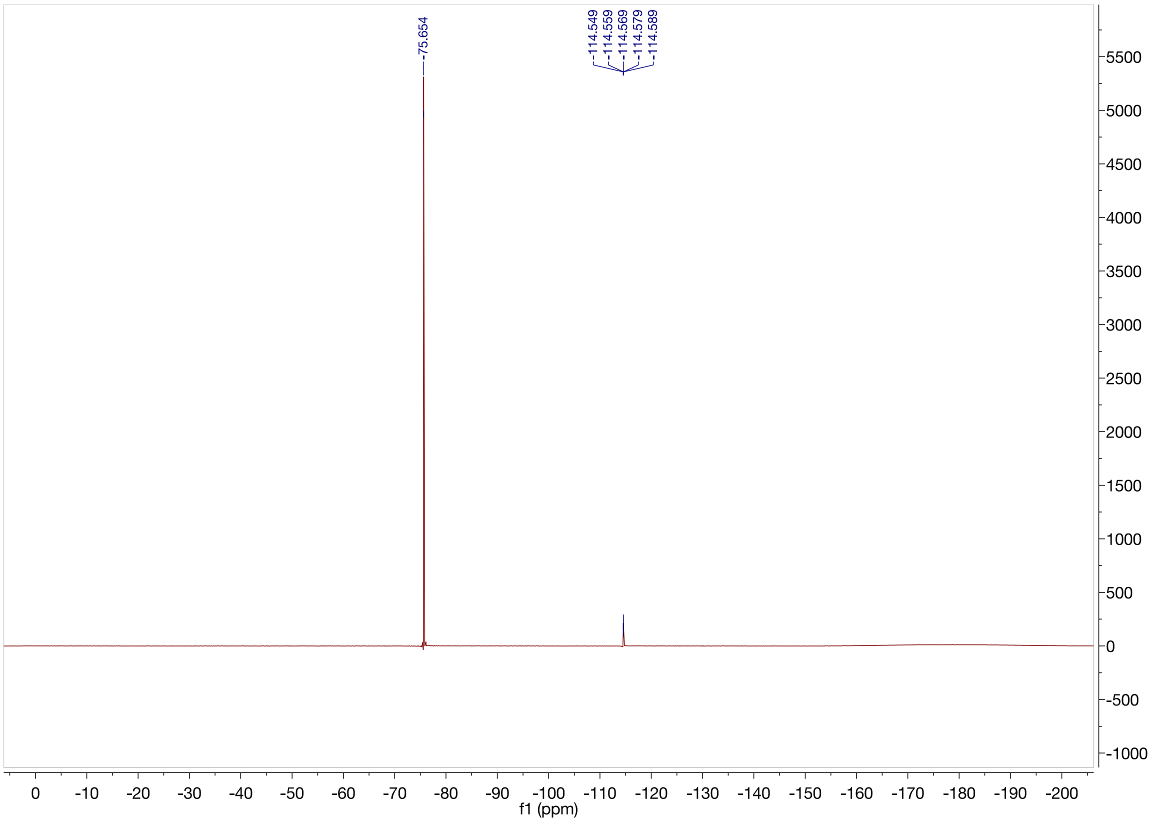

^{19}F {^{1}H} NMR (D_2O, 470.8 MHz) of (**1a**)

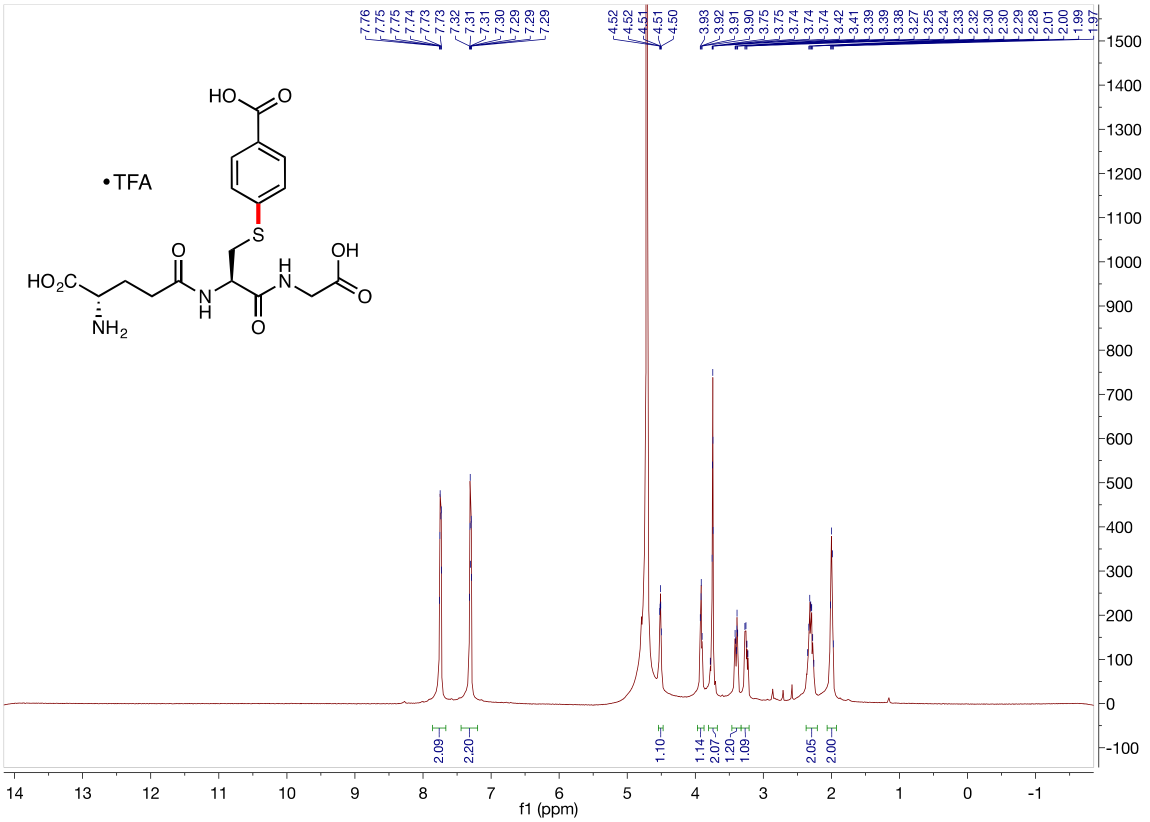

^{1}H NMR (D_2O, 500.4 MHz) of (**1b**)

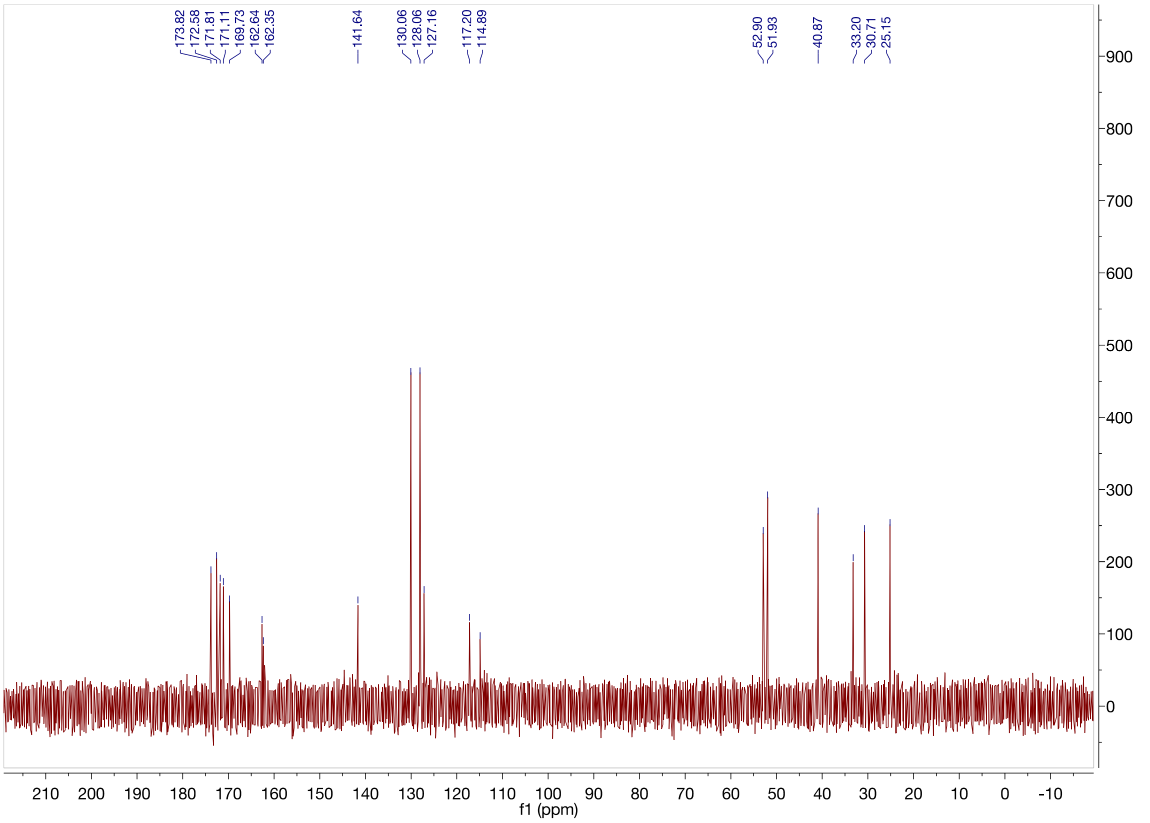

^{13}C {^{1}H} NMR (D_2O, 125.8 MHz) of (**1b**)

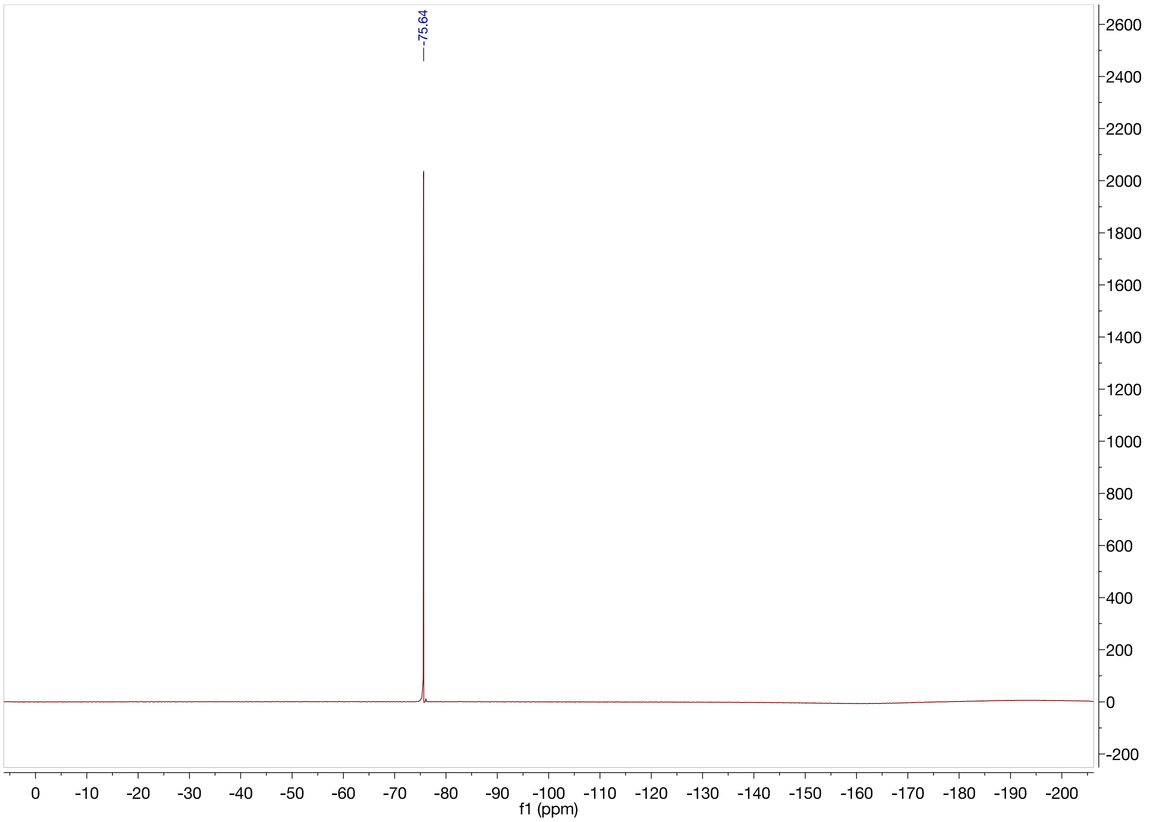

^{19}F {^{1}H} NMR (D_2O, 470.8 MHz) of (**1b**)

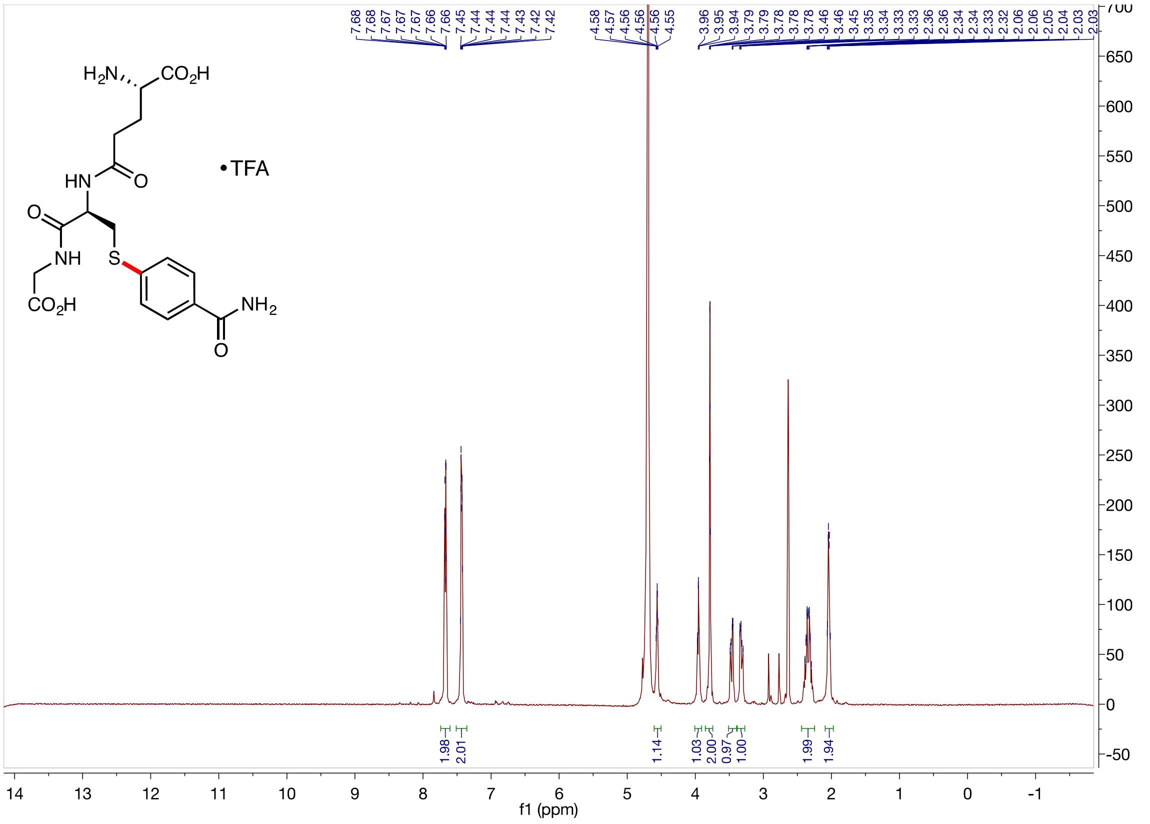

^{1}H NMR (D_2O, 500.4 MHz) of (**1c**)

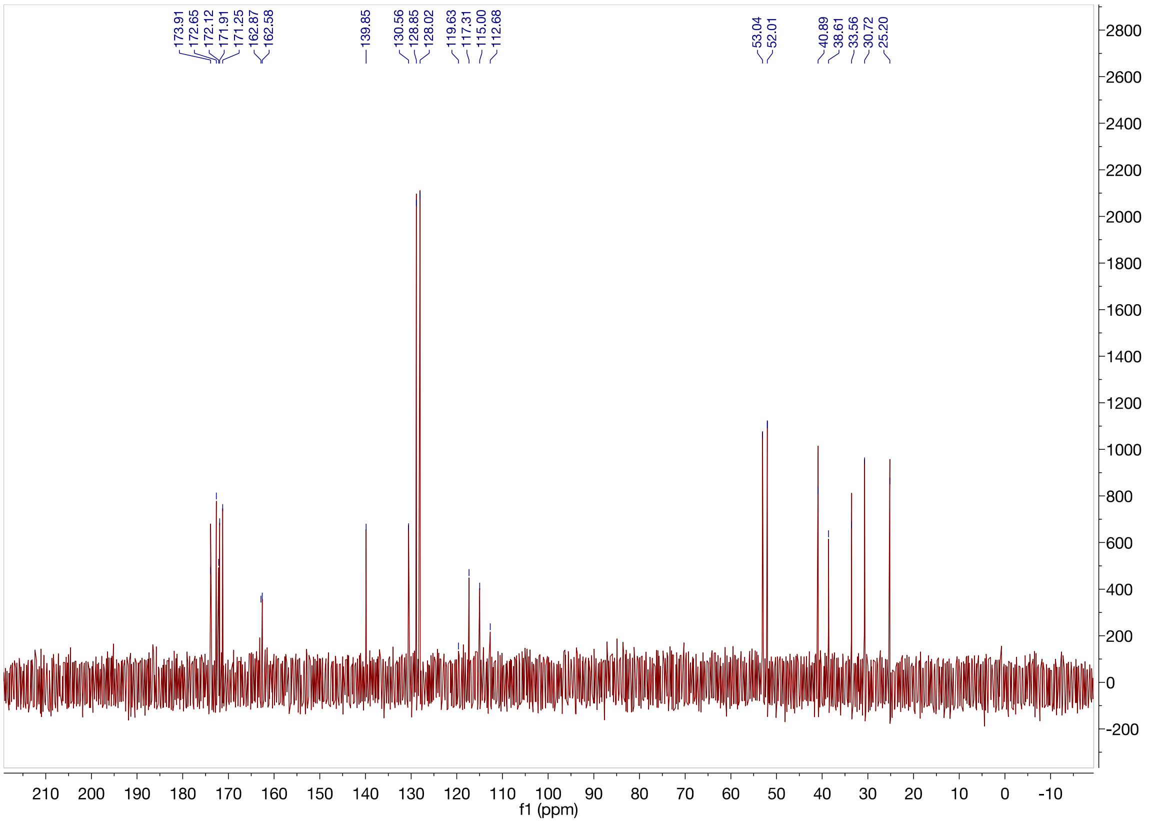

^{13}C {^{1}H} NMR (D_2O, 125.8 MHz) of (**1c**)

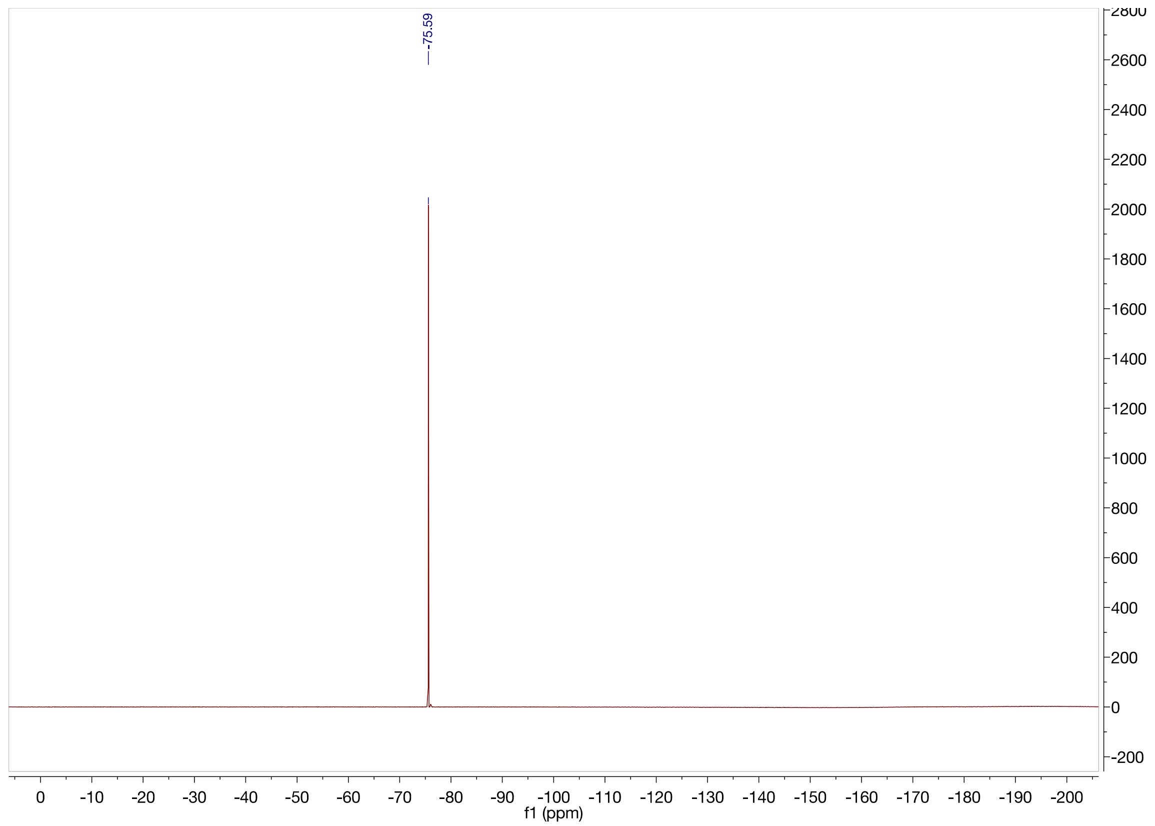

^{19}F {^{1}H} NMR (D_2O, 470.8 MHz) of (**1c**)

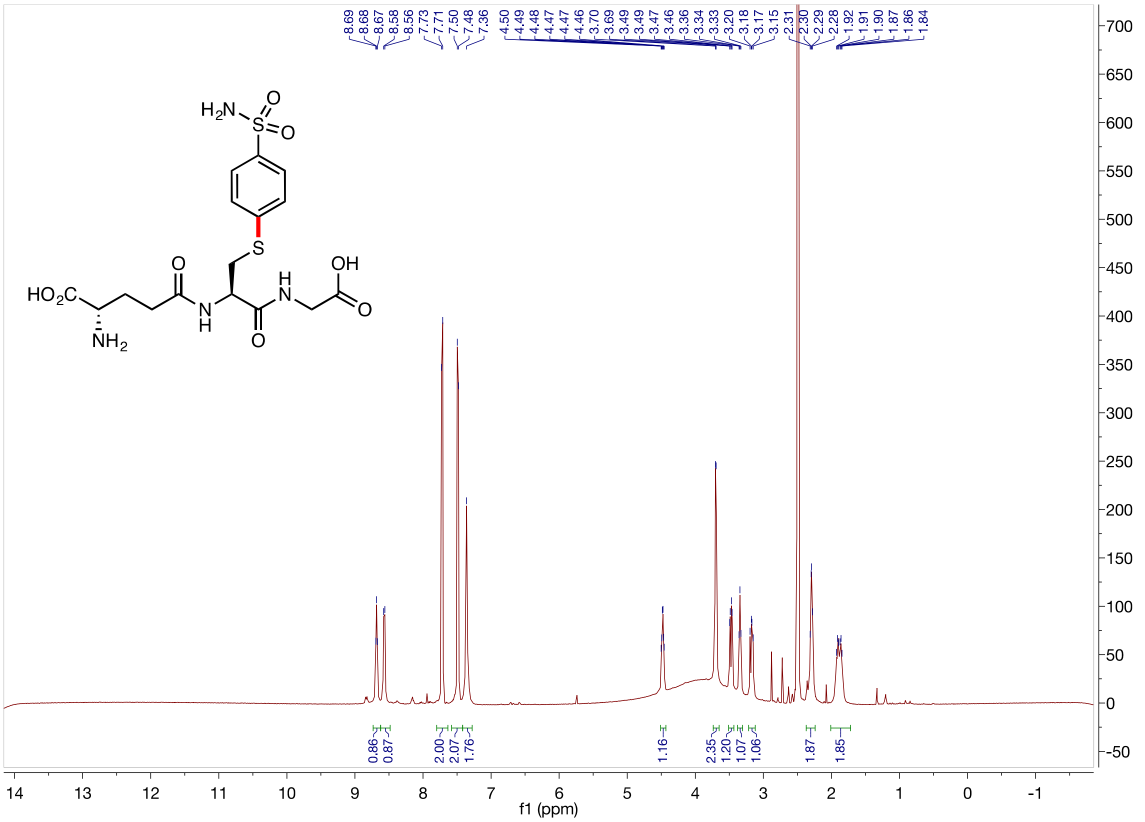

^{1}H NMR (DMSO-d_6, 500.4 MHz) of (**1d**)

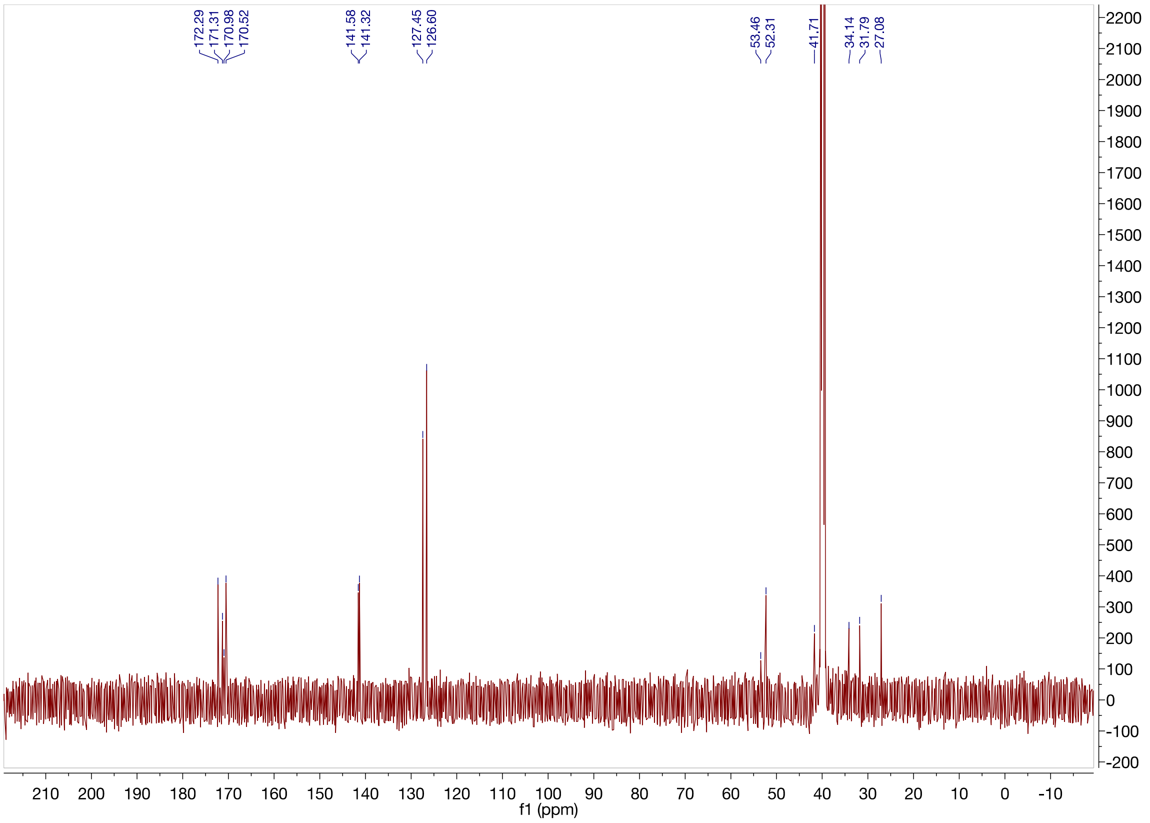

^{13}C {^{1}H} NMR (DMSO-d_6, 125.8 MHz) of (**1d**)

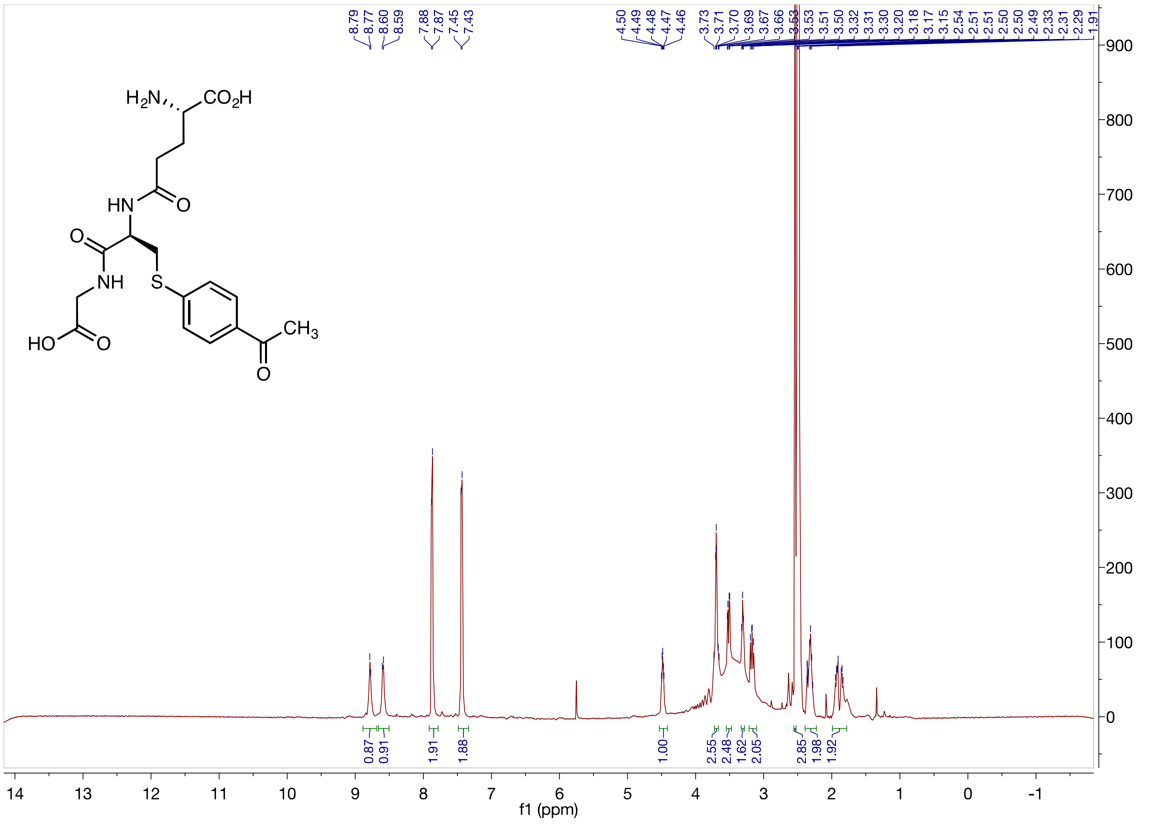

^{1}H NMR (DMSO-d_6, 500.4 MHz) of (**1e**)

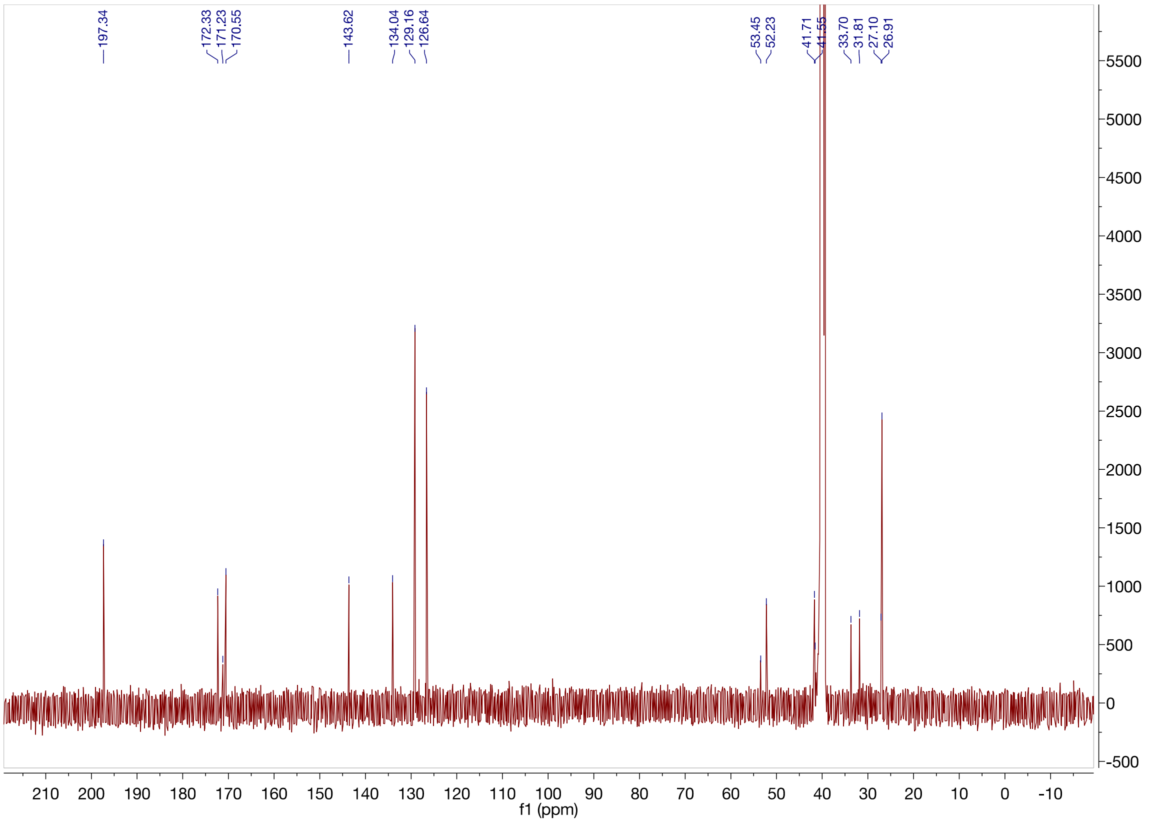

^{13}C {^{1}H} NMR (DMSO-d_6, 125.8 MHz) of (**1e**)

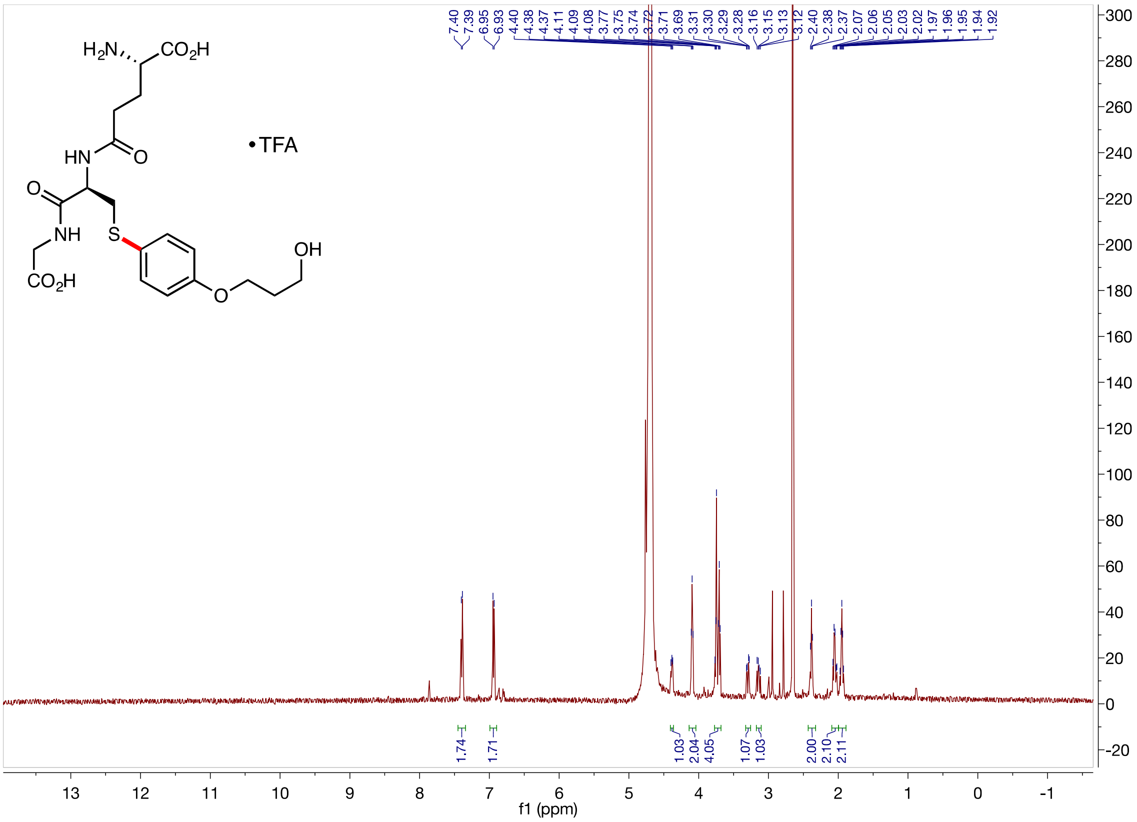

^{1}H NMR (D_2O, 500.4 MHz) of (**1f**)

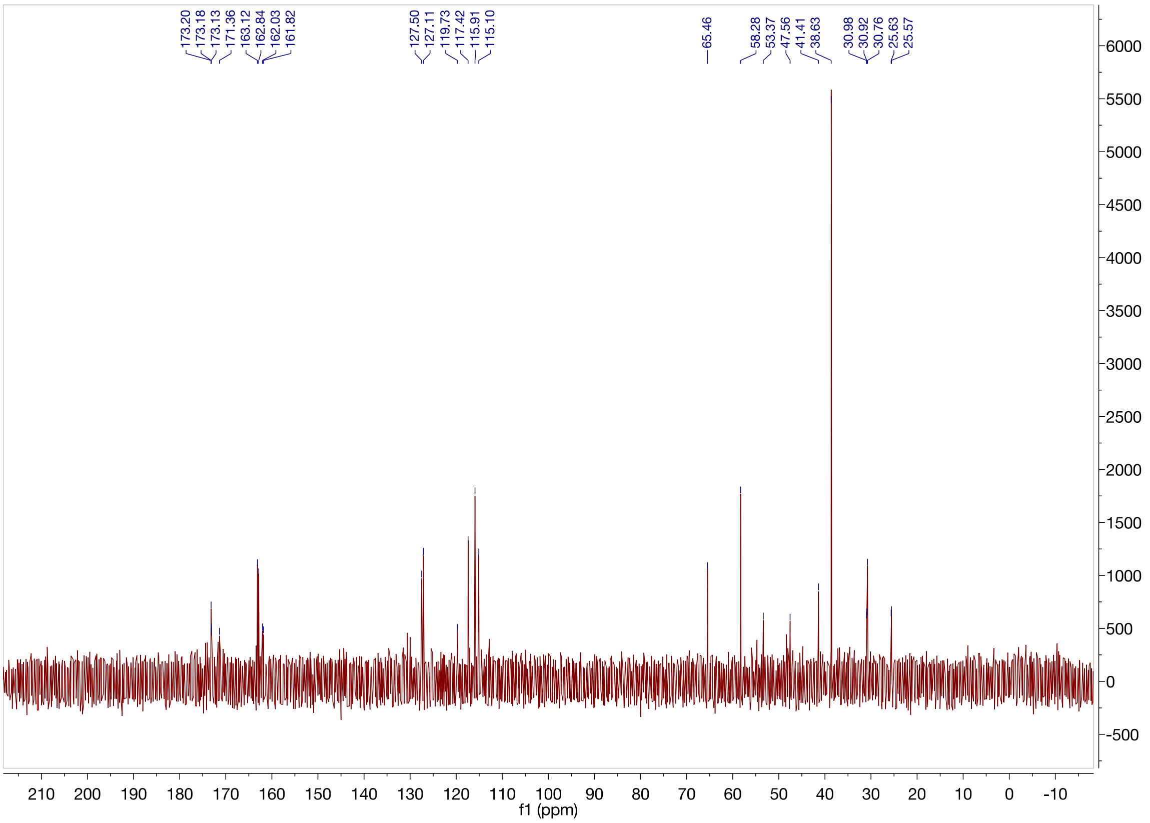

^{13}C {^{1}H} NMR (D_2O, 125.8 MHz) of (**1f**)

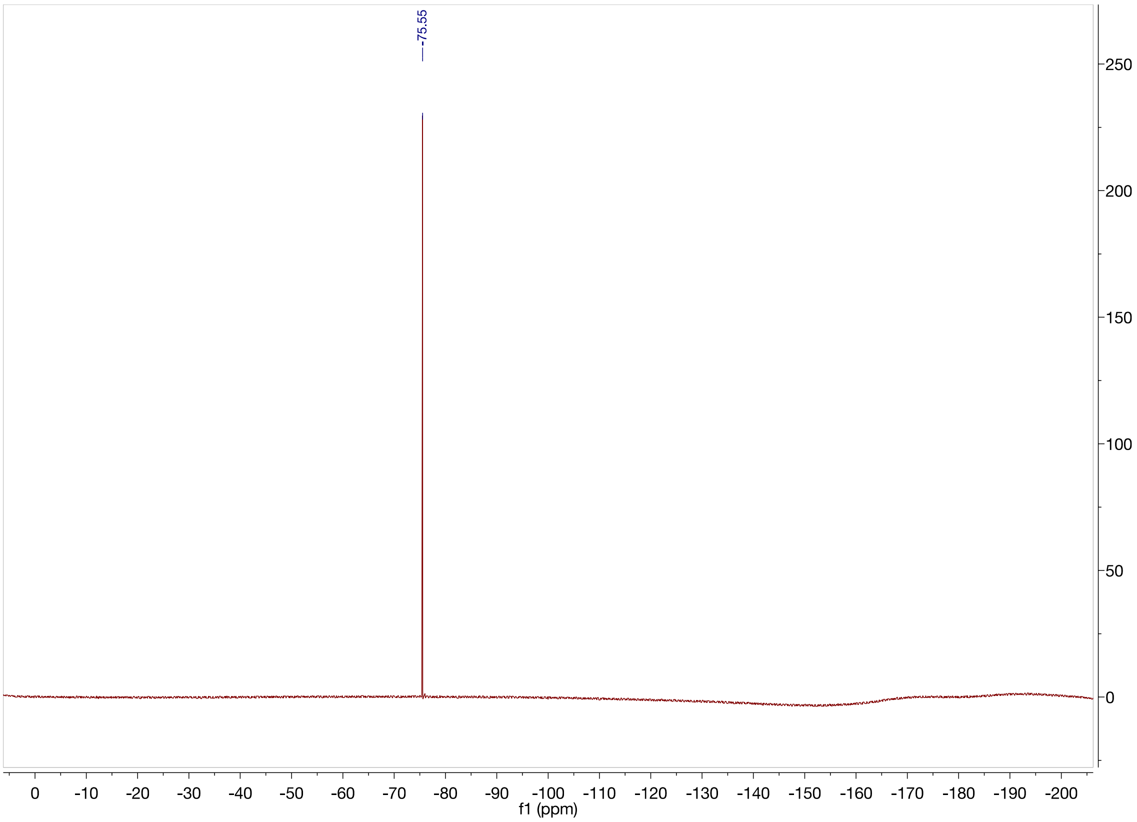

$^{19}F\{^{1}H\}$ NMR (D_2O, 470.8 MHz) of (**1f**)

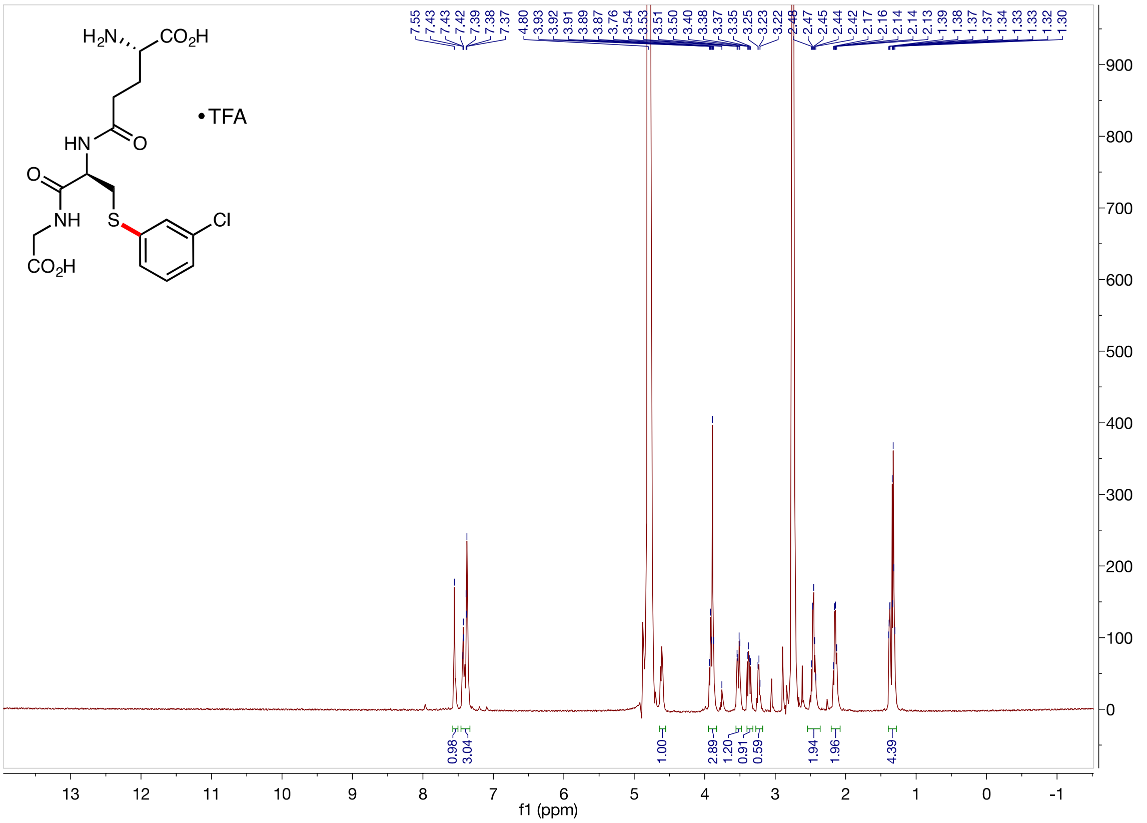

^{1}H NMR (D$_2$O, 500.4 MHz) of (**1g**)

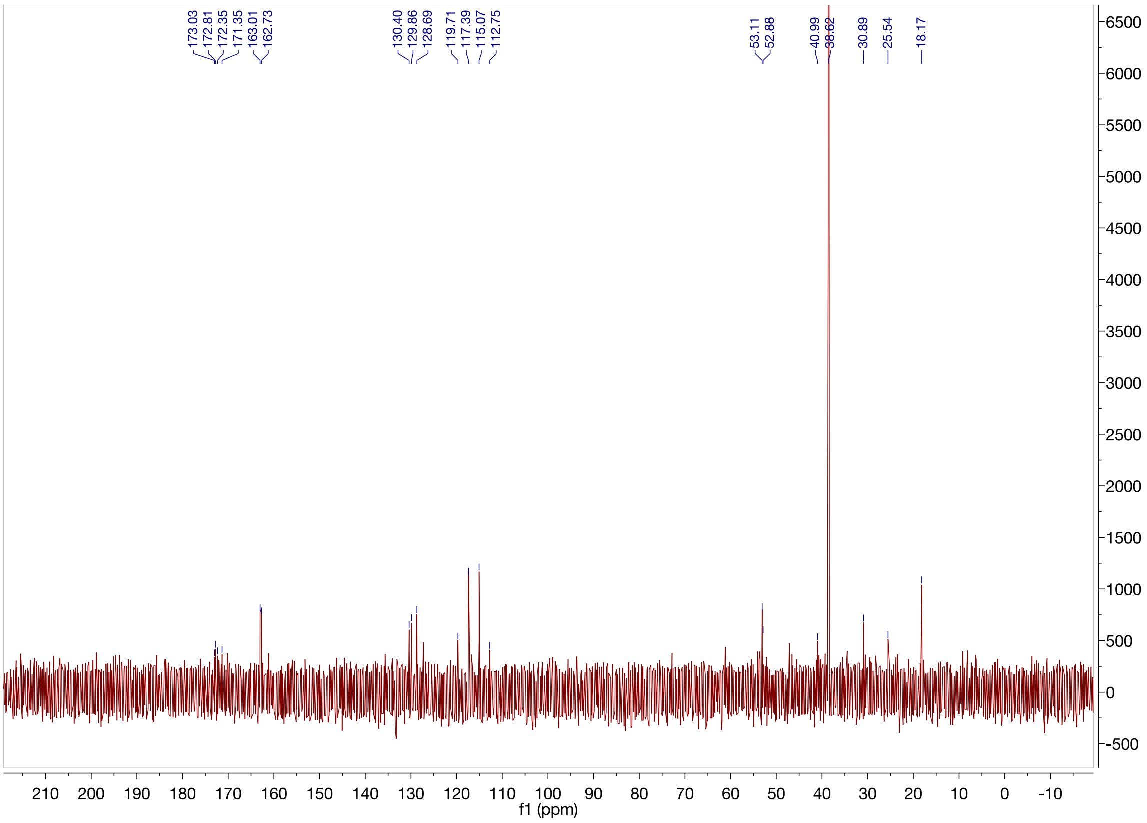

^{13}C {^{1}H} NMR (D$_2$O, 125.8 MHz) of (**1g**)

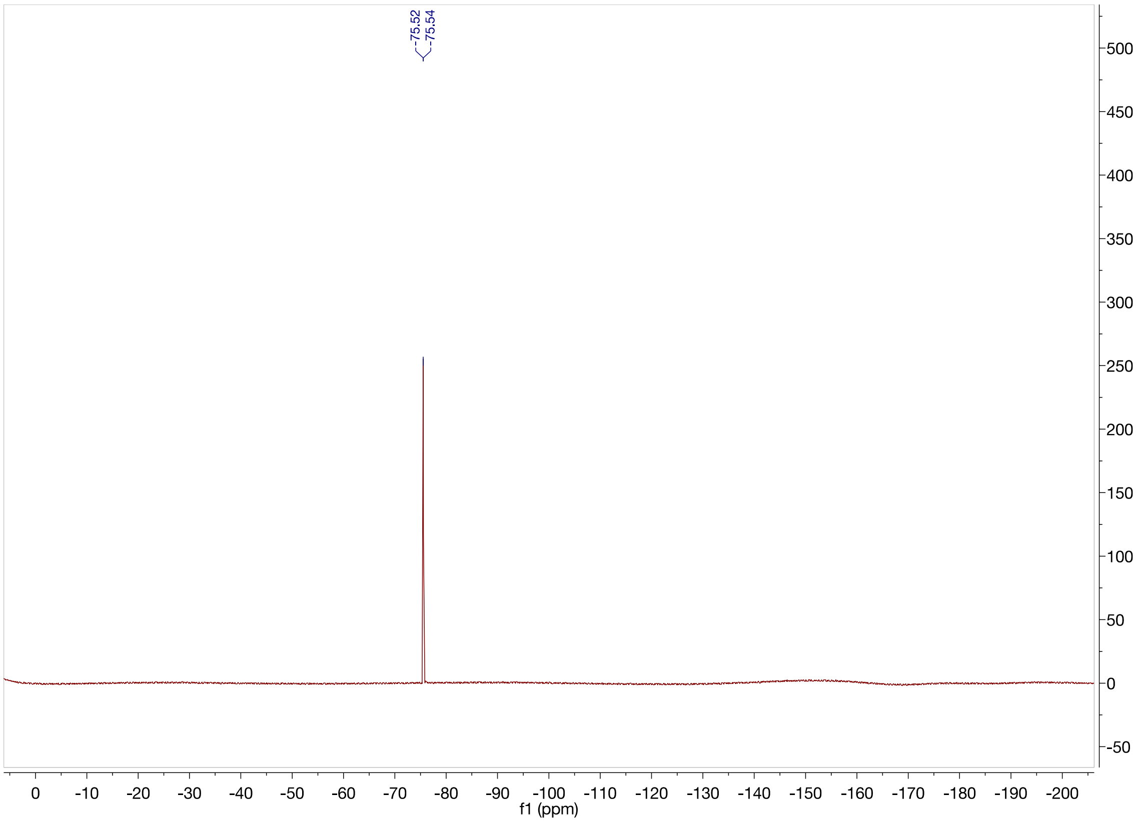

^{19}F {^{1}H} NMR (D_2O, 470.8 MHz) of (**1g**)

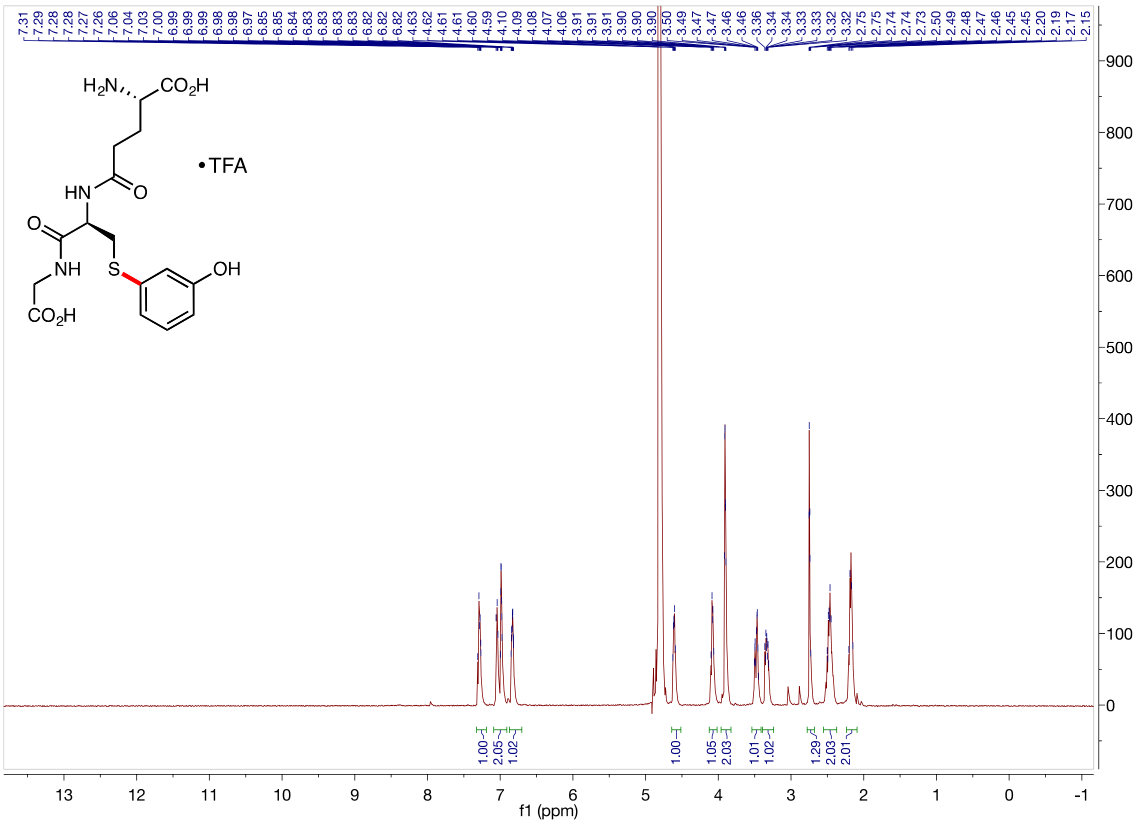

^{1}H NMR (D_2O, 500.4 MHz) of (**1h**)

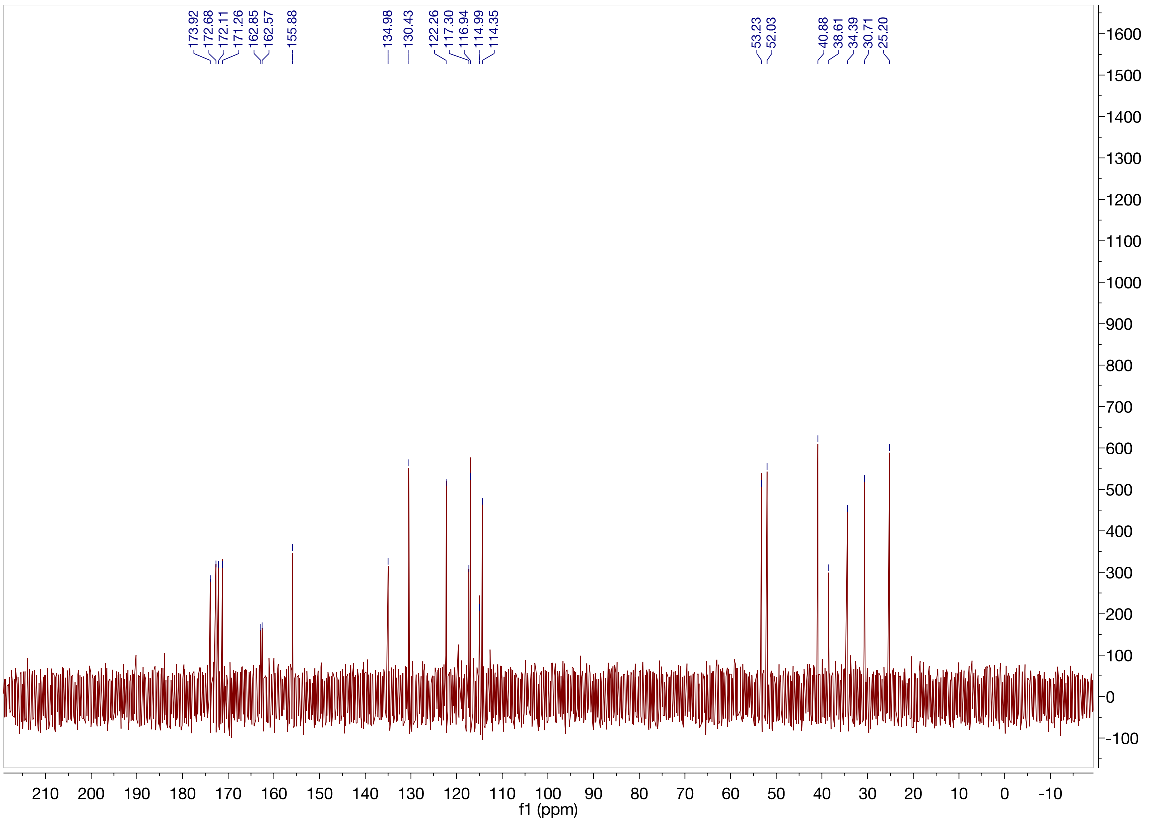

^{13}C {^{1}H} NMR (D_2O, 125.8 MHz) of (**1h**)

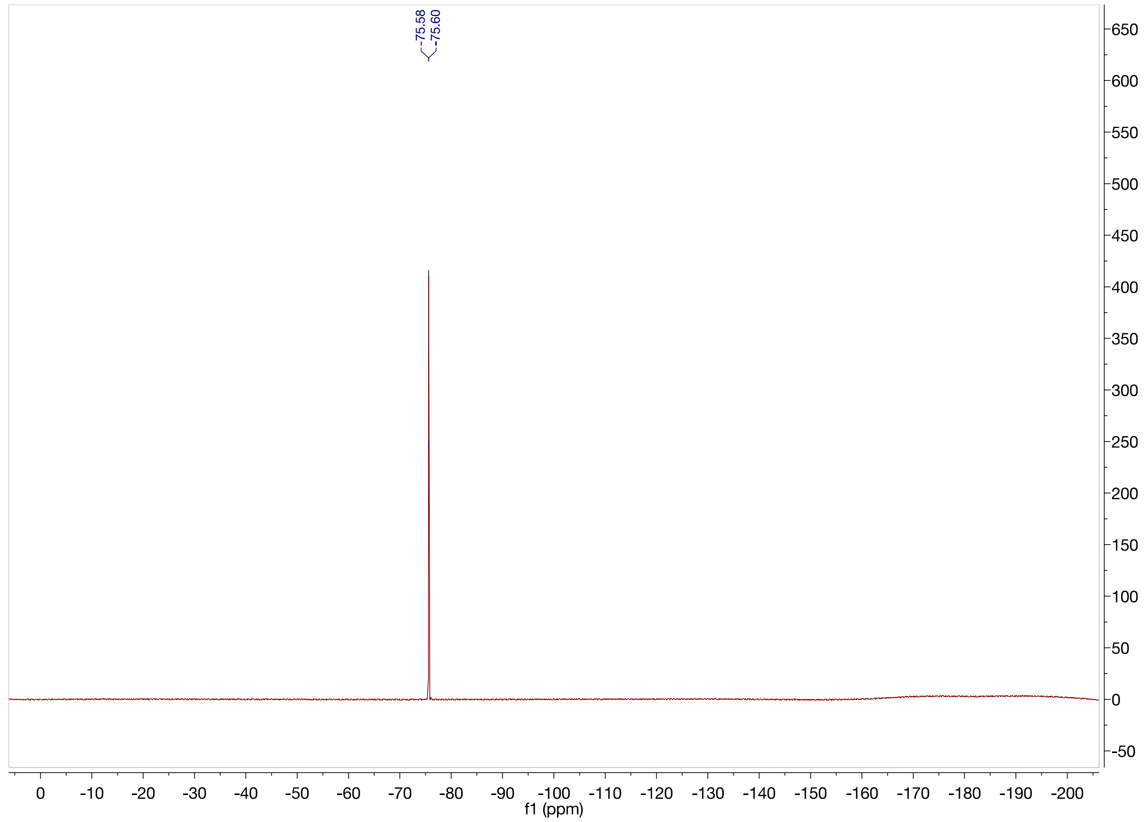

^{19}F {^{1}H} NMR (D_2O, 470.8 MHz) of (**1h**)

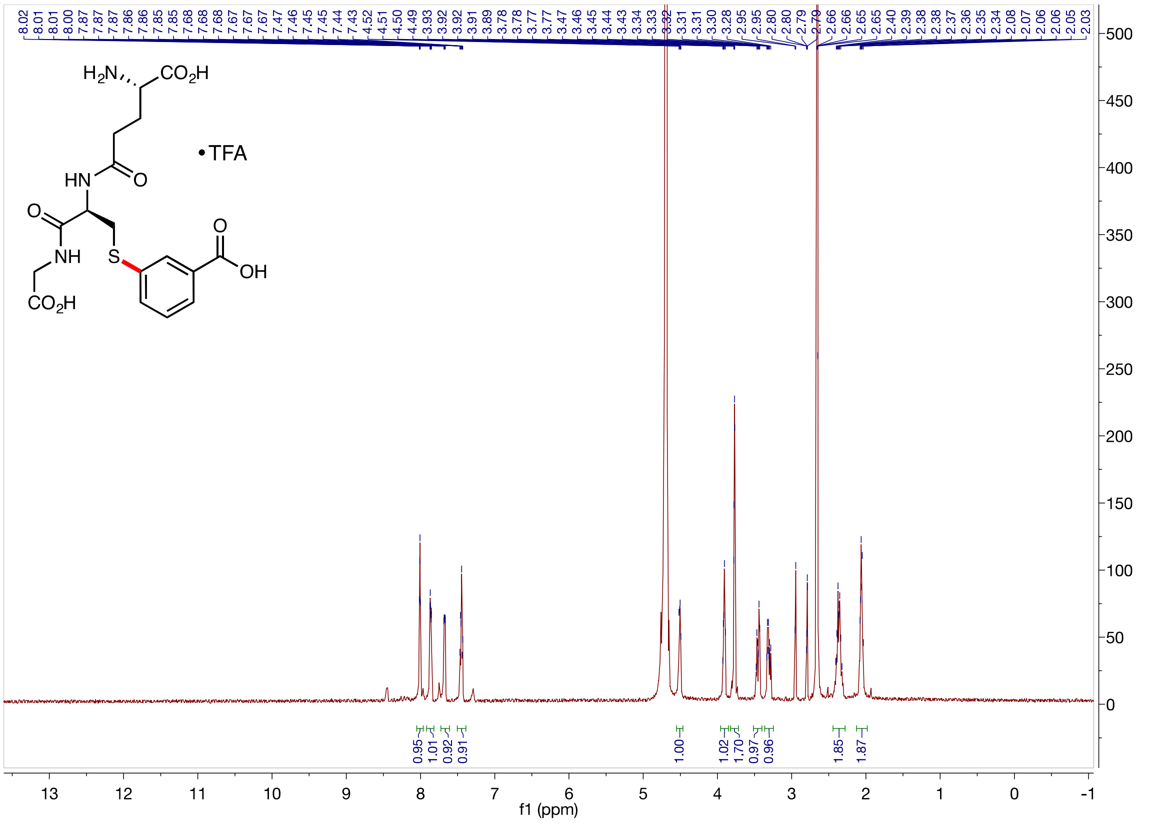

^{1}H NMR (D_2O, 500.4 MHz) of (**1i**)

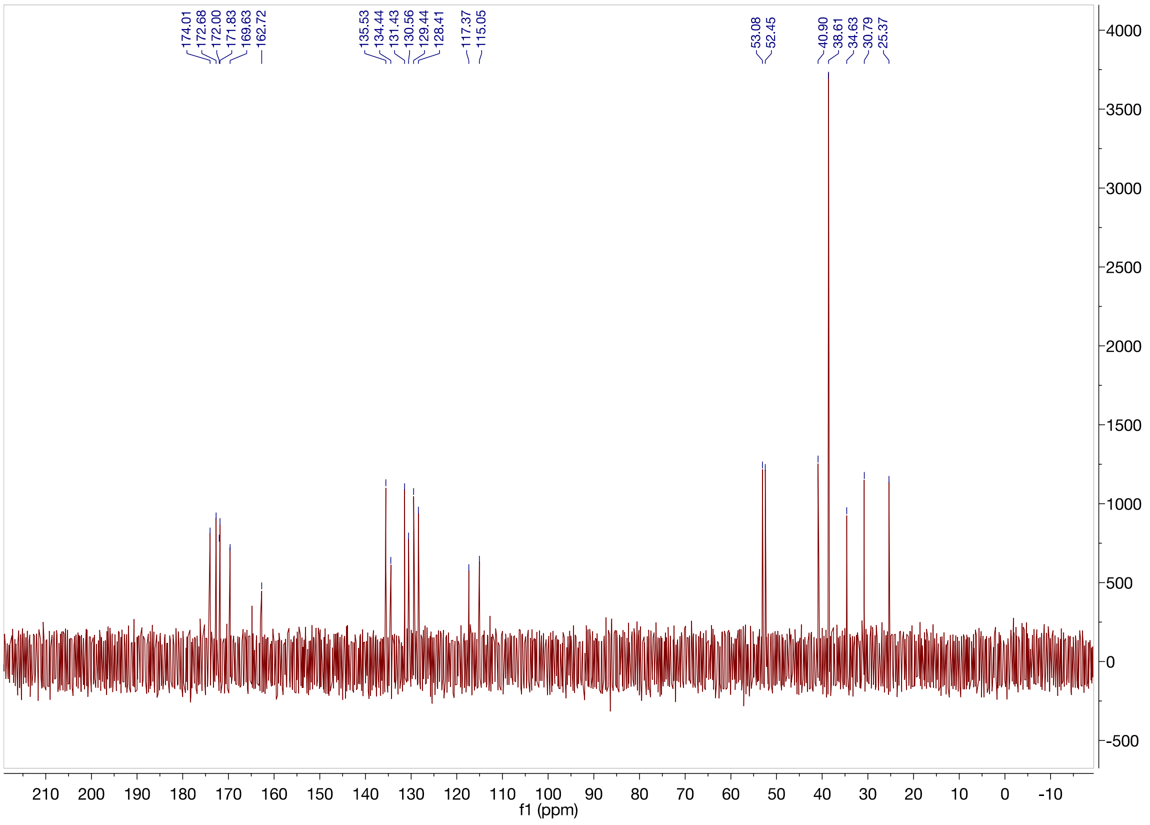

^{13}C {^{1}H} NMR (D_2O, 125.8 MHz) of (**1i**)

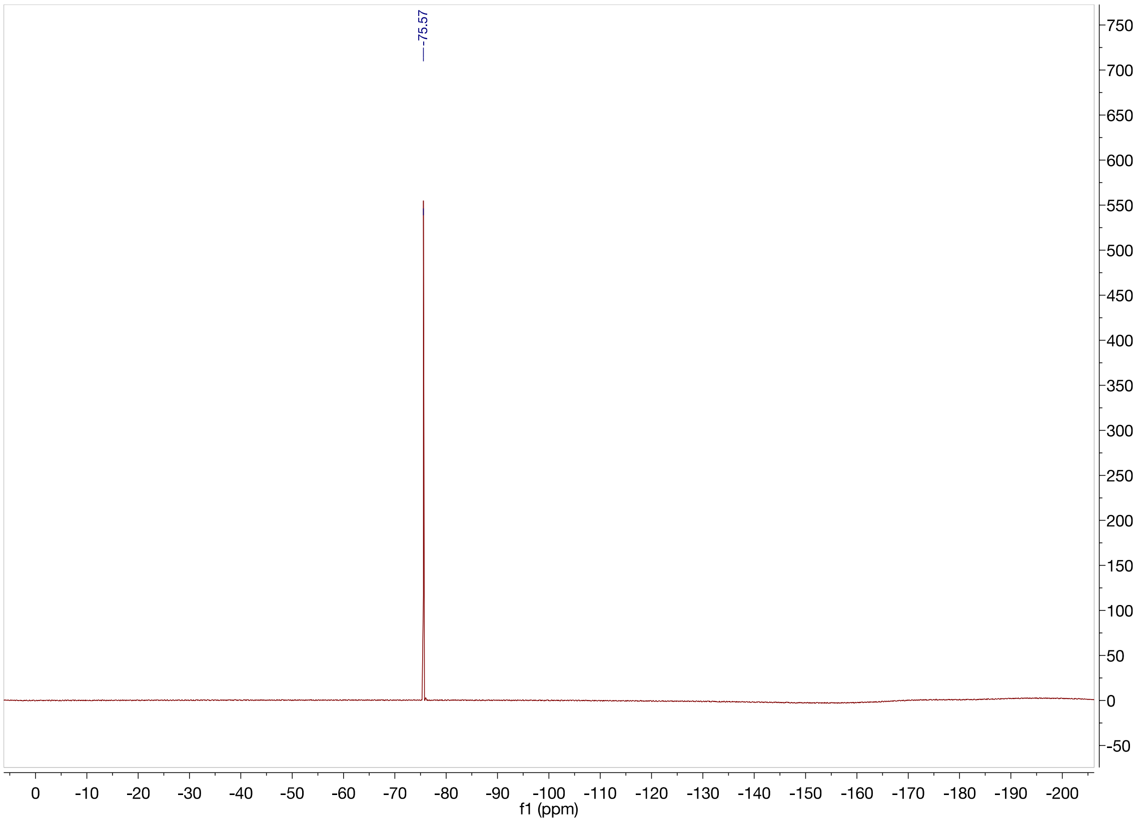

$^{19}F\ \{^{1}H\}$ NMR (D_2O, 470.8 MHz) of (**1i**)

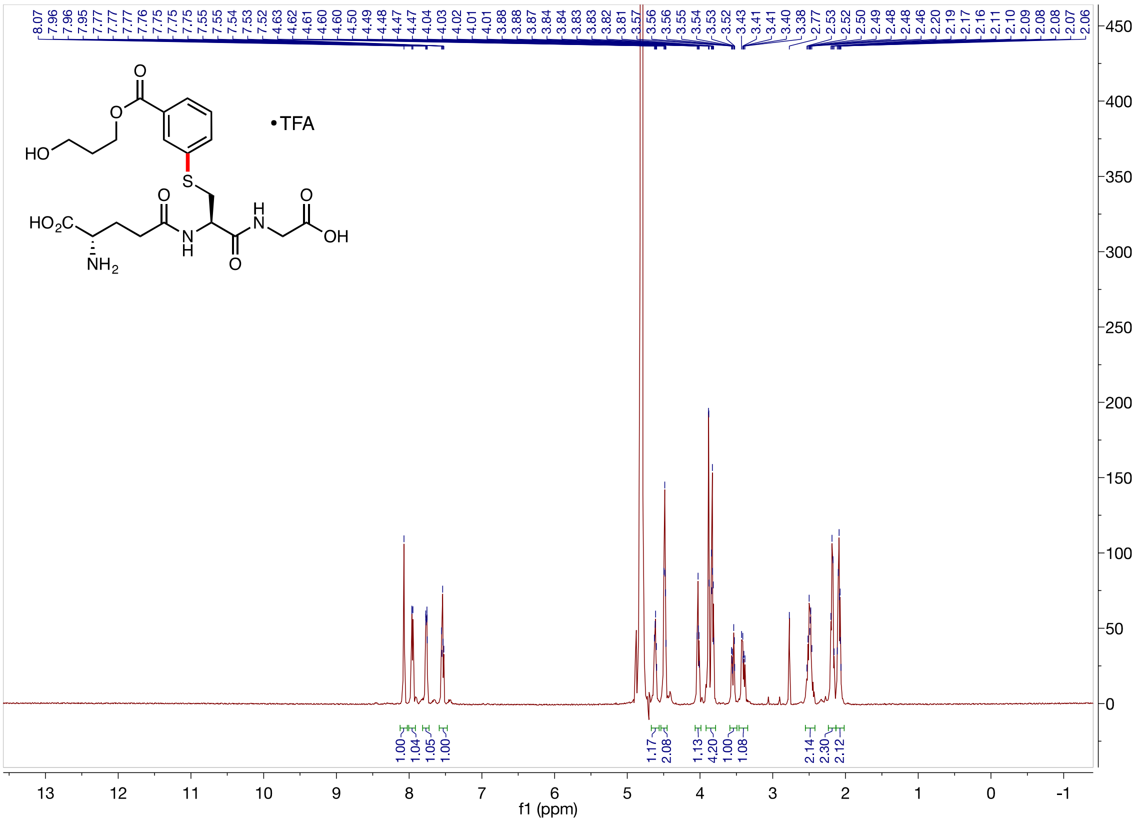

^{1}H NMR (D_2O, 500.4 MHz) of (**1j**)

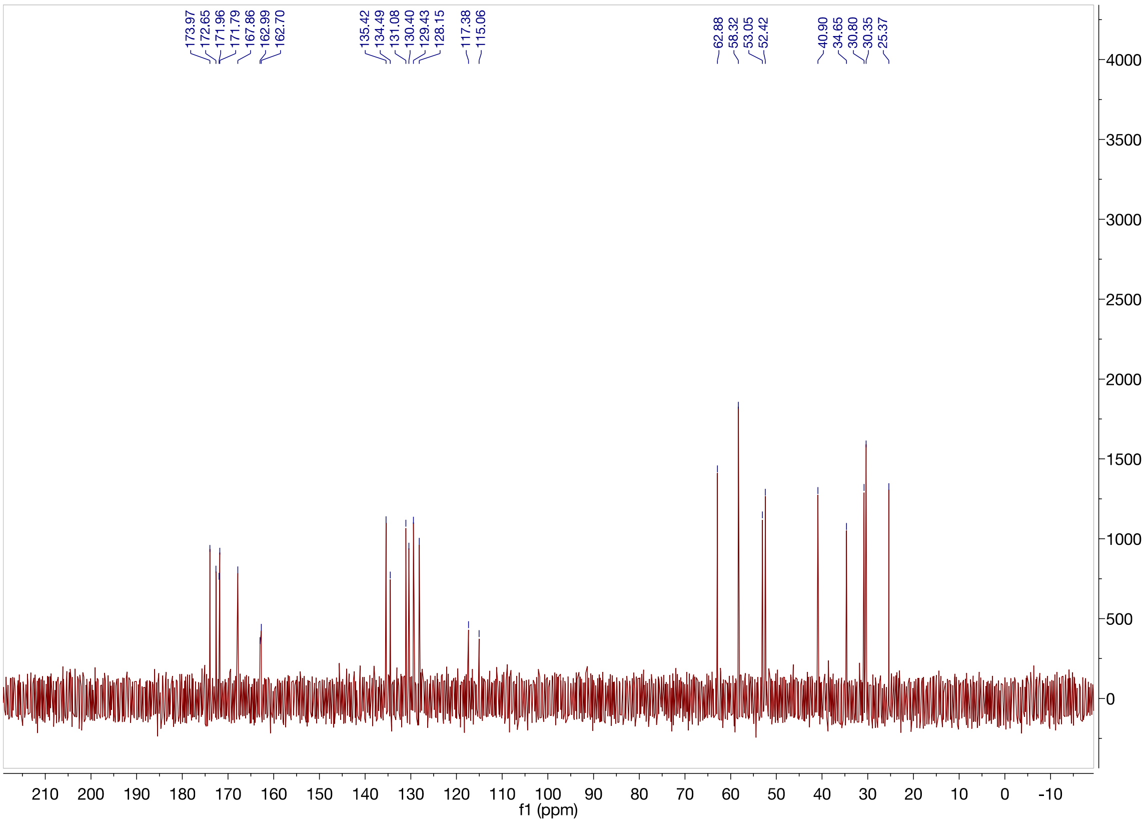

^{13}C {^{1}H} NMR (D_2O, 125.8 MHz) of (**1j**)

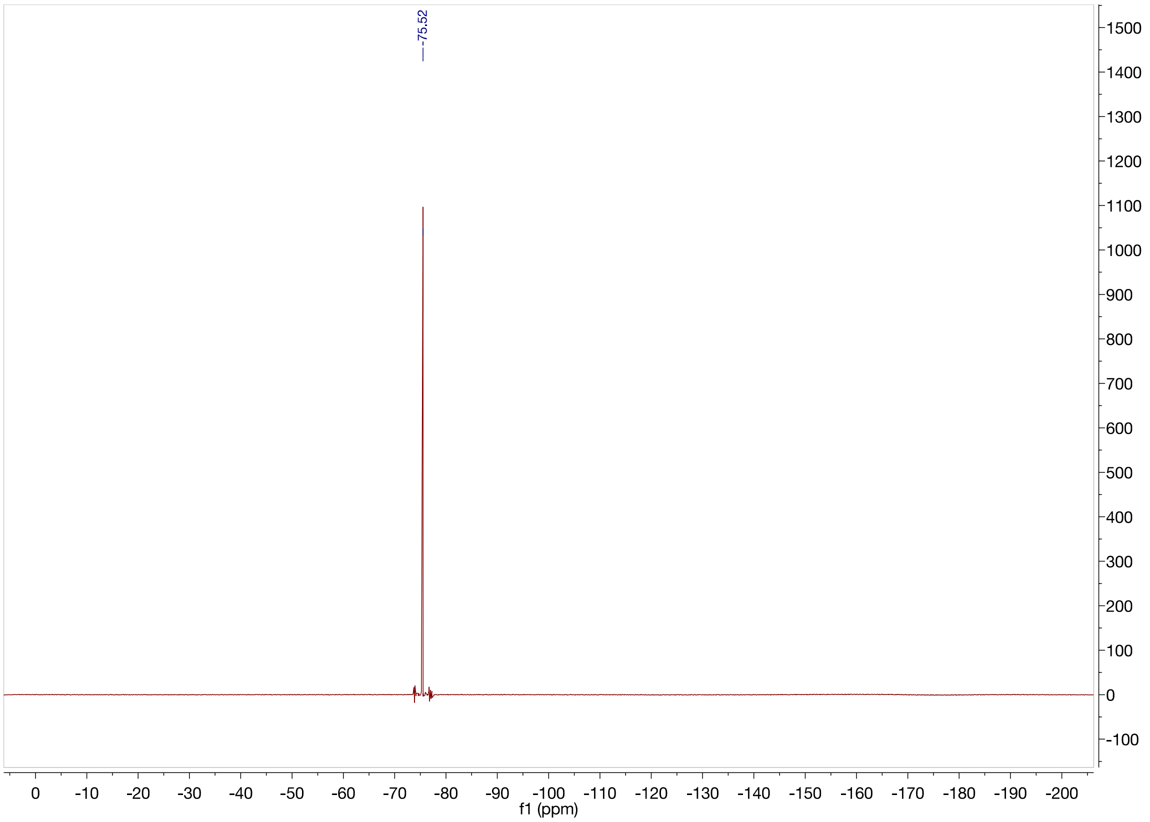

$^{19}F\ \{^{1}H\}$ NMR (D_2O, 470.8 MHz) of (**1j**)

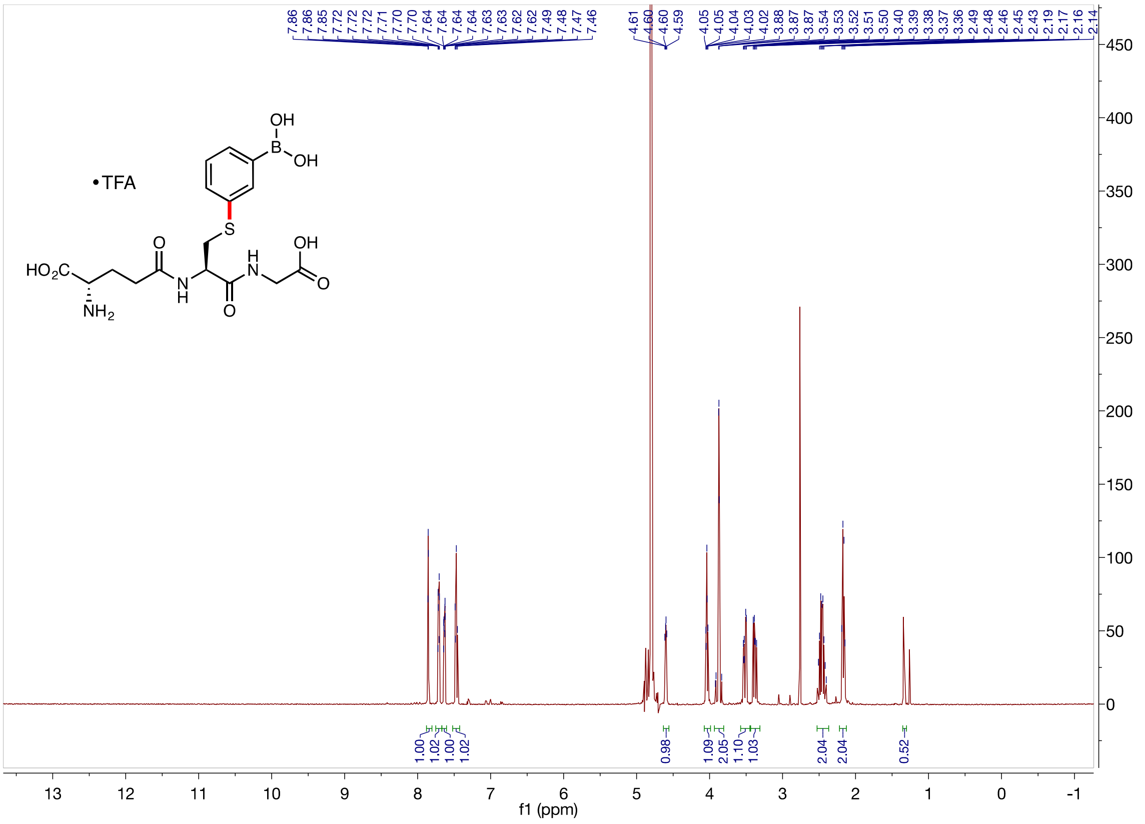

^{1}H NMR (D_2O, 500.4 MHz) of (**1k**)

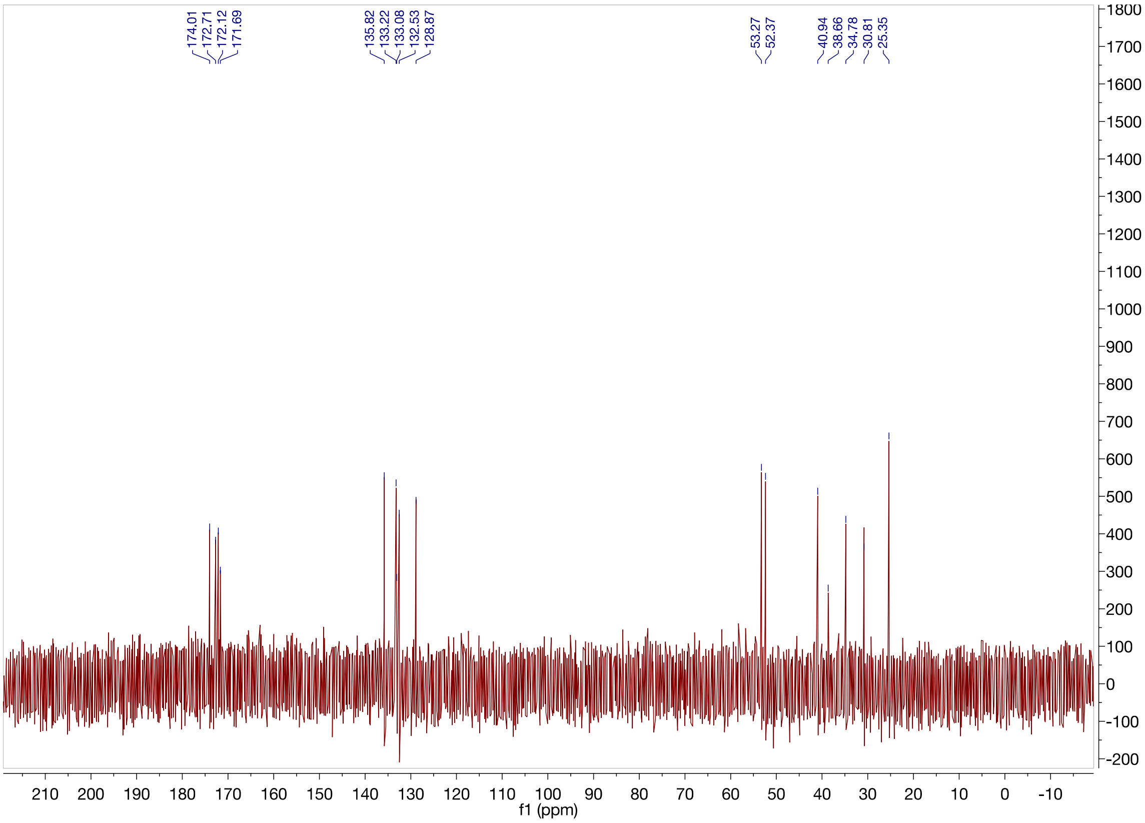

^{13}C {^{1}H} NMR (D_2O, 125.8 MHz) of (**1k**)

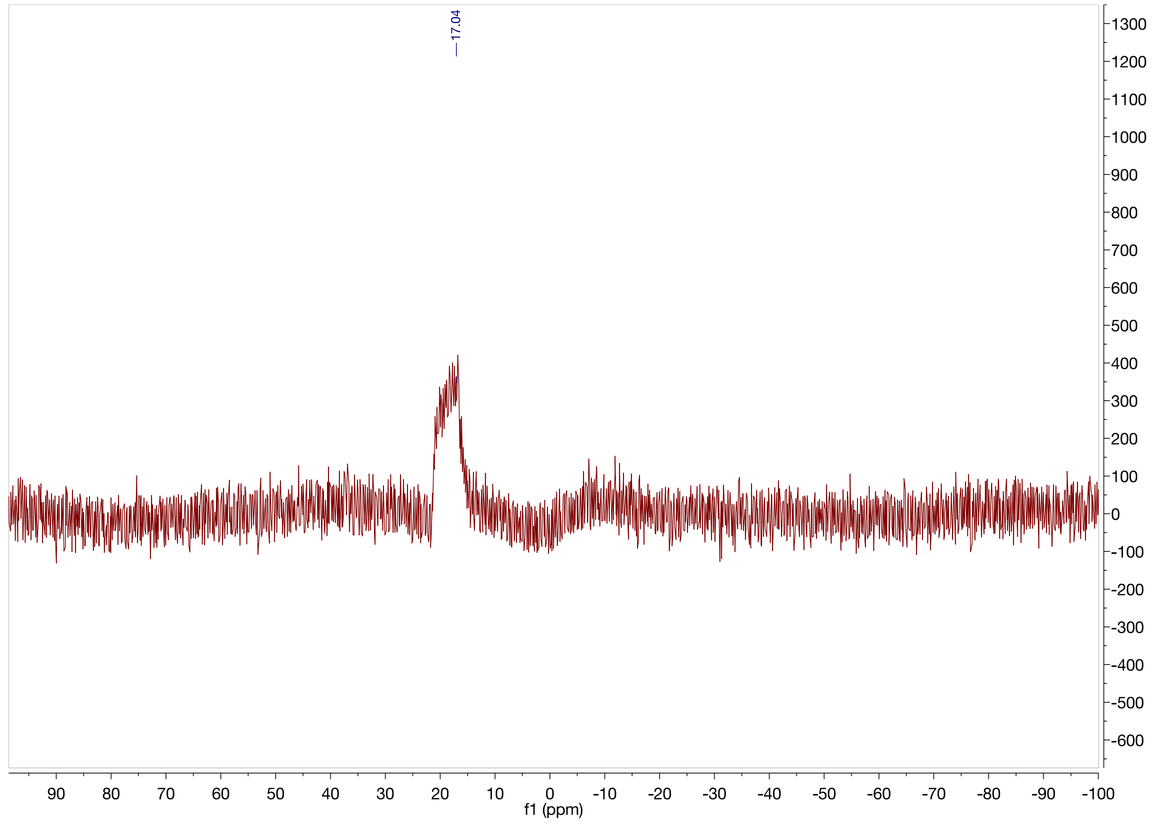

^{11}B NMR (D_2O, 500.4 MHz) of (**1k**)

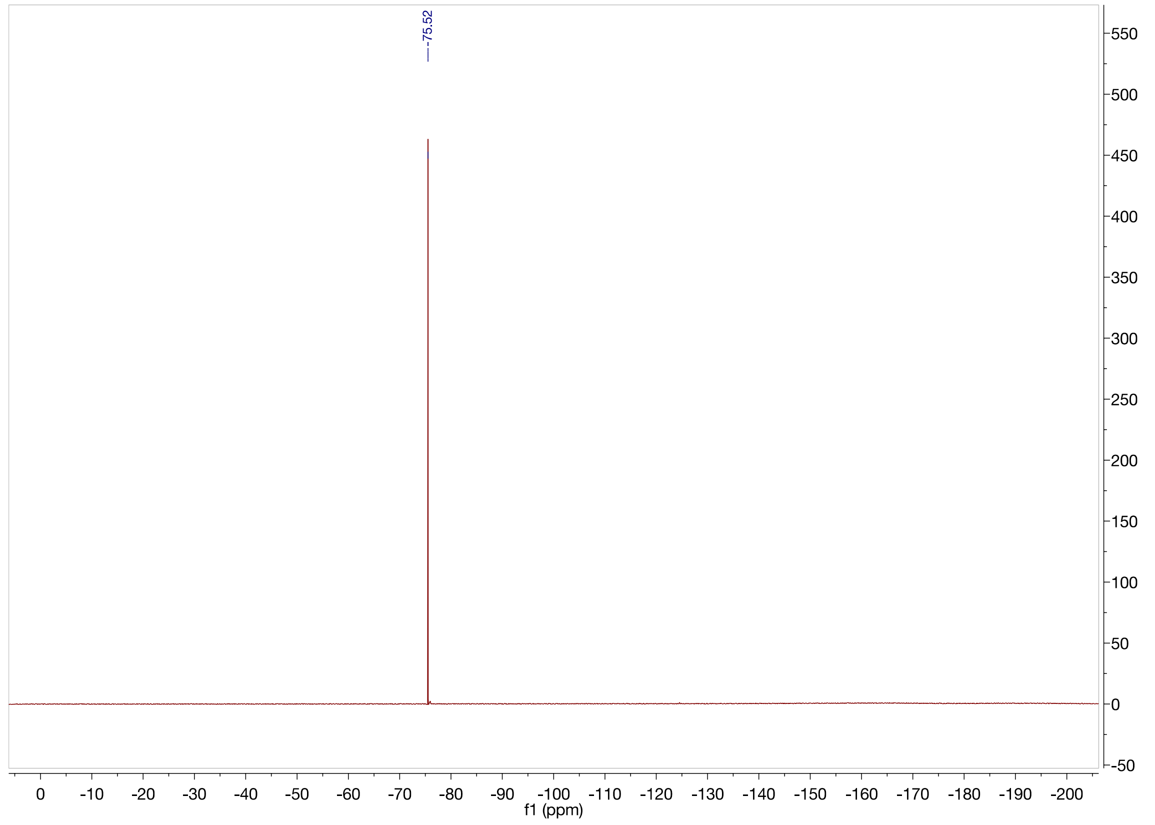

^{19}F {^{1}H} NMR (D_2O, 470.8 MHz) of (**1k**)

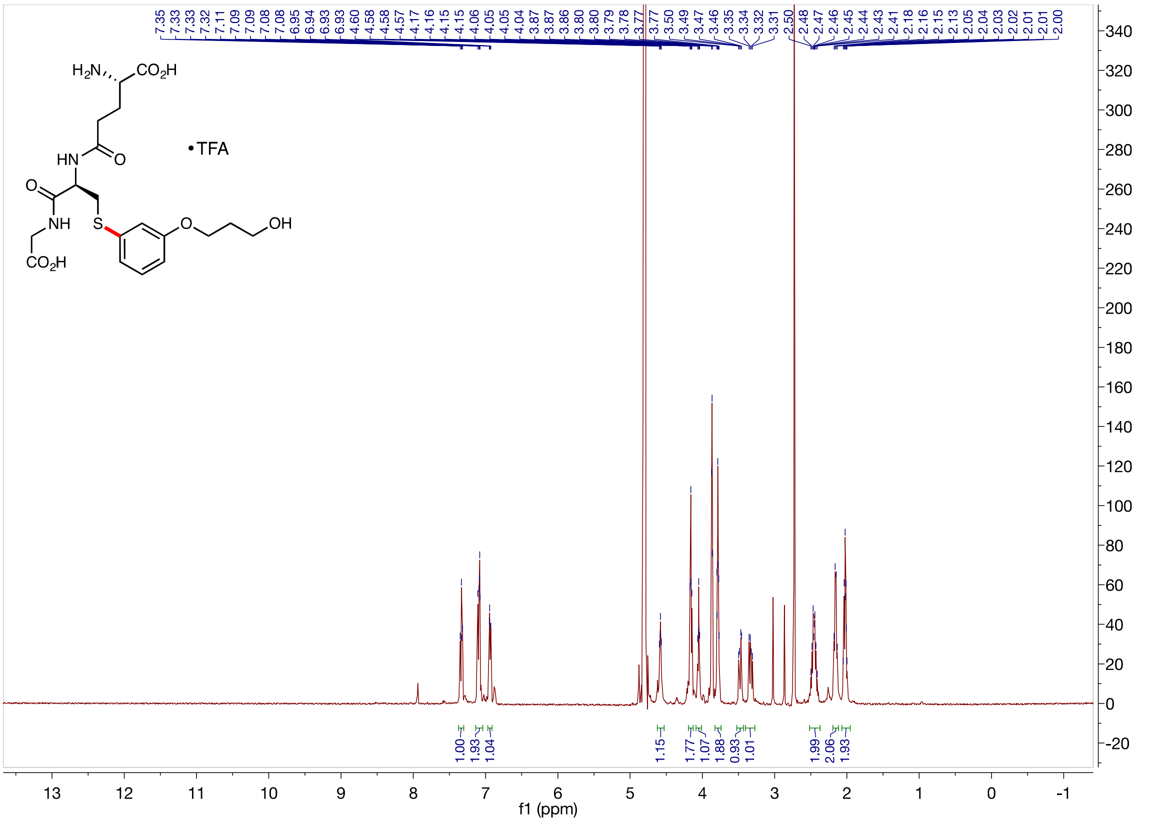

^{1}H NMR (D_2O, 500.4 MHz) of (**1l**)

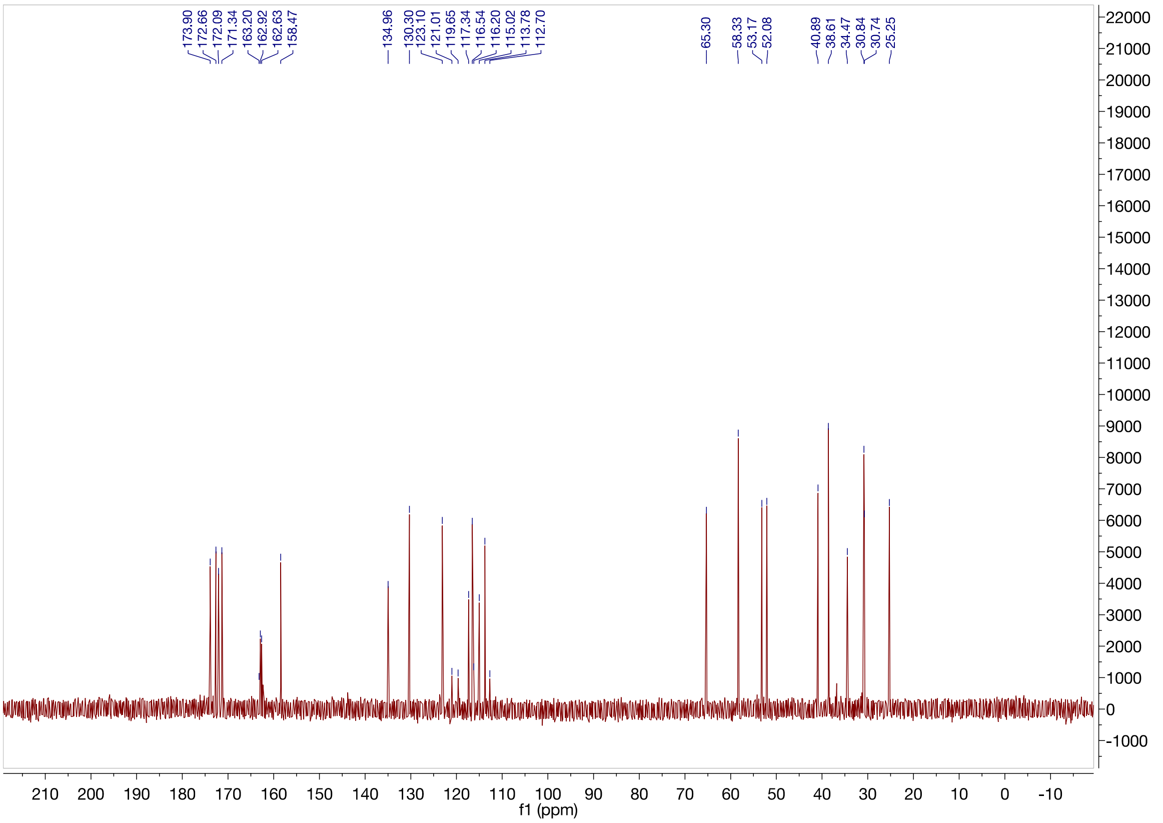

^{13}C {^{1}H} NMR (D_2O, 125.8 MHz) of (**1l**)

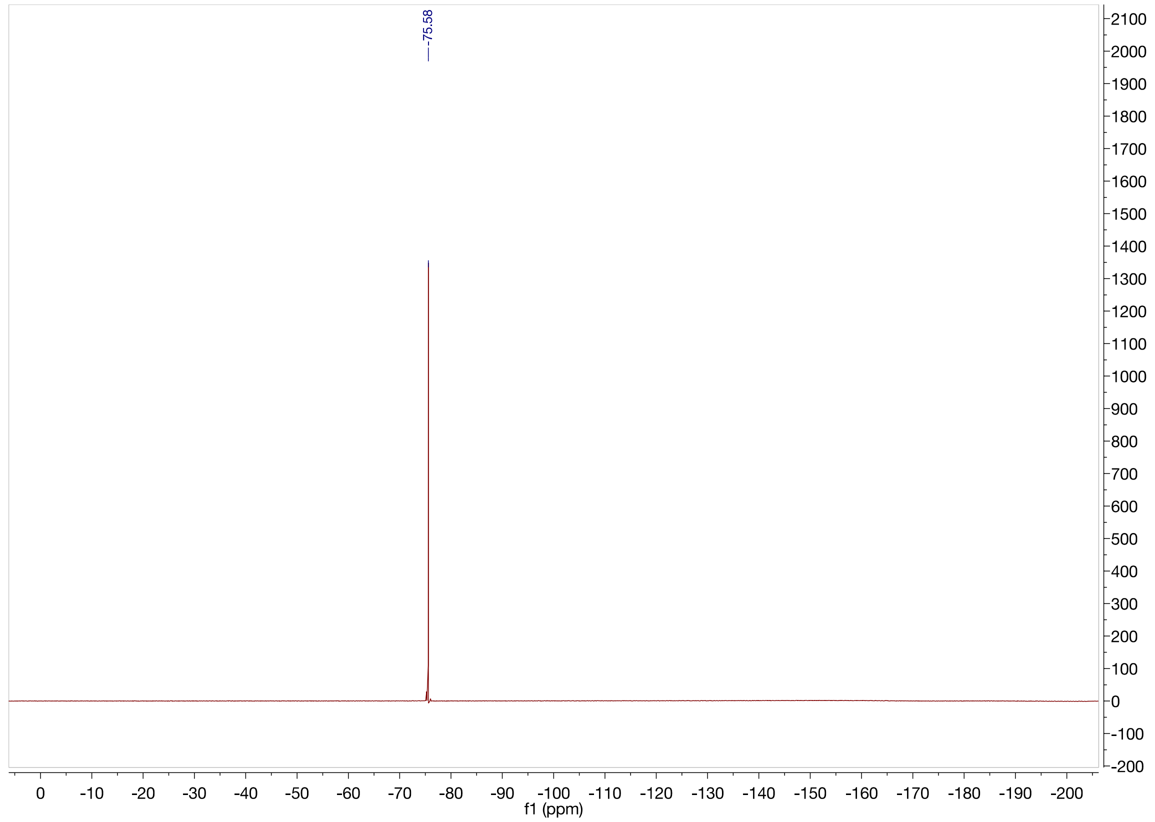

$^{19}F\ \{^{1}H\}$ NMR (D_2O, 470.8 MHz) of (**1l**)

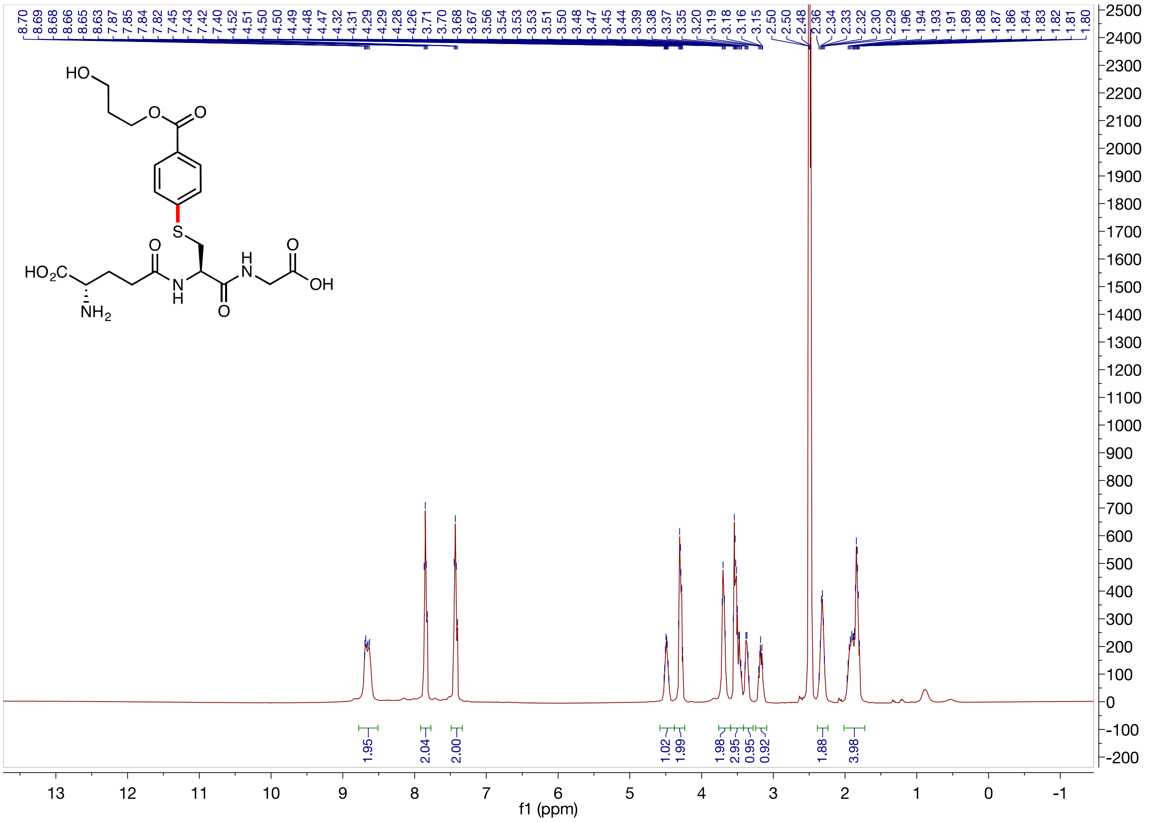

^{1}H NMR (DMSO-d_6, 500.4 MHz) of (**1m**)

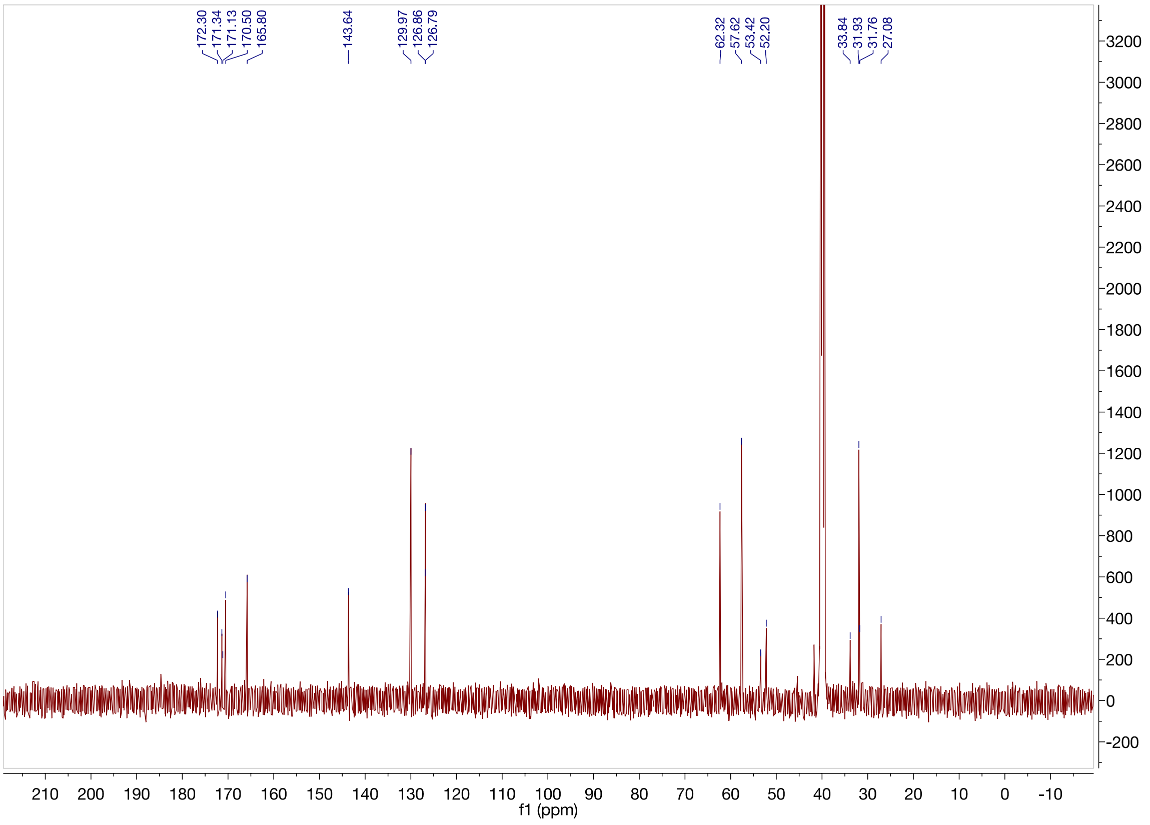

^{13}C {^{1}H} NMR (DMSO-d_6, 125.8 MHz) of (**1m**)

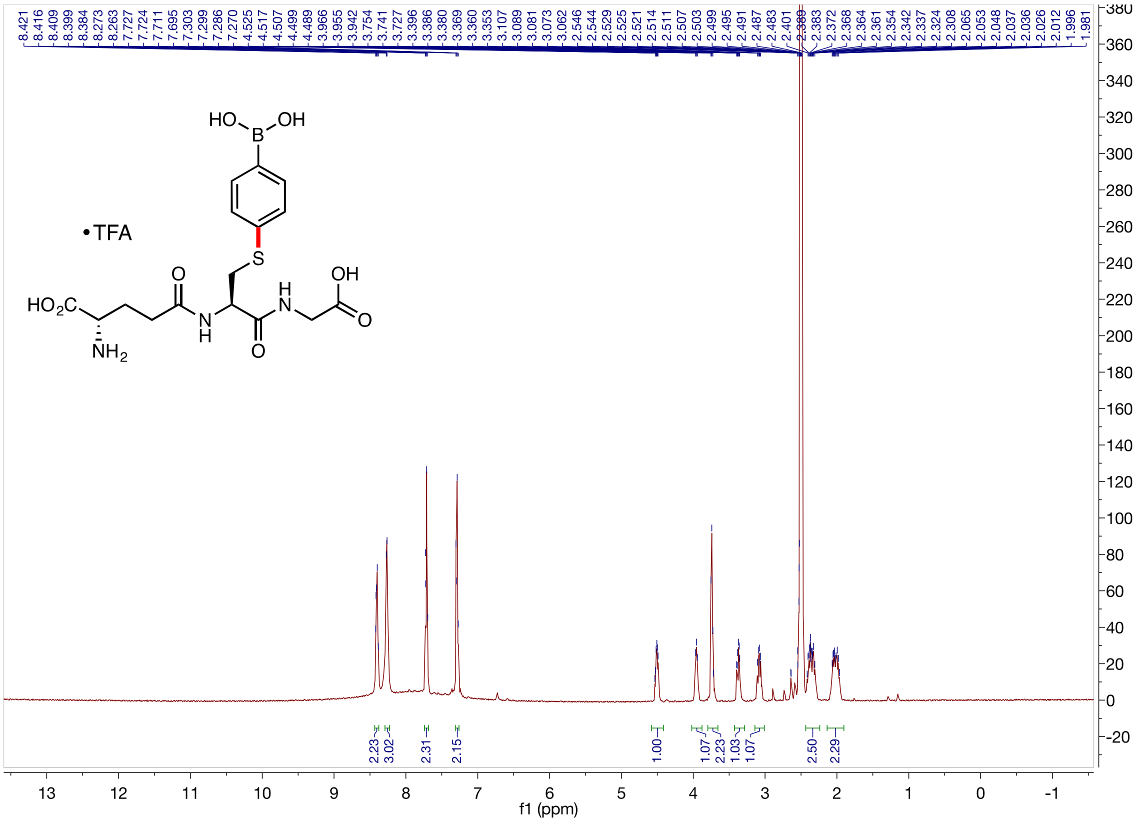

^{1}H NMR (DMSO-d_6, 500.4 MHz) of (**1n**)

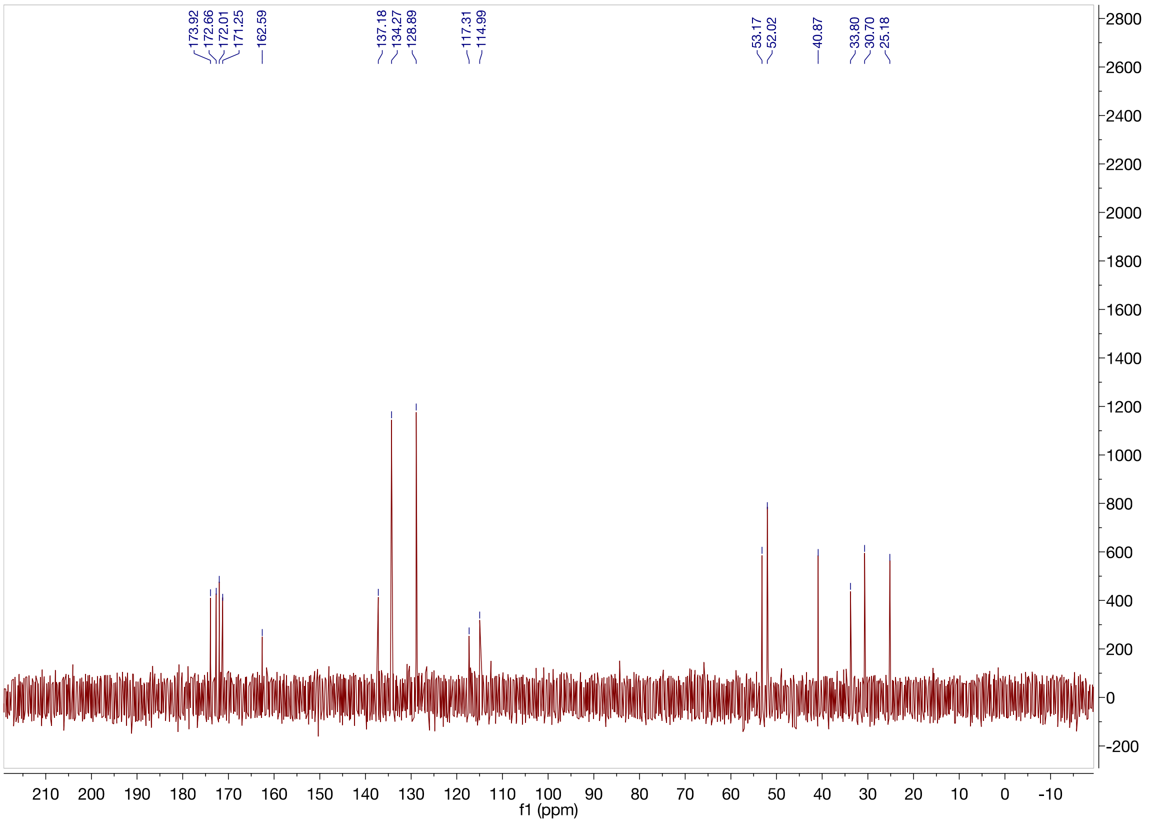

^{13}C {^{1}H} NMR (D_2O, 125.8 MHz) of (**1n**)

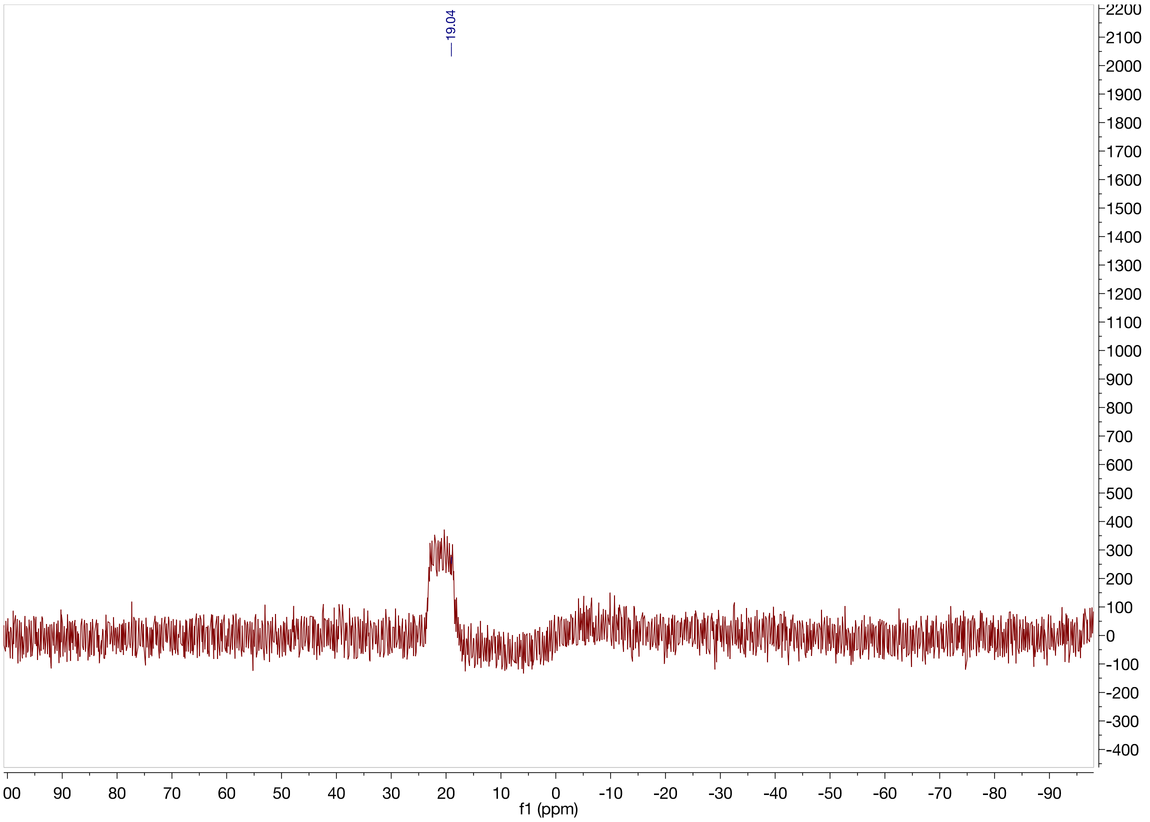

^{11}B NMR (D_2O, 500.4 MHz) of (**1n**)

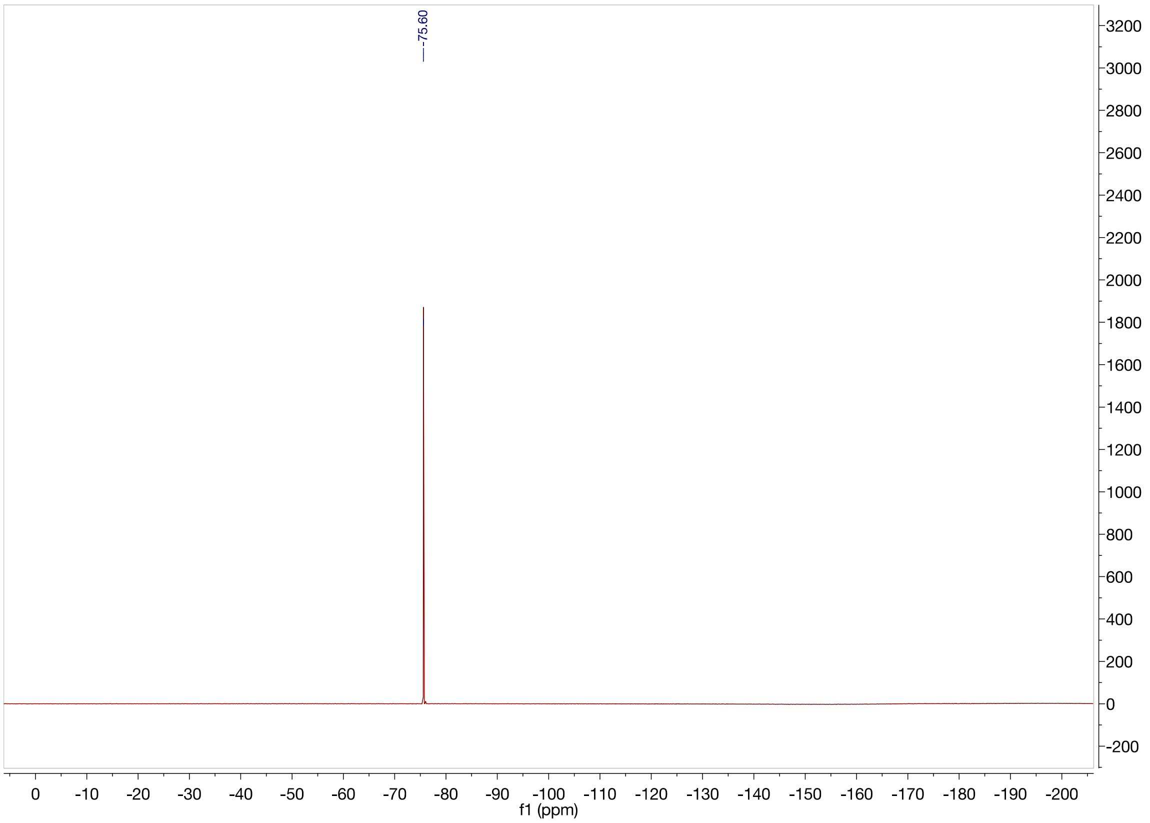

^{19}F {^{1}H} NMR (D_2O, 470.8 MHz) of (**1n**)

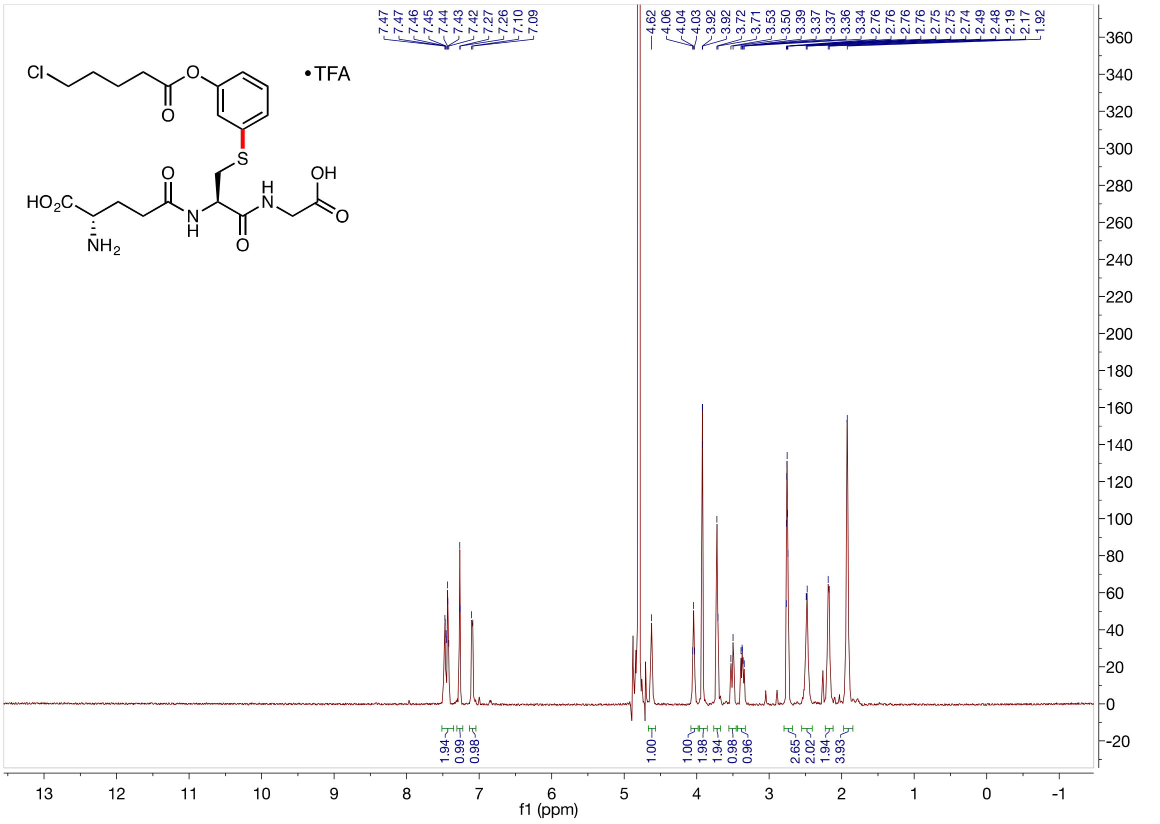

^{1}H NMR (D_2O, 500.4 MHz) of (**1o**)

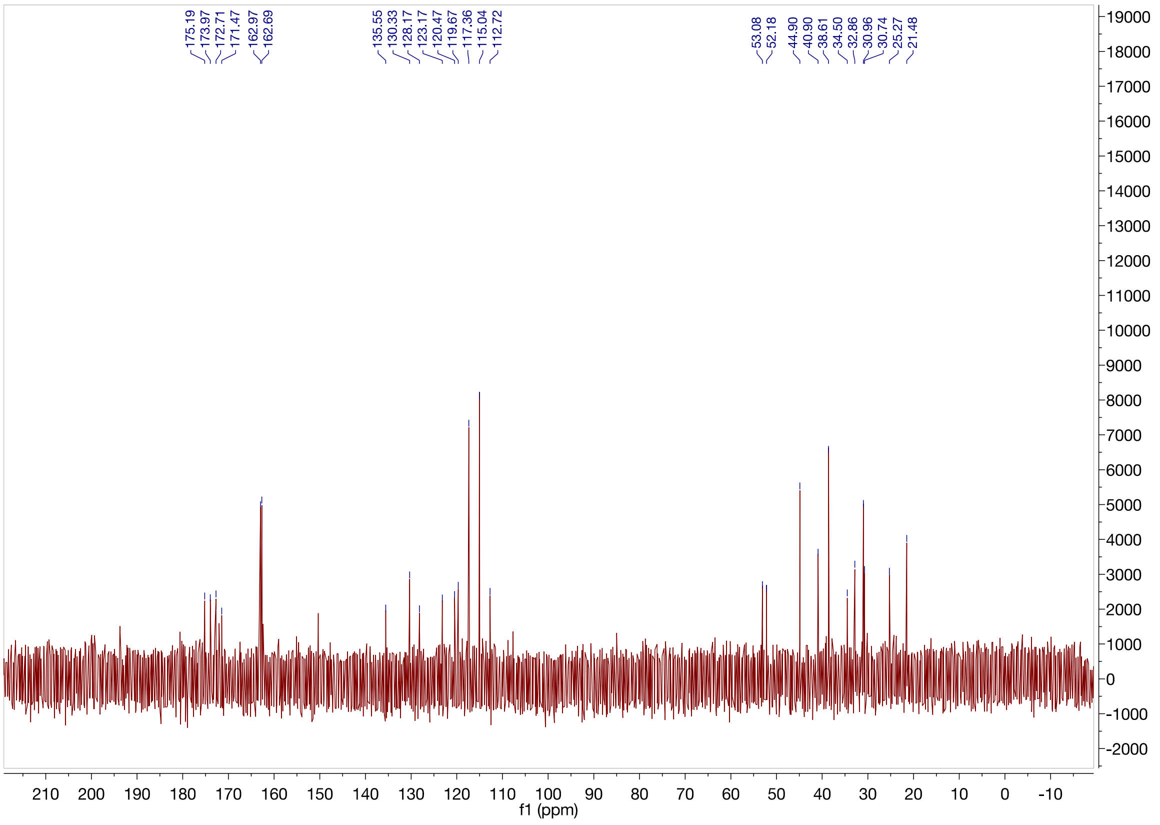

^{13}C {^{1}H} NMR (D_2O, 125.8 MHz) of (**1o**)

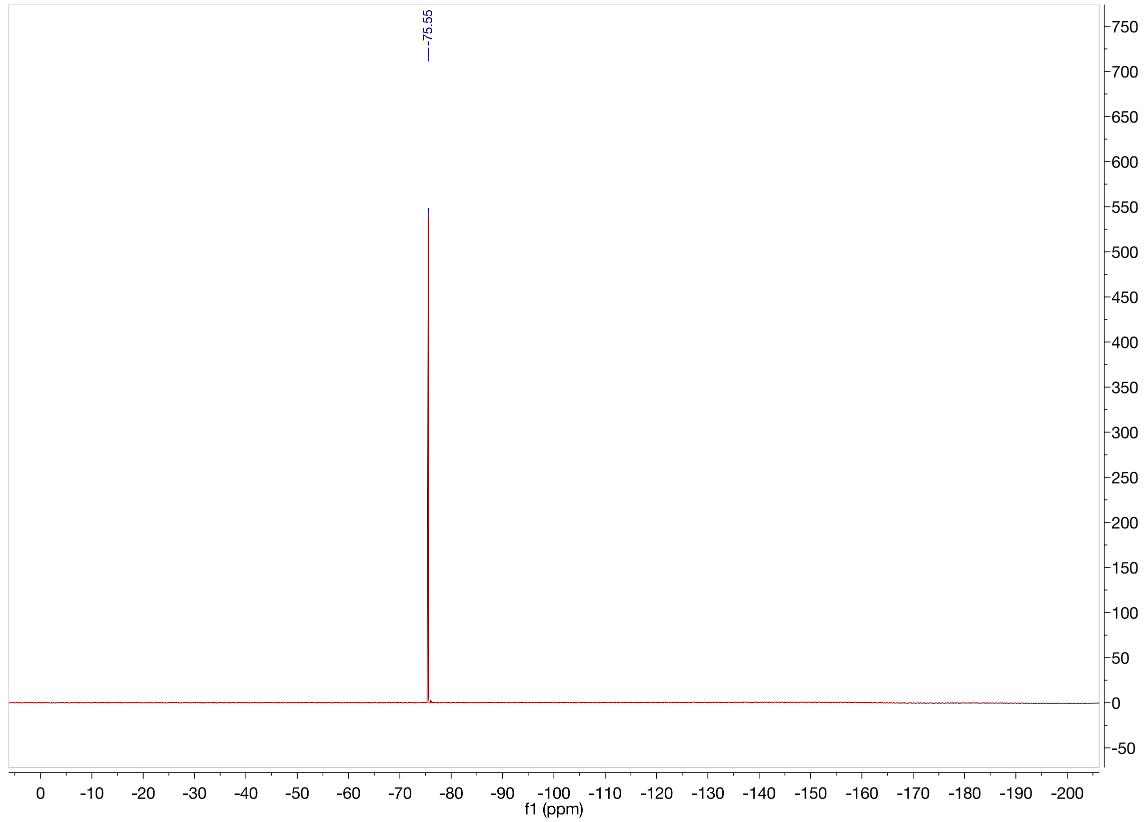

^{19}F {^{1}H} NMR (D_2O, 470.8 MHz) of (**1o**)

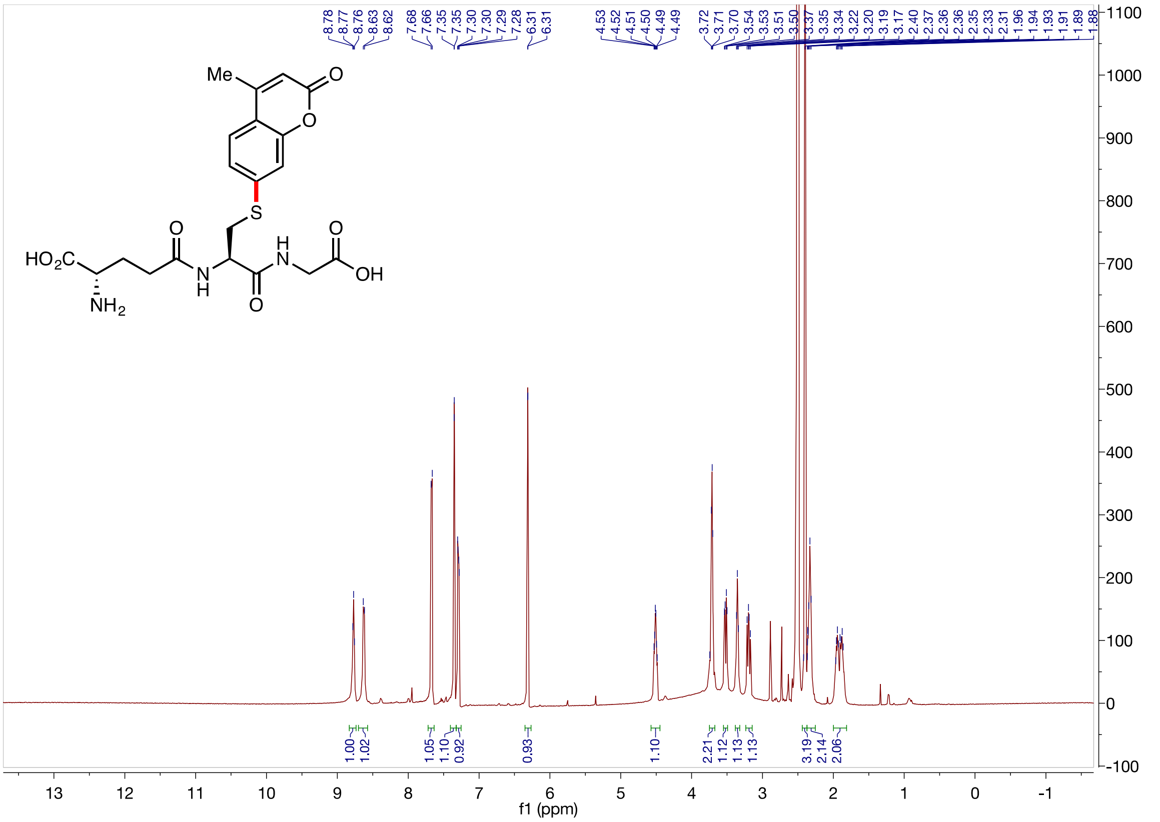

^{1}H NMR (DMSO-d_6, 500.4 MHz) of (**1p**)

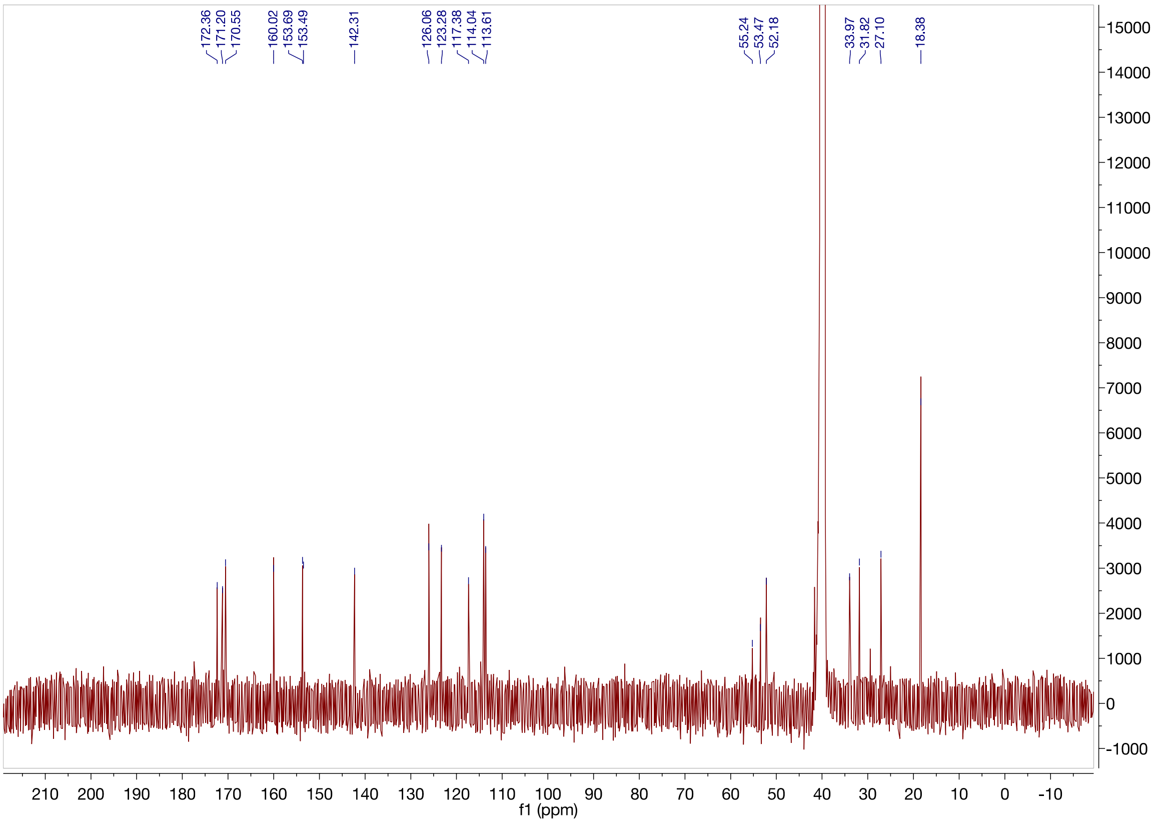

^{13}C {^{1}H} NMR (DMSO-d_6, 125.8 MHz) of (**1p**)

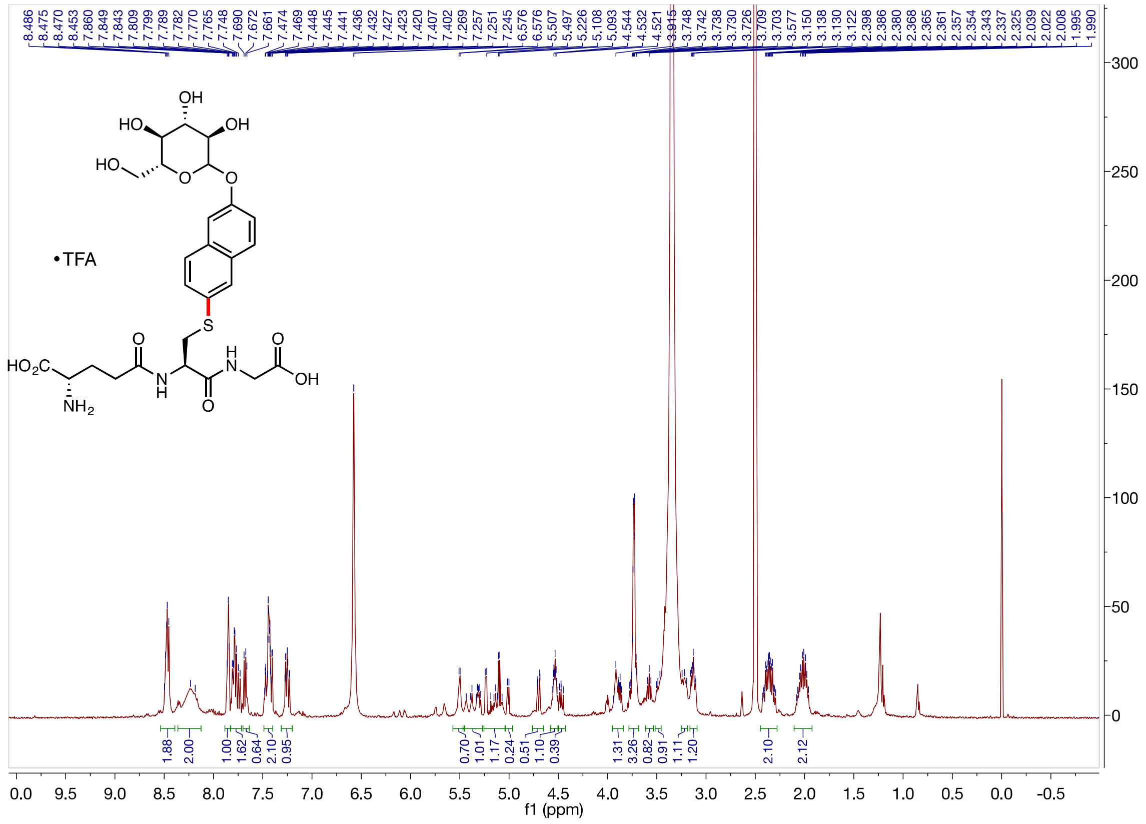

^{1}H NMR (DMSO-d_6, 500.4 MHz) of (**1r**)

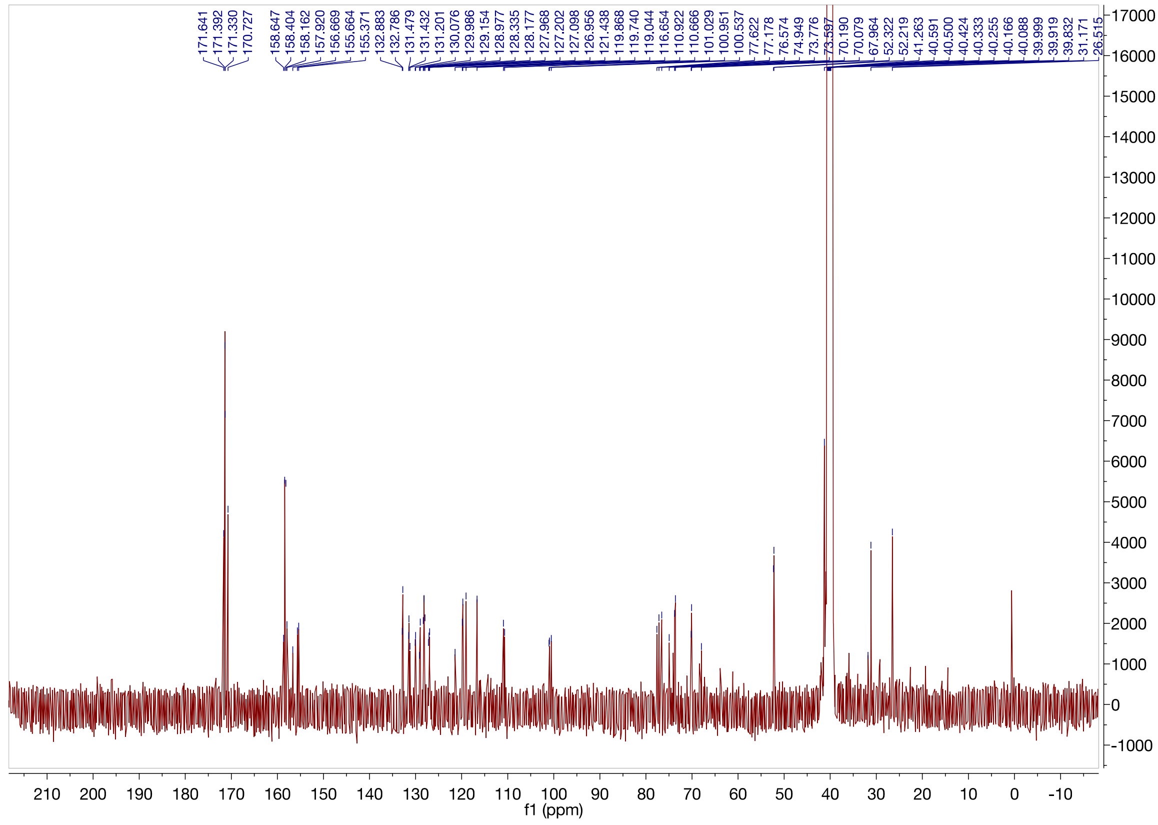

^{13}C {^{1}H} NMR (DMSO-d_6, 125.8 MHz) of (**1r**)

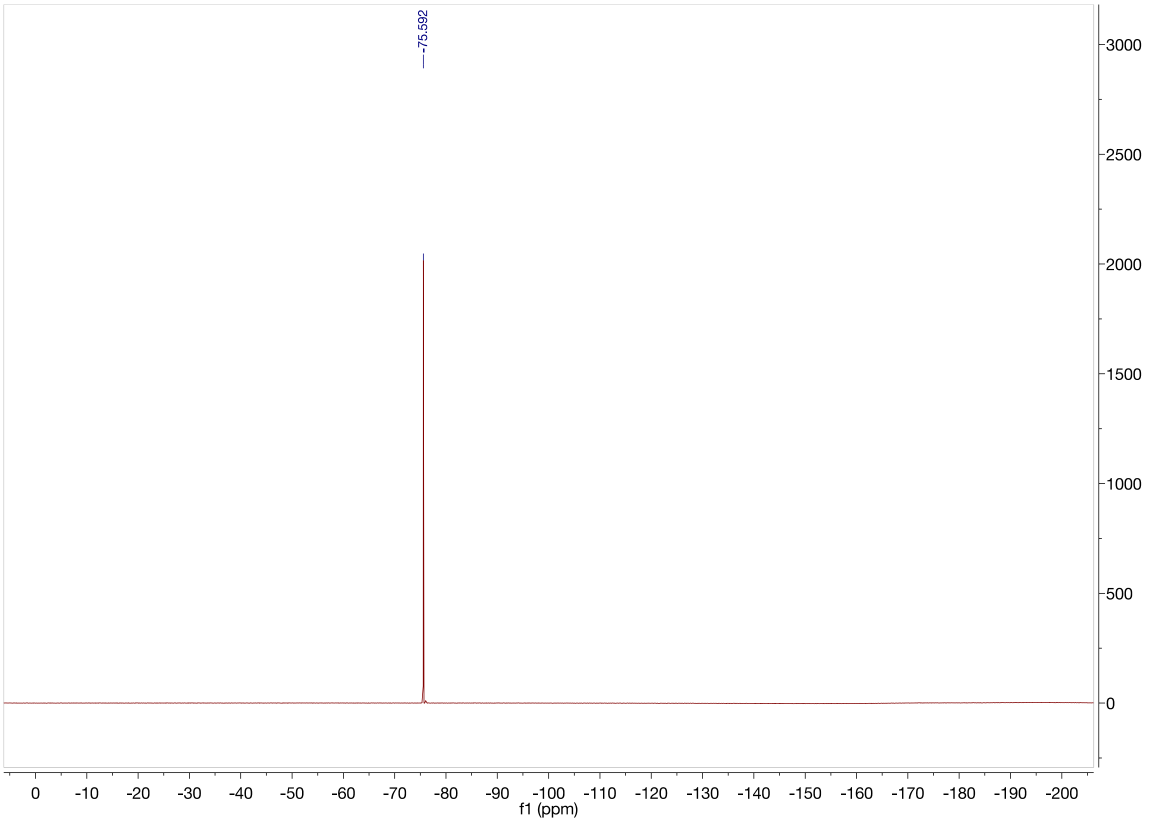

^{19}F {^{1}H} NMR (DMSO-d_6, 470.8 MHz) of (**1r**)

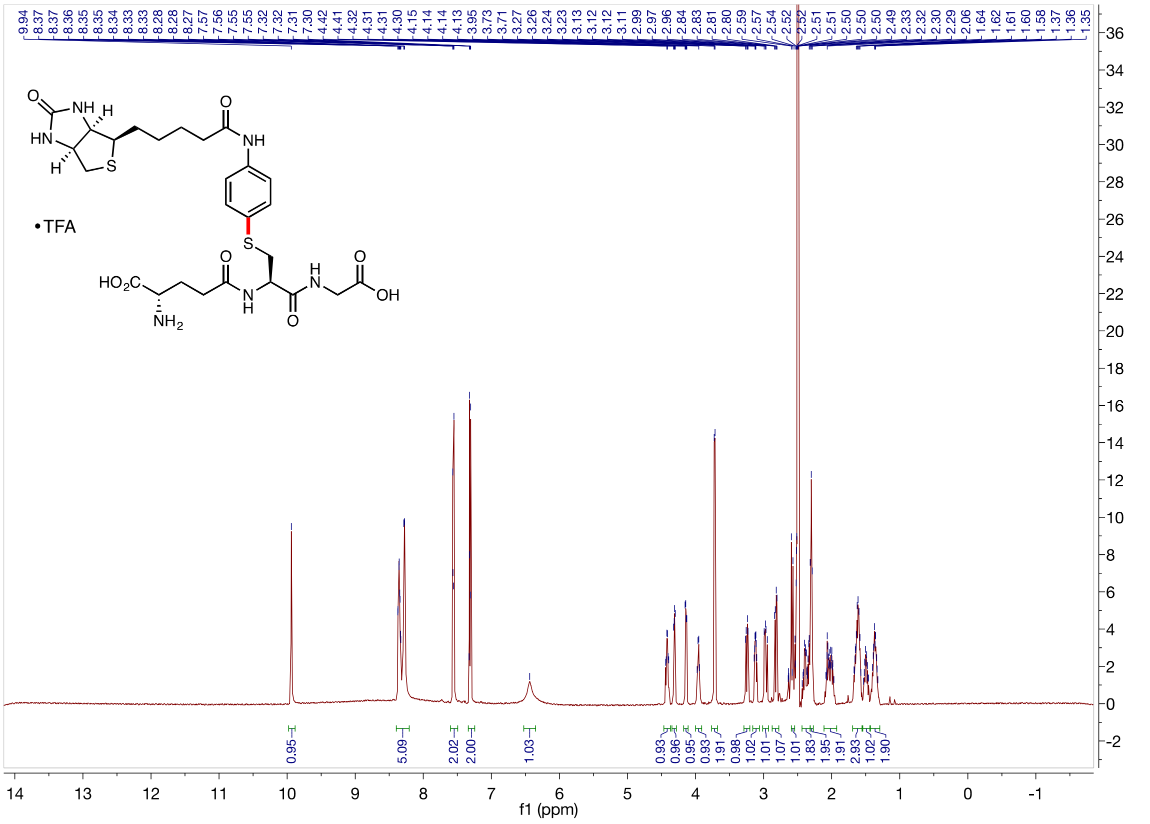

^{1}H NMR (DMSO-d_6, 500.4 MHz) of (**1q**)

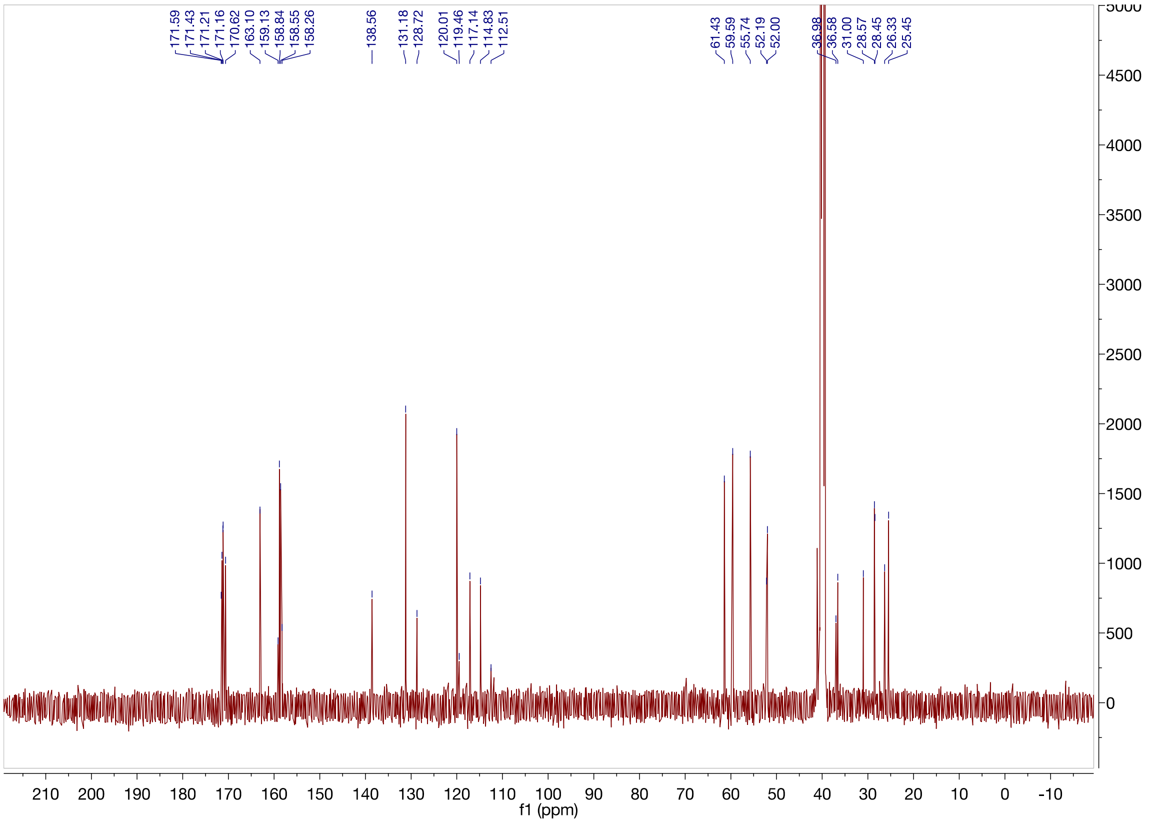

$^{13}C\ \{^{1}H\}$ NMR (DMSO-d_6, 125.8 MHz) of (**1q**)

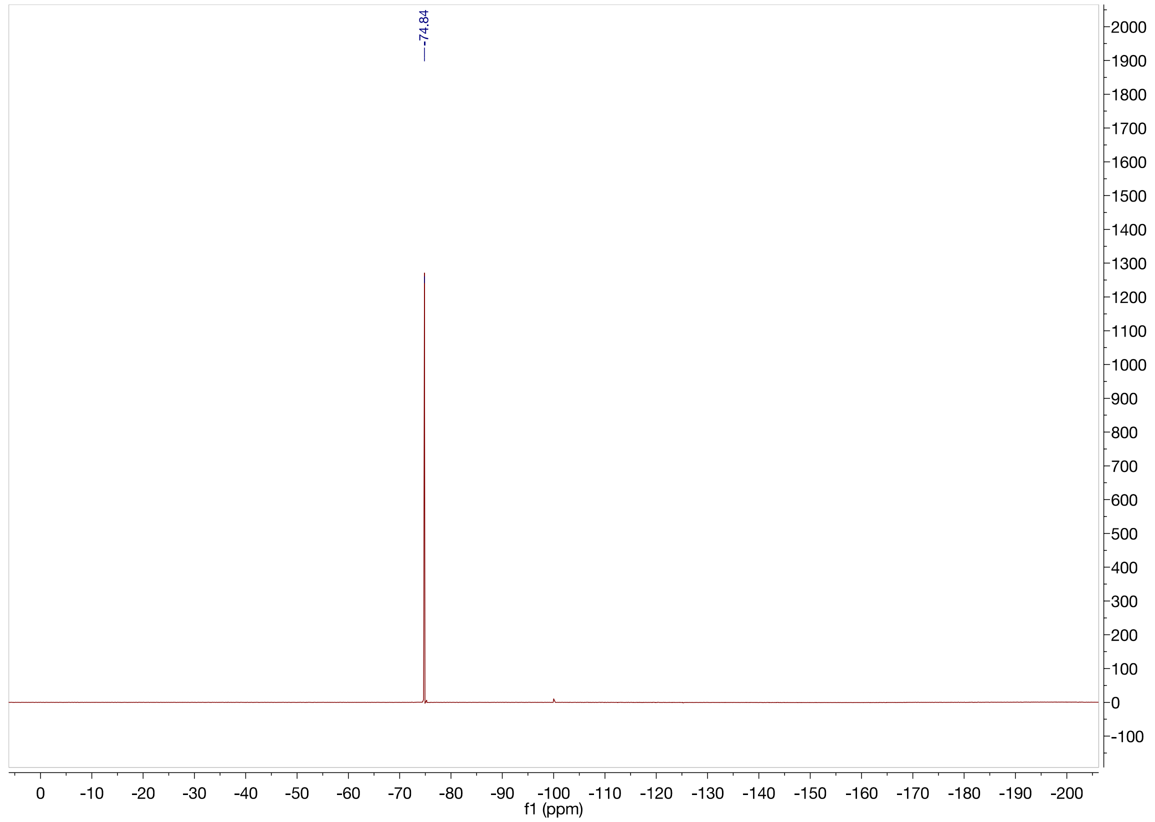

^{19}F {^{1}H} NMR (DMSO-d_6, 470.8 MHz) of (**1q**)

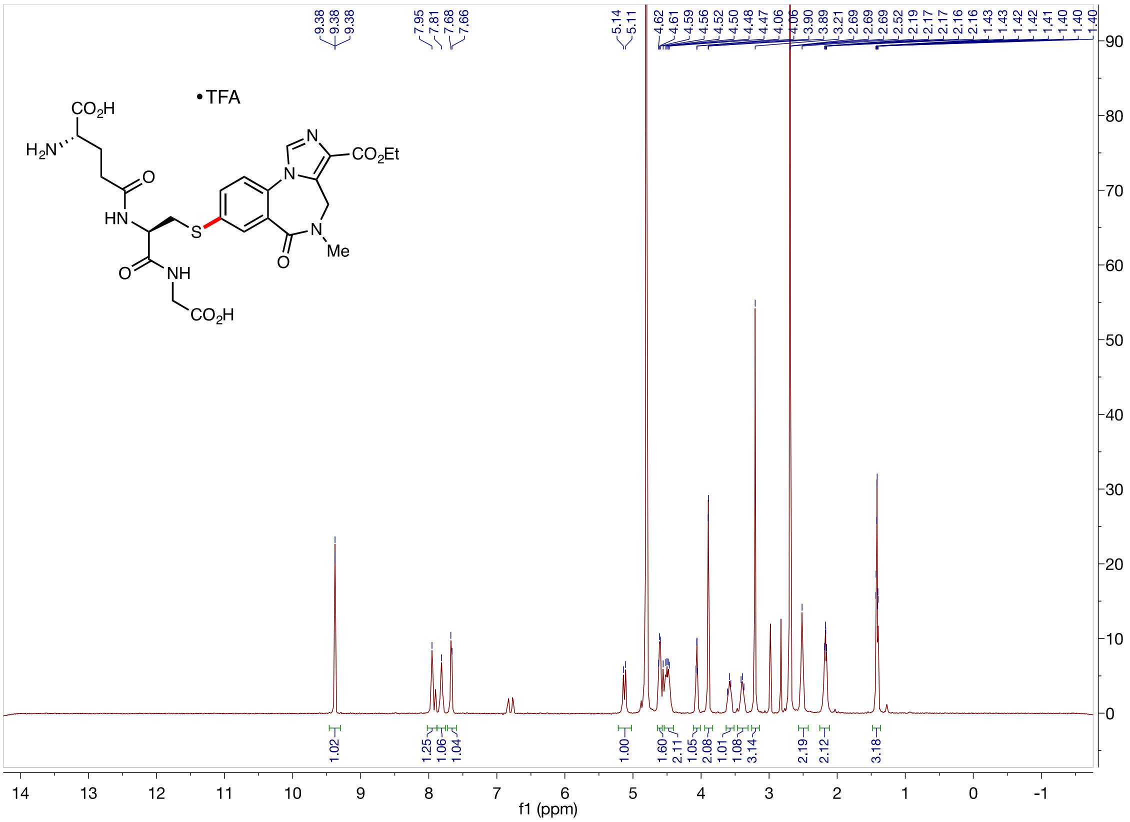

^{1}H NMR (DMSO-d_6, 500.4 MHz) of (**1s**)

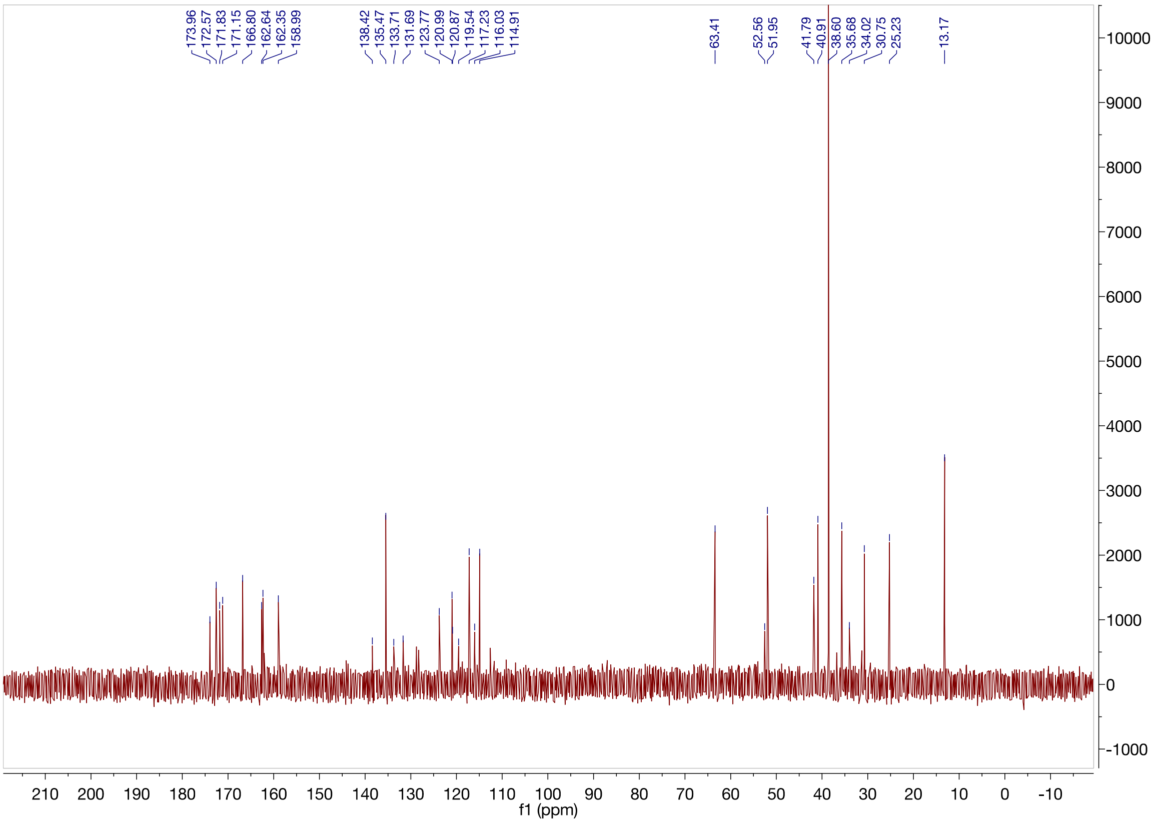

^{13}C {^{1}H} NMR (DMSO-d_6, 125.8 MHz) of (**1s**)

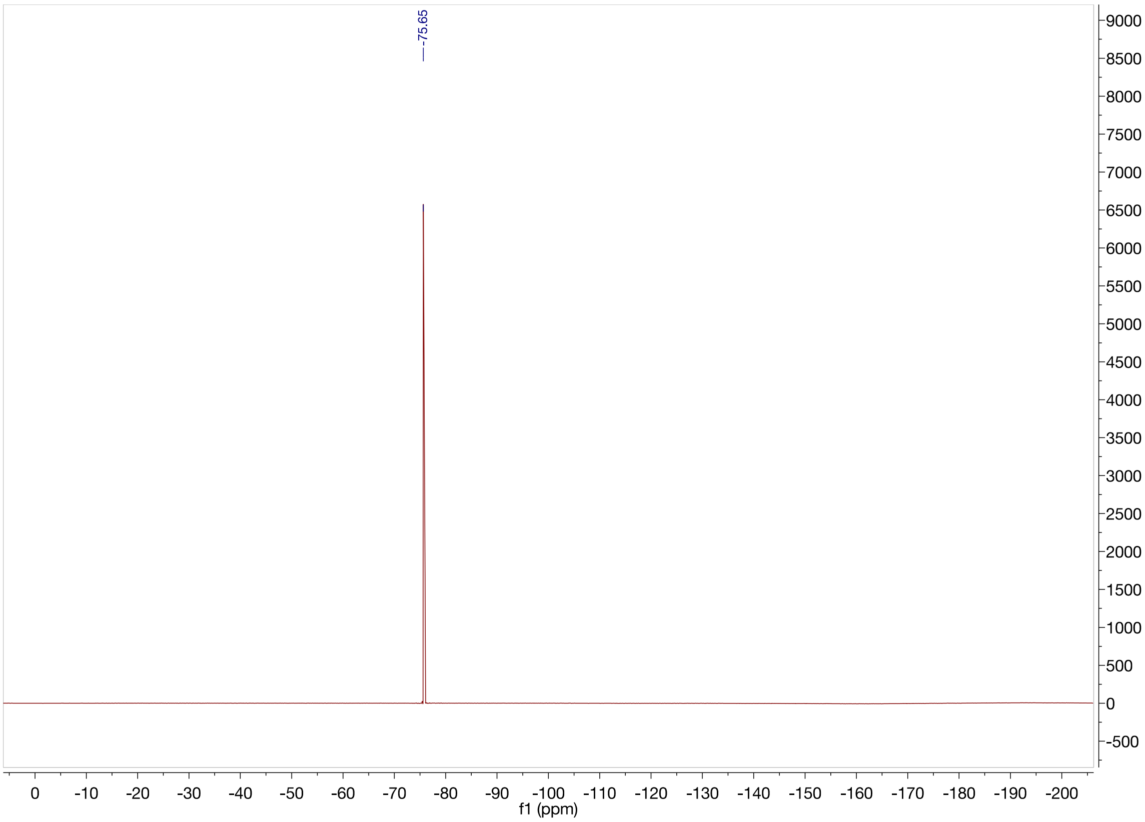

^{19}F {^{1}H} NMR (DMSO-d_6, 470.8 MHz) of (**1s**)

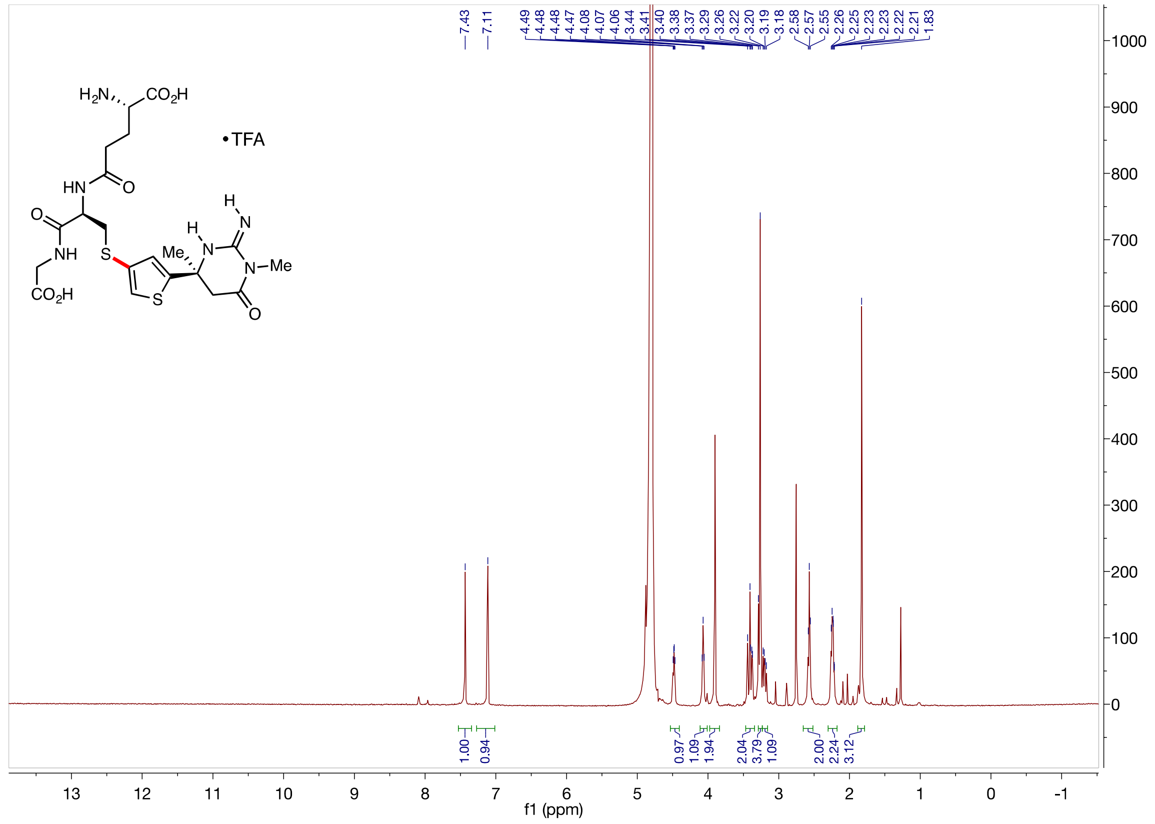

^{1}H NMR (D_2O, 500.4 MHz) of (**1t**)

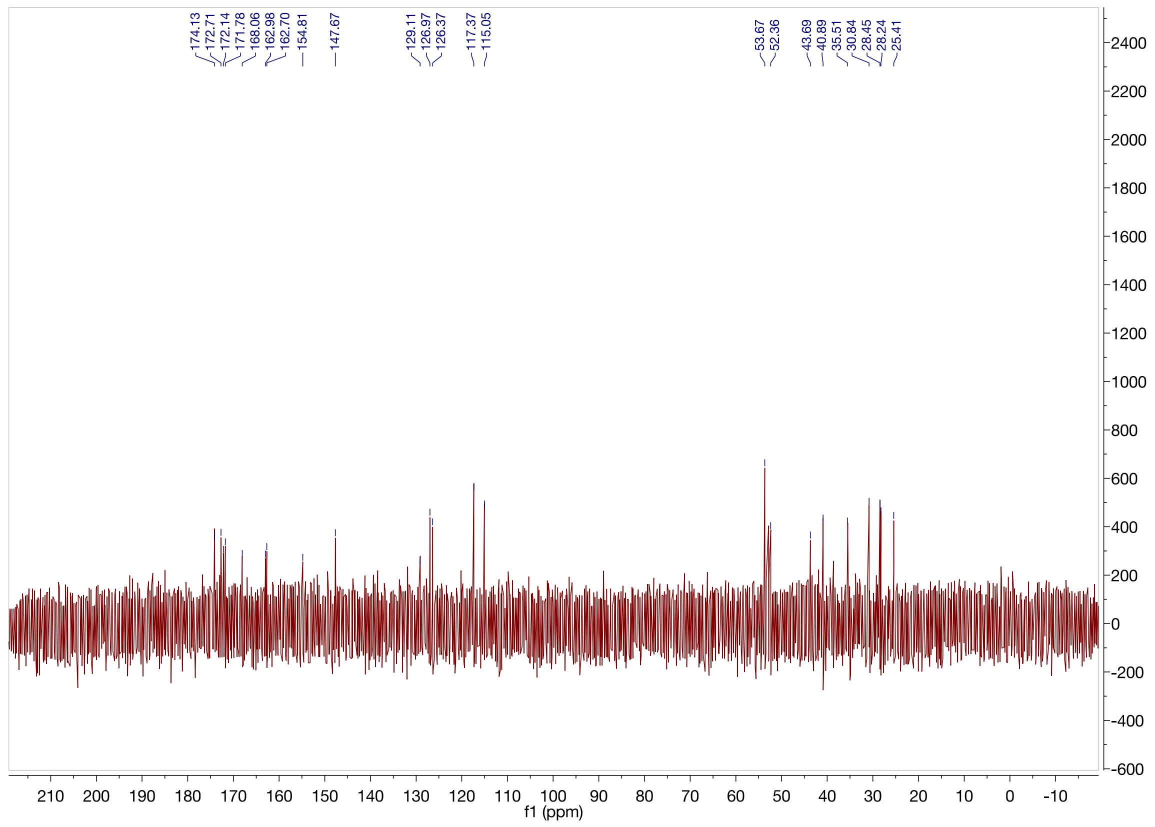

^{13}C {^{1}H} NMR (D_2O, 125.8 MHz) of (**1t**)

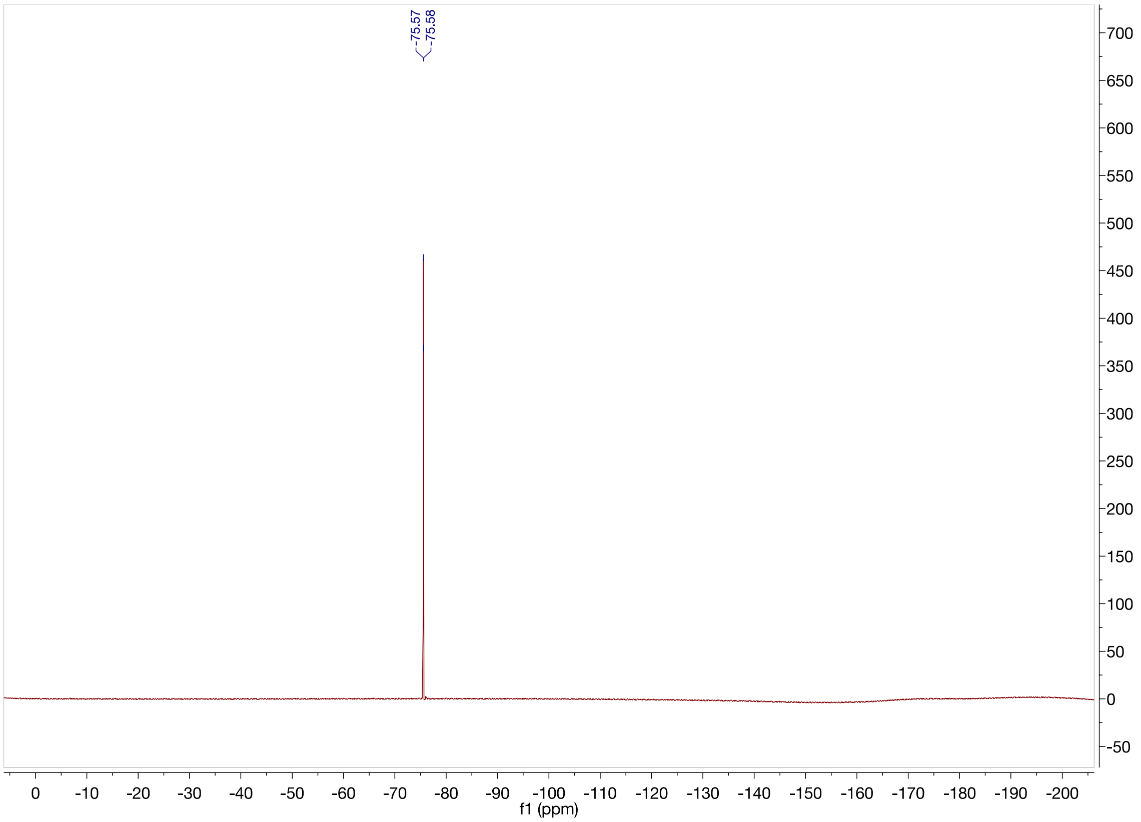

$^{19}F\{^{1}H\}$ NMR (D_2O, 470.8 MHz) of (**1t**)

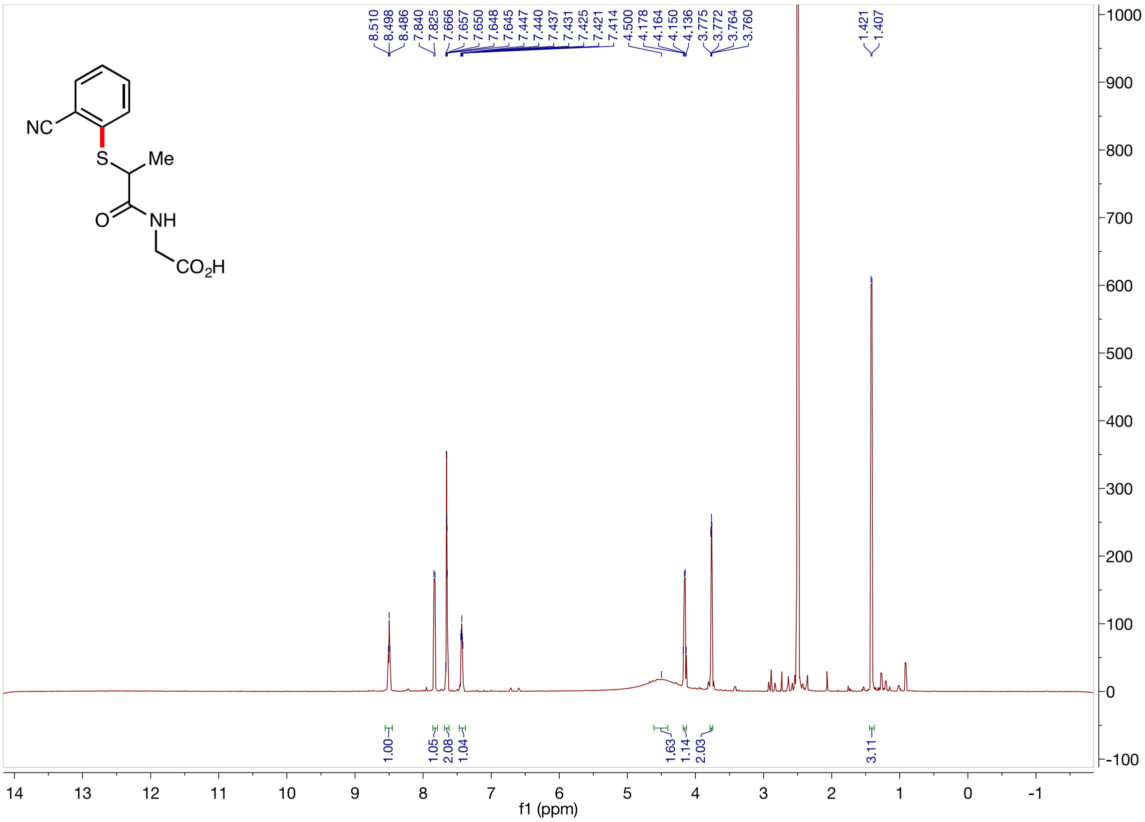

^{1}H NMR (DMSO-d_6, 500.4 MHz) of (**7a**)

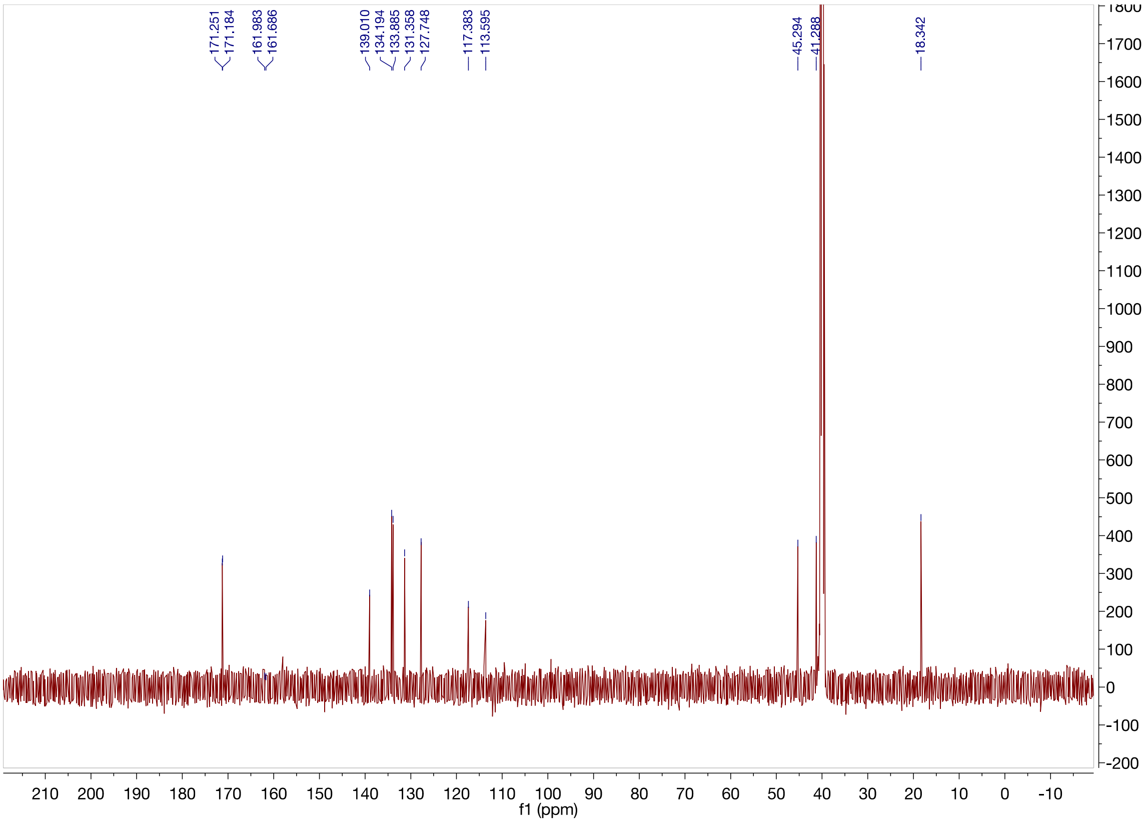

^{13}C {^{1}H} NMR (DMSO-d_6, 125.8 MHz) of (**7a**)

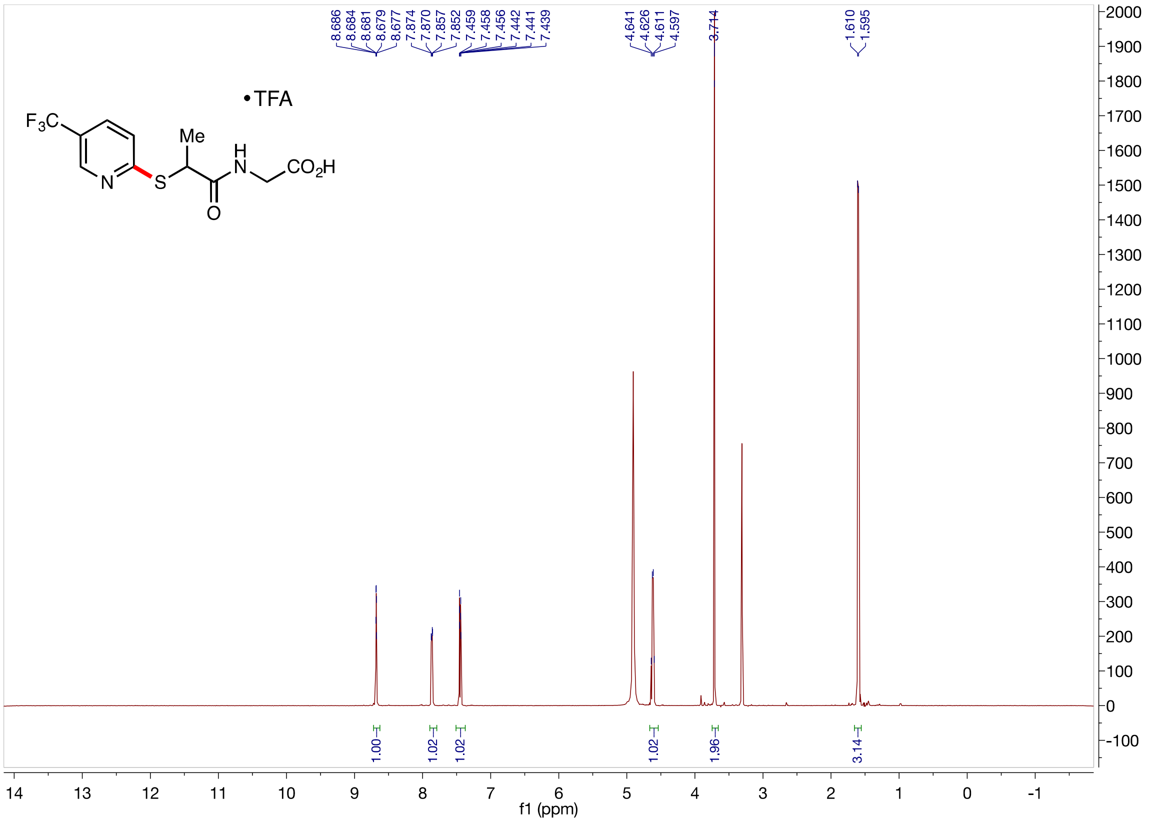

^{1}H NMR (CD_3OD, 500.4 MHz) of (**7b**)

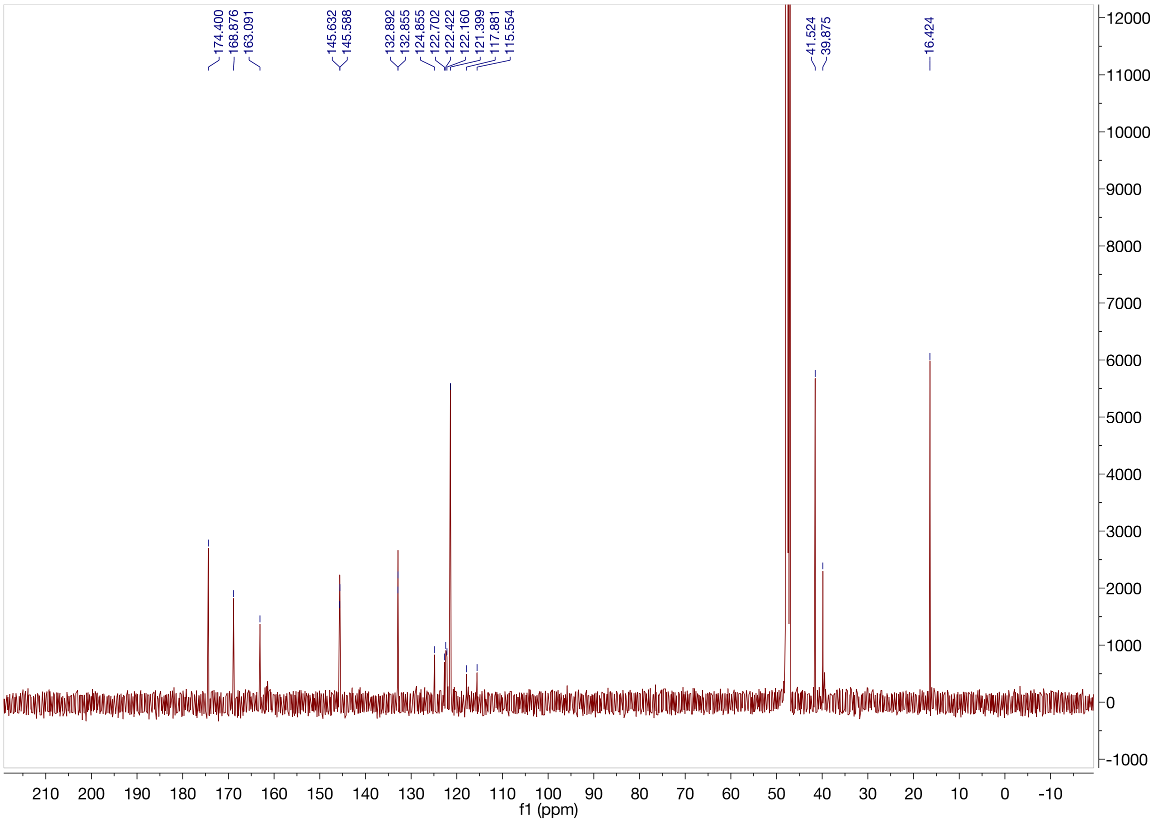

^{13}C {^{1}H} NMR (CD_3OD, 125.8 MHz) of (**7b**)

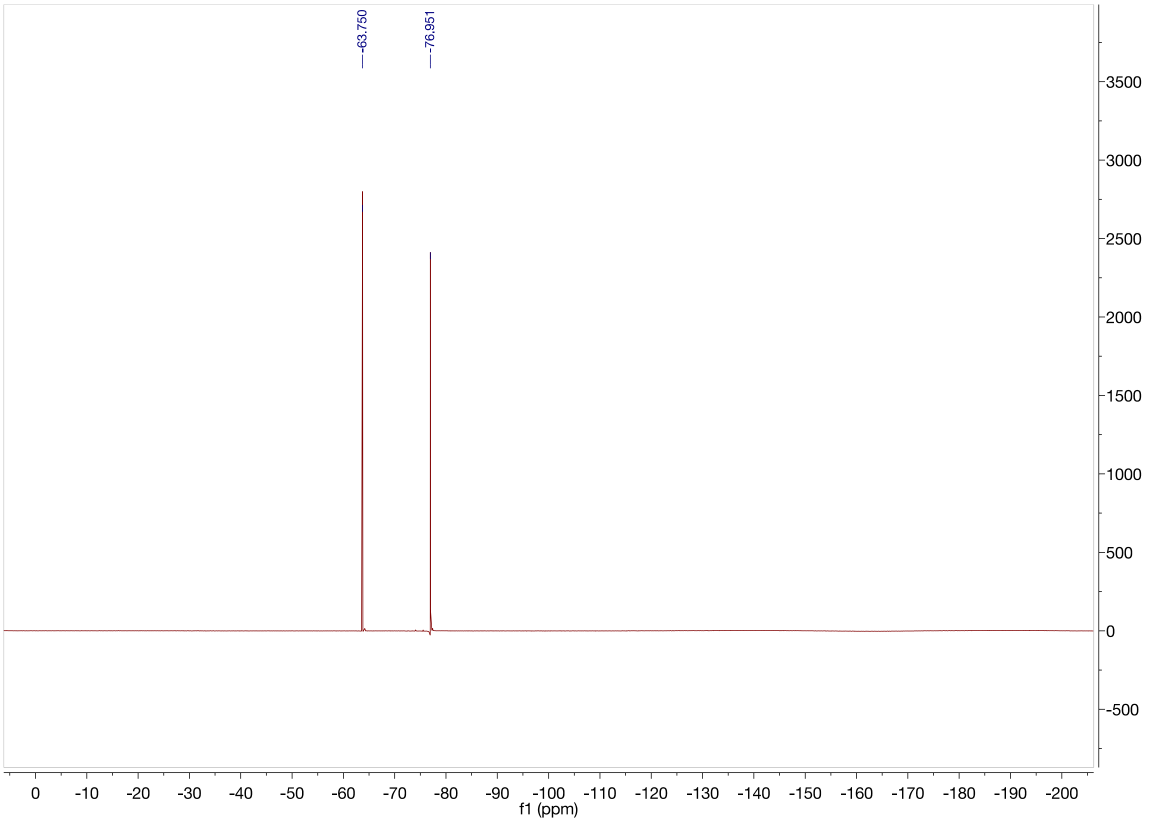

^{19}F {^{1}H} NMR (CD_3OD, 470.8 MHz) of (**7b**)

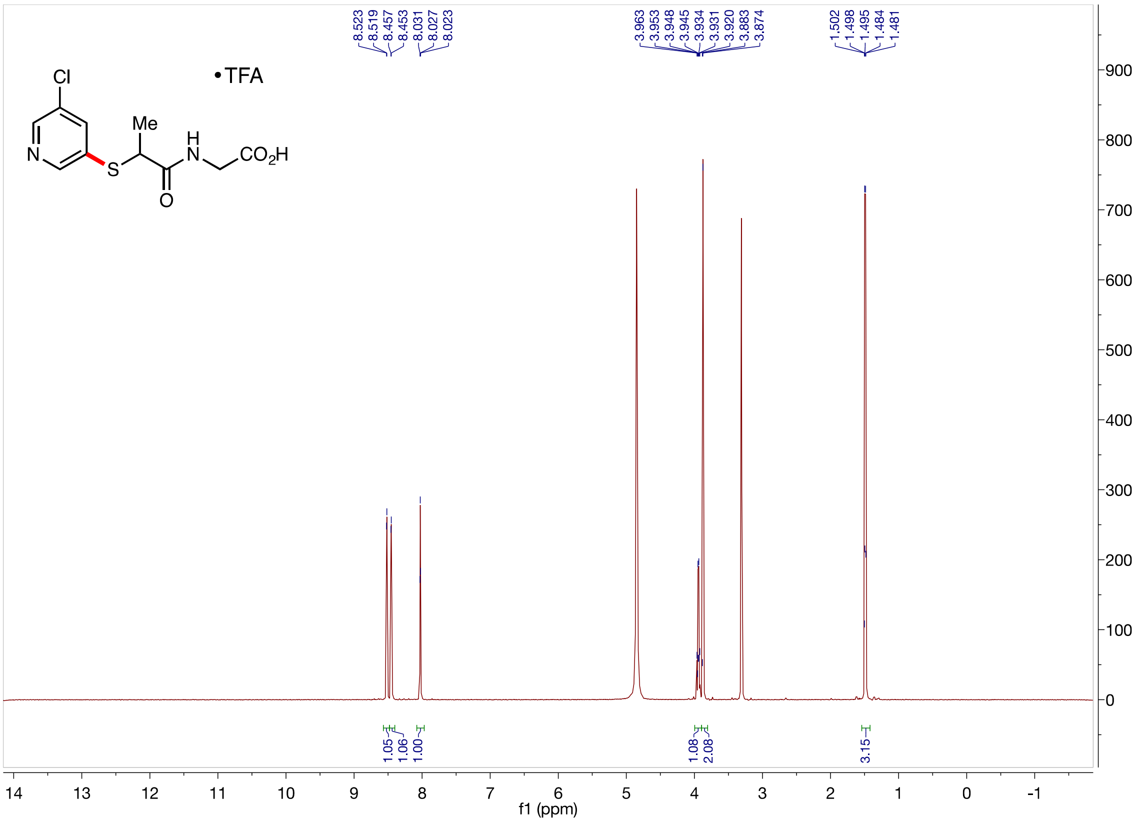

^{1}H NMR (CD$_3$OD, 500.4 MHz) of (**7c**)

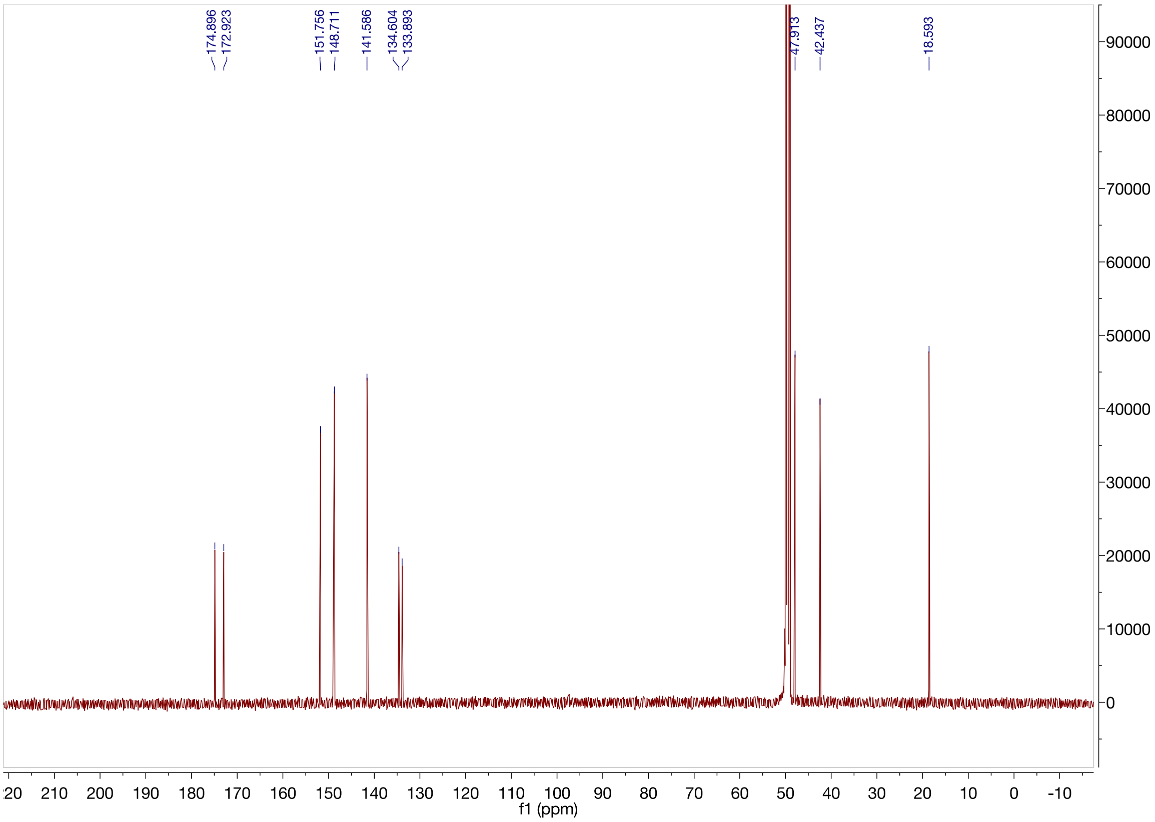

^{13}C {^{1}H} NMR (CD$_3$OD, 125.8 MHz) of (**7c**)

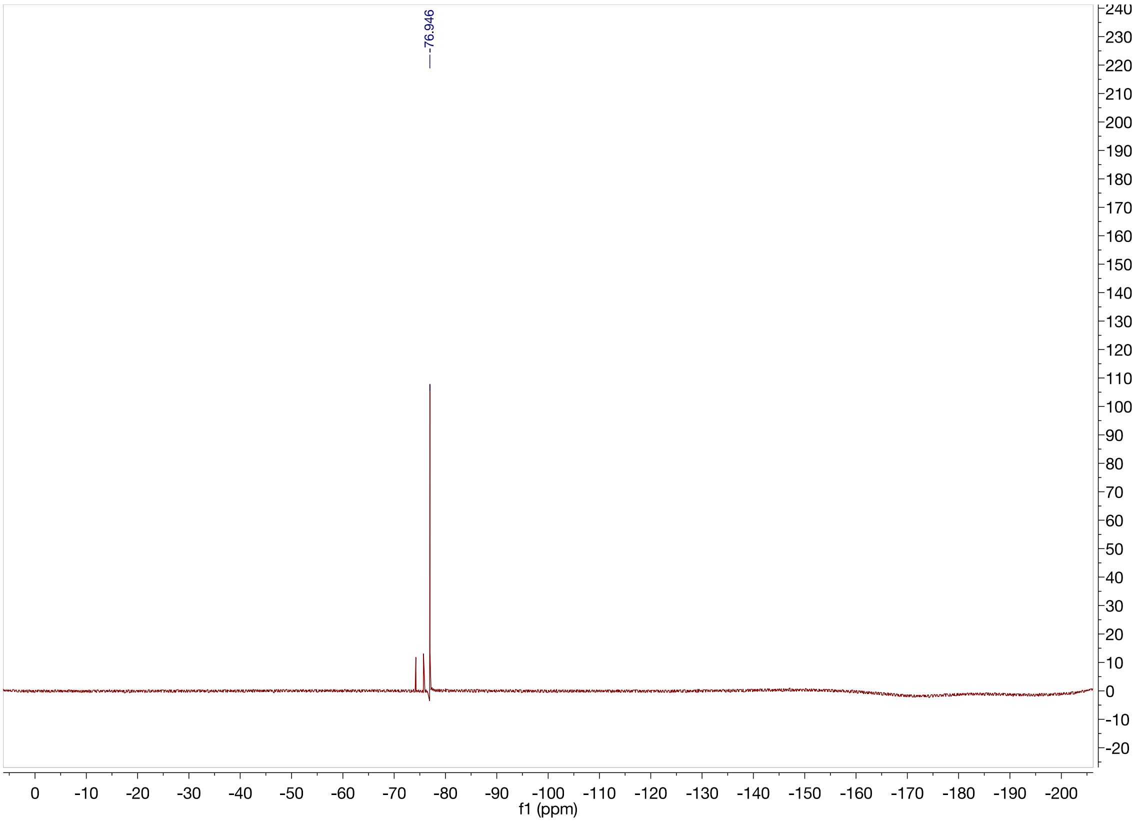

^{19}F {^{1}H} NMR (CD_3OD, 470.8 MHz) of (**7c**)

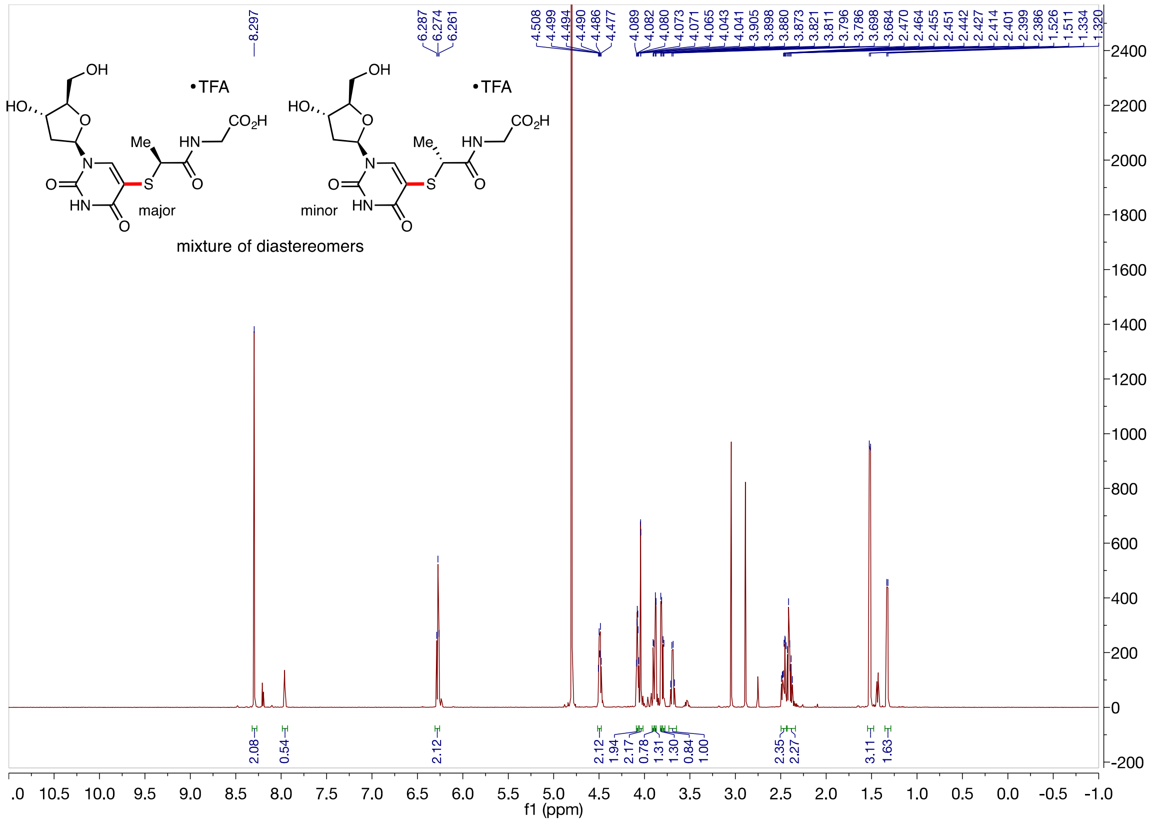

^{1}H NMR (D_2O, 500.4 MHz) of (**7d**)

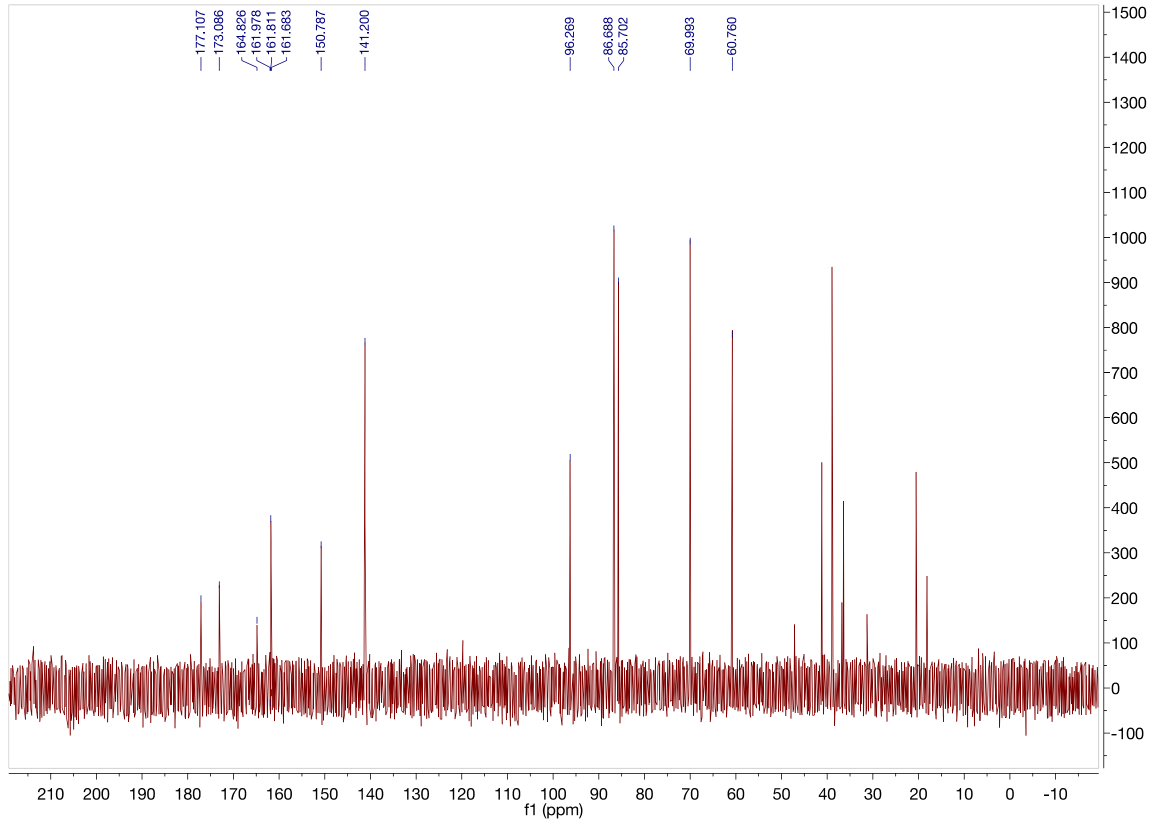

^{13}C {^{1}H} NMR (D_2O, 125.8 MHz) of (**7d**)

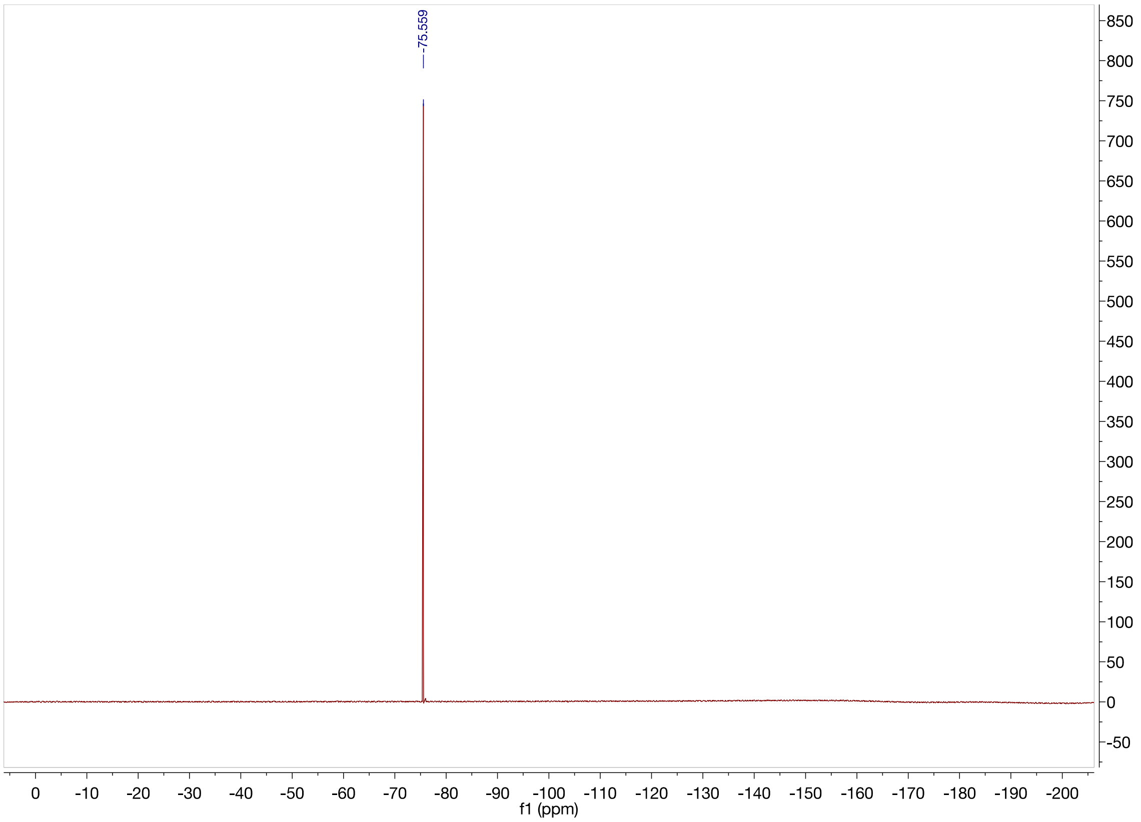

^{19}F {^{1}H} NMR (D_2O, 470.8 MHz) of (**7d**)

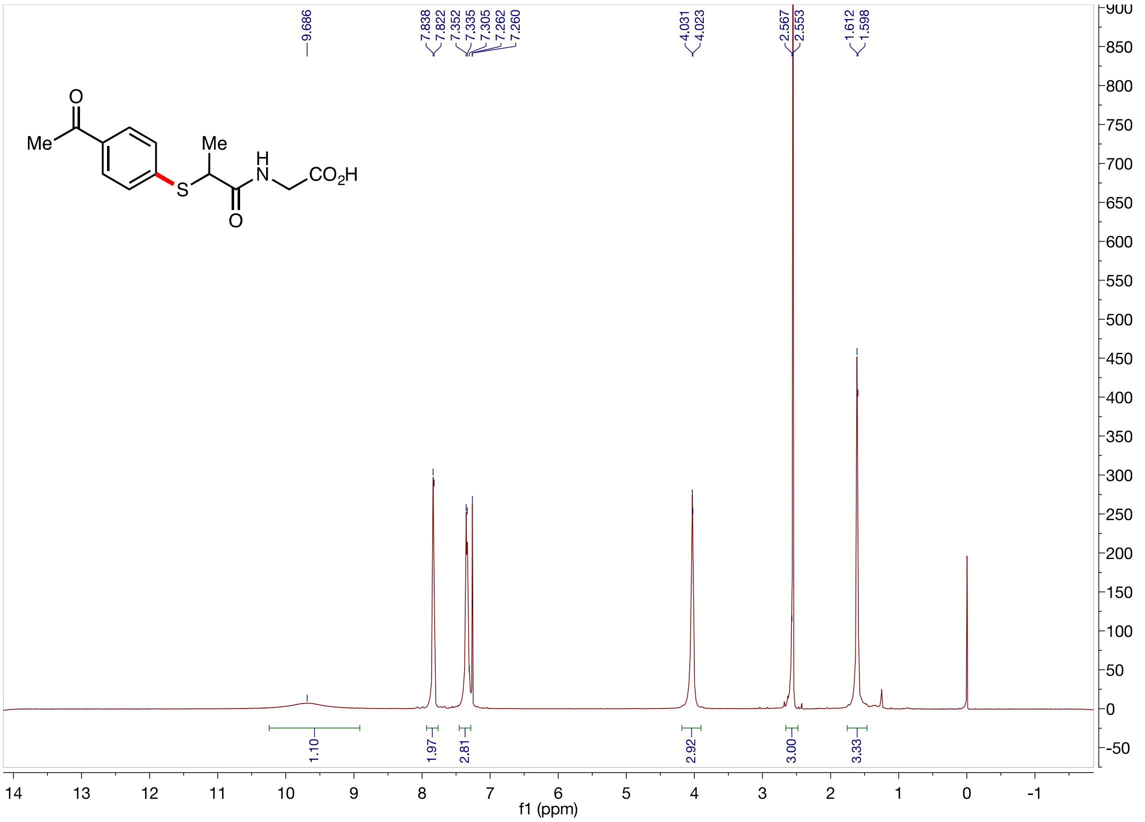

^{1}H NMR ($CDCl_3$, 500.4 MHz) of (**7e**)

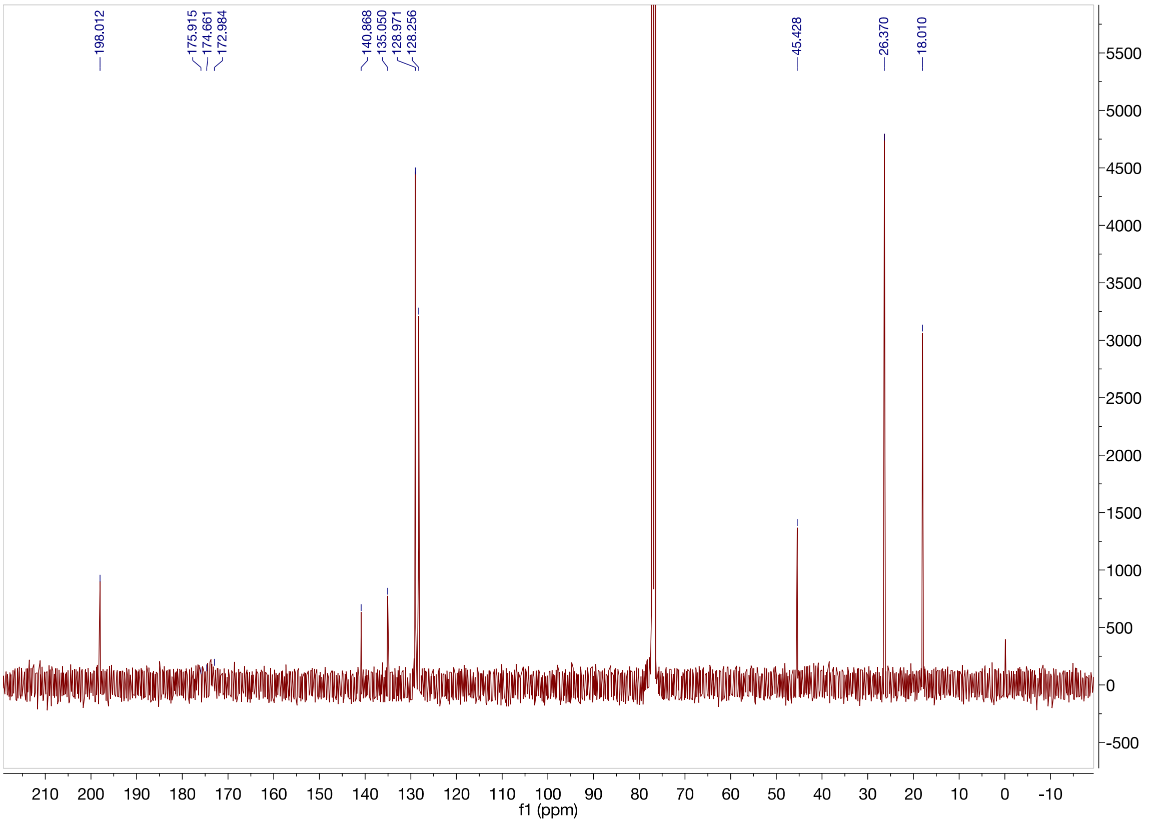

^{13}C {^{1}H} NMR ($CDCl_3$, 125.8 MHz) of (**7e**)

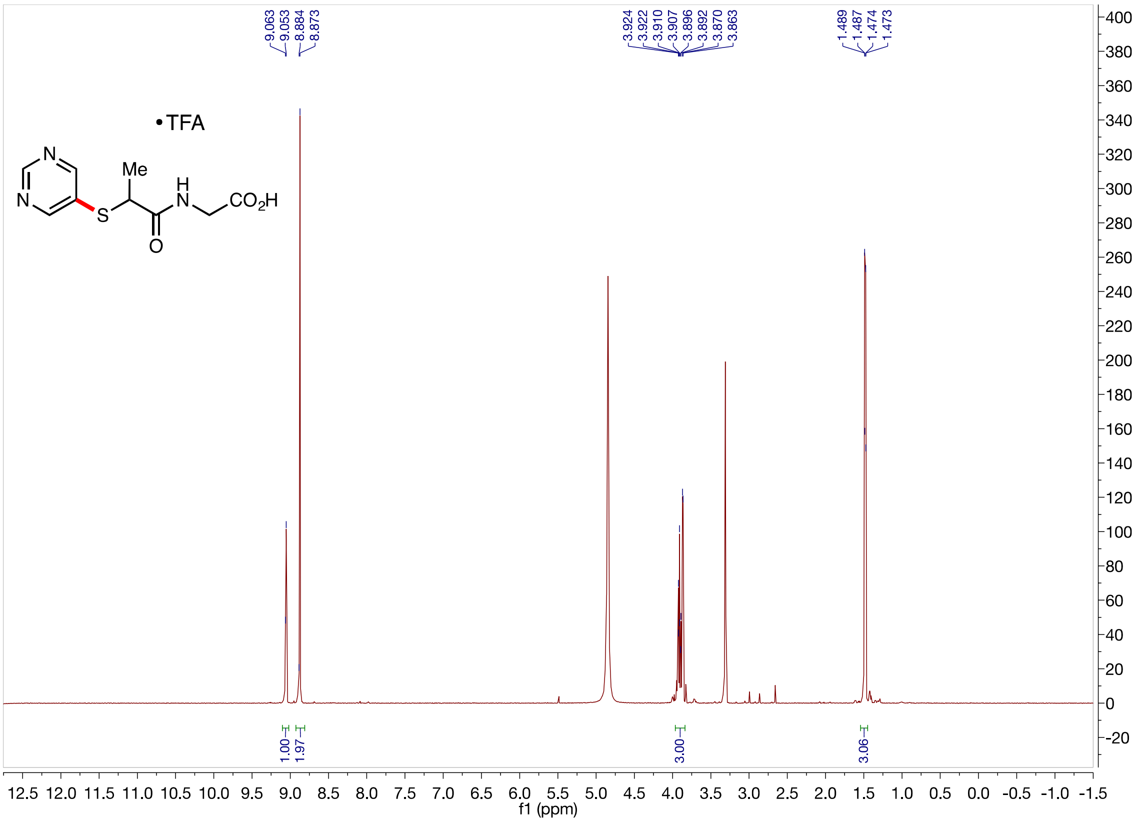

^{1}H NMR (CD_3OD, 500.4 MHz) of (**7f**)

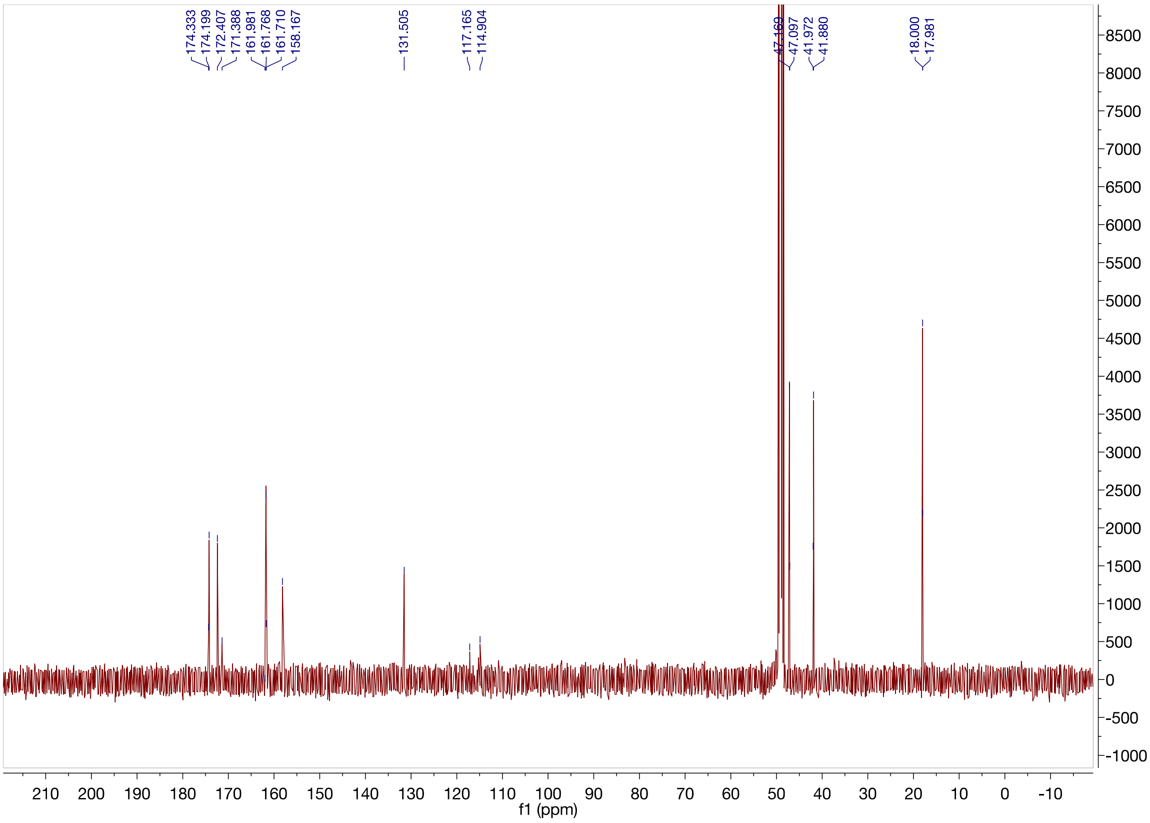

^{13}C {^{1}H} NMR (CD_3OD, 125.8 MHz) of (**7f**)

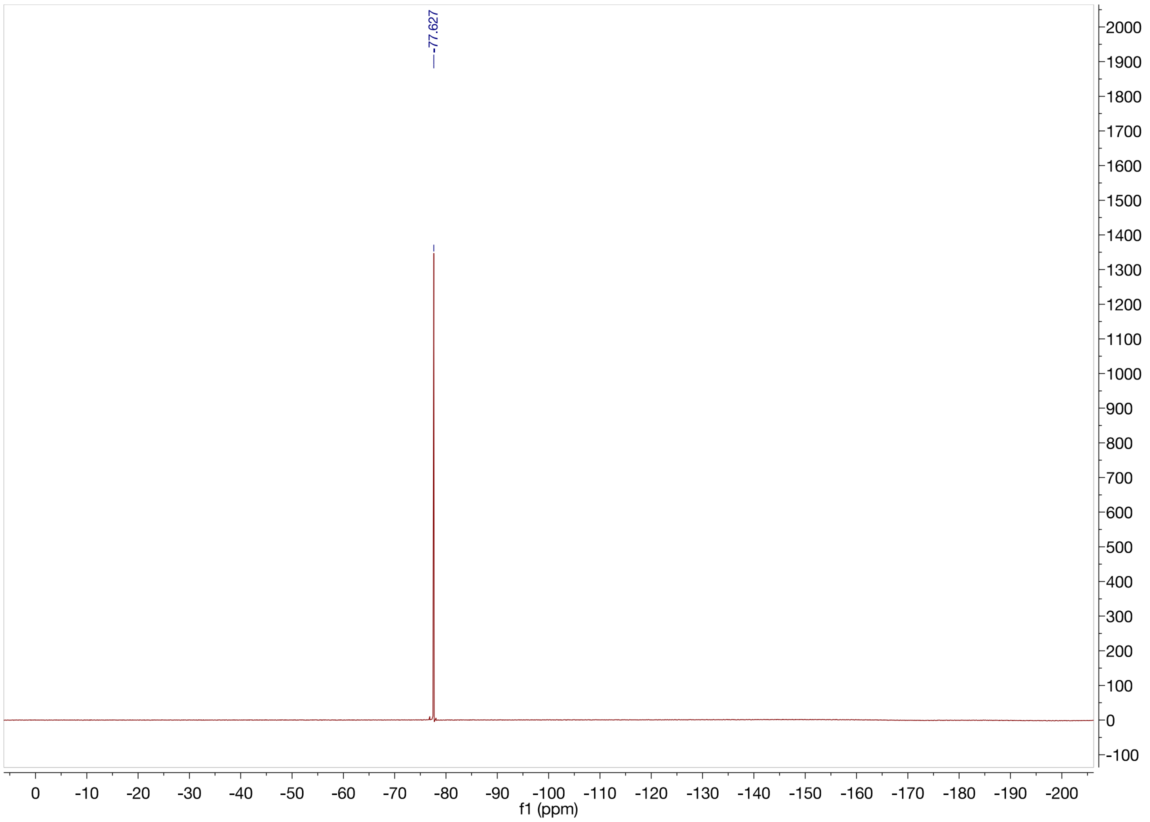

^{19}F {^{1}H} NMR (CD_3OD, 470.8 MHz) of (**7f**)

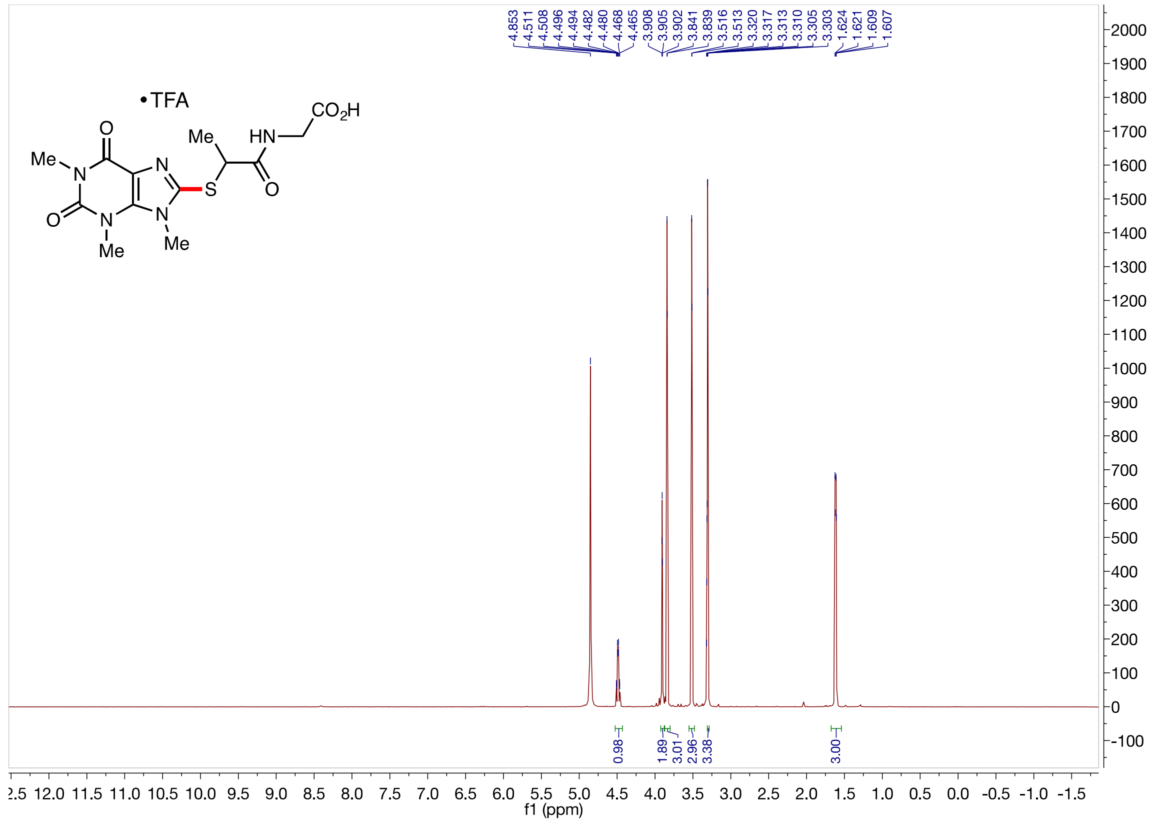

^{1}H NMR (CD$_3$OD, 500.4 MHz) of (**7g**)

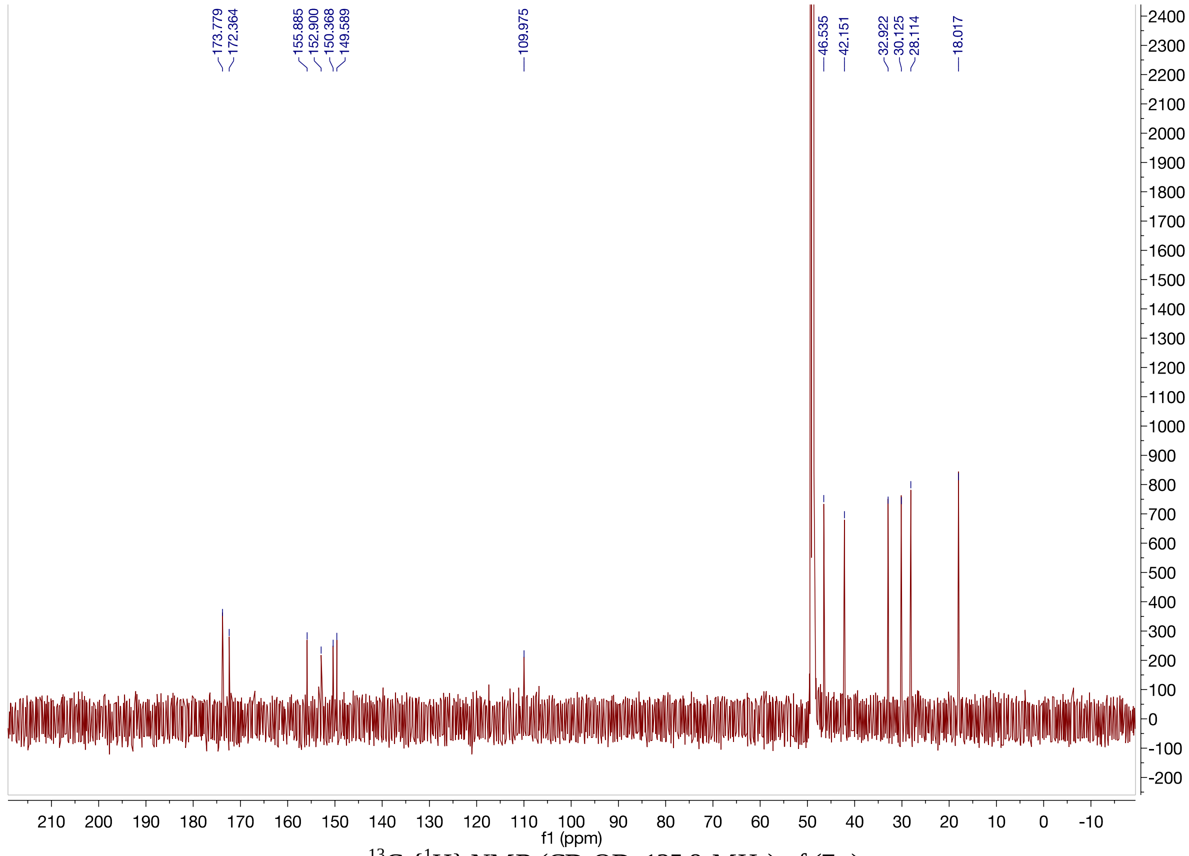

^{13}C {^{1}H} NMR (CD$_3$OD, 125.8 MHz) of (**7g**)

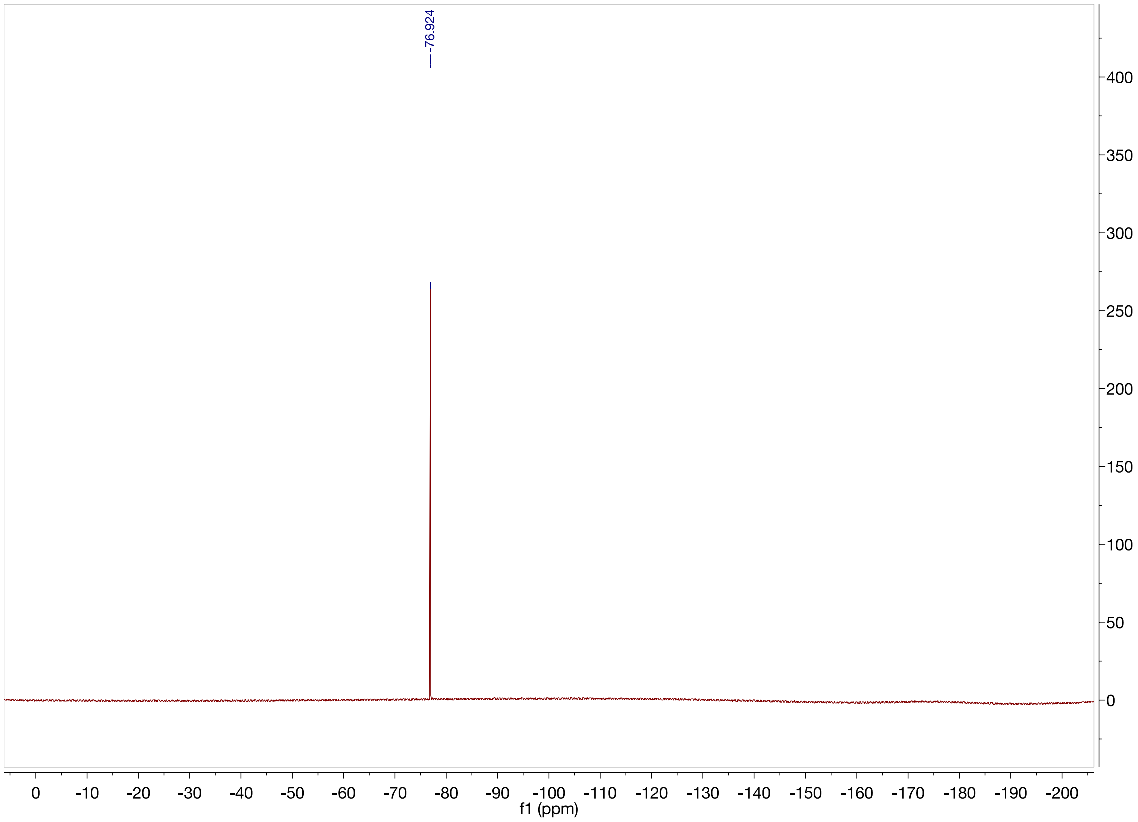

^{19}F {^{1}H} NMR (CD_3OD, 470.8 MHz) of (**7g**)

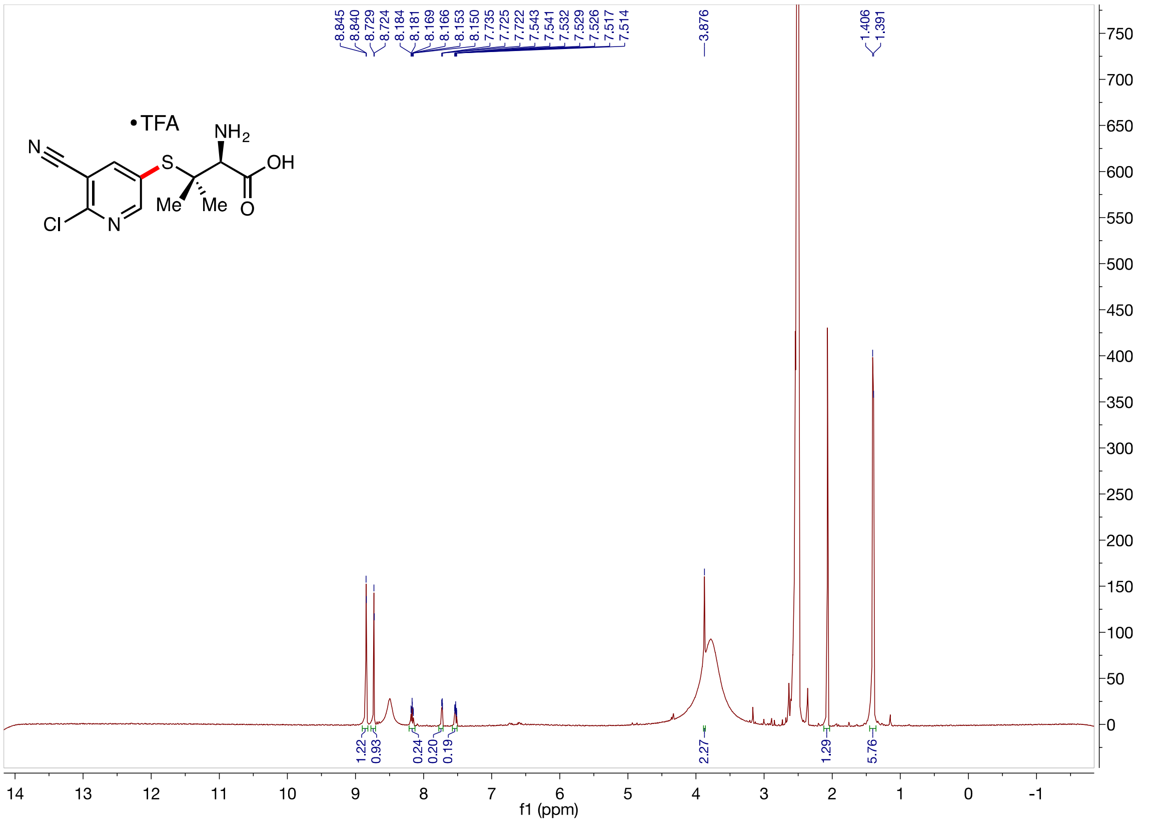

^{1}H NMR (DMSO-d_6, 500.4 MHz) of (**8a**)

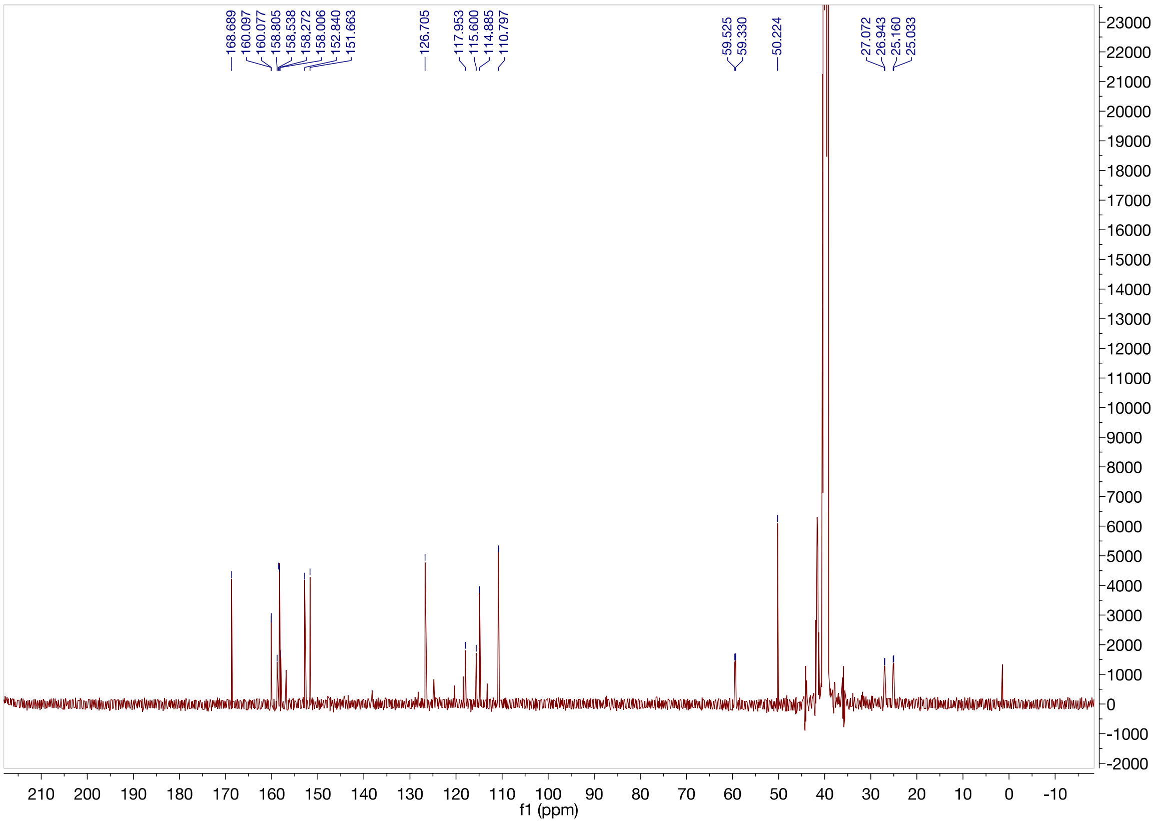

^{13}C {^{1}H} NMR (DMSO-d_6, 125.8 MHz) of (**8a**)

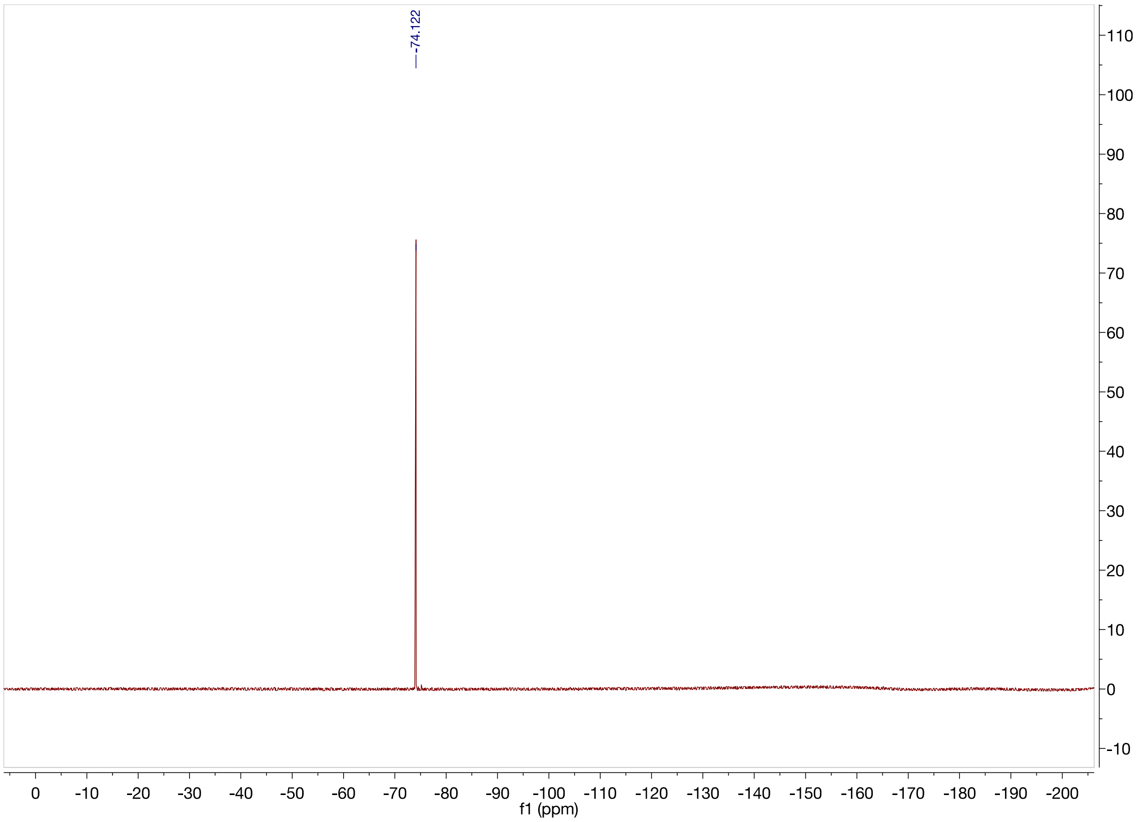

^{19}F {^{1}H} NMR (DMSO-d_6, 470.8 MHz) of (**8a**)

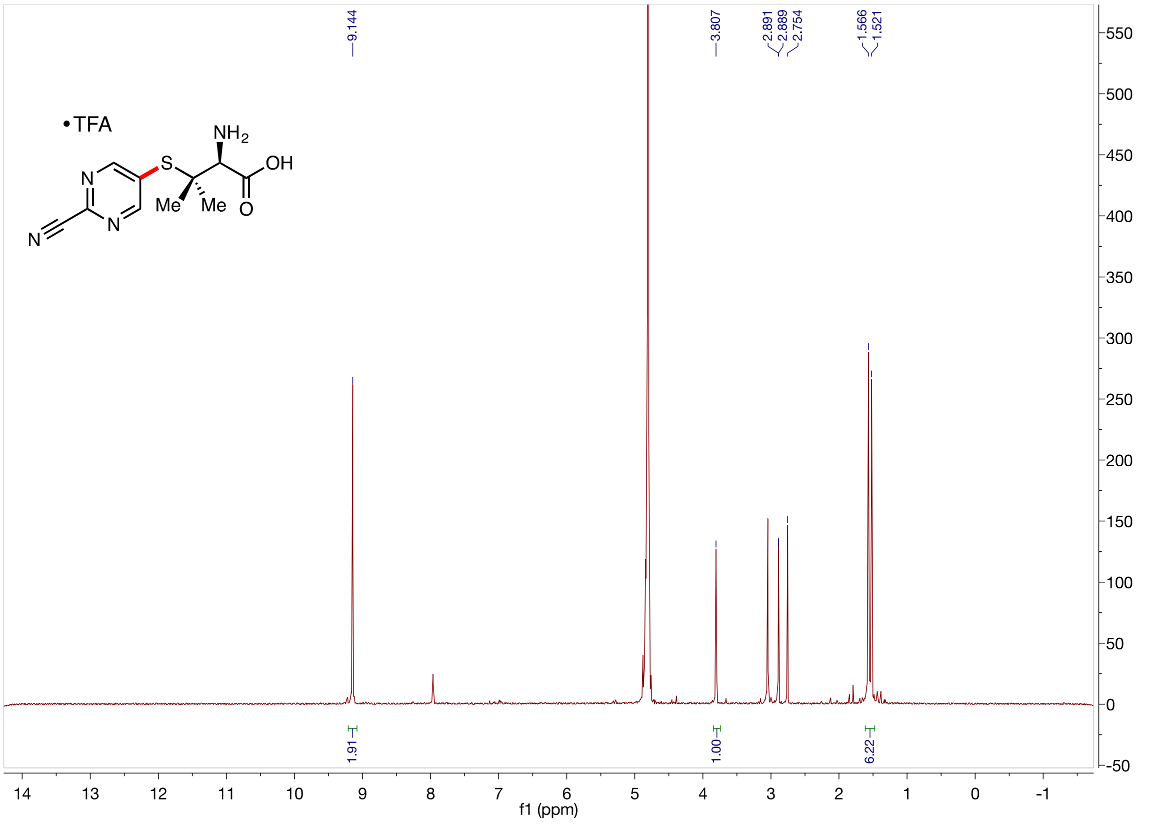

^{1}H NMR (D_2O, 500.4 MHz) of (**8b**)

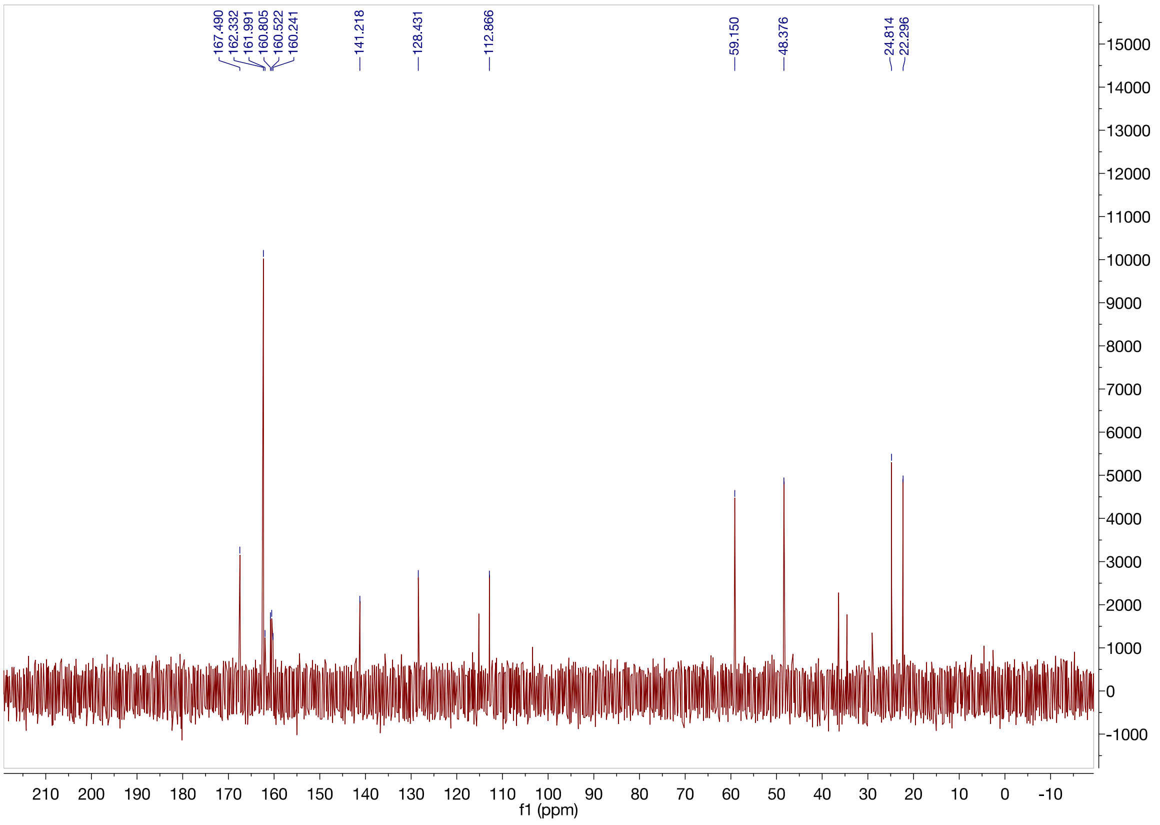

^{13}C {^{1}H} NMR (D_2O, 125.8 MHz) of (**8b**)

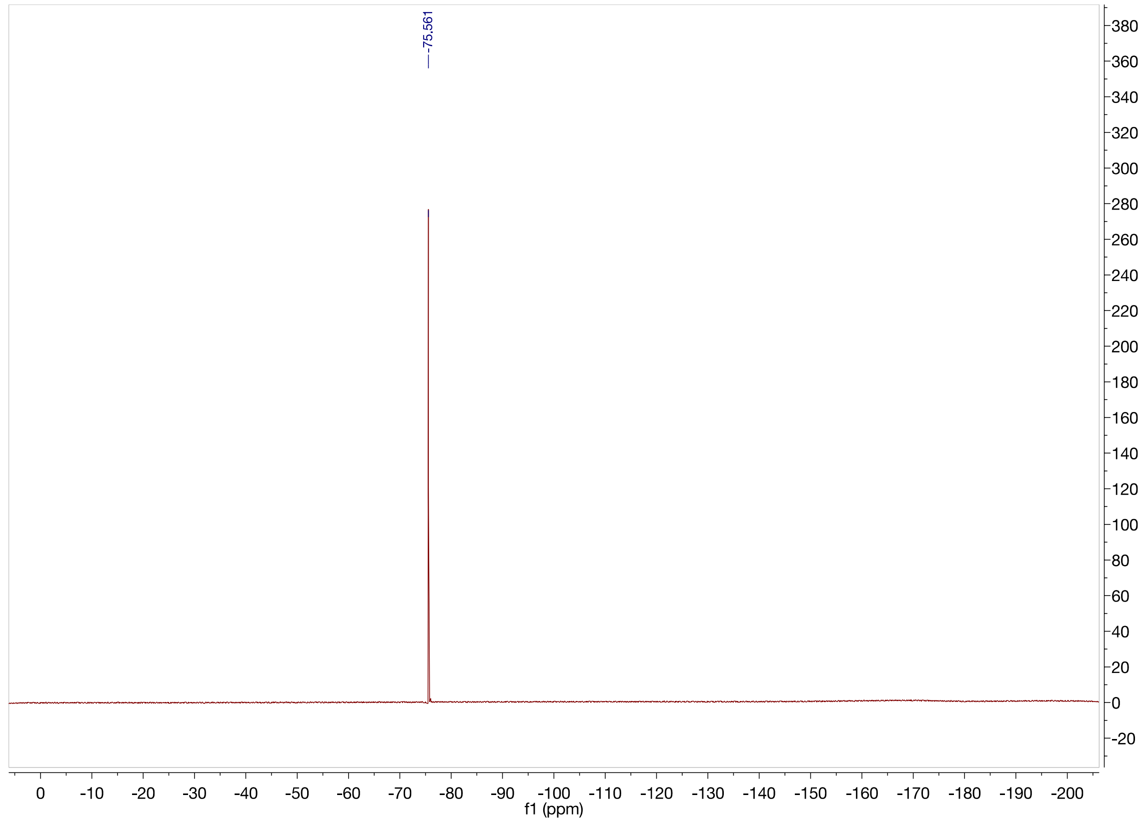

^{19}F {^{1}H} NMR (D_2O, 470.8 MHz) of (**8b**)

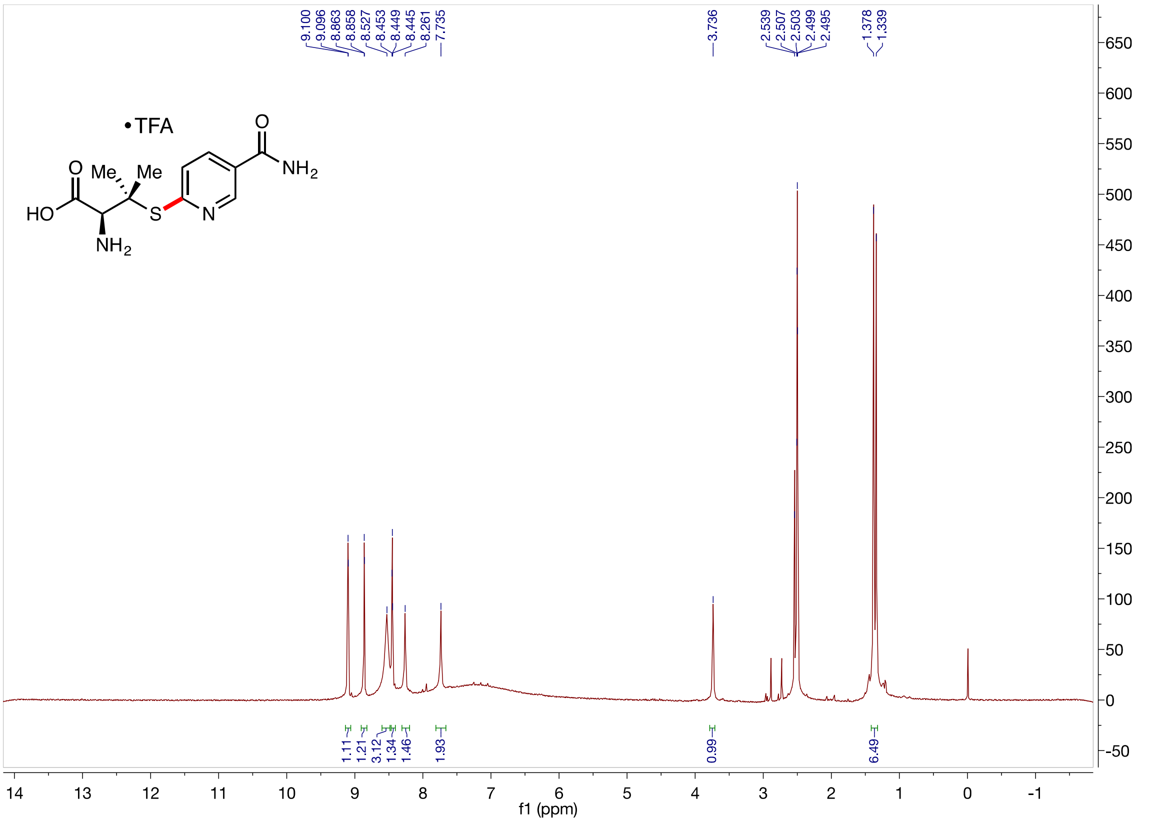

^{1}H NMR (DMSO-d_6, 500.4 MHz) of (**8c**)

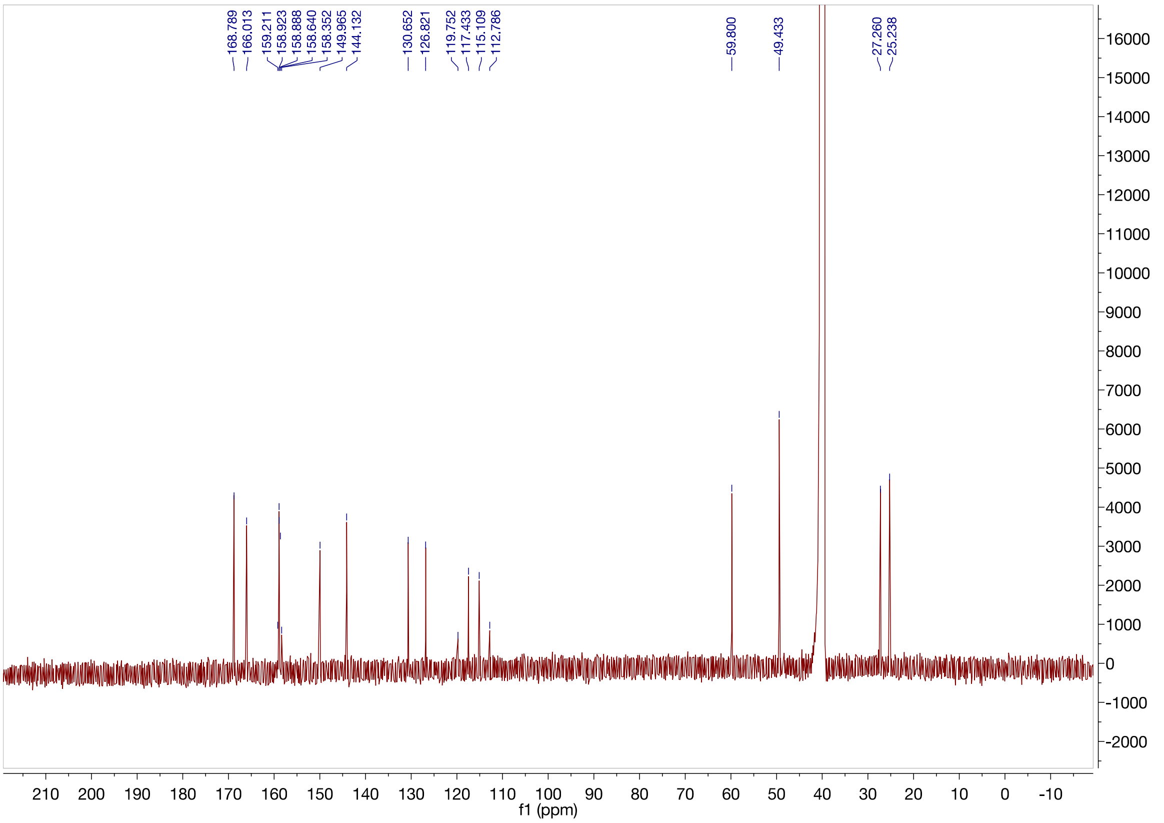

^{13}C {^{1}H} NMR (DMSO-d_6, 125.8 MHz) of (**8c**)

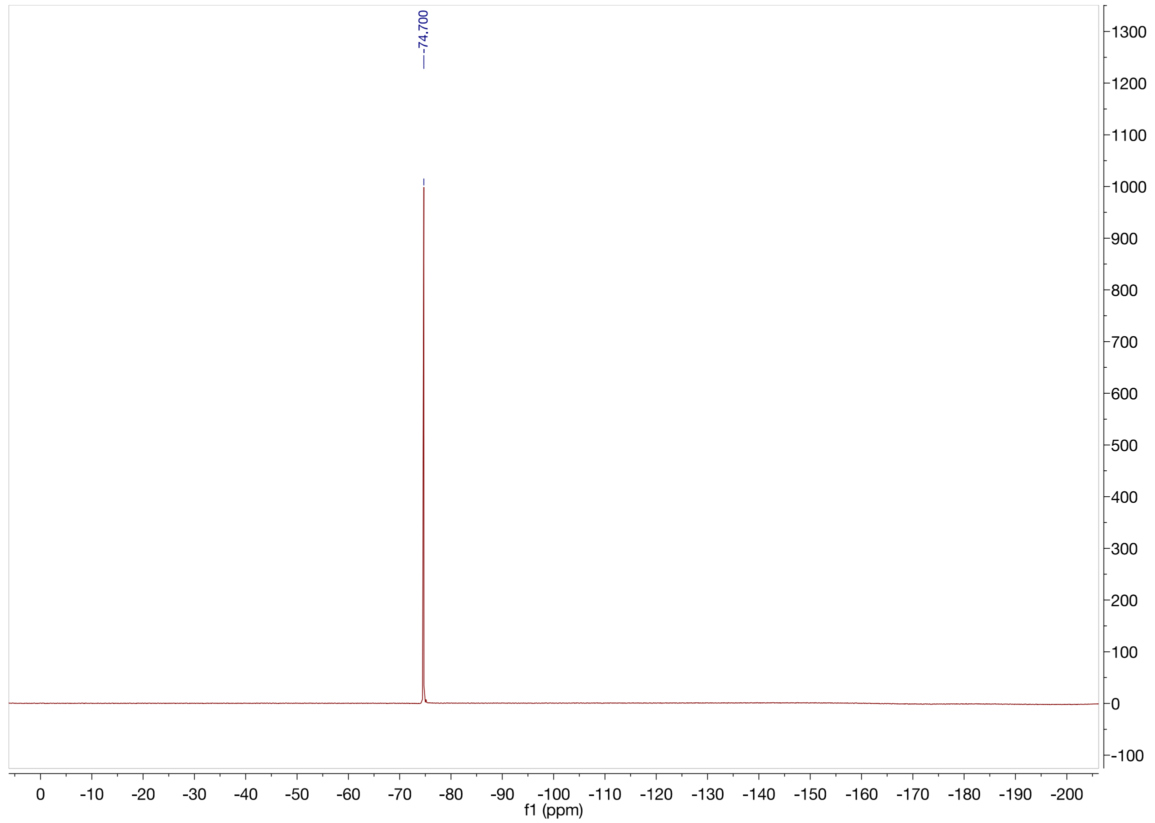

$^{19}F\ \{^{1}H\}$ NMR (DMSO-d_6, 470.8 MHz) of (**8c**)

[1] A. Gutiérrez-Bonet, J. C. Tellis, J. K. Matsui, B. A. Vara and G. A. Molander, *ACS Catal.,* 2016, **6**, 8004-8008.

[2] C. B. Kelly, N. R. Patel, D. N. Primer, M. Jouffroy, J. C. Tellis and G. A. Molander, *Nat. Protoc.,* 2017, **12**, 472-492.

[3] H. Uoyama, K. Goushi, K. Shizu, H. Nomura and C. Adachi, *Nature* 2012, **492**, 234-239.

[4] K. Lin, C. B. Kelly, M. Jouffroy and G. A. Molander, *Org. Synth*. 2017, **94**, 16-33.

[5] M. Jouffroy, C. B. Kelly and G. A. Molander, *Org. Lett.*, 2016, **18**, 876-879.

[6] E. B. Corcoran, M. T. Pirnot, S. Lin, S. D. Dreher, D. A. DiRocco, I. W. Davies, S. L. Buchwald and D. W. C. MacMillan, *Science,* 2016, **353**, 279-283.
